# EpoR stimulates rapid cycling and larger red cells during mouse and human erythropoiesis

**DOI:** 10.1038/s41467-021-27562-4

**Published:** 2021-12-17

**Authors:** Daniel Hidalgo, Jacob Bejder, Ramona Pop, Kyle Gellatly, Yung Hwang, S. Maxwell Scalf, Anna E. Eastman, Jane-Jane Chen, Lihua Julie Zhu, Jules A. A. C. Heuberger, Shangqin Guo, Mark J. Koury, Nikolai Baastrup Nordsborg, Merav Socolovsky

**Affiliations:** 1 Department of Molecular, Cell and Cancer Biology, University of Massachusetts Chan Medical School, Worcester, MA USA; 2 Department of Nutrition, Exercise and Sports, University of Copenhagen, Copenhagen, Denmark; 3 Program in Bioinformatics and Computational Biology, University of Massachusetts Chan Medical School, Worcester, MA USA; 4 Department of Cell Biology and Yale Stem Cell Center, Yale University, New Haven, CT USA; 5 Institute for Medical Engineering & Science, MIT, Cambridge, MA USA; 6 Department of Molecular Medicine, University of Massachusetts Chan Medical School, Worcester, MA USA; 7 Centre for Human Drug Research, Leiden, The Netherlands; 8 Department of Medicine, Division of Hematology and Oncology, Vanderbilt University Medical Center, Nashville, TN USA; 9 Present Address: Harvard Department of Stem Cell and Regenerative Biology, Harvard University, Cambridge, MA USA

**Keywords:** Cell division, Cell growth, Haematopoietic system

## Abstract

The erythroid terminal differentiation program couples sequential cell divisions with progressive reductions in cell size. The erythropoietin receptor (EpoR) is essential for erythroblast survival, but its other functions are not well characterized. Here we use *Epor*^*−/−*^ mouse erythroblasts endowed with survival signaling to identify novel non-redundant EpoR functions. We find that, paradoxically, EpoR signaling increases red cell size while also increasing the number and speed of erythroblast cell cycles. EpoR-regulation of cell size is independent of established red cell size regulation by iron. High erythropoietin (Epo) increases red cell size in wild-type mice and in human volunteers. The increase in mean corpuscular volume (MCV) outlasts the duration of Epo treatment and is not the result of increased reticulocyte number. Our work shows that EpoR signaling alters the relationship between cycling and cell size. Further, diagnostic interpretations of increased MCV should now include high Epo levels and hypoxic stress.

## Abstract (web summary)

Maturing erythroblasts become smaller with every cell division. Here, the authors show that Epo stimulation promotes cell division and also generates larger red cells, and that this occurs in mouse and human cells, suggesting that red cell size could be a diagnostic marker for hypoxic stress.

## Introduction

Red-cell formation (erythropoiesis) is continuous throughout life, replenishing senescent red cells and responding to increased demand during anemia, bleeding, or hypoxic stress. Anemia resulting from nutritional deficiencies, malaria, chronic disease, cancer, or hereditary hemoglobinopathies, accounts for 8.8% of all disabilities globally^1^. Erythropoietin (Epo) is the principal and essential regulator of definitive (adult-type) erythropoiesis, regulating erythropoietic rate in the basal state and during the stress response. Epo acts through its receptor, EpoR, a transmembrane type I cytokine receptor^2^, first expressed in the earliest erythroid-committed progenitors. EpoR expression peaks in colony-forming-unit-erythroid (CFU-e) progenitors^3,4^ (Supplementary Fig. 1) with the onset of erythroid terminal differentiation (ETD)^5^, a process that starts with the induction of erythroid gene transcription^4^. During ETD, erythroblasts undergo 3–5 maturational cell divisions in which they become smaller, express hemoglobin, and enucleate to form reticulocytes. EpoR rescues proerythroblasts and basophilic erythroblasts (here collectively termed ‘early erythroblasts’) and CFU-e from apoptosis^6,7^, a principal mechanism of erythropoietic rate regulation^8,9^. EpoR is downregulated in late erythroblasts, which no longer depend on its signaling for survival^10–12^ (Supplementary Fig. 1). *Epo*^*−/−*^ or *Epor*^*−/−*^ mice die on embryonic day 13 (E13) as a result of severe anemia^5,13,14^. Their fetal liver, the site of hematopoiesis at mid-gestation, contains CFU-e progenitors but is entirely devoid of cells undergoing ETD^5,13,14^.

The absolute dependence of definitive early erythroblasts on EpoR signaling for survival makes it challenging to identify other essential functions of EpoR in these cells. Key open questions include a role for EpoR in cell-cycle regulation. Although early reports suggested that Epo does not alter the erythroblast cell cycle^15^, EpoR signaling induces cell-cycle genes in these cells^16^, and is essential for the cycling of Epo-dependent cell lines^17,18^ and cultured CFU-e^19^. EpoR also promotes cycling in yolk-sac-derived primitive erythroblasts during early embryonic development^20^. Therefore, EpoR may also be required for the cycling of adult-type erythroblasts, a function that may contribute to the erythropoietic stress response.

A second open question is whether EpoR is required for induction of erythroid genes. EpoR and similar cytokine receptors do not instruct lineage choice and are instead required for essential permissive functions^21–24^. It is not clear, however, whether these include signals that facilitate erythroid gene transcription. EpoR signaling was shown to phosphorylate GATA1, a key erythroid transcriptional regulator, but the broad impact of this on GATA1 function is not clear^25^.

To address these gaps, we developed a genetic system that identifies essential non-survival functions of EpoR signaling. We rescued mouse *Epor*^*−/−*^ fetal liver progenitors from apoptosis by transduction with the anti-apoptotic protein Bcl-x_L_, and compared their ensuing differentiation with that of *Epor*^*−/−*^ progenitors that were rescued by re-introduction of the EpoR. We found that the Bcl-x_L_ survival signal, in the absence of any EpoR signaling, supported expression of the erythroid transcriptional program and formation of enucleated red cells. However, key ETD features were abnormal. First, erythroblasts underwent slower and fewer cell cycles, suggesting a cell-cycle role for EpoR. We confirmed this role in adult mice in vivo, finding that Epo administration shortened the cycle of early erythroblasts, cells that are already amongst the fastest cycling cells in the bone marrow^26–28^. Second, we found that, unexpectedly, despite stimulating rapid cycling, EpoR signaling increases cell size in both erythroblasts and red cells. This contrasts with the well-established inverse relationship between the number of erythroblast cell divisions and red-cell size^29–32^. Using mice doubly deleted for both EpoR and HRI, we found that EpoR regulation of red-cell size is also independent of the well-described iron and heme-regulated pathway^33–35^. We confirmed these findings in healthy human volunteers that were administered Epo, finding an increased MCV that persisted long after Epo and reticulocyte levels returned to baseline. Our work reveals novel EpoR functions, and suggests hypoxia, anemia, and other high-Epo syndromes as new diagnostic interpretations of increased red-cell size in the clinic.

## Results

### Non-survival EpoR signals are essential for normal erythroid differentiation

**Fig. 1:**
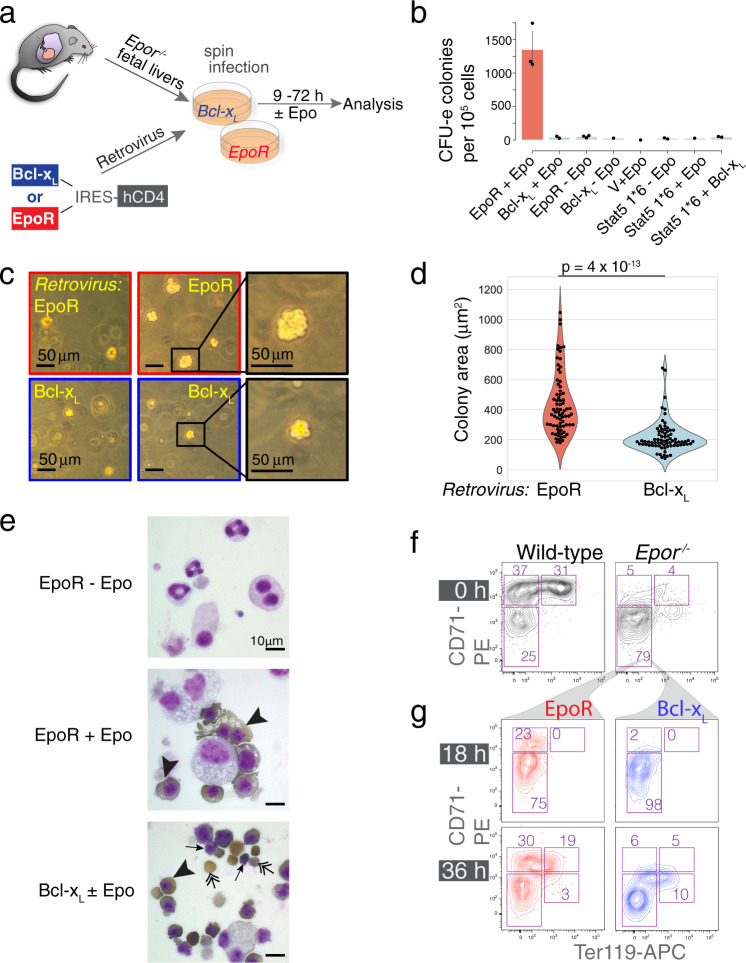
Abnormal ETD in the absence of EpoR signaling. **a** Experimental design. E12.5 *Epor*^*−/−*^ fetal livers were transduced with bicistronic retroviral vectors encoding either Bcl-x_L_ or EpoR, linked by an internal ribosomal entry site (IRES) to human CD4 (hCD4) or GFP reporters. Transduced cells differentiated in vitro into red cells over the ensuing 72 h. **b** *Epor*^*−/−*^ CFU-e colonies, scored 48 h following transduction with either EpoR or Bcl-x_L_. Epo was added to the medium where indicated. *Epor*^*−/−*^ fetal liver cells were also transduced with retroviral vectors encoding the following: ‘empty’ vector (‘V’), constitutively active Stat5 (Stat5 1*6), or doubly transduced with both Bcl-x_L_ and Stat5 1*6. Data pooled from 3 independent experiments. Data are means ± SD. Only CFU-e colonies of a size comparable to those of wild-type colonies were scored. **c** Representative colonies from an experiment as in (**b**). **d** Colony area occupied by each of 75 colonies for each genotype (EpoR-*Epor*^*−/−*^ or Bcl-x_L_-*Epor*^*−/−*^). Data pooled from 3 independent experiments as in (**b**). Two-sided *t* test, unequal variance. **e** Cytospin preparations of transduced *Epor*^−/−^ fetal liver cells cultured in liquid medium for 36 h, in the presence or absence of Epo as indicated. Cells were stained for hemoglobin with diaminobenzidine (brown stain, arrowheads) and counter-stained with Giemsa. Representative of 4 independent experiments. Double-headed arrows point at enucleated red cells; arrows point at pyrenocytes (extruded nuclei). The micrograph in the bottom panel is representative of cultures both in the presence or absence of Epo. **f**, **g** Flow-cytometric CD71/Ter119 profiles of freshly harvested *Epor*^*−/−*^ and wild-type littermate fetal livers (**f**), and of *Epor*^*−/−*^ fetal liver cells 18 and 36 h post transduction and culture in Epo-containing medium (**g**).

Erythroid differentiation in *Epor*^*−/−*^ fetal liver is arrested at the CFU-e stage^5,13,14,20^. *Epor*^*−/−*^ CFU-e can be rescued in vitro by transduction with EpoR or a similar cytokine receptor^5,22^. Here we asked whether transducing *Epor*^*−/−*^ CFU-e with Bcl-x_L_, an anti-apoptotic transcriptional target of EpoR signaling^36–39^, would be sufficient to support erythroid differentiation. As control, we transduced *Epor*^*−/−*^ cells from the same fetal livers with the EpoR. The use of bicistronic retroviral expression vectors allowed us to track transduced cells (Fig. 1a).

As expected, *Epor*^*−/−*^ cells transduced with ‘empty’ vector failed to give rise to CFU-e-derived colonies in semi-solid medium, whereas EpoR-transduced *Epor*^*−/−*^ cells (EpoR-*Epor*^*−/−*^) generated CFU-e colonies in an Epo-dependent manner. Bcl-x_L_-transduced *Epor*^*−/−*^ cells (Bcl-x_L_-*Epor*^*−/−*^) failed to give rise to CFU-e colonies of the usual size and appearance (Fig. 1b). Instead, they generated a similar number of much smaller colonies with fewer cells (colony areas were 439 ± 208 μm^2^ versus 217 ± 106 μm^2^, mean ± SD, for EpoR-*Epor*^*−/−*^ v. Bcl-x_L_-*Epor*^*−/−*^, *p* = 3.6 × 10^−13^; Fig. 1c, d). Co-transduction of *Epor*^*−/−*^ cells with both Bcl-x_L_ and a constitutively active form of Stat5, an EpoR-activated transcription factor, was also not sufficient to support the formation of normally-sized *Epor*^*−/−*^ CFU-e colonies (Fig. 1b).

Liquid cultures of Bcl-x_L_-*Epor*^*−/−*^ in the presence or absence of Epo, and of EpoR-*Epor*^*−/−*^ erythroblasts with Epo, contained hemoglobinized cells by 36 h post transduction, while EpoR-*Epor*^*−/−*^ erythroblasts without Epo did not (Fig. 1e). However, differentiation of Bcl-x_L_-*Epor*^*−/−*^ erythroblasts appeared to be accelerated, with cultures containing smaller and morphologically more mature erythroblasts, including many enucleated cells; there were few if any enucleated cells in cultures of EpoR-*Epor*^*−/−*^ erythroblasts at this time (Fig. 1e).

Differentiation abnormalities of Bcl-x_L_-*Epor*^*−/−*^ erythroblasts were also evident from flow cytometric analysis. In wild-type progenitors, the transition from the CFU-e stage to ETD is marked by sharp upregulation of CD71 (encoded by the transferrin receptor, *Tfrc*), followed by upregulation of Ter119^4,26,40^. *Epor*^*−/−*^ progenitors arrest in development prior to CD71 upregulation (the small number of Ter119^+^ cells in *Epor*^*−/−*^ fetal liver are yolk-sac-derived erythroblasts^40^, Fig. 1f). Transduction of *Epor*^*−/−*^ fetal liver cells with EpoR allowed them to resume the expected sequence of cell surface marker expression, upregulating CD71 by 18 h and Ter119 by 36 h (Fig. 1g). By contrast, Bcl-x_L_-*Epor*^*−/−*^ cells failed to upregulate CD71 at any point of the culture although they did upregulate Ter119 (Fig. 1g).

Thus, our initial analysis showed that, when rescued from apoptosis by Bcl-x_L_, *Epor*^*−/−*^ progenitors can differentiate into hemoglobinized, enucleated red cells in the absence of additional EpoR signals. However, their ETD is abnormal, failing to upregulate CD71, and differentiating prematurely into fewer and smaller red cells.

### Erythroblasts undergo fewer and slower cell cycles in the absence of EpoR signaling

**Fig. 2:**
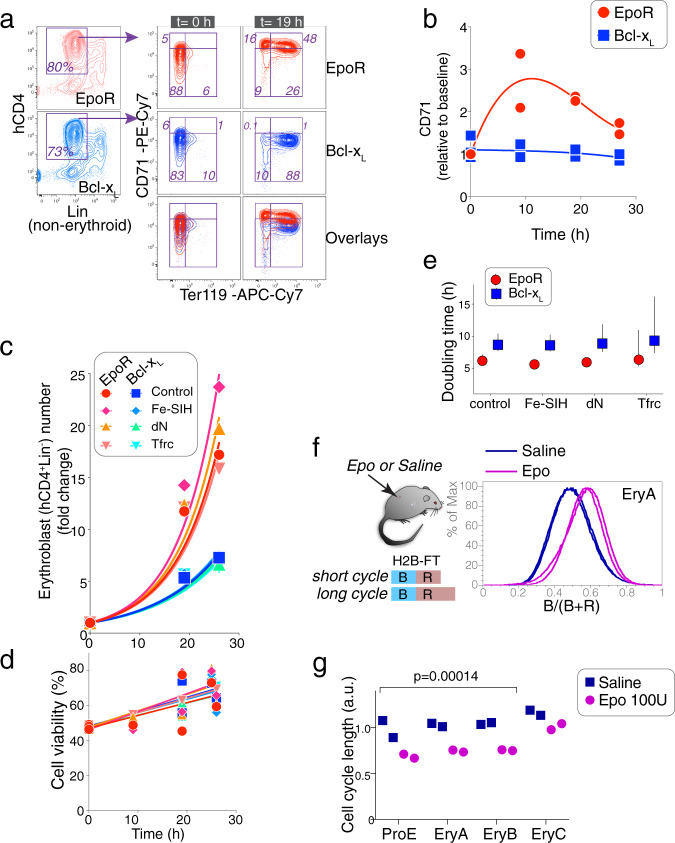
EpoR stimulates cell-cycle shortening in early erythroblasts in vitro and in vivo. **a** The EpoR is required for CD71 upregulation. *Epor*^*−/−*^ fetal livers were transduced with either EpoR or Bcl-x_L_ retroviral vectors carrying the hCD4 reporter (see Supplementary Fig. 2b for experimental design). Transduced cells (hCD4^+^Lin^−^) were examined for expression of CD71 and Ter119. **b** Time course of CD71 expression following transduction as in (**a**). MFI; median fluorescence intensity, relative to *t* = 0; *n* = 2 independent experiments. **c** Growth of *Epor*^*−/−*^ fetal liver cells transduced with either Bcl-x_L_ or EpoR. Viable hCD4^+^Lin^−^ cells were counted at the indicated times. Fe-SIH (10 μM) or deoxynucleosides (dN, 0.7 μM) were added to the medium as indicated. ‘Tfrc’ cells were doubly transduced with both *Tfrc*, and either *Epor* or *Bcl-x*_*L*_. Data, pooled from *n* = 4 independent experiments and expressed relative to cell number at *t* = 0, were fit with exponential curves (*R*^2^ values 0.8–0.94, least-squares fit). **d** Trypan blue negative cells, for the set of experiments in (**c**). **e** Cell doubling times ±95% confidence intervals, calculated from the fitting of exponential growth curves to the data in (**e**). **f** Cell-cycle shortening in early erythroblasts in vivo. Mice transgenic for the chimeric histone H2B-fluorescence-timer protein (H2B-FT) were injected with either saline or Epo (100 U) at 0 and 24 h. Bone marrow was analyzed at 36 h. H2B-FT fluoresces blue (‘B’) for 1–2 h immediately following synthesis, and matures into a red fluorescent protein (‘R’). The ratio of blue to total fluorescence (*B*/(*B* + *R*)) is a function of cell-cycle length^27^. Shown are histograms of *B*/(*B* + *R*) in EryA erythroblasts (Ter119^high^CD71^high^FSC^high^). Histogram overlays are for *n* = 2 mice injected with either saline or Epo. **g** Relative cell-cycle lengths for the 4 mice analyzed in (**f**), for each of the indicated erythroblast maturation stages: ProE (Ter119^med^CD71^high^), EryA (Ter119^high^CD71^high^FSC^high^), EryB (Ter119^high^CD71^high^FSC^low^), EryC (Ter119^high^CD71^low^FSC^low^). *p*-value is for a two-tailed paired *t* test, pairing Epo-injected and Saline-injected mice for each erythroblast stage (ProE and EryA/B/C). Late erythroblasts (EryC) may divide, but their cell cycle is no longer sensitive to Epo concentration.

CFU-e express the receptor tyrosine kinase Kit and the Interleukin-3 (IL3) receptor^22,41,42^. Addition of stem cell factor (SCF, the Kit ligand) and IL3 to the media increased the overall yield of transduced *Epor*^*−/−*^ fetal liver cells, but the difference in cell number between Bcl-x_L_-*Epor*^*−/−*^ and EpoR-*Epor*^*−/−*^ erythroblasts remained (Supplementary Fig. 2a). We modified our transduction protocol to make use of this improvement in yield, culturing freshly transduced *Epor*^*−/−*^ progenitors for 15 h in SCF and IL3 before transitioning the cells to an Epo-containing medium for the remainder of differentiation. Since SCF and IL3 also promote the growth of myeloid cells, all analysis was performed on cells that were both negative for non-erythroid lineage markers and positive for reporters of transduction (hCD4 and/or GFP, Supplementary Fig. 2b, Fig. 2a).

Pre-incubation with SCF and IL3 did not ameliorate the abnormalities of Bcl-x_L_-*Epor*^*−/−*^ erythroblast differentiation. In particular, these cells failed to upregulate CD71 (Fig. 2a, b). We examined the possibility that these abnormalities were the result of overexpression of Bcl-x_L_, rather than the absence of EpoR signaling, by co-transducing *Epor*^*−/−*^ progenitors with both EpoR and Bcl-x_L_, each linked to a distinct reporter (Supplementary Fig. 2c). The doubly transduced progenitors were indistinguishable from cells transduced with only the EpoR, indicating that the lower cell number and failure to upregulate CD71 were not the result of Bcl-x_L_ overexpression, but rather, of absent EpoR signaling (Supplementary Fig. 2d-g).

The transferrin receptor is critical for iron import into erythroid cells. Iron deficiency leads to microcytic anemia. We therefore examined whether iron deficiency might account for the abnormal differentiation of Bcl-x_L_-*Epor*^*−/−*^ erythroblasts, by co-transducing *Epor*^*−/−*^ progenitors with *Tfrc*, in addition to either Bcl-x_L_ or EpoR (Fig. 2c–e). In an alternative approach, we added iron-loaded ferric-salicylaldehyde isonicotinoyl hydrazone (Fe-SIH) to the culture medium of both Bcl-x_L_-*Epor*^*−/−*^ and EpoR-*Epor*^*−/−*^ erythroblasts. SIH is a cell-membrane-permeable synthetic iron chelate, which, when pre-loaded with iron, will deliver iron intracellularly for heme synthesis, bypassing defects in Tfrc iron transport in erythroid cells^43^. Neither of these approaches altered the proliferative defect of Bcl-x_L_-*Epor*^*−/−*^ erythroblasts (Fig. 2c, e). The viability of all erythroblasts was high with no significant difference between Bcl-x_L_-*Epor*^*−/−*^ and EpoR-*Epor*^*−/−*^ erythroblasts (Fig. 2d), suggesting that the proliferative deficit of Bcl-x_L_-*Epor*^*−/−*^ erythroblasts is the result of fewer cell divisions. In the first 26 h of culture, there was a substantial difference in doubling time (6.1 h v. 8.6 h for EpoR-*Epor*^*−/−*^ v. Bcl-x_L_-*Epor*^*−/−*^ erythroblasts, Fig. 2c, e). The doubling time of 6 h for EpoR-*Epor*^*−/−*^ is in good agreement with our recent finding of a 6 h cell cycle in wild-type early erythroblasts in vivo^26^, and with the finding that early erythroblasts have the shortest cell cycle amongst bone-marrow hematopoietic progenitors^27^.

Iron may affect cell growth by acting as a cofactor in ribonucleotide reductase (RNR) catalysis of deoxyribonucleotide synthesis^44^. However, supplementation of the culture medium with deoxyribonucleosides (dN), which bypass RNR via the deoxyribonucleoside kinase salvage pathway^45^, had little effect on the proliferative defect (Fig. 2c, e). Taken together, in the absence of EpoR signaling, erythroblasts fail to upregulate CD71 and also undergo fewer and longer cell divisions. Supplementation with iron or deoxyribonucleosides does not rescue these deficits.

### Epo administration shortens cell-cycle duration in early erythroblasts in vivo

To test whether EpoR stimulation alters cell-cycle length in vivo, we used a mouse transgenic for histone H2B fused to a fluorescent timer protein (H2B-FT, Fig. 2f), which fluoresces blue when first synthesized but matures over 1–2 h into a red fluorescent protein^27^. The ratio of blue fluorescence to total fluorescence (red + blue) is an indicator of cell-cycle length^27^. Administration of Epo (100 U) once daily resulted in a clear shift in the ratio of blue to total fluorescence at 36 h, in all bone-marrow early erythroblast subsets (Fig. 2f, g and Supplementary Fig. 3). These data confirm that Epo/EpoR signaling increases cell-cycle speed in wild-type erythroblasts in vivo.

### EpoR shortens both G1 and S phase through an iron-independent mechanism

The onset of ETD is associated with cell-cycle shortening, from ~15 h in CFU-e, to 6 h in early erythroblasts^4,26,40^, including a shortened, 4-h-long S phase^26^. We asked whether the cell-cycle shortening effect of EpoR (Fig. 2e, g) is exerted in G1 or in S phase. The shortening of G1 by cytokine receptor signaling is well documented^46–50^. However, to our knowledge, there are no reports of cytokine signaling altering S phase speed.

**Fig. 3:**
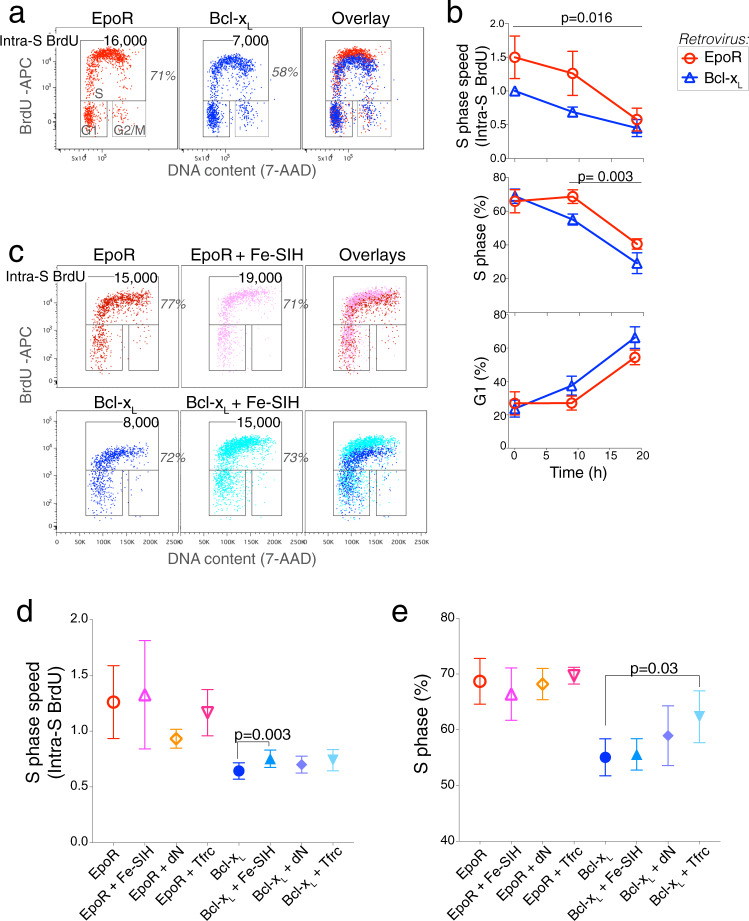
EpoR regulates the speed of S phase. **a** Cell-cycle analysis of *Epor*^*−/−*^ fetal liver cells transduced with either EpoR or Bcl-x_L_ and cultured as in Supplementary Fig. 2b. Cells were pulsed with BrdU for 30 min at *t* = 9 h and were immediately harvested for analysis. The fraction (%) of erythroblasts (hCD4^+^Lin^−^) in S phase is indicated, as is S phase speed, measured as the intra-S phase rate of BrdU incorporation (BrdU MFI within the S phase gate). **b** Summary of cell-cycle status and S phase speed, as measured by intra-S phase BrdU incorporation in EpoR or Bcl-x_L_-transduced *Epor*^*−/−*^ fetal liver cells. Data is pooled from 6 independent experiments similar to (**a**). In all cases, cells were pulsed with BrdU for 30 min prior to harvesting for analysis. Data are mean ± sem. Intra-S phase BrdU (MFI) is expressed as the ratio to BrdU MFI of Bcl-x_L_-transduced fetal liver cells at *t* = 0 in each experiment. Significance *p*-values are two-tailed paired *t* test, pairing EpoR and Bcl-x_L_-transduced cells for each time point across all experiments (upper panel), and for *t* = 9 and *t* = 19 h in all experiments (middle and lower panels). **c** Effect of the cell-permeable iron carrier, Fe-SIH (10 μM) on S phase speed. Experiment and cell-cycle analysis as in (**b**). Cells were harvested at *t* = 9 h. **d**, **e** Summary of S phase speed (**d**) and cell-cycle status (**e**) in EpoR and Bcl-x_L_-transduced *Epor*^*−/−*^ fetal liver cells at *t* = 9 h, experimental design as in Fig. 2b, and (**a**) to (**c**) above. S phase speed is expressed relative to the speed at *t* = 0 in each experiment. Shown are the effects of adding Fe-SIH or dN to the medium, or of doubly transducing cells with both Bcl-x_L_ and Tfrc. Data are mean ± sem for *n* = 4 independent experiments each for Fe-SIH and dN, and *n* = 3 for *Tfrc*. All experiments also had *Epor*^*−/−*^ fetal liver cells transduced with EpoR and with Bcl-x_L_ without additional additives or transductions. *P*-value is for a two-sided *t* test, unequal variance.

To examine this, we pulsed cultures of EpoR or Bcl-x_L_-transduced *Epor*^*−/−*^ erythroblasts with bromodeoxyuridine (BrdU), a nucleoside analog that is incorporated into DNA during S phase, and analyzed the cells 30 min following the pulse. The fraction of cells that are labeled with an anti-BrdU antibody indicates the proportion of cells in S phase at the time of the pulse. Further, the amount of BrdU incorporated into S phase cells during the 30 min pulse, as measured by the BrdU mean fluorescence intensity (MFI) of S phase cells, indicates the intra-S phase rate of DNA synthesis, which is inversely related to S phase duration^26^. We found that, in the first 10 h of ETD, BrdU MFI in S phase cells was 50% higher in EpoR-*Epor*^*−/−*^, compared with Bcl-x_L_-*Epor*^*−/−*^ erythroblasts, suggesting that EpoR signaling increases S phase speed (Fig. 3a, b).

If the slowing of S phase alone could account for the increased cell-cycle length of Bcl-x_L_-*Epor*^*−/−*^ erythroblasts, S phase would constitute a larger fraction of total cell-cycle duration. However, the fraction of Bcl-x_L_-*Epor*^*−/−*^ erythroblasts in S phase was actually somewhat lower, with a corresponding increase in the fraction of cells in G1 (Fig. 3b), and little change in the fraction of cells in G2 or M (not shown). These observations suggest that, in the absence of EpoR signaling, both S and G1 phases lengthen.

Supplementing the culture medium with Fe-SIH increased S phase speed modestly in all *Epor*^*−/−*^ erythroblasts (Fig. 3c). There was no rescue of S phase speed in Bcl-x_L_-*Epor*^*−/−*^ erythroblasts by either the addition of deoxyribonucleosides or double transduction with both Bcl-x_L_ and Tfrc (Fig. 3d), although there was a small increase in the number of cells in S phase in the latter (Fig. 3e). Taken together, these results indicate that EpoR is essential for accelerating both G1 and S phases of the cycle in early ETD, via mechanisms that are largely independent of iron and the nucleotide pool.

### Imaging flow cytometry shows *Epor*^*−/−*^ erythroblasts and reticulocytes are smaller

**Fig. 4:**
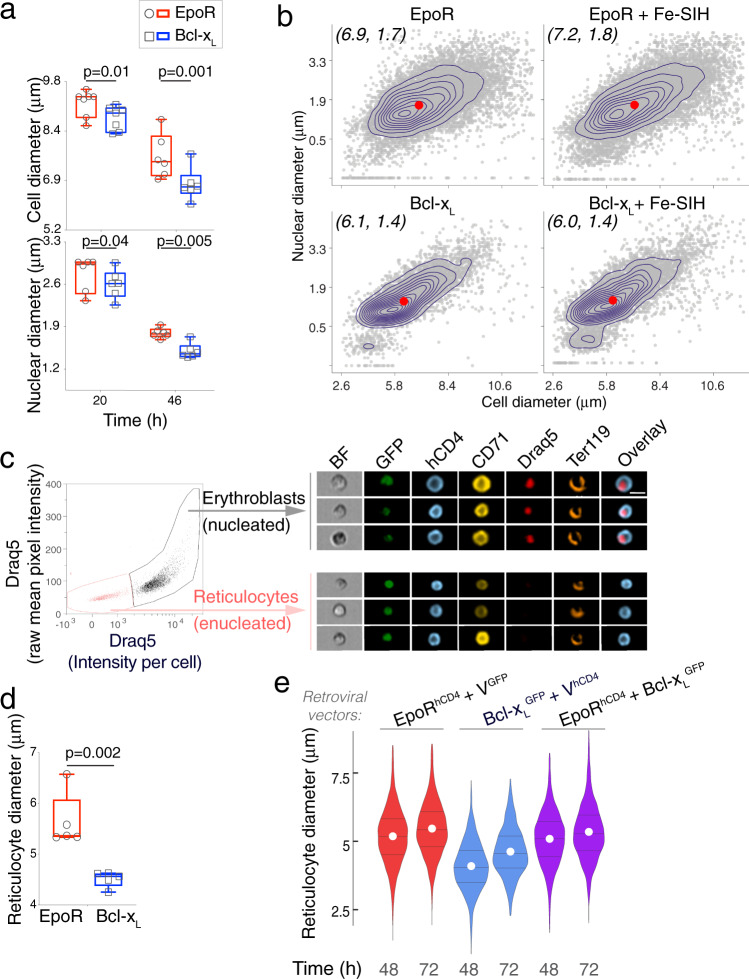
Smaller erythroblasts that differentiate into smaller reticulocytes in the absence of EpoR. **a** Cell and nuclear diameter of hCD4^+^Lin^−^ erythroblasts, measured by imaging flow cytometry. Experiment as in Fig. 2b. Polystyrene beads of known diameters were used for calibration (see “Methods”, Supplementary Fig. 4a). Datapoints are population medians for individual samples, with 50,000 cells imaged per sample. Box and whiskers mark the 25th to 75th percentiles and min to max values, respectively, with the median indicated. Data pooled from 7 independent experiments, *p*-values are from two-tailed paired *t*-tests, pairing EpoR and Bcl-x_L_-transduced cells in each experiment. **b** A representative experiment as in (**a**), showing individual sample contour plots overlaid on scatter plots (each dot is one cell), of nuclear diameter vs. cell diameter. Red dots indicate distributions’ medians. The effect of adding Fe-SIH to the culture medium is also shown. Data are hCD4^+^Lin^−^ erythroblasts at 48 h post transduction. **c** Distinguishing erythroblasts from reticulocytes using imaging flow cytometry, with the nuclear dye Draq5. The analysis was performed on Ter119^+^ cells. Representative images of 3 independent experiments are shown, from cultures of *Epor*^*−/−*^ fetal liver cells that were doubly transduced with bicistronic retroviral vectors encoding GFP and hCD4 reporters (see Supplementary Fig. 2c), at 48 h post transduction. Scale bar = 10 µm. **d** Reticulocyte cell diameter in cultures of EpoR*-Epor*^*−/−*^ or Bcl-x_L_-*Epor*^*−/−*^ at 48 h post transduction, identified as in (**c**). Data are population medians from 5 independent experiments. Box and whiskers as in (**a**). Two-tailed paired *t*-tests. **e** Reticulocyte diameters in cultures of *Epor*^*−/−*^ fetal liver cells that were doubly transduced with bicistronic vectors carrying GFP and hCD4 reporters (Supplementary Fig. 2c). These vectors were either ‘empty’ (V^GFP^, V^hCD4^) or encoded either Bcl-x_L_ or EpoR (Bcl-x_L_^GFP^, EpoR^hCD4^). Violin lines mark the 25th, 50th, and 75th percentile with a white circle marking the mean. Representative of two independent experiments.

Nutritional deficiencies, drugs, or genetic perturbations that reduce the number of cell divisions lead to the formation of larger red cells (macrocytosis^29–32,51^). Therefore, we expected that the fewer cell divisions of Bcl-x_L_-*Epor*^*−/−*^ erythroblasts would result in larger size for these cells. Instead, they appeared to be smaller (Fig. 1e). To address this question quantitatively, we measured cell and nuclear size in EpoR-*Epor*^*−/−*^ and Bcl-x_L_-*Epor*^*−/−*^ erythroblasts by imaging flow cytometry (Fig. 4a, b). We calibrated the measured cell areas by comparing them with beads of known diameter (Supplementary Fig. 4a). We found that both cell and nuclear size were significantly smaller in the absence of EpoR (7.5 ± 0.6 μm and 6.7 ± 0.7 μm for EpoR-*Epor*^*−/−*^ and Bcl-x_L_-*Epor*^*−/−*^ erythroblasts, respectively, mean ± sem, *p* = 0.001, *t* = 46 h). Although Bcl-x_L_-*Epor*^*−/−*^ erythroblasts express significantly lower CD71 (Fig. 2a, b), the addition of Fe-SIH to the culture did not alter their smaller cell or nuclear size (Fig. 4b).

We asked whether the smaller size of Bcl-x_L_-*Epor*^*−/−*^ erythroblasts could reflect an accelerated process of differentiation. If at any given time of the culture Bcl-x_L_-*Epor*^*−/−*^ erythroblasts were smaller only as a result of being at a more advanced maturation stage, they should give rise to normally-sized enucleated reticulocytes, albeit at an earlier time. However, imaging flow cytometry showed that Bcl-x_L_-*Epor*^*−/−*^ reticulocytes were significantly smaller (5.6 ± 0.5 μm vs. 4.5 ± 0.15 μm for of EpoR-*Epor*^*−/−*^ vs. Bcl-x_L_-*Epor*^*−/−*^, mean ± sem, *p* = 0.002, Fig. 4c, d).

To assess whether the smaller size of Bcl-x_L_-*Epor*^*−/−*^ erythroblasts is the result of overexpression of Bcl-x_L_, rather than absent EpoR signaling, we doubly transduced *Epor*^*−/−*^ fetal liver cells with both EpoR and Bcl-x_L_. We used the Bcl-x_L_-linked GFP and the EpoR-linked hCD4 fluorescence reporters to quantify expression and ensured that all comparisons were made between cells expressing similar levels of each retroviral vector (Supplementary Fig. 4b–d). We found that erythroblasts and reticulocytes transduced with both EpoR and Bcl-x_L_ were similar in size to those transduced with only the EpoR, and significantly larger than those transduced with only Bcl-x_L_ (Fig. 4e; Supplementary Fig. 4c, d). Therefore, Bcl-x_L_ overexpression is not the cause of the smaller size of Bcl-x_L_-*Epor*^*−/−*^ erythroblasts and reticulocytes.

The level of EpoR expression in transduced *Epor*^*−/−*^ cells positively correlated with erythroblast cell diameter (Supplementary Fig. 5). The relationship follows classical dose/response kinetics (Spearman correlation = 0.97, *p*-value = 0.004). By contrast, there was no correlation between Bcl-x_L_ expression and cell diameter.

### EpoR regulation of red-cell size is independent of HRI

HRI is activated by iron and heme deficiency and mediates the formation of smaller, hypochromic red cells, by inhibiting translation^52^. Bcl-x_L_-*Epor*^*−/−*^ erythroblasts failed to upregulate CD71 (Tfrc), the principal iron transporter. Although iron supplementation did not rescue the smaller size of Bcl-x_L_-*Epor*^*−/−*^ erythroblasts (Fig. 4b), it remained possible that intracellular iron delivery was somehow incomplete.

**Fig. 5:**
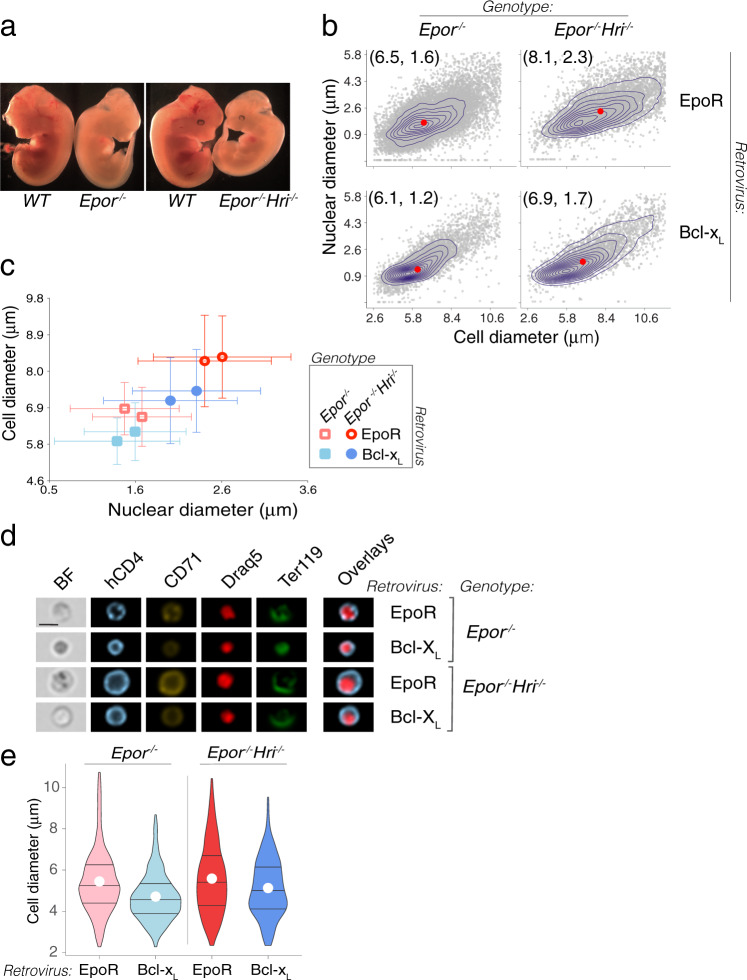
EpoR regulates cell size independently of HRI. **a** *Epor*^*−/−*^ and doubly deleted *Epor*^*−/−*^*Hri*^*−/−*^ E12.5 embryos with wild-type littermates. **b** Cell and nuclear diameters in fetal livers from either *Epor*^*−/−*^ or *Epor*^*−/−*^*Hri*^*−/−*^ embryos, transduced with either EpoR or Bcl-x_L_, at *t* = 48 h post transduction. Individual sample contour plots are overlaid on scatter plots (each dot is one cell). Red dots indicate distributions’ medians. **c** Summary data for cell and nuclear area, for two independent experiments as in (**b**), each containing all 4 genotype/retrovirus combinations. Data are mean ± SD for each cell population. Each transduced population consisted of pooled fetal liver cells from either *Epor*^*−/−*^ or *Epor*^*−/−*^*Hri*^*−/−*^ embryos. Cell diameter data for all genotypes is significantly different (*p* = 0.0008, one-way ANOVA between population means); On the *Hri*^*−/−*^ *Epor*^*−/−*^ background, cell diameter is significantly different between Bcl-x_L_ and EpoR-transduced cells (*p* = 0.019, one-way ANOVA from two independent experiments). **d** Imaging flow cytometry of representative Lin^−^hCD4^+^Ter119^+^ erythroblasts from each of the genotype/retroviral combinations at *t* = 48 h. Representative of at least 3 independent experiments. Scale bar = 10 µm. **e** *Epor*^*−/−*^ and *Epor*^*−/−*^*Hri*^*−/−*^ reticulocyte cell diameter, from cultures transduced with either EpoR or Bcl-x_L_. Representative of 2 experiments. Violin lines mark the 25th, 50th, and 75th percentile with a white circle marking the mean.

To determine definitively the relevance of the iron/heme/HRI pathway to cell size regulation by EpoR, we generated *Epor*^*−/−*^*Hri*^*−/−*^ mice (Fig. 5a). Similar to *Epor*^*−/−*^ mice, *Epor*^*−/−*^*Hri*^*−/−*^ mice died at mid-gestation with severe anemia. We rescued both *Epor*^*−/−*^ and *Epor*^*−/−*^*Hri*^*−/−*^-fetal liver cells in parallel, by transduction with either Bcl-x_L_ or EpoR (Fig. 5b–e). In agreement with the known role of HRI as a negative regulator of erythroblast size, both Bcl-x_L_-transduced and EpoR-transduced erythroblasts were larger on the *Epor*^*−/−*^*Hri*^*−/−*^ genetic background than on the *Epor*^*−/−*^ background. Importantly, for a given genetic background, either *Epor*^*−/−*^*Hri*^*−/−*^ or *Epor*^*−/−*^, the difference in size between Bcl-x_L_ and EpoR-rescued cells remained (Fig. 5b–e). These results clearly show that EpoR signaling regulates cell size independently of the HRI pathway, since, even in the absence of HRI, EpoR signaling promotes the formation of larger erythroblasts (Fig. 5b–d) and reticulocytes (Fig. 5e).

### Accelerated maturation in the absence of EpoR, assessed independently of cell size

Cell size is frequently used as indicator of erythroid maturational stage^53–56^. Our initial impression was that Bcl-x_L_-*Epor*^*−/−*^ erythroblasts completed their maturation sooner than EpoR-*Epor*^*−/−*^ erythroblasts (Fig. 1e, g). However, the finding that Bcl-x_L_-*Epor*^*−/−*^ erythroblasts are smaller throughout maturation makes cell size an unreliable indicator of maturational stage in these cells. We therefore assessed maturation using two alternative measures.

First, the cell surface marker Ter119, whose expression increases with maturation^53–56^, reached significantly higher levels in Bcl-x_L_-*Epor*^*−/−*^ erythroblasts than in EpoR-*Epor*^*−/−*^ erythroblasts at 48 h (Supplementary Fig. 6a). Second, we looked at nuclear offset, a quantitative measure of nuclear eccentricity that is independent of cell size^56^ (Supplementary Fig. 6b–d). The nuclear offset is the ratio of the delta centroid (the distance between the geometrical centers of the cell and the nucleus) to the cell diameter (Supplementary Fig. 6b). Nuclear offset increased continuously throughout ETD, but did so earlier in Bcl-x_L_-*Epor*^*−/−*^ erythroblasts, with the difference between Bcl-x_L_-*Epor*^*−/−*^ and EpoR-*Epor*^*−/−*^ erythroblasts peaking at 48 h (*p* = 0.02) (Supplementary Fig. 6c, d). Taken together, both Ter119 expression and nuclear offset suggest that EpoR signaling prolongs erythroblast maturation.

### The differences between EpoR-transduced and Bcl-x-transduced *Epor*^*−/−*^ erythroblasts are maintained across a wide range of EpoR expression levels

The *Epor*^*−/−*^ transduction model allows confident identification of essential non-survival functions of the EpoR. This model suffers, however, from two drawbacks. First, it is in vitro; we therefore tested whether our conclusions hold in vivo (Fig. 2f, Supplementary Fig. 3, and below). Second, retroviral expression of EpoR and Bcl-x_L_ may differ in timing or expression level from their physiological profiles. We have shown above that the slower cycles, lower CD71 expression and smaller cell size of Bcl-x_L_-*Epor*^*−/−*^ erythroblasts result from absence of EpoR signaling rather than Bcl-x_L_ overexpression (Supplementary Figs. 2, 4). To investigate the potential effect of EpoR overexpression, we transduced *Epor*^*−/−*^ erythroblasts with high-titer, undiluted retroviral supernatant, resulting in a ~3.5-fold higher expression of EpoR, compared with physiological expression in fresh or cultured erythroblasts (Supplementary Fig. 7a). We then transduced *Epor*^*−/−*^ fetal liver cells with either undiluted, high-titer retroviral supernatant or with five-fold and ten-fold dilutions of the same supernatant. EpoR expression decreased 8-fold by qRT-PCR in cells transduced with a 10-fold diluted supernatant. We found that the differences between EpoR-transduced and Bcl-x_L_-transduced *Epor*^*−/−*^ erythroblasts, in CD71 expression, cell number, maturation rate, and cell size, were all maintained regardless of retroviral titer (Supplementary Fig. 7b–f). Therefore, EpoR functions identified with the *Epor*^−/−^ transduction model are not narrowly dependent on EpoR or Bcl-x_L_ expression levels. Further, the viability of EpoR or Bcl-x_L_-transduced *Epor*^−/−^ erythroblasts is comparable to that of wild-type erythroblasts (Supplementary Fig. 8).

### Epo increases cell size and prolongs maturation of wild-type erythroblasts in vitro and in vivo

**Fig. 6:**
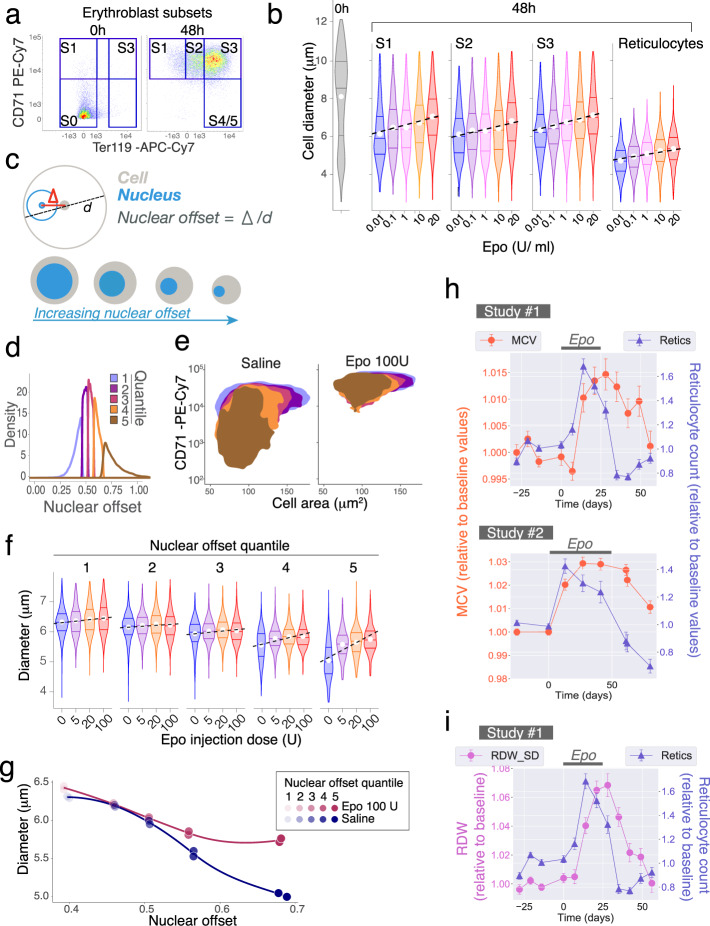
Red-cell size regulated by Epo concentration in mice and humans. **a**, **b** Epo regulates erythroblast diameter. CFU-e progenitors (‘S0’) enriched from wild-type fetal livers were differentiated in vitro in a range of Epo concentrations and analyzed at 48 h. Representative of two independent experiments. **a** S0 cells differentiate into the S1, S2, and S3 subsets by 48 h. **b** Cell diameter distributions for each erythroblast subset and Epo concentration. Violin lines are 25th, 50th, and 75th percentile. White circles mark the mean. **c** Nuclear offset measures nuclear eccentricity, independently of cell size. It is the ratio of the delta centroid (distance between the centers of the cell and the nucleus, Δ) and cell diameter. Nuclear offset increases during erythroid morphological maturation. **d**–**g** Mice were injected with saline (*n* = 2) or Epo (*n* = 2 mice with either 5 U, 20 U, or 100 U). Bone marrow analyzed at 48 h. **d** Nuclear offset quintiles for Ter119^+^ erythroblasts in saline-injected mice. **e** CD71/forward-scatter (FSC) histograms for the nuclear offset quintiles in (**d**) of Ter119^+^ erythroblasts. Quintile values that were determined for saline-injected mice were also applied to the Epo-injected mice. Sequential quintiles are seen to contain increasingly mature erythroblasts. **f** Cell diameter in each nuclear offset quintile in (**e**), for each Epo dose. Violin lines are 25th, 50th, and 75th percentile, white circle marks the mean. Data representative of *n* = 2 mice per Epo dose. **g** Median cell diameter and median nuclear offset values in each nuclear offset quintile, for mice injected with Epo (100 U) or Saline. Datapoints are individual mice. **h** Human intervention studies. Epo was administered during the period indicated. In study #1, *n* = 25 subjects were treated with Epo and *n* = 9 subjects with placebo. In study #2, *n* = 24 each for placebo and Epo. Data is fractional change relative to the baseline values of each participant. Additional hematological parameters and data for placebo groups are in Supplementary Figs. 14, 15 and Supplementary statistical analysis. MCV, mean corpuscular volume; Retics, reticulocyte count. **i** RDW_SD and reticulocyte counts for human intervention study #1 described in panel (**h**).

To test our conclusions outside the *Epor*^*−/−*^ transduction model, we asked whether Epo concentration affects cell size and maturation rate in wild-type erythroblasts in culture, and during Epo administration to mice in vivo. We differentiated wild-type fetal liver CFU-e (‘S0’ in Fig. 6a^40^) in vitro in the presence of a range of Epo concentrations. Cell size increased in an Epo-concentration-dependent manner, at every stage of differentiation, including reticulocytes (Fig. 6b, Supplementary Fig. 9a). The Epo-concentration range affecting cell size, from 0.01 to 10 Units/ml, corresponds to the entirety of the physiological and stress range in vivo^57,58^. Higher Epo also increased reticulocyte size heterogeneity (Supplementary Fig. 9b).

Epo also caused a dose-dependent delay in maturation. As expected, higher Epo resulted in higher cell number at all stages of differentiation (Supplementary Fig. 9c). However, the distribution of erythroblasts at higher Epo concentrations was increasingly skewed in favor of earlier differentiation subsets (Supplementary Fig. 9d). Similarly, the intensity of Ter119 expression decreased at higher Epo concentrations (Supplementary Fig. 9e). We further assessed cell maturation by measuring the nuclear offset, which decreased with increasing Epo concentration at all flow-cytometric stages (Supplementary Fig. 9f–g). Together these findings show that Epo prolongs ETD in a dose-dependent manner.

We also assessed the effect of Epo on erythroblast cell size in vivo. We injected mice with a range of Epo doses and used nuclear offset as a size-independent measure of maturational stage (Fig. 6c). We divided the nuclear offset distribution of all Ter119^+^ bone-marrow erythroblasts from saline-injected mice into quintiles (Fig. 6d). Increasing nuclear offset quintiles corresponded to increasingly mature erythroblast subsets as judged by the established criteria of decreasing CD71 and cell area, confirming the utility of this approach (Fig. 6e). We then used the nuclear offset quintiles values from these control mice to classify Ter119+ erythroblasts from Epo-injected mice into five maturational stages. We found that for a given nuclear offset-defined maturational stage, there was an Epo dose-dependent increase in cell diameter. This effect was particularly striking in erythroblasts that corresponded to the two most mature quintiles (Fig. 6f, g), confirming that Epo dose regulates erythroblast cell size.

Taken together, graded increases in Epo/EpoR signaling result in graded increases in cell size, shown by varying either the ligand concentration in wild-type erythroblasts (Fig. 6b, Supplementary Fig. 9a) or receptor expression in *Epor*^*−/−*^ erythroblasts (Supplementary Fig. 5a).

### EpoR signaling delays induction of p27^KIP1^, leading to increased number of cell cycles

To investigate the molecular mechanisms underlying EpoR-regulated functions, we compared gene expression in differentiating EpoR-*Epor*^*−/−*^ and Bcl-x_L_-*Epor*^*−/−*^ erythroblasts, using RT-qPCR (Supplementary Fig. 10). ETD markers *Slc4a1* (*Band3*) and *Hbb1* were induced similarly in both cell types. There were no significant differences in transcription factor expression, with the exception of Tal1, whose levels were 30% lower in Bcl-x_L_-*Epor*^*−/−*^ (*p* < 0.005). Tal1 was previously linked to cell-cycle regulation in hematopoietic cells^59^^,^^60^.

Among cell-cycle regulators, the CDK inhibitor p27^KIP1^ (*Cdkn1b*) was induced prematurely in Bcl-x_L_-*Epor*^*−/−*^, reminiscent of its premature expression in *Epor*^*−/−*^ primitive erythroblasts^20^. A second member of the CIP/KIP family, p57^KIP2^ (*Cdkn1c*), was also expressed at somewhat higher levels. The induction of p27^KIP1^ toward the end of ETD in wild-type erythroblasts contributes to mitotic exit^61–64^. To determine the effect of its premature induction, we transduced wild-type fetal liver S1 cells (CD71^high^Ter119^neg^) with either p27^KIP1^ or ‘empty vector’ (Supplementary Fig. 11a). p27^KIP1^-transduced cells showed reduced proliferation, without affecting cell viability, suggesting they underwent fewer cell cycles (Supplementary Fig. 11b, c). Unlike Bcl-x_L_-*Epor*^*−/−*^ erythroblasts, however, p27^KIP1^-transduced cells were larger, and slower to undergo maturation, as judged by lower nuclear offset (Supplementary Fig. 11d–f). Therefore, while the EpoR-mediated negative regulation of p27^KIP1^ increases cell-cycle number, its regulation of cell size and maturation rate are mediated by other pathways. Addition of the phosphatidylinositol 3-kinase (PI3K) inhibitor, LY294002^65^, to wild-type erythroblasts resulted in premature induction of p27^KIP1^ mRNA (Supplementary Fig. 12), suggesting that negative regulation of p27^KIP1^ by EpoR is mediated via PI3K.

### Several EpoR signaling pathways are implicated in the regulation of cell size

EpoR activates three principal signaling pathways: ras/MAP kinase, Stat5^39,66^, and PI3K^67,68^. Neonatal mice hypomorphic for Stat5 have microcytic anemia^69^. Here we found that, similarly, circulating red cells from E13.5 *Stat5*-deficient embryos are smaller than those of wild-type littermates (Supplementary Fig. 13a, b). Using U0126, a MEK1- and MEK2-specific inhibitor^70^, and the PI3K inhibitor LY294002, we examined the role of these pathways in the regulation of cell size. PI3K inhibition significantly decreased the size of early (‘S2’) and late (‘S3') erythroblasts and reticulocytes, but MEK1/2 inhibition had no consistently significant effect (Supplementary Fig. 13c, d). Therefore, it is likely that cell size regulation by EpoR is the integrated result of multiple signaling pathways.

### Epo administration increases red-cell size (MCV) and size variation (RDW) in human volunteers

We examined the effect of Epo on red-cell size in healthy volunteers in three intervention studies. Participants were either given Epo (studies #1 and #2, Fig. 6h, Supplementary Figs. 14 and 15), or subjected to phlebotomy (study #3, Supplementary Fig. 16). In studies #1 and #2, the effect of Epo on athletic performance was examined, and will be either reported elsewhere (study #1) or was previously reported (study #2^71^). Here we present the detailed blood parameters associated with these studies.

In study #1 (Fig. 6h, Supplementary Fig. 14), baseline parameters were established during four weekly blood samplings, followed by injection with Epo (20 IU/kg every other day, 25 subjects) or placebo (9 subjects) for 3 weeks. On average, hemoglobin increased by 5% over baseline values in the Epo group during the treatment period. Blood sampling continued for an additional 5 weeks following cessation of treatment. In study #2 (Fig. 6h, Supplementary Fig. 15), baseline measurements were followed by weekly dosing with Epo (24 subjects) or placebo (24 subjects) for 7 weeks, with Epo dosing adjusted to achieve an increase of 10–15% in hemoglobin. Follow-up continued for a month after cessation of treatment. In study #3 (Supplementary Fig. 16), 21 subjects participated in a randomized double-blind placebo-controlled crossover study in which 900 ml of whole blood was withdrawn from the treatment group by venipuncture. Subjects were then followed for 25 days.

In all three studies, there was a significant increase in MCV in the treatment groups compared with baseline values and with the placebo group, which persisted well beyond the treatment period (Supplementary Figs. 14–16, Supplementary statistical analysis). There was no correlation between MCV and the reticulocyte count, whose time courses were clearly divergent (*r* < 0.1 between MCV and reticulocyte count in all three studies, Pearson’s product-moment correlation, Supplementary statistical analysis). In studies #1 and #2, the reticulocyte count increased during Epo treatment, but declined sharply below baseline values as soon as Epo treatment ceased. By contrast, MCV values remained high (Fig. 6h). Similarly, in study #3, MCV values continued to climb at a time when the reticulocyte count was declining (Supplementary Fig. 16). Thus, the increase in MCV is not the result of an increase in the number of reticulocytes. Together with the increase in MCV, there was an increase in red-cell distribution width (RDW-SD, Fig. 6i, Supplementary Figs. 14 and 15; no RDW is available for study #3). There was a significant, positive correlation between MCV and RDW-SD (*r* = 0.51, *p* = 2 × 10^−28^ for study #1; *r* = 0.52, 2 × 10^−24^ for study #2).

Red-cell volume declines continuously as red cells age^72–76^. Therefore, we considered the possibility that the persistently elevated MCV following Epo administration might be the result of the expected increase in the relative number of younger red cells, rather than an increase in their size. To address this, we simulated the expected increase in MCV that would arise only from an increase in the proportion of younger red cells, assuming no effect of EpoR signaling on red-cell size (Supplementary analysis: ‘Simulation of MCV’). This simulation indicated that an increased proportion of younger red cells cannot fully account for the extent or duration of the observed increase in MCV following Epo administration, consistent with a direct role for EpoR signaling in the regulation of cell size.

## Discussion

**Fig. 7:**
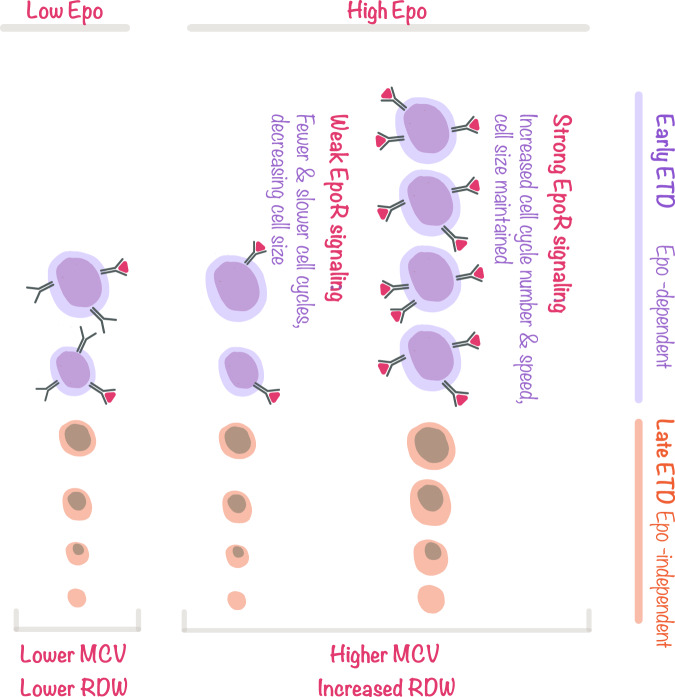
EpoR signaling promotes rapid cycling while maintaining cell size in early erythroblasts. Proposed model explaining EpoR-dependent functions during ETD. EpoR expression is limited to early erythroblasts, which are sensitive to EpoR signaling. When EpoR signaling is weak or absent, as in late erythroblasts, or in early erythroblasts in the presence of low Epo, cell divisions lead to cell size reductions. In contrast, strong EpoR signaling, as seen in Epo-sensitive early erythroblasts, can override this default state, simultaneously increasing rapid cycling while maintaining cell size. As consequence, high-Epo levels increase the duration of the early ETD phase, increase the relative frequency of early erythroblasts, and also increase erythroblast cell size at every maturation stage, giving rise to larger red cells. In high Epo, red-cell size is also more heterogeneous, a result of the varying sensitivities of early erythroblasts to Epo. Erythroblasts with low sensitivity to Epo, here represented as cells expressing low levels of EpoR, receive only weak EpoR signals even in the presence of high Epo, giving rise to smaller red cells.

Using a genetic model in which we provide *Epor*^*−/−*^ erythroblasts with exogenous survival signaling, we identified novel non-redundant functions for EpoR during ETD. EpoR signaling determines the number and speed of cell divisions and duration of terminal differentiation. While it has little effect on the broad ETD transcriptional program, it drives the formation of qualitatively different, larger red cells. In wild-type erythroblasts, EpoR signaling increases cell size in an Epo dose-dependent manner at every stage of erythroid terminal differentiation (ETD), leading to the production of larger reticulocytes and RCs. Human intervention studies are consistent with a similar effect of EpoR signaling on red-cell size in human erythropoiesis. In the discussion below we integrate the apparently disparate EpoR functions into a coherent model (Fig. 7). We also discuss previously unexplained instances of macrocytic and heterogeneously-sized red cells, now interpretable as the result of increased EpoR signaling during hypoxic stress.

The ETD is a time of rapid change in many aspects of the cell. Our results support a model in which ETD has two-phases: an early, Epo-dependent phase, and an Epo-independent late phase^39^ (Fig. 7). EpoR expression peaks in early erythroblasts, which are highly dependent on EpoR signaling for survival^6,53,77^, and exquisitely sensitive to Epo, as judged by Stat5 phosphorylation^66^. By contrast, late erythroblasts downregulate EpoR^12^ and are relatively resistant to apoptosis^53,77^. The functions we identified here for EpoR signaling in ETD are similarly focused on early erythroblasts. In addition to EpoR signaling, ETD is also supported by the erythroblastic island niche, an area that was not addressed in our model.

We identified five key non-survival functions of EpoR signaling, in *Epor*^*−/−*^ and in wild-type erythroblasts, in vitro and in vivo: (1) EpoR prolongs ETD, as determined by delayed expression of Ter119 and delayed increase in nuclear offset; (2) it increases the number of cell cycles; (3) it skews the distribution of developing erythroblasts in favor of earlier erythroblasts; (4) it increases cell-cycle speed; and (5) it increases cell size throughout ETD, generating larger and more heterogeneous red cells. The prolongation of ETD is consistent with the increase in the number of cycles. Neither informs us directly regarding the stage(s) of ETD that are being prolonged. However, the skewed distribution in favor of early erythroblasts indicates, based on the ergodic principle^78^ (see the “Methods” section), that EpoR signaling prolongs early ETD relative to the late ETD phase. Together, these observations suggest that EpoR prolongs the early phase of ETD by increasing the number of early ETD cell cycles. This conclusion is consistent with our data, showing the largest differences in cell-cycle number in response to EpoR occur in the first 24 h of ETD; and with the known responsiveness of early ETD to EpoR signaling. In addition, it explains the observation that EpoR increases cell-cycle speed, since early ETD cell cycles are unusually fast^26,27^, and much faster than cycles in late ETD^26,27,79^; our observations show that EpoR signaling regulates the speed of these unique cycles.

Therefore, of the five EpoR functions, the first four are outcomes of an EpoR-driven increase in the number and speed of early ETD cell cycles (Fig. 7). One of the factors known to regulate the onset of late ETD is p27^KIP1^, whose induction promotes slower cycling and cell-cycle exit^61,80,81^. Here we found that EpoR signaling increases cell-cycle number by inhibiting p27^KIP1^ mRNA induction through the PI3K pathway, which was previously reported to also lead to p27^KIP1^ proteosomal degradation^63^. A similar role for EpoR, delaying p27^KIP1^ induction and morphological maturation, was noted in primitive yolk-sac erythroblasts^20^. The converse was found in *Klf1*^*−/−*^ erythroblasts, which fail to induce p27^KIP1^ and fail to undergo cell-cycle exit^64^. Here we found that exogenous premature expression of p27^KIP1^ in wild-type erythroblasts reduced their cycling, but did not accelerate maturation, and like other factors that reduce cycling, resulted in larger erythroblasts. Therefore, the effects of EpoR signaling on erythroblast maturation rate and cell size are unrelated to its suppression of p27^KIP1^.

The most surprising of our findings was the effect of EpoR signaling on cell size. We found that erythroblasts differentiating in the absence of EpoR gave rise to smaller red cells, in spite of undergoing fewer cell cycles. Further, in wild-type fetal liver erythroblasts, cell size was sensitive to Epo concentration within the physiological and stress range. These findings appear contrary to the well-established link between the loss in cell size and the number of cell divisions during ETD. Thus, deletions of E2F4^29^, cyclin D3^30^, CDK2, or CDK4^31^ each reduce the number of cell divisions during ETD and result in macrocytic red cells. Similarly, macrocytic red cells are seen when nucleotide pools limit DNA synthesis rate, as in patients treated with hydroxyurea^32^, or in B12 or folate deficiencies. The EpoR effect on red-cell size was also independent of a second established pathway, in which red-cell size is regulated by iron status via HRI^34,52^. Neither iron supplementation nor deletion of HRI corrected the cell size deficit of *Epor*^*−/−*^ erythroblasts. While these experiments do not exclude an interaction between HRI and EpoR signaling^35^, they show conclusively that EpoR stimulation of larger red-cell size is independent of HRI.

Our data therefore suggest that EpoR regulates red-cell size through a novel mechanism. The finding that the EpoR-driven increase in cell size begins in early erythroblasts suggests that it takes place in the very same cells in which EpoR signaling also induces additional rapid cycles. We propose that the well-established coupling of cell size loss with cell divisions is a default state, seen in cells where EpoR signaling is weak or absent. We further suggest that strong EpoR signaling, as may occur in early erythroblasts^66^, can override this default state and maintain cell size in spite of rapid cycling (Fig. 7). The maintenance of cell size in dividing cells is the norm in most tissues^82,83^ and so it is possible that EpoR signaling permits early erythroblasts to employ similar pathways of size control as those found outside ETD. The mechanisms that determine the characteristic size of a cell and that maintain it through cell divisions are not fully understood, but are thought to depend on strong growth factor signaling to promote the metabolic pathways required for building biomass^83^. To maintain their size, cells must attain a size threshold before committing to cell division; in avian erythroblasts and other cell types, a larger size correlates with a longer G1 phase^82,84^. The ability of EpoR signaling to increase cell size in early erythroblasts, which are some of the most rapidly dividing cells in vivo^26,27^, predicts that these cells have exceptionally efficient mechanisms for growth. Conversely, this also implies that impairments in growth pathways would have a specifically deleterious effect, potentially contributing to the selective damage of ribosomopathies in the erythroid lineage^85^.

Together with an increase in cell size, high Epo also increased cell size heterogeneity, in mouse and human. Unlike low Epo levels, which generate only weak signaling and therefore relatively uniform small cells, high-Epo levels might be expected to support the survival of erythroblasts with varying Epo sensitivities^53,86^, in which the strength of EpoR signaling may vary, giving rise to a range of red-cell sizes (Fig. 7).

The relationship between high MCV, high RDW, and high levels of Epo may have been overlooked previously by being attributed to an increase in reticulocytes. We have excluded this possibility, finding no correlation between reticulocyte numbers and MCV. We also found that the extent and duration of increase in MCV following Epo administration cannot be accounted for solely by the skewing in the age distribution of circulating red cells in favor of younger cells (see Supplementary Analysis: Simulation of MCV). Indeed, our mouse data show increased cell size throughout terminal differentiation, including larger than normal reticulocytes, and not simply more numerous reticulocytes.

Recent GWAS and other studies have linked multiple genomic loci to the regulation of MCV^87–90^. These include *Epo*, *Epor,* and *Lnk*, all expected to alter EpoR signaling strength^91^. An Epo-mediated increase in MCV in clinical settings might be tempered by iron status or by pathology affecting terminal differentiation. Nevertheless, our work predicts that in the absence of erythroid pathology or nutritional deficiencies, Epo levels might be a key determinant of MCV. Indeed, an increase in Epo might account for the unexplained macrocytosis in hypoxemic patients with chronic obstructive pulmonary disease^92,93^ and in iron-replete pregnancy^94,95^. An increase in RDW was recently proposed as a potential longer-term biomarker for brief hypoxemic episodes in conditions such as acute respiratory distress, sepsis, or congestive heart failure^96,97^. Indeed, clinically, the RDW may prove to be a more sensitive marker of EpoR signaling than the MCV. The regulation of MCV by Epo also clarifies unexplained changes in red-cell volume associated with Kit function. Kit regulates the proliferation of early erythroid progenitors but is downregulated with entry into ETD. Gain of function Kit mutations in mice lead to erythrocytosis as a result of excess progenitors entering ETD; the red cells are microcytic^98^, presumably in response to a compensatory decrease in Epo. Conversely, loss of function Kit mutations are associated with an increased MCV, which is in proportion to the severity of anemia^98,99^, and can be now be explained by a paucity of progenitors entering ETD and the expected compensatory increase in Epo^100^. Transgenic expression of Epo rescues the lethal c-Kit^W/W^ mutation, also resulting in macrocytic red cells^99^. Given the persistence of higher MCV and RDW beyond the period in which Epo is elevated, these markers may be useful additions to a panel of diagnostic markers for detecting hypoxic stress in the clinic as well as Epo doping by athletes.

The adaptive value, if any, of a higher MCV in erythropoietic stress is not yet clear. Surprisingly, the increase in MCV in our human intervention studies was not associated with increased corpuscular hemoglobin (MCH). On the contrary, we found a statistically significant decrease in mean corpuscular hemoglobin concentration (MCHC) in both Epo intervention studies, though not in the phlebotomy intervention. Interestingly, a lower MCHC may enhance the action of 2, 3, diphosphoglycerate (2,3-DPG), an allosteric regulator that binds hemoglobin and lowers its affinity for oxygen. Red-cell 2,3-DPG increases in response to anemia or hypoxia, improving oxygen unloading in tissues^101,102^. The affinity of 2,3-DPG to hemoglobin increases significantly at lower MCHC^103^. A lower MCHC may therefore improve the 2,3-DPG-dependent unloading of oxygen. Indeed, a lower MCHC is also an HRI-regulated outcome characteristic of microcytic iron-deficiency anemia, possibly for similar reasons. The EpoR-regulated increase in MCV might therefore provide a mechanism for lowering MCHC and improving oxygen unloading in tissues during hypoxic stress.

## Methods

### Explanation of the ergodic principle

The ergodic principle can be applied in biology to a multi-stage process in the steady state (e.g., steady-state differentiation in tissue, or the cell cycle^104^^,^^82^). It suggests that in a snapshot in time of cells undergoing the process, the number of cells at each stage is inversely proportional to the length of time that cells spend at that stage. Hence, finding that a differentiation stage contains many cells suggests that cells spend a longer period of time in that stage; conversely, if a differentiation stage is sparsely populated, this would suggest that transit through that stage is fast. Therefore, as applied here, finding that EpoR signaling skews the erythroblast population in favor of early erythroblasts suggests that cells are spending proportionally more time in the early erythroblast stage.

### Mice

*Stat5*^*−/−*^ mice were obtained from Dr. Lothar Hennighausen (National Institute of Diabetes and Digestive and Kidney Diseases, Bethesda, MD). *Epor*^*+/−*^ mice were obtained from the Lodish laboratory, Whitehead Institute for Biomedical Research, Cambridge, MA. Balb/C mice were obtained from the Charles River Laboratories, Wilmington, MA. The Epo/Saline injection experiment on adult mice was conducted on male C57BL6 fluorescence timer (FT) transgenic mice. Mice were housed at a dedicated facility, with regulated temperature (range 20–26 °C), a 12 h/12 h dark/light cycle, and 30–70% humidity. Mice were fed on Iso Pro 3000 irradiated rodent diet #5P76. All experiments were conducted in accordance with animal protocol A-1586 approved by the University of Massachusetts Chan Medical School Institutional Animal Care and Use Committee.

### Culture medium and growth factors

Fetal liver cells were cultured in IMDM with added L-glutamine and 25 mM HEPES (Gibco), 20% fetal calf serum (Hyclone), 1% penicillin/streptomycin (ThermoFisher Scientific), 2 × 10^−4^ M β-Mercaptoethanol (Sigma), supplemented when indicated with 0.5 IU/ml Epo (Procrit, Amgen; 1 IU/ml = 1.2 ng/ml) and 100 ng/ml SCF (Peprotech), and 10 ng/ml IL3 (Peprotech).

### Isolation of mouse erythroid progenitors

To isolate wild-type S0 cells, fetal liver cells were depleted of lineage-positive cells by labeling with biotin-conjugated CD71, Ter119, Gr1, Mac1, and CD41 antibodies followed by magnetic separation using either EasySep beads a (StemCell Technologies) or MojoSort^TM^ Streptavidin Nanobeads (BioLegend) according to the manufacturers’ instructions.

### Flow cytometry

Fetal liver cells were analyzed on LSRII (BD Biosciences) cytometers using DIVA software (BD Biosciences). Dead cells were excluded using DAPI (Roche). FACS data were analyzed using FlowJo software (Tree Star Inc., CA).

Antibodies used:

PE Mouse Anti-Human CD4 (RPA-T4) (BD Biosciences) dilution 1:50

PE/Cy7 Rat Anti-Mouse CD71 (RI7217) (BioLegend) dilution 1:100

APC/Cyanine7 Rat Anti-Mouse Ter119 (Ter119) (BioLegend) dilution 1:100

PE Rat Anti-Mouse Ter119 (Ter119) (BD Biosciences) dilution 1:100

APC Rat Anti-Mouse Ter119 (Ter119) (BD Biosciences) dilution 1:100

biotin Rat Anti-Mouse CD71 (C2) (BD Biosciences) dilution 1:100

biotin Rat Anti-Mouse Ter119 (BD Biosciences) dilution 1:100

biotin Rat Anti-Mouse Ly-6G and Ly-6C/Gr1 (RB6-8C5) (BD Biosciences) dilution 1:100

biotin Rat Anti-Mouse CD11b/Mac1 (M1/70) (BD Biosciences) dilution 1:100

biotin Rat Anti-Mouse CD41 (MWReg30) (Thermo Scientific) dilution 1:100

FITC Rat Anti-Mouse Ly-6G and Ly-6C/Gr1 (RB6-8C5) (BD Biosciences) dilution 1:100

FITC Rat Anti-Mouse CD11b/Mac1 (M1/70) (BD Biosciences) dilution 1:100

FITC Rat Anti-Mouse CD41 (MWReg30) (BD Biosciences) dilution 1:100

FITC Rat Anti-Mouse CD45R/B220 (RA3-6B2) (BD Biosciences) dilution 1:100

FITC Hamster Anti-Mouse CD3e (145-2C11) (BD Biosciences) dilution 1:100

PE Rat Anti-Mouse Ly-6G and Ly-6C/Gr1 (RB6-8C5) (BioLegend) dilution 1:100

PE Rat Anti-Mouse CD11b/Mac1 (M1/70) (BioLegend) dilution 1:100

PE Rat Anti-Mouse CD41 (MWReg30) (BD Biosciences) dilution 1:100

PE Rat Anti-Mouse CD45R/B220 (RA3-6B2) (BD Biosciences) dilution 1:100

PE Hamster Anti-Mouse CD3e (500A2) (BioLegend) dilution 1:100

### Imaging flow cytometry

Imaging flow cytometry was used to analyze cell fluorescence in conjunction with morphological parameters. It was performed on an Amnis Flowsight cytometer (Luminex Corporation, TX) using INSPIRE software v6.5 (Luminex Corporation, TX). Live nuclear diameter was measured using the cell-permeable far-red fluorescent DNA dye, DRAQ5® (Cell Signaling). Amnis data was analyzed using IDEAS software v6.0 (Luminex Corporation, TX). New mask functions were generated to analyze bright-field cell area (Definition: Object (M01, Ch01, Tight)) as well as Draq5 fluorescence nuclear area (Definition: Morphology (M11, Ch11)). Raw mean Draq5 pixel intensity feature was generated using Draq5 Morphology mask for nuclear area. Raw flow cytometric feature data were exported and analyzed in R programming language.

#### Calibration of nuclear and cell diameters measured by imaging flow cytometry

Imaging flow cytometry was performed on standardized bead sizes, 2.0μ, 3.4μ, 5.1μ, 7.4μ, 9.96μ, and 14.3μ (Spherotech Inc.). IDEAS bright-field cell area mask (Definition: Object(M01, Ch01, Tight)) was fitted to the bead image acquisition. The data were analyzed using R, and within each bead group, values that lie greater or less than 3 standard deviations from the mean were removed (0.9% of events were removed with this threshold). To correct biases in the cell area values calculated by the Amnis software, we fit a linear model (polynomial curve) using the manufacturer bead sizes as a predictor for the Amnis calculated cell area (Stats, base R, degree = 2), with an R^2^ value of 0.97. This model was then used to predict cell diameters from experimental cell areas.

#### Analysis of imaging flow-cytometry data

Further analysis of exported imaging flow-cytometry data was done using RStudio Version 1.2.1335, RStudio, Inc. Population distributions were log normalized. Population dataset were filtered by removing outliers that are 3 or more standard deviations from the mean.

#### Fluorescence quantile analysis (Supplementary Fig. 4)

Events whose cell areas were 3 standard deviations from the mean were removed. For CD4 and GFP intensities, quantiles were calculated across all samples using the quantile function (Stats, base R). To visualize the data, a density plot was drawn using ggplot2 (geom_density)^105^ and colors chosen from viridis^106^. Each event was then categorized by which bin it fell into (for CD4 and GFP respectively). After this, each event had 2 associated values, which quantile of GFP and which quantile of CD4 that it belonged to. For each of the 3 samples (Bcl-x_L_^GFP^ + V^hCD4^, V^GFP^ + EpoR^hCD4^, and Bcl-x_L_^GFP^ + EpoR^hCD4^), the mean cell diameter was then calculated within each of these composite bins (i.e., the mean cell diameter in Sample X, for GFP quantile Y and CD4 quantile Z). Next, the ratio of the mean cell diameters within each CD4/GFP bin were calculated between Bcl-x_L_^GFP^ + V^hCD4^ and Bcl-x_L_^GFP^ + EpoR^hCD4^ using V^GFP^ + EpoR^hCD4^ as a reference. These data were plotted as a heatmap using ggplot2 and the geom_tile function. To show the distributions of cell diameters for all samples within each composite bin, example density plots were drawn using ggplot2 (geom_density). Example insets were colored by sample, and separate panels were drawn for each composite quantile bin.

#### Nuclear offset

The intensity weighted delta centroid XY feature was used to measure the distance between the centroid features of two images: CD71 fluorescence for the cell image and DRAQ5 fluorescence for the nucleus. To calculate the cell diameter, first the correlation between CD71 area feature and the bright-field-based area feature was obtained by plotting both values for each event. This allowed us to assign a bright-field area value to each event based on the CD71 area, and then use this value, in combination with the bead calibration curve (see “Calibration of nuclear and cell diameters” above), to calculate cell diameter. Nuclear offset was then calculated by dividing the delta centroid by cell diameter.

#### Identification of enucleated reticulocytes

Cells were selected by gating on focused, single cell, live, lineage (Gr1, Mac1, CD41, B220, CD3e) negative, hCD4 and GFP positive, and Ter119 positive events. The raw mean pixel intensity of Draq5 (nuclei) was plotted against the total Draq5 intensity (nuclei), giving two clearly distinct populations. We visually confirmed lack of Draq5 signal in the enucleated reticulocyte population.

### Cytospins

Cells were spun onto coated Shandon^TM^ Cytoslides (Thermo Scientific) using a Shandon^TM^ Cytospin3 at 800 rpm for 5 minutes. The slides were dried, fixed and stained^40^. Cytospin preparations were examined using a Zeiss Axioskop 40 microscope using a SPOT Flex Camera (Diagnostic Instruments, Inc.) and imaged using SPOT v.5.6 software (SPOT Imaging).

### Cell-cycle analysis

Cell-cycle status and S phase speed were analyzed using BrdU incorporation^26^. Briefly, cells were pulsed at a final concentration of 33 μM BrdU for 30 min. Cells were immediately labeled with the LIVE/DEAD Kit (Invitrogen L23105), fixed, and permeabilized. Erythroid subsets were identified using anti-CD71 (BD Biosciences 113812) and anti-Ter119 (BD Biosciences 553673). BrdU incorporation was measured by biotin-conjugated anti-BrdU (MOBU-1, BioLegend) followed by a secondary stain with Brilliant Violet 421™ Streptavidin (BioLegend). DNA content was measured by 7AAD (BD Biosciences).

### Retroviral Transduction and in vitro differentiation of fetal liver cells

Epor, Bcl-x_L_, and Tfrc were subcloned into MSCV-IRES-hCD4 retroviral vector. Bcl-x_L_ and Tfrc were also subcloned into MSIG-IRES-GFP retroviral vector (MSIG 1.1 SK). High-titer viral supernatants were prepared by co-transfecting the pCL-Eco packaging vector and desired plasmid into Phoenix cells using Lipofectamine 2000 transfection reagent (ThermoFisher Scientific). High-titer virus was collected in ‘erythroid medium’: IMDM (L-glutamine, 25 mM HEPES) (Gibco), 20% fetal calf serum, 1% penicillin/streptomycin, 10^−4^ M β-Mercaptoethanol.

Retroviral transduction was done by spin infection of *Epor*^*−/−*^ or *Epor*^*−/−*^ *Hri*^*−/−*^ fetal liver cells at 2000 rpm, 30 °C for 1 h on 50 µg/ml fibronectin (GIBCO) coated dishes in 4 µg/ml polybrene (Sigma), supplemented in some experiments with 0.5 U/ml Epo (Amgen). Transduced cells were incubated for 15 h with 100 ng/ml SCF and 10 ng/ml IL3 (Peprotech). Cells were then transferred to differentiation medium: IMDM (L-glutamine, 25 mM HEPES) (Gibco), 20% fetal calf serum, 1% penicillin/streptomycin, 10^−4^ M β-Mercaptoethanol, and 0.5 U/ml Epo (Amgen) for the indicated times. In the case of experiments that include *Epor*^*−/−*^ *Hri*^*−/−*^, the media was also supplemented with 1 mg/ml iron-saturated human transferrin (Sigma). Where indicated, liquid cultures were also supplemented with Fe-loaded salicylaldehyde isonicotinoyl hydrazone (Fe-SIH, 10 µM, a lipophilic iron chelator, a gift from the late Dr. Prem Ponka (McGill University, Montréal, Québec, Canada), with 0.7 µM deoxyribonucleosides (3′-Deoxythymidine, 2′-Deoxyguanosine monohydrate, 2′-Deoxyadenosine monohydrate, 2′-Deoxycytidine, Sigma),

### In vitro differentiation of fetal liver cells with PI3K and MEK1/MEK2 inhibitors

Isolated wild-type S0 cells were cultured in differentiation media (Epo 0.5 U/ml) with 1 µM or 10 µM PI3K inhibitor, LY294002 (EMD Millipore) or MEK1/MEK2 inhibitor, U0126 (EMD Millipore). Inhibitors were replenished every 24 h.

### Colony-formation assays in methylcellulose

Retroviral transduction was done by spin infection of *Epor*^*−/−*^ fetal liver cells as described above. From each transduced sample (4 h post infection), 200,000 cells were mixed with 1 ml MethoCult (M3234, STEMCELL Technologies) supplemented with 2 U/ml Epo (Amgen). Erythroid (CFU-e) was scored from duplicate plates on day 3 of culture. Expression of hemoglobin in erythroid colonies was confirmed by staining with diaminobenzidine (Sigma) in situ before scoring. Colony area was measured using ImageJ version: 2.0.0-r-54/1.51 h.

### Quantitative RT-PCR assay

Total RNA was isolated from in vitro cultured fetal liver cells using the AllPrep DNA/RNA Micro Kit (Qiagen) and quantified by Quant-iT RiboGreen RNA reagent kit (Thermo Scientific) on the 3300 NanoDrop Fluorospectrometer. Reverse transcription was done using the SuperScript III first-strand synthesis system (Invitrogen) with random hexamer primers. Quantitative PCR was performed using the ABI 7300 sequence detection system with TaqMan reagents and TaqMan MGB probes (Applied Biosystems). Each reaction was carried out on a dilution series of the template cDNA to ensure linearity of signal.

TaqMan MGB probes used: β-actin (Mm02619580_g1), PU.1 (Mm00488140_m1), GATA1 (Mm01352636_m1), GATA-2 (Mm00492300_m1), Alas2 (Mm01260713_m1), Band3 (Mm01245920_g1), β-globin (Mm01611268_g1), p21 (Mm00432448_m1), p27 (Mm00438168_m1), p57 (Mm01272135_g1), p16ink4a (Mm01257348_m1), p15ink4b (Mm00483241_m1), p18ink4c (Mm00483243_m1), p19ink4d (Mm00486943_m1), CCND1 (Mm00432359_m1), CCND2 (Mm00438071_m1), CCND3 (Mm01612362_m1), CCNE1 (Mm00432367_m1), CCNE2 (Mm00438077_m1), CCNA2 (Mm00438063_m1), CCNA1 (Mm00432337_m1), E2F2 (Mm00624964_m1), E2F4 (Mm00514160_m1), Tfrc (Mm00441950_m1), Bcl-xL (Mm00437783_m1), DNMT1 (Dnmt100599784), Ifitm1 (Mm01279023_m1), Ifitm3 (Mm00847057_s1), Tal1 (Mm00441665_m1), NFE2 (Mm00801891_m1), LMO2 (Mm00493153_m1), cdk6 (Mm00438163_m1), cdk6 (Mm01311342_m1), cdk4 (Mm00726334_s1), cdk2 (Mm00443947_m1), cdc25a (Mm00483162_m1), cdc25b (Mm00499136_m1), cdc25c (Mm00486880_m1), Klf1 (Mm00516096_m1).

### Epo stimulation in vivo

Epo (Epoetin alfa; Amgen) was injected subcutaneously in a total volume of 300 μL in sterile isotonic saline, at the indicated doses and frequencies.

### Human intervention studies

Human intervention studies 1 and 3 were performed at the University of Copenhagen. In intervention study 1, subjects received recombinant human erythropoietin (rhEPO). In intervention study 3, participants were subjected to phlebotomy. Thirty-four healthy non-smoking males (*n* = 19) and females (*n* = 15) of European descent (age 25 ± 3 years, height 179 ± 10 cm and weight 70 ± 10 kg) participated in the erythropoietin treatment intervention: *n* = 25 received Epo, *n* = 9 received Placebo. Another 21 healthy non-smoking male subjects of European descent (age 29 ± 6 years, 184 ± 7 cm, and 77 ± 8 kg) participated in the phlebotomy intervention. No participant had donated blood for at least three months prior to the start of the study or been exposed to high altitude (>1000 m) for at least two months.

The human studies were conducted in Copenhagen, Denmark according to all applicable national and international rules and regulations including the Helsinki II declaration. Ethics approval letters for the studies (protocol numbers H-2-2014-109 & H-17036662, enclosed with the Supplementary Information files; registration number, registration number NCT04227665 for Study #1) were granted by the Regional Branch (Copenhagen Region) of the Danish National Committee on Health Research Ethics (https://en.nvk.dk/). Both studies aim to identify novel biomarkers following either phlebotomy (Study #3) or Epo administration (Study #1). All participants were informed both orally and in writing of potential risks and discomforts associated with participation before written consent was obtained. Participants were compensated for their participation (Study #1: sports equipment equivalent to ~5500 Danish kroner; Study #2, 5000 Danish kroner). Participants were recruited via advertising on social media, dedicated web-pages, and flyers. There is a potential selection bias toward healthier than average participants since the studies examined the effect of Epo on athletic performance. This appears unlikely to influence the results.

#### Experimental design

*rhEPO treatment:* The study used a randomized single-blinded placebo-controlled design. After weekly baseline collection of venous blood for 4 weeks, the participants received eleven intravenous injections of 20 IU·kg bw^−1^ epoetin alpha (Eprex, Janssen, Birkerød, Denmark) (rhEPO group, 25 participants; 13 male and 12 female) or saline (placebo group, 9 participants; 6 male and 3 female) every second day. Venous blood samples were collected weekly during the treatment and for 5 weeks following treatment.

*Phlebotomy*: The intervention applied a randomized single-blinded placebo-controlled crossover design. The week before phlebotomy, two baseline venous blood samples were collected with 4 days apart. Next, the participants were phlebotomized of two whole-blood units, corresponding to 900 mL or sham-phlebotomized followed by venous blood collection 3, 14, and 25 days later. A recovery period of >4 months was applied before the participants crossed over and repeated the experiment.

#### Blood sample analysis

All venous blood samples were collected in 2 mL EDTA-anticoagulated vacutainers (Becton Dickinson, New Jersey, USA) after at least 10 min of rest in a seated position and with <30 s use of tourniquet. In the rhEPO trial, samples were immediately analyzed for a complete blood count using a Sysmex XN-450 (Sysmex, Kobe, Japan) including mean cell volume, hemoglobin concentration, reticulocyte count, reticulocyte percentage, and red-cell distribution width. In the phlebotomy trial, samples were stored at 4 °C and analyzed within 2 h of collection for mean cell volume, hemoglobin concentration, reticulocyte count, and reticulocyte percentage using a Sysmex XE-2100 (Sysmex, Kobe, Japan).

Human intervention study 2 was performed at the Centre for Human Drug Research, Leiden, Netherlands. This study was reported elsewhere^71^, but reporting did not include MCV and RDW information. Briefly, non-professional well trained male cyclists ages 28–50 were randomly assigned to placebo or recombinant human Epo (epoetin β) groups. Baseline measurements were followed by weekly dosing with Epo (24 subjects) or placebo (24 subjects) for 7 weeks. Epo dosing (5000–10,000 IU) was adjusted for each subject, to achieve an increase of 10–15% in hemoglobin over baseline. Follow-up continued for a month after cessation of treatment.

### Statistics

For the human studies, we computed baseline-corrected values at each post-baseline time point for each subject by subtracting the corresponding subject-level mean baseline measurement, which was used to fit linear mixed-effect models using the nlme package^107^. For intervention studies 1 and 2, the model includes subject as random effect, treatment, time, and the interaction of treatment by time as fixed effects. To test whether Epo treatment and placebo differ significantly at each post-baseline time point, a set of pre-defined contrasts were performed using the multcomp package^108^ followed by multiplicity adjustment using Benjamini–Hochberg procedure^109^. For intervention study 3, each post-baseline time point was analyzed separately with the model that includes subject as random effect, treatment, period, and sequence of treatments as fixed effects. See Supplementary Information.

For mouse and in vitro experiments, we used both parametric and non-parametric statistical significance tests for sample comparisons as indicated in each figure legend.

### Reporting summary

Further information on research design is available in the Nature Research Reporting Summary linked to this article.

## Supplementary information

Supplementary Information

Reporting Summary

## Data Availability

Complete blood-count source data for the human studies are provided in the ‘Supplementary statistical analysis of human intervention studies’ in the Supplementary Information file. Additional flow-cytometry data is available upon request. Source data are provided with this paper.

## Source data

Source Data

## Code availability

The Supplementary MCV simulation python script is deposited in https://github.com/socolovm/Simulation-of-MCV.
