# Supplementary material for: EpoR stimulates rapid cycling and larger red cells during mouse and human erythropoiesis
Source: Nat Commun. 2021 Dec 17;12:7334. doi: 10.1038/s41467-021-27562-4 (PMC8683474; doi:10.1038/s41467-021-27562-4)
Supplement: Supplementary file 1 — Supplementary Information [file 41467_2021_27562_MOESM1_ESM.pdf]

Inventory of Supplementary Information for:

EpoR Stimulates Rapid Cycling and Larger Red Cells During Mouse and Human Erythropoiesis

Daniel Hidalgo[1*], Jacob Bejder[2*], Ramona Pop[1†], Kyle Gellatly[3], Yung Hwang[1], S. Maxwell Scalf[4], Anna E. Eastman[4], Jane-Jane Chen[5], Lihua Julie Zhu[1,3,6], Jules A.A.C. Heuberger[7], Shangqin Guo[4], Mark J. Koury[8], Nikolai Baastrup Nordsborg[2$], Merav Socolovsky[1$]

[1] Department of Molecular, Cell and Cancer Biology, University of Massachusetts Chan Medical School, Worcester MA USA
[2] Department of Nutrition, Exercise and Sports, University of Copenhagen, Denmark
[3] Program in Bioinformatics and Computational Biology, University of Massachusetts Chan Medical School, Worcester MA USA
[4] Department of Cell Biology and Yale Stem Cell Center, Yale University, New Haven, CT, USA
[5] Institute for Medical Engineering & Science, MIT, Cambridge, USA
[6] Department of Molecular Medicine, University of Massachusetts Chan Medical School, Worcester MA USA
[7] Centre for Human Drug Research, Leiden, The Netherlands
[8] Department of Medicine, Division of Hematology and Oncology, Vanderbilt University Medical Center, Nashville, USA

[†] Present address: Harvard Department of Stem Cell and Regenerative Biology, Harvard University, Cambridge, MA USA
[*] Contributed equally to this work

[$] Corresponding authors:

Supplementary Information Files:

		Page:
**I**	Supplementary Figures 1 to 16	1 - 28
**II**	Supplementary Analysis: Simulations of MCV time course during and following Epo administration	29 - 39
**III**	Supplementary Statistical Analysis Of Human Intervention Studies	40 -101
**IV**	Supplementary Human Intervention Study Protocols	102 -128

Hidalgo et al., Supplementary Figures and Figure Legends

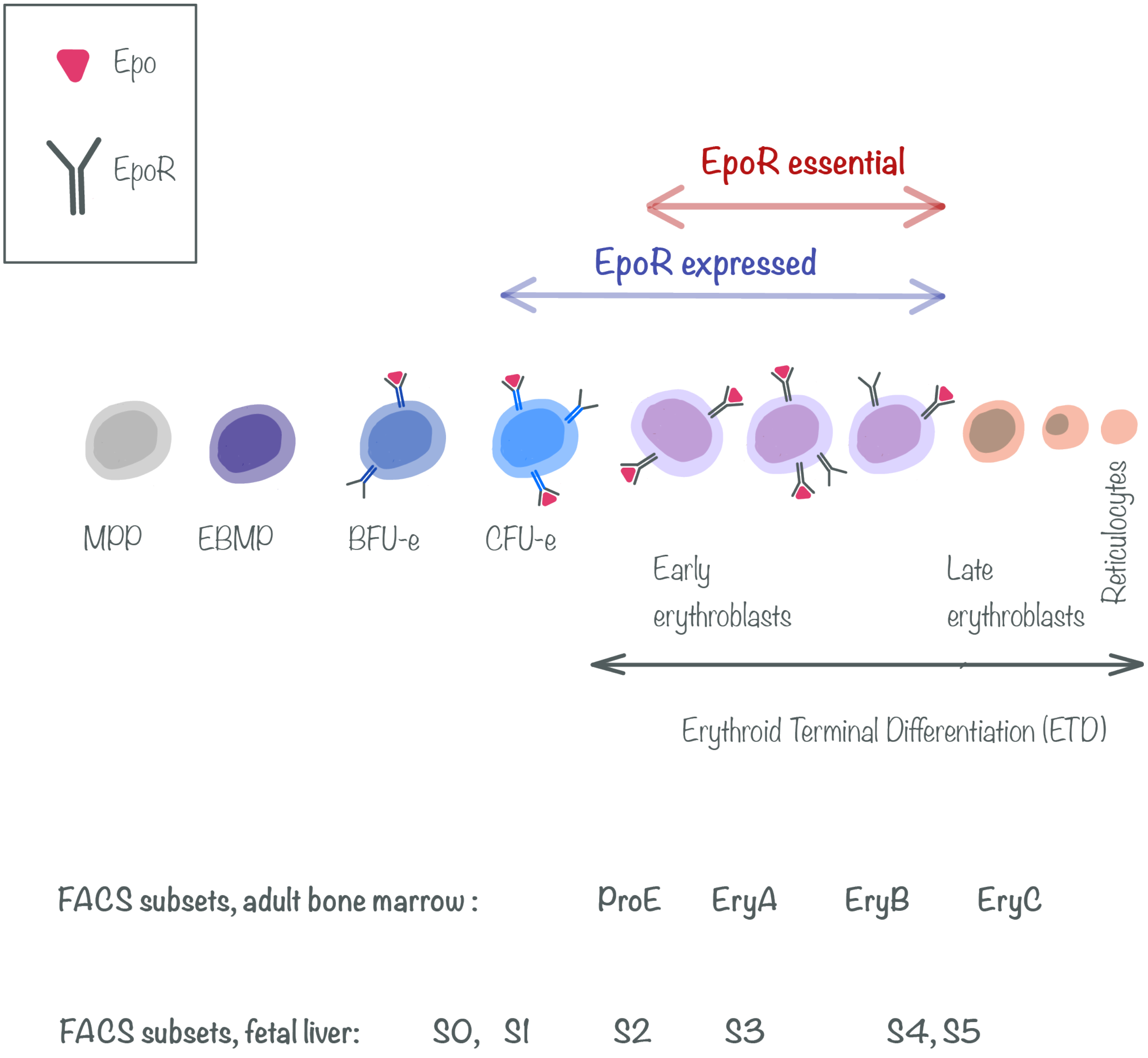

Supplementary Figure 1 Developmental stages of erythropoiesis when EpoR is expressed or essential.
The principal stages of the erythroid trajectory [1]. EpoR expression is induced at the BFU-e stage, and is downregulated in late erythroblasts. It is essential for the survival of early erythroblasts. FACS subsets used in the analysis of mouse fetal liver or adult bone marrow are also indicated; their horizontal position corresponds to their approximate developmental stage. MPP= Multipotential progenitors; EBMP = Erythroid, Basophil/mast cell, Megakaryocytic progenitors; BFU-e = Burst -forming unit erythroid; CFU-e = Colony-forming unit erythroid

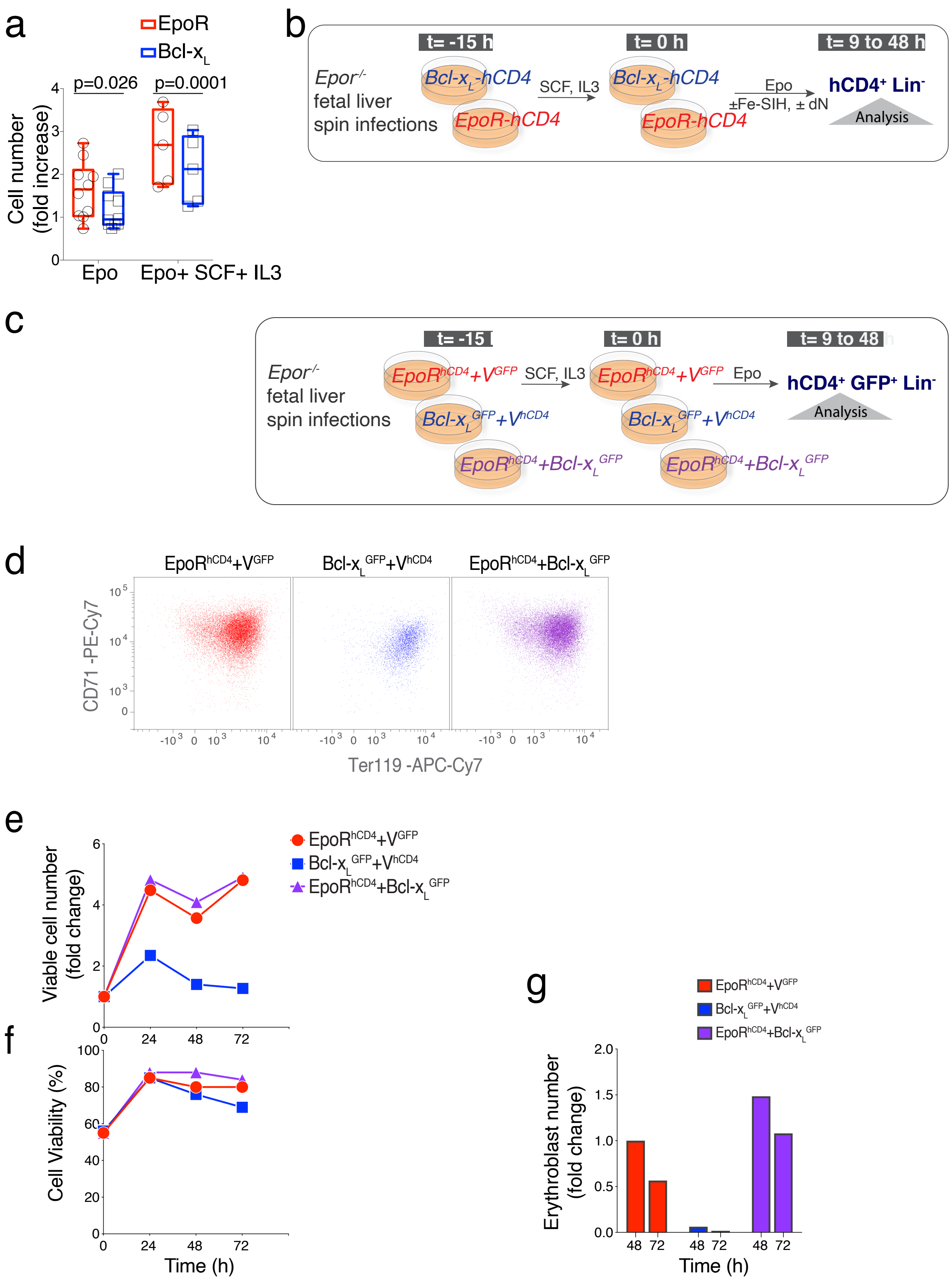

Supplementary Figure 2 Reduced growth of Bcl-x$_L$- *Epor$^{-/-}$* erythroblasts and their failure to upregulate CD71 persists in SCF and is not the result of bcl-x$_L$ over-expression

a Effect of IL-3 and SCF on viable cell number. *Epor*$^{-/-}$ fetal livers were transduced as in Fig 1a, and cultured for 19 hours, in the presence of Epo, and either in the presence or absence of IL-3 and SCF. Trypan-blue negative cells were scored. Data points are independent cultures pooled from three independent experiments. Box is 25th to 75th percentiles with the median marked; whiskers are min to max values. 2-tailed *t*-test, unequal variance.

b Modified experimental design, used in all subsequent experiments unless otherwise indicated. *Epor*$^{-/-}$ fetal livers were transduced at t= -15 h with bicstronic retroviral vectors encoding Bcl-x_L or EpoR and the hCD4 reporter and cultured in the presence of IL-3 and SCF for 15 hours. At t= 0 h, they were switched to an Epo containing medium, in the presence of additional factors as indicated in each experiment. Analysis was performed on transduced cells expressing hCD4 and negative for non-erythroid lineage markers (CD41, Mac1, Gr1, B220, CD3e).

c Experimental design: *Epor*$^{-/-}$ fetal livers were doubly transduced with bicistronic retroviral vectors, in the three shown combinations. Vectors encoded Bcl-x_L, EpoR or 'empty vector' (V); reporter constructs (GFP or hCD4) for each vector are shown as a superscript. Transduced cells were cultured in IL-3 and SCF for 15 hours, and switched to an Epo containing medium at t=0. Analysis was performed on cells that were doubly positive for both hCD4 and GFP, and negative for non-erythroid lineage markers (hCD4$^+$GFP$^+$Lin$^-$, where Lin= CD41, Mac1, Gr1, B220, CD3e).

d Flow cytometric analysis of *Epor*$^{-/-}$ hCD4$^+$GFP$^+$Lin$^-$ erythroblasts, showing failure to upregulate CD71 in cells lacking the EpoR, but not in cells doubly transduced with both EpoR and Bcl-x_L.

e Growth curves of doubly transduced *Epor*$^{-/-}$ fetal liver cells as in 'a'. Viable hCD4$^+$Lin$^-$ cells were counted at the indicated time points. Data are representative of two independent experiments, expressed relative to t=0.

f Cell viability, expressed as the fraction (%) of trypan blue negative cells, for the same set of samples shown in 'c'.

g Relative cell number of doubly transduced erythroblasts (Lin-hCD4+GFP+), for each of the indicated retroviral combinations at 48 and 72 hours of differentiation.

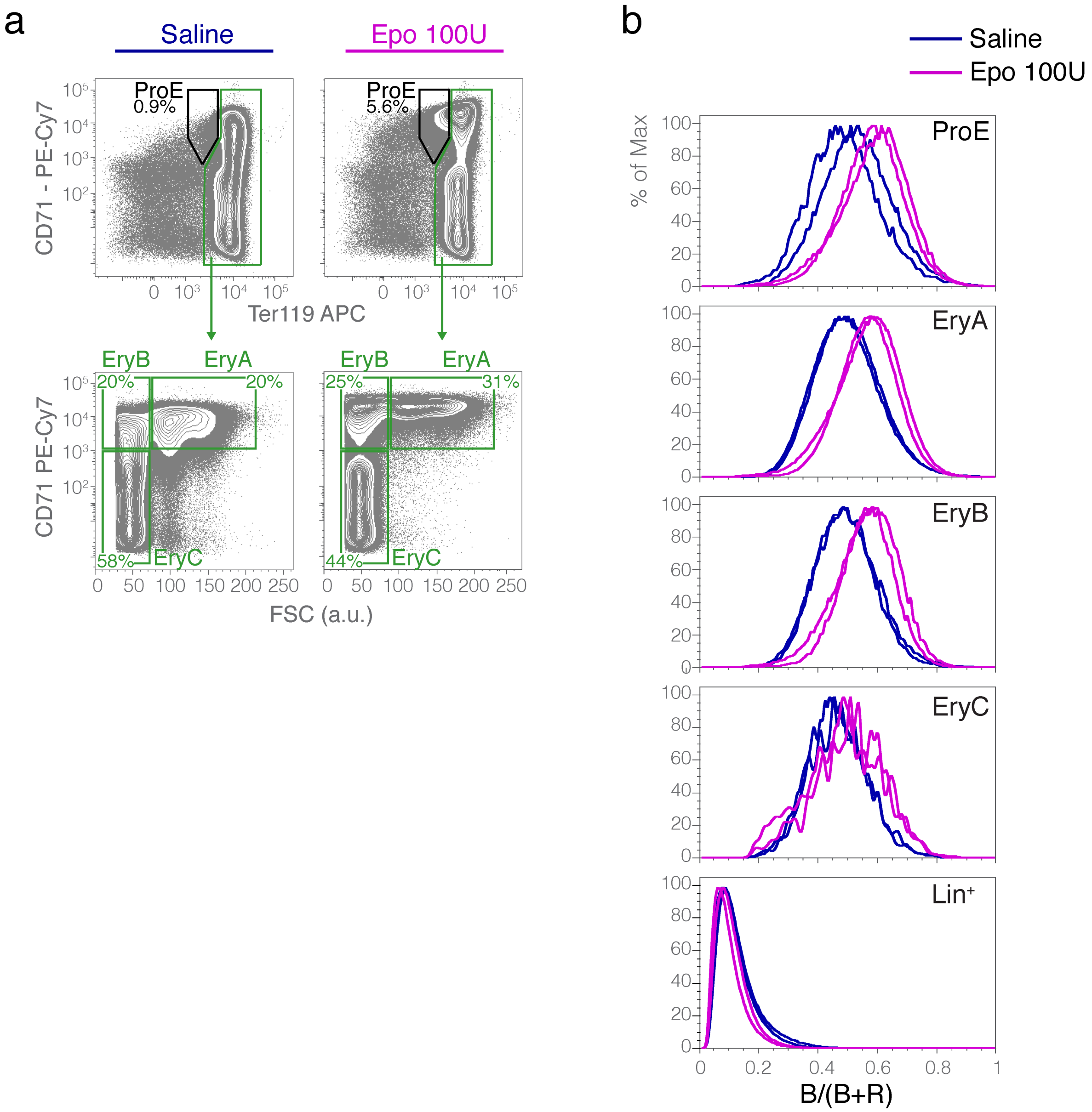

Supplementary Figure 3 EpoR stimulates cell cycle shortening *in vivo*

a, b Mice transgenic for the fluorescent timer protein fusion H2B-FT were injected with either saline or Epo (100 U) and analyzed at 36 h (see Figure 2f for additional detail).

a Representative flow cytometric profiles of bone marrow erythroblasts

b Histograms of *B/(B+R)* in live cells, for each of the erythroblast subsets gated in ‘a’, and for non-erythroid lineage-positive cells in the same bone-marrow sample (‘Lin+’). Histogram overlays are for 2 mice injected with saline and 2 mice injected with Epo.

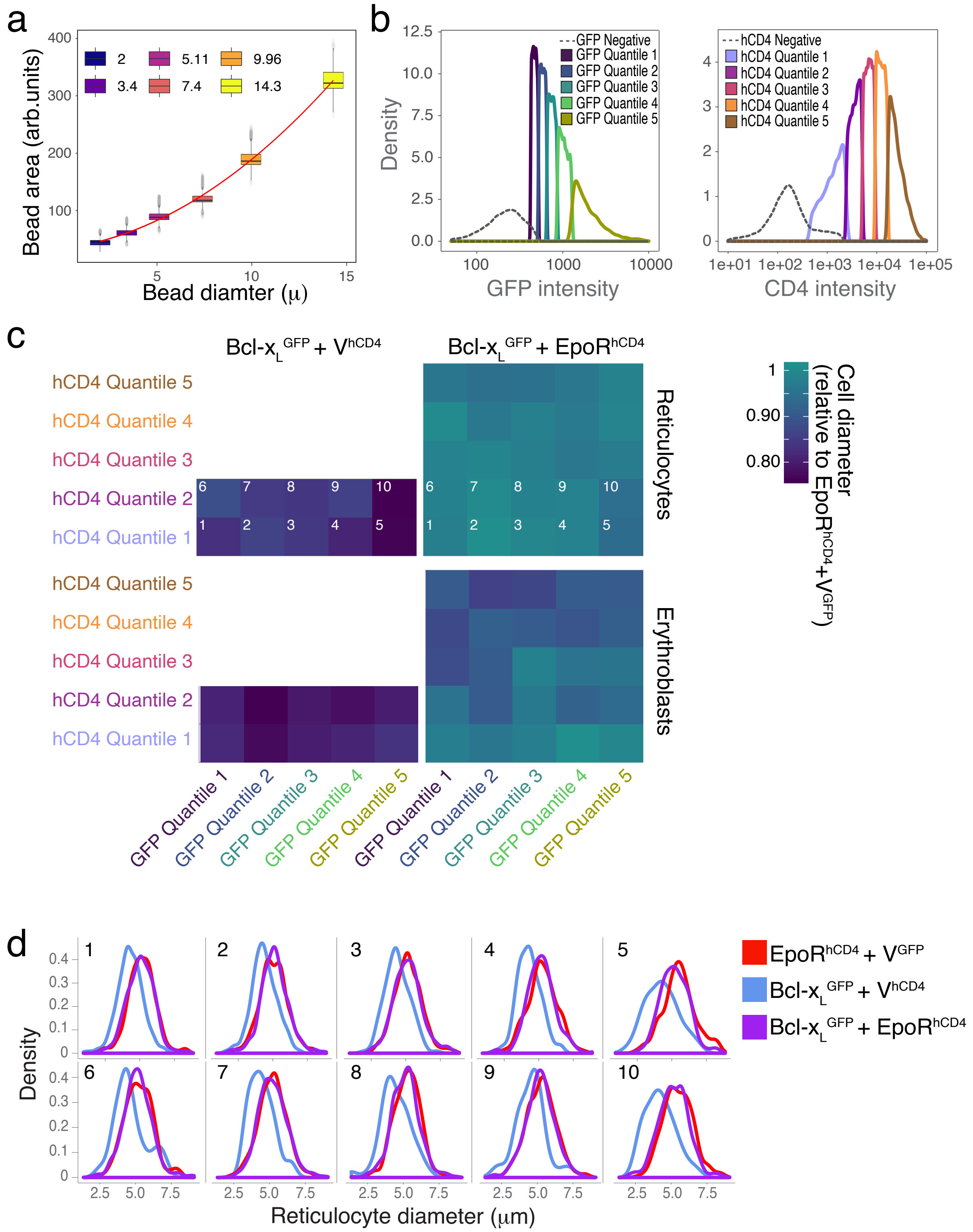

Supplementary Figure 4 A smaller cell size in the absence of EpoR is not the result of Bcl-x$_L$ over-expression

a Calibration of the cell diameter measurement, representative of n=8 independent experiments. Imaging flow cytometry was used to measure the area of polystyrene beads (Spherotech) of known diameter (indicated in legend, in micrometers). Data were fitted with a polynomial curve. The box and whiskers mark the 25th to 75th percentiles and min to max values, respectively, for all data-points with the median indicate. Calibration was performed for each imaging flow cytometry experiment.

b - c *Epor*$^{-/-}$ fetal livers were doubly transduced with bicistronic retroviral vectors, in three combinations, as described in **ED Figure 2**: EpoR-IRES-hCD4 + 'empty' GFP vector ($EpoR^{hCD4}$ + V^{GFP}); Bcl-x_L-IRES-GFP + 'empty' hCD4 vector (Bcl-x_L^{GFP} + V^{hCD4}); or with Bcl-x_L-IRES-GFP + EpoR-IRES-hCD4 (Bcl-x_L^{GFP} + $EpoR^{hCD4}$). Transduced cells were cultured in IL-3 and SCF for 15 hours, and switched to an Epo containing medium at t= 0 h. Analysis was performed on cells that were doubly positive for both hCD4 and GFP, and negative for non-erythroid lineage markers ($hCD4^+GFP^+Lin^-$, where Lin= CD41, Mac1, Gr1, B220, CD3e). The analysis below was performed at t= 48 h.

b In order to compare cells with similar expression of each of the retroviral constructs, we divided the erythroblast population ($hCD4^+GFP^+Lin^-$) into five fluorescence intensity quantiles for each of the GFP and hCD4 channels. The dashed line indicates the baseline fluorescence of cells that are not transduced with the corresponding vector (that is, cells transduced with only a GFP vector when examining the hCD4 channel, and vice versa).

c Erythroblast (upper panel) and reticulocyte (lower panel) cell diameters for cells expressing Bcl-x_L^{GFP} + V^{hCD4}, compared with cells expressing Bcl-x_L^{GFP} + $EpoR^{hCD4}$. The comparison is done separately for each quantile combination, and is depicted relative to the diameter of cells expressing $EpoR^{hCD4}$ + V^{GFP} in the same quantile combination. For example, the diameters of cells transduced with Bcl-x_L^{GFP} + V^{hCD4} in quantile combination 10 (containing cells in the 5th quantile of the GFP channel and 2nd quantile of the hCD4 channel), are compared with diameters in quantile combination 10 of cells transduced with Bcl-x_L^{GFP} + $EpoR^{hCD4}$, and expressed as a ratio to the diameters in quantile combination 10 of cells transduced with $EpoR^{hCD4}$ + V^{GFP}. Quantile combinations containing fewer than 10 cells were excluded.

d Reticulocyte diameters for each quantile combination, represented as histograms. Data as described in 'c'.

a

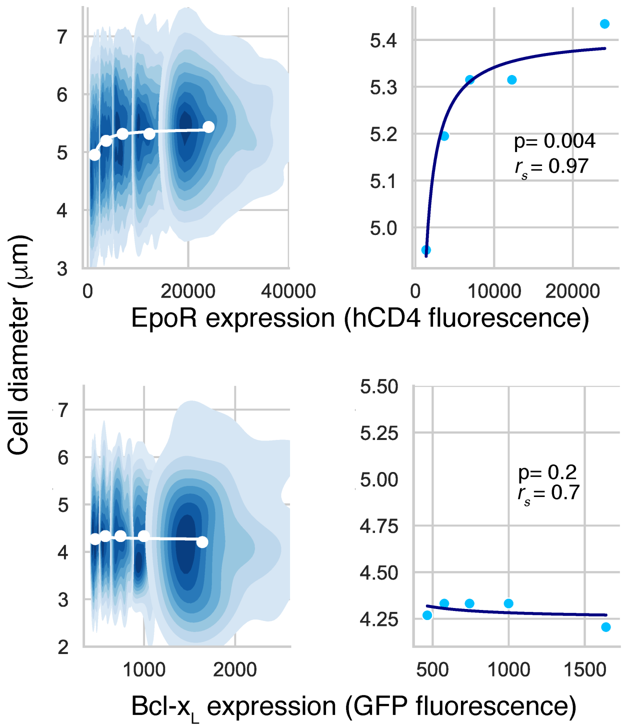

b

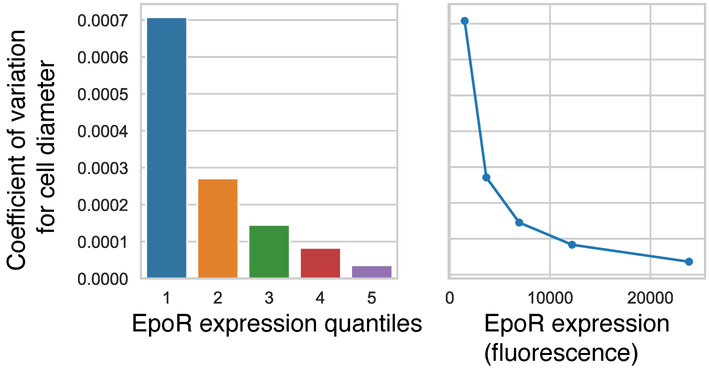

Supplementary Figure 5 Cell surface expression of EpoR regulates erythroblast cell size

Epor$^{-/-}$ fetal liver cells were doubly transduced with bicistronic retroviral vectors and cultured for 48 hours in Epo = 0.5 U/ml (experiment as in **ED Figure 5**).

a Cell diameter is plotted against EpoR expression for cells transduced with EpoR^{hCD4}; or against Bcl-x_L expression for cells transduced with Bcl-x_L^{GFP} . Transduced erythroblasts were divided into quintiles based on their expression of either GFP or hCD4. Median cell diameter is plotted against the median fluorescence for cells in each quintile. Left panels shows contour

plots for all cells in each quintile, with median value is shown in white. Right panels show median values for each quintile, fitted with a dose/response curve of the form $EC_{max}/(1+(EC_{50}/x))$. Spearman correlations and associated p-values are shown on each graph.

b Variability in the cell diameter measurement within each quintile, and as a function of EpoR expression levels in transduced cells, in the same dataset as in ‘a’.

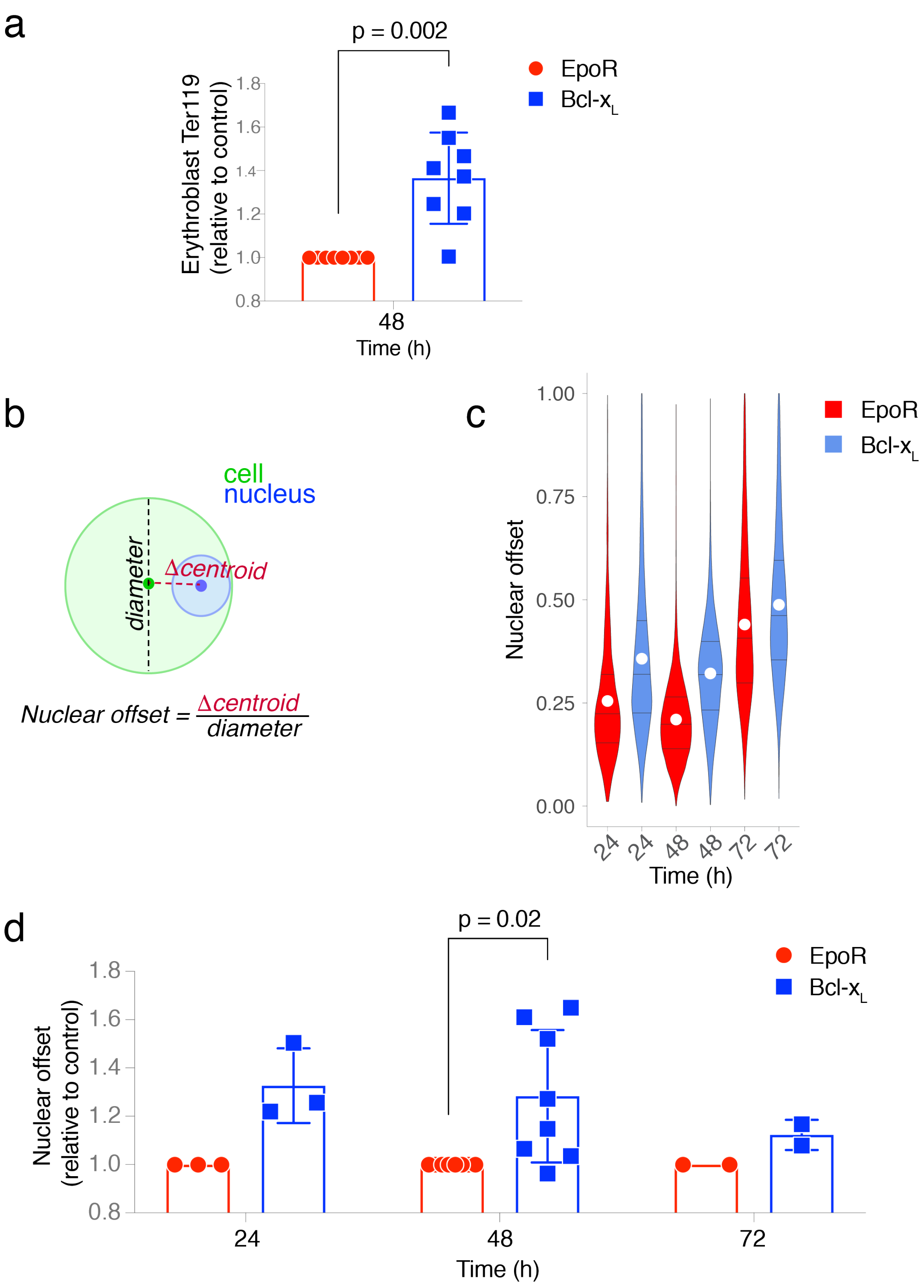

Supplementary Figure 6 **EpoR signaling prolongs the ETD stage**

a Ter119 intensity in *Epor*$^{-/-}$ erythroblasts (Lin^{-} hCD4^{+}) transduced with either Bcl-x_L or EpoR, at 48 h. Data are mean Ter119 intensities from 9 independent experiments. Box and error bars are mean ± SD. p values are for a 2-sided paired *t* test.

b Explanation of the nuclear offset measurement. The geometrical centers (=centroids) of the cell and the nucleus are indicated by green and blue solid circles. The distance between the centroid is the delta centroid (red dashed line, also known as the X,Y delta centroid). The nuclear offset is a dimensionless ratio of the delta centroid to the cell diameter.

c Representative nuclear offset measurements of *Epor*$^{-/-}$ erythroblasts (Lin^{-} hCD4^{+}) transduced with either Bcl-x_L or EpoR, at the indicated time points. Violin lines mark the 25th, 50th and 75th percentile with a white circle marking the mean.

d Nuclear offset measurements of *Epor*$^{-/-}$ erythroblasts (Lin^{-} hCD4^{+}) transduced with either Bcl-x_L or EpoR. Individual datapoints are population medians pooled from 9 independent experiments. Box and error bars are mean± SD. p values are for a 2-sided paired *t* test.

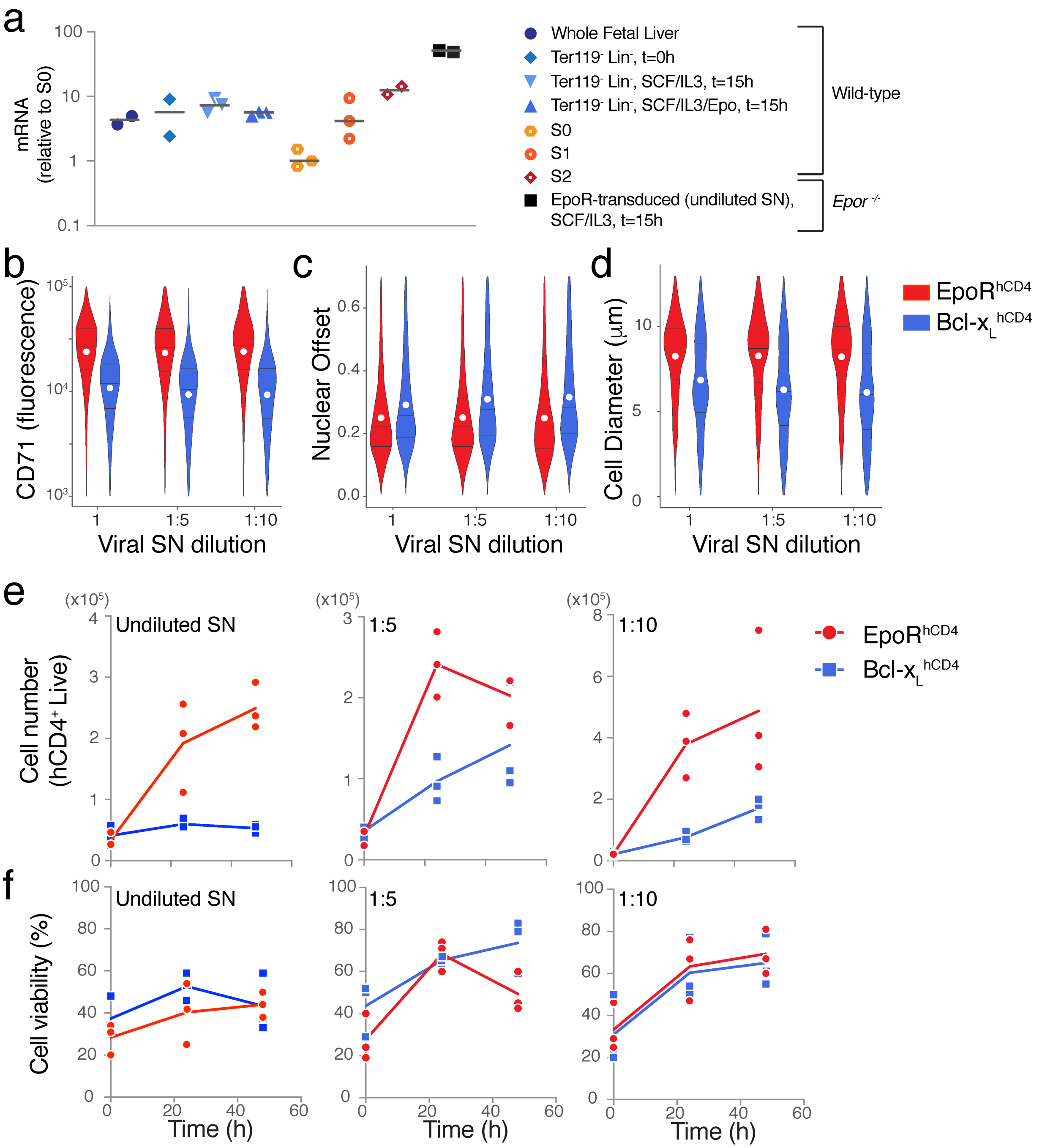

Supplementary Figure 7: Functional differences between EpoR -transduced and Bcl-x-transduced *Epor*$^{-/-}$ erythroblasts are maintained across a wide range of EpoR expression levels

a *Epor* gene expression in *Epor*-transduced *Epor-/-* fetal liver cells cultured for 15h, compared to *Epor* gene expression in similarly cultured wild-type fetal liver cells or in freshly isolated wild-type fetal liver subsets (Ter119^{-} Lin^{-}, S0, S1, S2). mRNA was measured by quantitative RT-PCR, normalized to the β-actin mRNA, and expressed relative to the S0 subset.

Data are 3 (for S0 and S1) or 2 (EpoR-transduced *Epor*$^{-/-}$ fetal liver cells) independent experiments and 2 (whole fetal liver cells and Ter119^{-}Lin^{-} t=0h) or 3 (Ter119^{-} Lin^{-} SCF/IL3, Ter119^{-} Lin^{-} SCF/IL3/Epo t=15h) technical replicates.

b Effect of retroviral titres on CD71 expression. *Epor*$^{-/-}$ fetal livers were transduced with retroviral supernatants ('SN') which were either undiluted ('1'), or diluted by 1:5 or 1:10 as indicated. Retroviral vectors encoded Bcl-x_L or EpoR and the hCD4 reporter. Expression of Epor mRNA was 8-fold lower in cells transduced with the ten-fold dilution of retroviral SN. Analysis was performed on hCD4^{+} Lin^{-} cells. Violin lines mark the 25th, 50th and 75th percentile with a white circle marking the mean.

c,d Nuclear offset and cell diameter measurements at t=48h following EpoR or Bcl-x_L retroviral transduction as in 'b'. Violin lines mark the 25th, 50th and 75th percentile with a white circle marking the mean.

e Viable cell number following EpoR or Bcl-x_L retroviral transduction as in 'b'.

f Cell viability, expressed as the fraction (%) of trypan blue negative cells, for the cultures shown in 'e'.

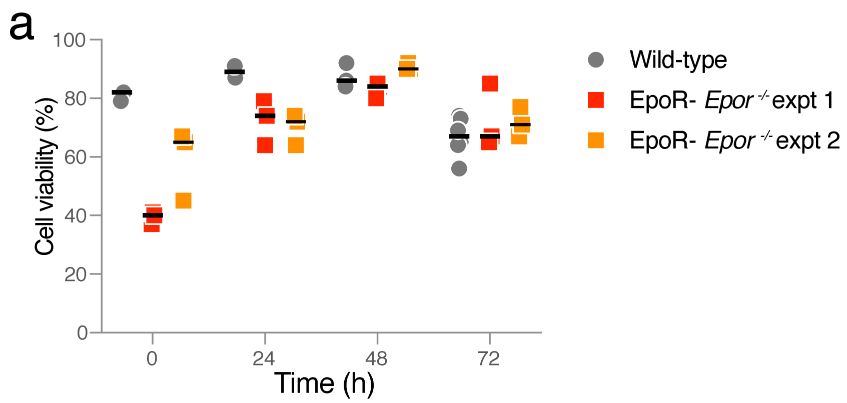

Supplementary Figure 8: EpoR-transduced *Epor-/-* erythroblasts have similar viability to that of wild-type fetal liver cells

a Viability of wild-type and EpoR-transduced fetal liver cells during in vitro culture, measured as the fraction of cells that are trypan- blue negative. *Epor*$^{-/-}$ fetal livers were transduced with bicistronic retroviral vector encoding EpoR and the hCD4 reporter. Wild-type fetal liver cells and EpoR- *Epor*$^{-/-}$ transduced cells were cultured in the presence of IL-3 and SCF for 15 hours. At t=0 h, they were switched to an Epo containing medium and differentiated *in vitro* for 72h. Data are 2 independent experiments.

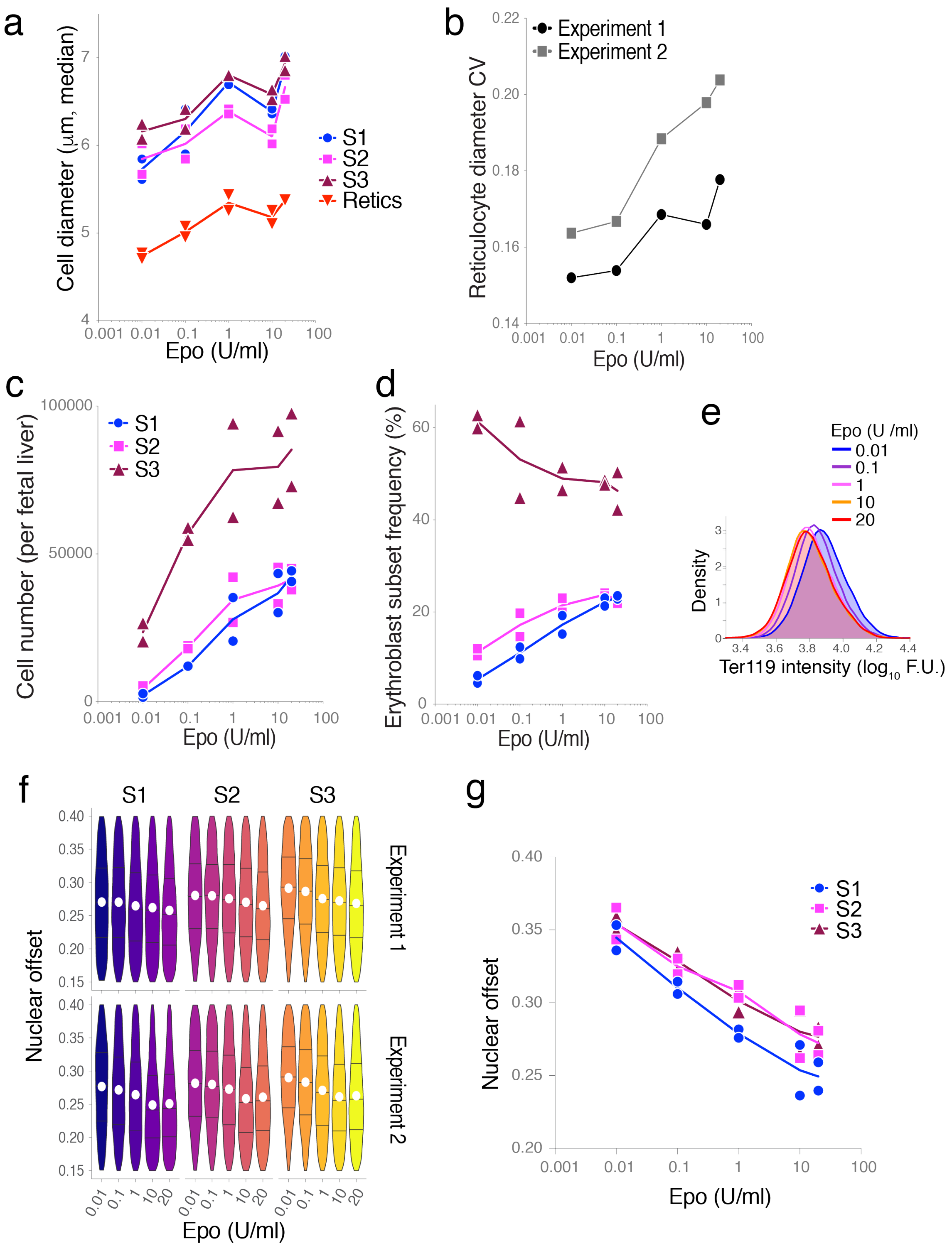

Supplementary Figure 9 Multiple ETD parameters are sensitive to Epo concentration
a - g Experiment as in **Figure 6a-b**. Wild-type fetal liver cells were enriched for CFU-e progenitors ('S0') and differentiated *in vitro*, in a range of Epo concentrations between 0.01 and 20 U/ ml. Cultures were analyzed at 48 h. Data are pooled from two independent experiments.

a Cell diameter (population medians) of erythroblast subsets S1 to S3 and of reticulocytes.
b Heterogeneity in reticulocyte diameter. CV, coefficient of variation.
c, d Viable cell number ('c') and cell frequency in the culture ('d') for each erythroblast subset.
e Ter119 intensity of all nucleated erythroblasts in the culture, for each of the indicated Epo concentrations at 48h.
f, g Nuclear offset at each Epo concentration and for each erythroblast subset. Violins ('e') are representative of two independent experiments. Data medians from both experiments are plotted in 'f'. Violin lines are the 25th, 50th and 75th percentile with a white circle marking the mean.

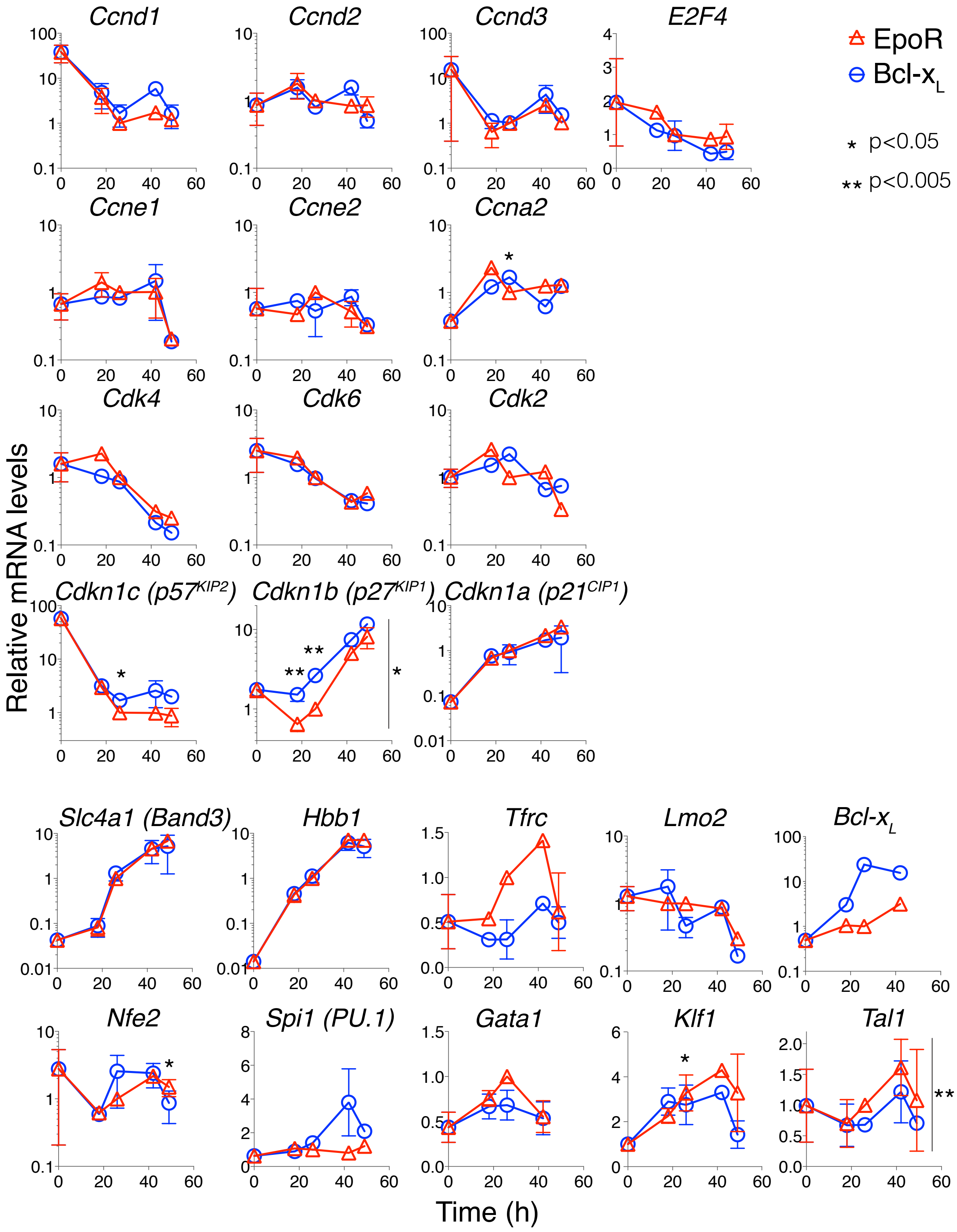

Supplementary Figure 10 Premature expression of $p27^{KIP1}$ in the absence of EpoR

Gene expression in Bcl-x_L or EpoR-transduced *Epor*$^{-/-}$ fetal liver cells during their differentiation in vitro. mRNAs were quantitated with RT-qPCR and are expressed relative to expression of β-actin in the same cells.

In each experiment, RT-qPCR was carried out using a dilution series of the cDNA. Data for each gene is mean± SD pooled from a number of independent as follows: Ccnd1, n=6; Ccnd2, n=7; Ccnd3, n=6; E2F4, n=5; Ccne1, n=5; Ccne2, n=6; Ccna1, n=5; Cdk4, n=3; Cdk6, n=3; Cdk2, n=3; Cdkn1c (p57^{KIP2}), n=7; Cdkn1b (p27^{KIP1}), n=7; Cdkn1a (p21^{CIP1}), n=2; Slc4a1(Band3), n=6; Hbb1, n=7; Tfrc, n=6; Lmo2, n=6; Bcl-x_L, a representative experiment of 3; Gata1, n=7; Spi1 (PU.1), n=7; Tal1, n=6; Nfe2, n=6. p values are for a 2-sided paired *t* test.

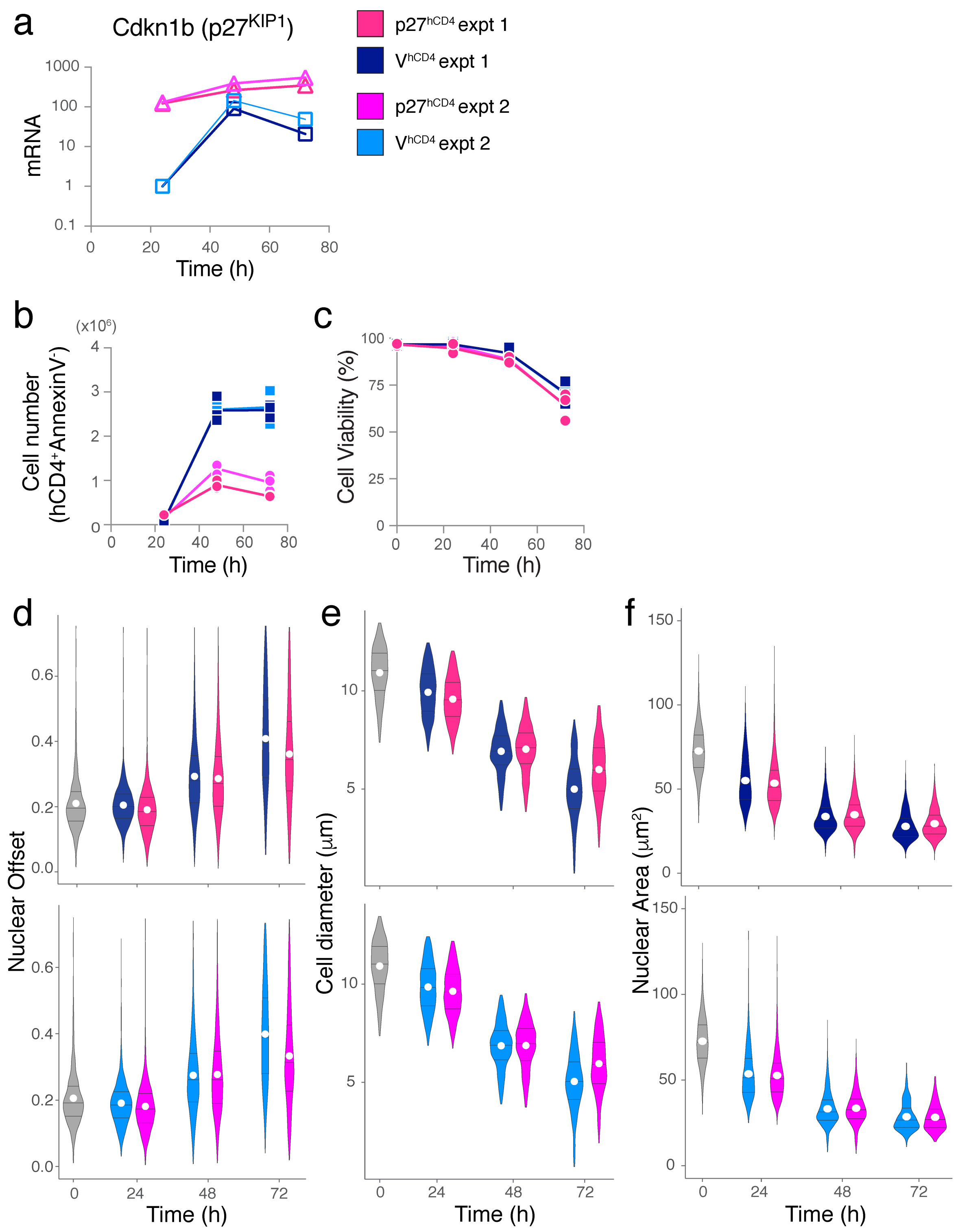

Supplementary Figure 11: Premature expression of p27 only partly reproduces the Bcl-x$_L$-*Epor*$^{-/-}$ phenotype

a $p27^{KIP1}$ gene expression in differentiating wild-type fetal liver cells transduced with either $p27^{KIP1}$ or with 'empty vector'; the bicistronic vectors also expressed the hCD4 reporter. S1 subset cells ($CD71^{high}Ter119^{neg}$) were FACS sorted from E13.5 fetal liver cells, transduced with retroviral vectors and cultured in IL-3, SCF, and Epo (0.5U/mL) -containing medium *in vitro* for 72 hours. mRNA was measured by qRT-PCR, normalized to the β-actin mRNA, and expressed relative to that in control (empty vector)-transduced cells at t=24h. Data are from two independent experiments.

b Viable cell number ($hCD4^+$ Annexin V^-) following $p27^{KIP1}$ or 'empty vector' retroviral transduction for the experiments in 'a'.

c Cell viability (% trypan blue negative cells), for the experiments in 'a'.

d-f Nuclear offset ('d'), cell diameter ('e') and nuclear diameter ('f') following $p27^{KIP1}$ or 'empty vector' retroviral transduction for the experiments in 'a'. Violin lines mark the 25th, 50th and 75th percentile with a white circle marking the mean. Colors as in 'a', grey marks t=0.

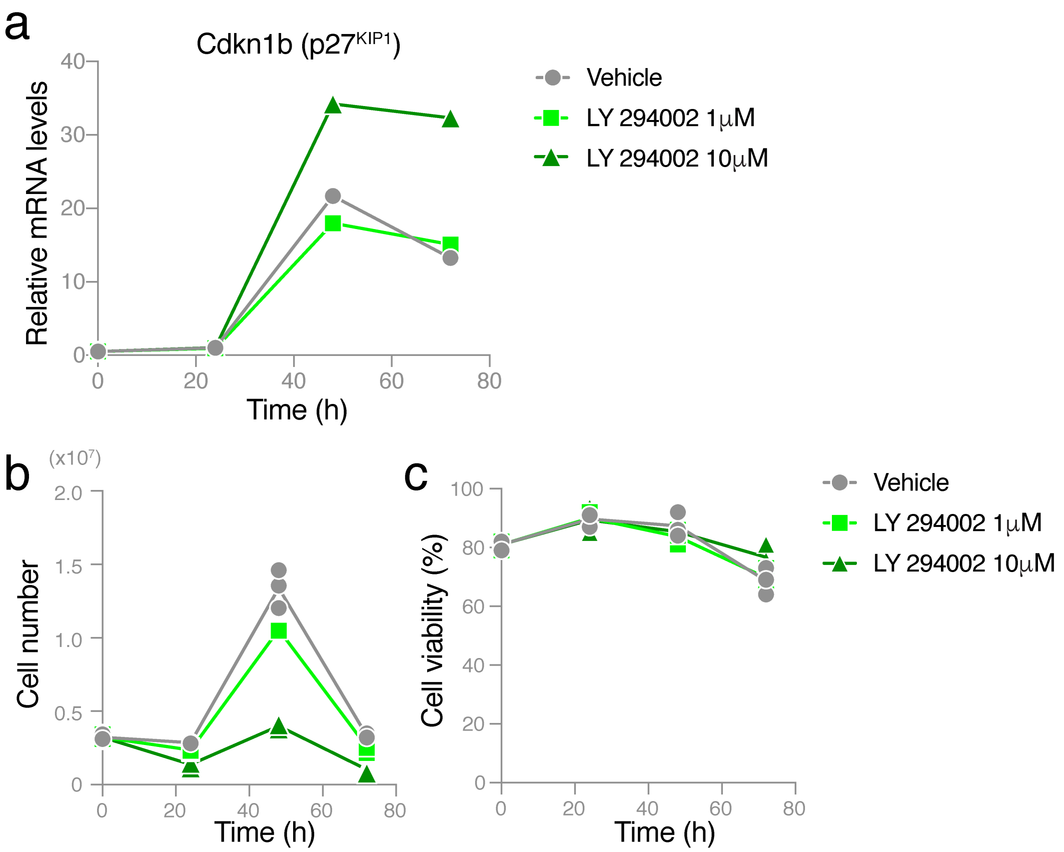

Supplementary Figure 12: PI3-kinase inhibition results in premature induction of p27^{KIP1} and in decreased number of differentiating erythroblasts

a p27^{KIP1} gene expression in wild type fetal liver cells treated with LY 294002. Ter119^{-}Lin^{-} cells were sorted from E13.5 wild-type fetal liver and cultured in the presence of either vehicle (DMSO) or the PI3 kinase inhibitor LY 294002 at the indicated concentrations. mRNA was measured by qRT-PCR, normalized to the β-actin mRNA, and expressed relative to vehicle. Data is median of 4 technical replicates.

b Viable cell number for the experiment in ‘a’. Data are 3 technical replicates.

c Cell viability (% of trypan blue negative cells) for the experiment in ‘a’.

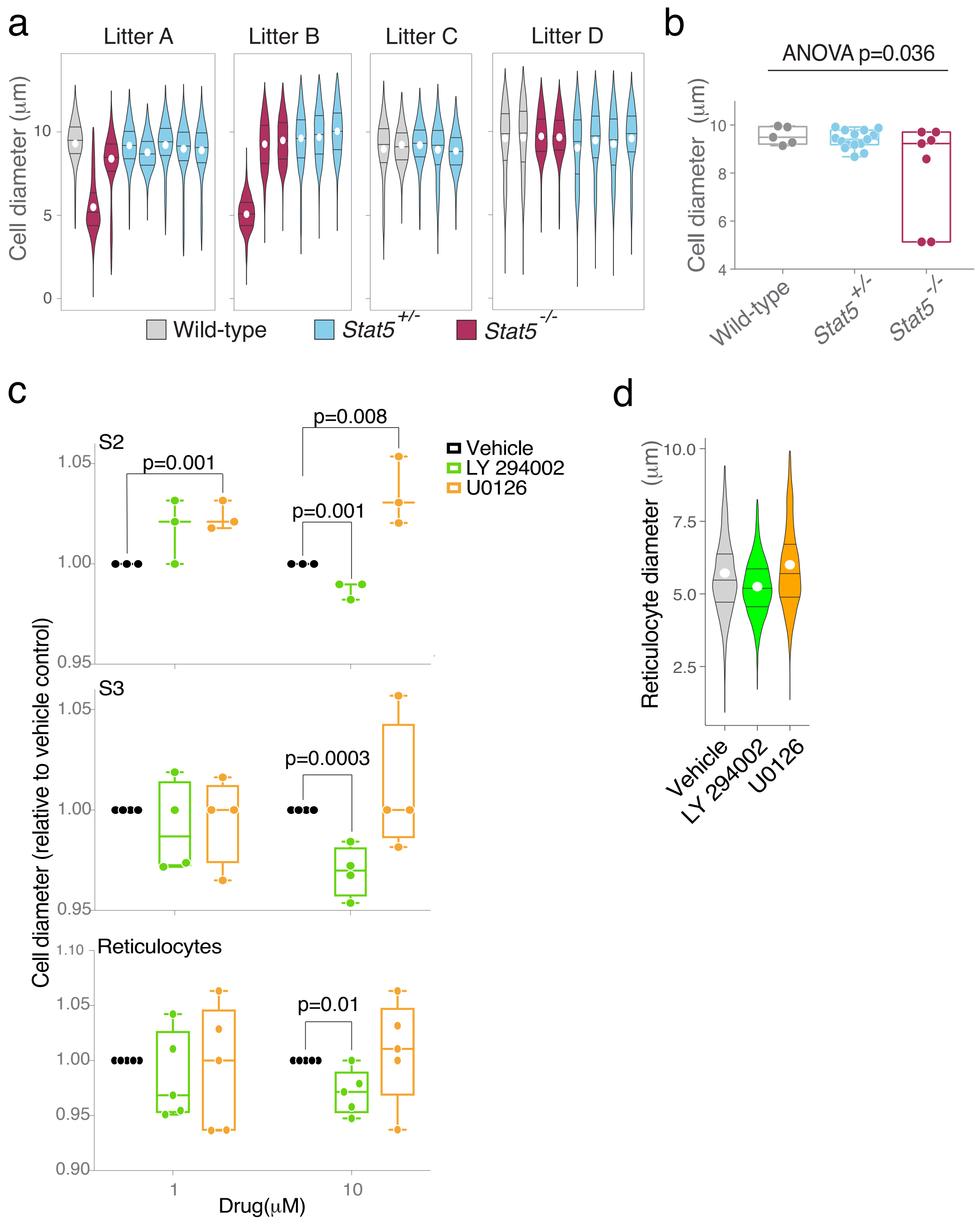

Supplementary Figure 13 EpoR regulates cell size via multiple signaling pathways

a Cell diameters of circulating red blood cells in E13.5 littermate embryos from matings of *Stat5*$^{+/-}$ x *Stat5*$^{+/-}$ mice. Violin lines mark the 25th, 50th and 75th percentile with a white circle marking the mean.

b Median cell diameters of circulating red blood cells from panel ‘a’, by genotype. The box and whiskers mark the 25th to 75th percentiles and min to max values, respectively with the median indicated. p value is one-way ANOVA. The data is pooled from a total of n= 5 wild type, n= 7 Stat5$^{-/-}$, and n= 15 Stat5$^{+/-}$ embryos, derived from n= 4 litters, as shown in panel ‘a’.

c Effect of inhibiting either PI3 kinase (with LY 294002) or MEK1/MEK2 (U0126) on the cell diameter of S2 and S3 erythroblasts and on reticulocytes. S0 cells were isolated from E13.5 wild-type fetal liver and differentiated *in vitro* in the presence of either vehicle (DMSO) or with inhibitors at the indicated concentrations. Diameters were measured at 48 h. Data are population medians from 3-5 experiments for each inhibitor concentration. Box and whiskers mark the 25th to 75th percentiles and min to max values respectively, with the median indicated. p values are for unpaired 2-tailed t-test. P value for one-way ANOVA is <0.0001.

d Violin plot of reticulocyte diameters for a representative experiment from ‘c’, inhibitor concentrations were each 10μM. Violin lines mark the 25th, 50th and 75th percentile with a white circle marking the mean. P value is for upaired t test. One -way ANOVA p value for all samples is <0.0001

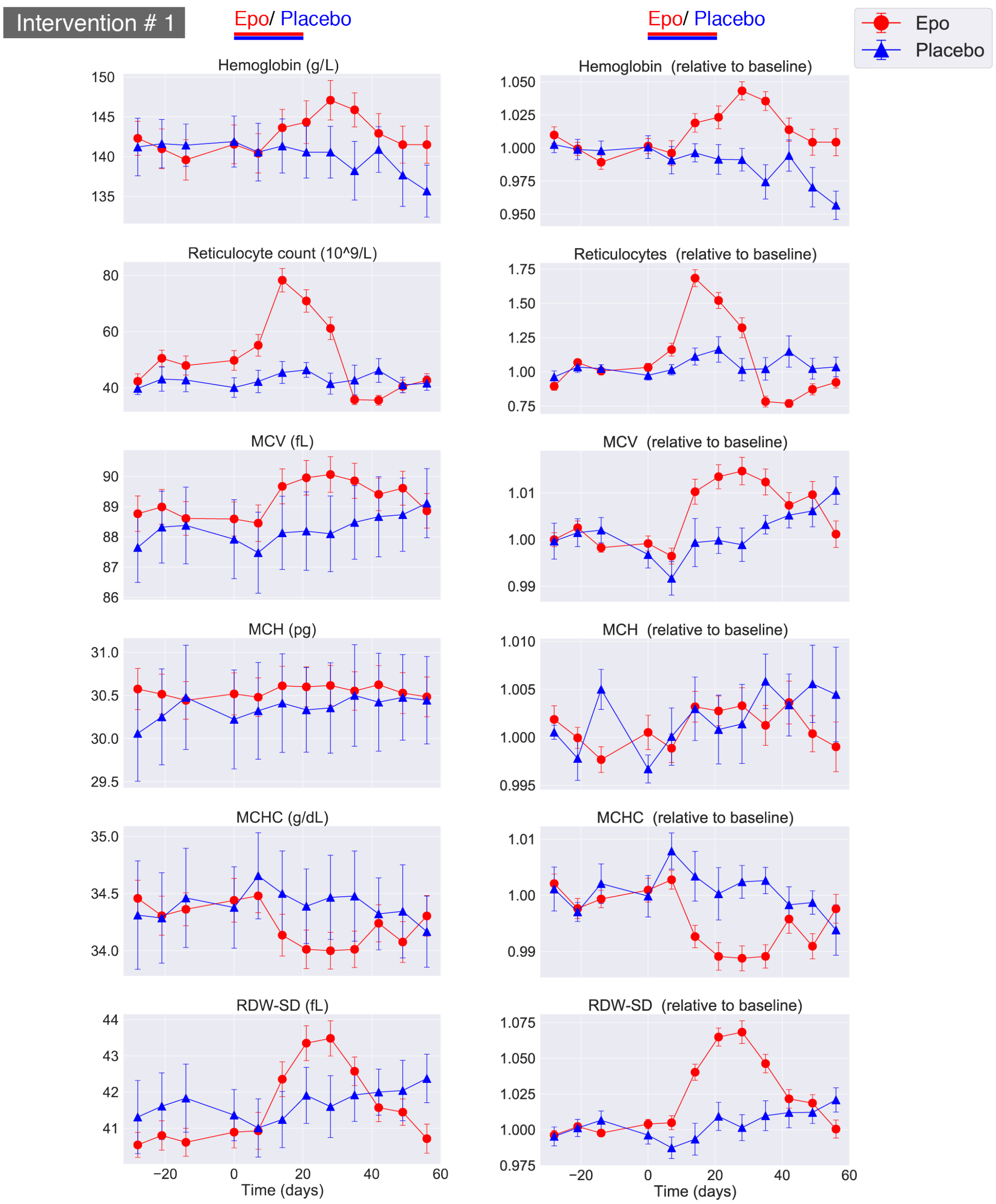

Corrected p values						
Day	Hemoglobin	Reticulocyte count	MCV	MCH	MCHC	RDW-SD
7						
14		0.0001			0.0289	0.00017
21		0.0005	0.0218		0.0289	<0.0001
28	0.003	0.0192	0.0097		0.0069	<0.0001
35	0.00079	0.0007			0.0069	0.0044
42		0.00026				
49						
56	0.006					

significantly higher
significantly lower

Supplementary Figure 14 Epo increases MCV and RDW independently of reticulocyte counts (University of Copenhagen, Denmark)

Hematological parameters in intervention study 1. An initial 4 weekly venopuncture sampling period was followed by intravenous injections every 48 hours for 3 weeks, of either Epo (epoetin alpha, 20 IU·kg bw^{-1}) or placebo (the treatment period is indicated by red or blue horizontal bars). Venous blood samples were collected weekly during the treatment period and for five weeks following treatment. There were 25 participants in the Epo group (13 male and 12 female) and 9 participants in the placebo group (6 male, 3 female).

Hematological parameters are shown raw (left column) and also relative to baseline values for each participant (right column). Data are mean ± sem. The table shows p values for significant differences between Epo and placebo for each parameter and time point (linear mixed-effect model with Benjamini–Hochberg correction for multiple comparisons, see supplementary statistical analysis).

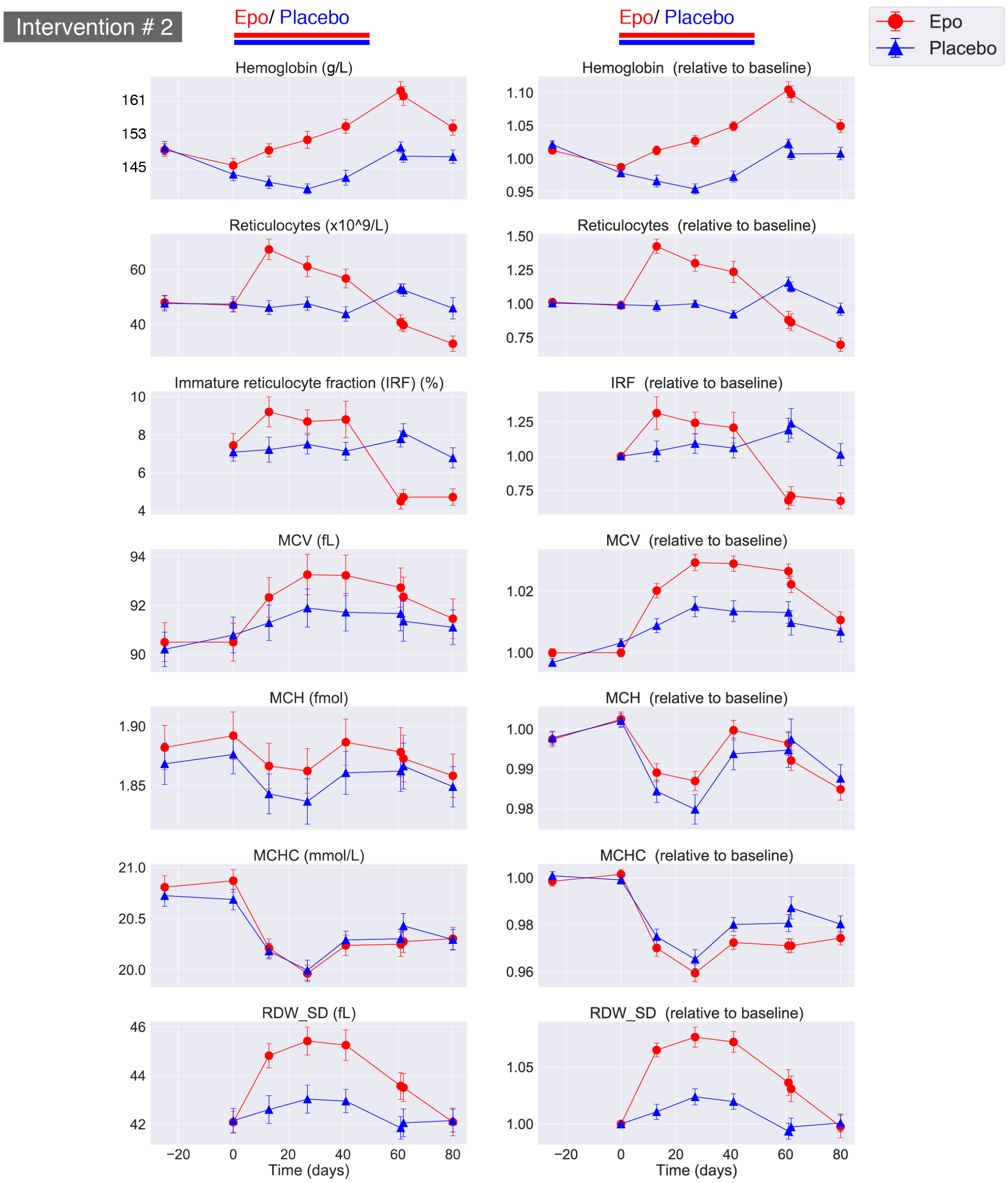

Corrected p values							
Day	Hemoglobin	Reticulocyte count	IRF	MCV	MCH	MCHC	RDW-SD
13	0.0002	<0.0001		0.0071			<0.0001
27	<0.0001	0.0001		0.003			<0.0001
41	<0.0001	0.0002		0.0019			<0.0001
61	<0.0001	0.0004	0.0002	0.0049			0.0004
62	<0.0001	0.0004	0.0002	0.0071		0.0153	0.0032
80	0.006	0.0002	0.0051				

significantly higher
significantly lower

Supplementary Figure 15 Epo increases MCV and RDW independently of reticulocyte counts (Centre for Human Drug Research, Leiden, Netherlands)

Hematological parameters in intervention study 2. Two baseline venopuncture sampling measurements were followed by weekly dosing with Epo (24 male subjects) or placebo (24 male subjects) for 7 weeks. Epo dosing (epoetin β, 5000 to 10000 IU) was adjusted for each subject, to achieve an increase of 10 to 15% in hemoglobin over baseline. Follow-up continued for a month after cessation of treatment.

Hematological parameters are shown raw (left column) and also relative to baseline values for each participant (right column). Data are mean ± sem. The table shows p values for significant differences between Epo and placebo for each parameter and time point (linear mixed-effect model with Benjamini–Hochberg correction for multiple comparisons, see supplementary statistical analysis).

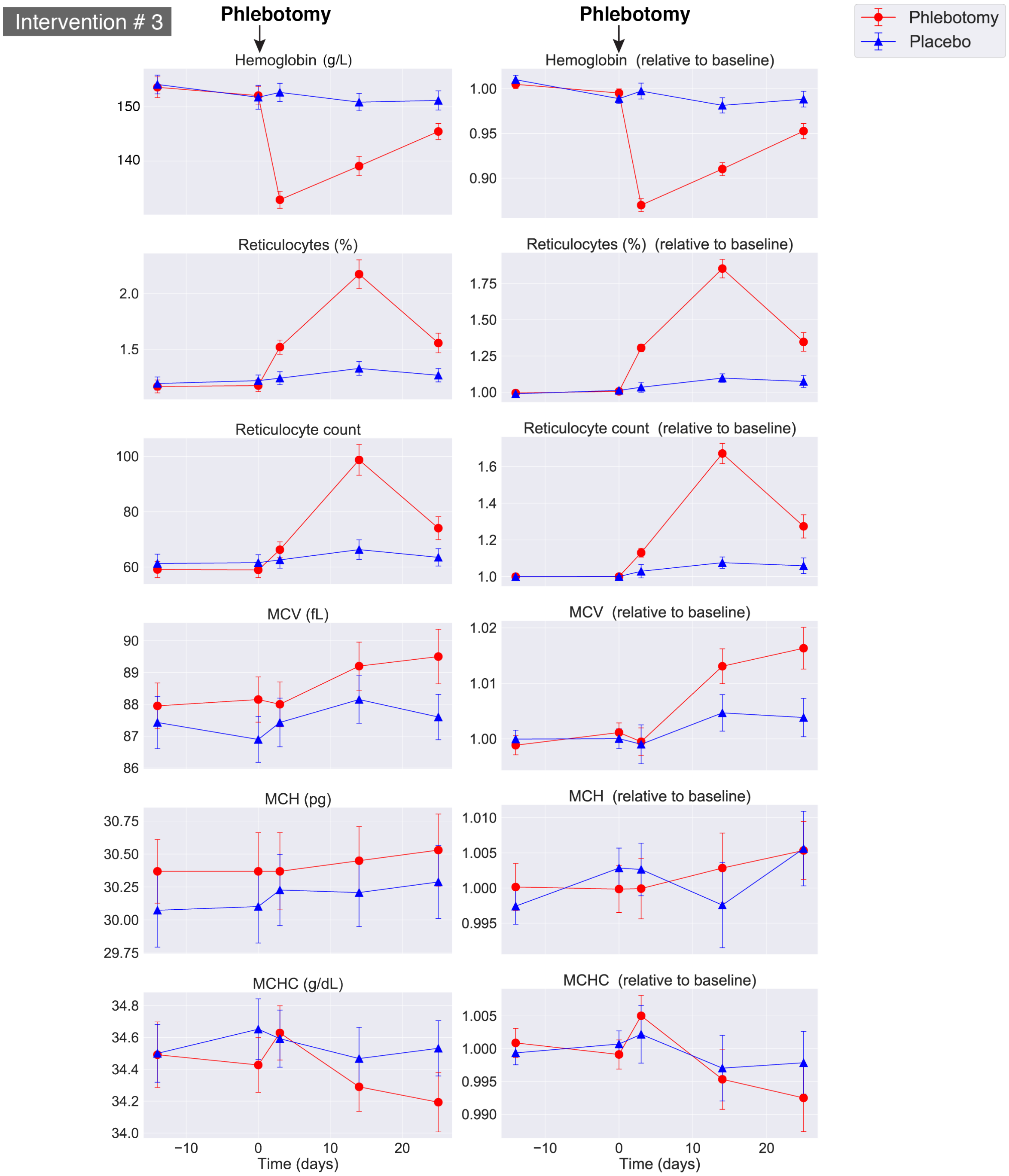

Corrected p values					
Day	Hemoglobin	Reticulocyte count	MCV	MCH	MCHC
3	<0.0001	0.03			
14	<0.0001	<0.0001			
25	0.0105	0.0047	0.0188		

significantly higher
significantly lower

Supplementary Figure 16 Phlebotomy results in increased MCV independently of reticulocyte counts (University of Copenhagen, Denmark)

Hematological parameters in intervention study 3. This was a single blind, placebo-controlled cross-over study, with 21 participants. Two baseline venopuncture sampling measurements 4 days apart were made in the week preceding phlebotomy. At t=0, participants were either phlebotomized of two whole-blood units (900 ml), or sham-phlebotomized. Venous blood was sampled 3, 14 and 25 days later. At least 4 months elapsed before cross-over.

Hematological parameters are shown raw (left column) and also relative to baseline values for each participant (right column). Data are mean ± sem. The table shows p values for significant differences between Epo and placebo for each parameter and time point (linear mixed-effect model with Benjamini–Hochberg correction for multiple comparisons, see supplementary statistical analysis).

Supplementary References for Suppelmentary Figures:

1. Tusi, B.K.*, et al.* Population snapshots predict early haematopoietic and erythroid hierarchies. *Nature* **555**, 54-60 (2018).

Supplementary analysis:

Simulations of MCV time course during and following Epo administration

Abstract

Our work shows that EpoR signaling increases erythroblast cell size at every stage of erythroid terminal differentiation, leading to the formation of larger reticulocytes. Two human intervention studies in which Epo is administered to healthy volunteers show an increase in MCV which could not be attributed to reticulocytosis, suggesting that Epo/EpoR signaling also increases red cell (RC) size in human erythropoiesis. However, an alternative explanation is that the observed increase in MCV is the result of skewing of the circulating RC pool in favor of younger, larger RCs, since RCs loose volume with aging. Here we consider this alternative possibility quantitatively. We simulate changes in MCV that would be predicted based on published parameters for the rate of volume loss during reticulocyte maturation and RC aging, including the early release of immature reticulocytes into the circulation in response to high Epo. Our simulation suggests that these factors can only account for 30 to 50% of the overall increase in MCV observed in the human studies. Therefore, additional factors, including EpoR signaling, contribute to the observed increase in cell size in response to Epo.

I Introduction

In human intervention studies, we found that Epo administration led to increased MCV. Contrary to expectation, there was no correlation between MCV and reticulocyte count (Supplementary Figures 14,15 and Figure 6; statistical analysis supplement). Two hypotheses might account for the increase in MCV:

The first, or *null hypothesis*, is based on the known negative correlation between red cell (RC) age and volume. Although the largest loss in RC volume occurs during reticulocyte maturation, RCs continue to lose volume throughout their life (in part as a result of vesiculation)[1-4]. In the steady state, the MCV is the average of the volume of an equal number of RCs of every age. In the period following Epo administration, the immediate increase in reticulocytes is followed, as these reticulocytes mature into RCs, by an increase in the relative number of younger, and therefore larger, RCs. This skew in the age distribution of RCs is expected to increase the MCV beyond the time of increased reticulocytes and may be sufficient to account for the observed increase that we see in the human studies.

In the *alternative hypothesis,* the observed increase in MCV cannot be accounted for solely by the skewing of the RC age distribution. We propose that it is therefore also the result of EpoR-signaling -mediated increase in cell size. This hypothesis is based on our experimental findings in the mouse, which show that EpoR signaling increases cell size in an Epo- dose dependent manner at every stage of erythroid terminal differentiation (ETD), leading to the production of larger reticulocytes and RCs.

Here we simulated the null hypothesis, and then determined whether it is sufficient to account for the observations in the human studies. Specifically, we simulated the time course of expected changes in MCV based on known Epo- induced changes in circulating reticulocyte number, their time of release from the bone marrow and time in circulation. We repeated the simulation for a range of erythropoietic rates, and for multiple Epo treatment durations, as well as for different red cell life- span values. This analysis showed that the increase in MCV predicted by the null hypothesis accounts for only 30 to 50% of the observed increase. We therefore conclude that additional factors contribute to the increase in MCV. These findings are consistent with our experiments in the mouse, which show an EpoR signaling- mediated increase in cell size.

II Results

We used the null hypothesis to simulate the time course of MCV and reticulocyte number, during and following Epo treatments that increase erythropoietic rate by up to 10 fold (see 'Methods and rationale' section). The null hypothesis posits that Epo treatment leads to an increase in the number of new reticulocytes generated per day, but does not change reticulocyte or RC size. We also incorporated into the simulation the early release of immature, larger reticulocytes from the bone-marrow niche during high erythropoietic rates, which results in increased size of circulating reticulocytes and increased reticulocyte circulation time [5-7].

To compare the human intervention studies with our simulation, we estimated the increase in erythropoietic rate in each of the studies. We did this by matching the observed increase in reticulocytes to the increase predicted by the simulations.

During human intervention study #1 (**Figure 1**), participants were administered with 20 IU/kg Epoetin alpha every 48 hours for 3 weeks (~70 IU/kg Epoetin alpha / week). Circulating reticulocytes increased by an average of 1.5 fold (weekly averages were 1.68, 1.52 and 1.32 fold). These values suggested an increase in erythropoietic rate of 1.25 to 1.5 -fold, which in our simulation resulted in 1.32 to 1.68 -fold increase in reticulocytes (**Figure 1,** lower panel). The observed increase in MCV was 1.47 ±0.29 %, double the corresponding simulated increase of 0.75 ±0.25% (upper and lower bounds of the simulated values correspond to the estimated range for erythorpoietic rate (Ery rate) of 1.25 to 1.5 fold). The predicted value for the post-treatment MCV was similarly lower than the observed value (**Figure 1,** upper panel, and **Table I**).

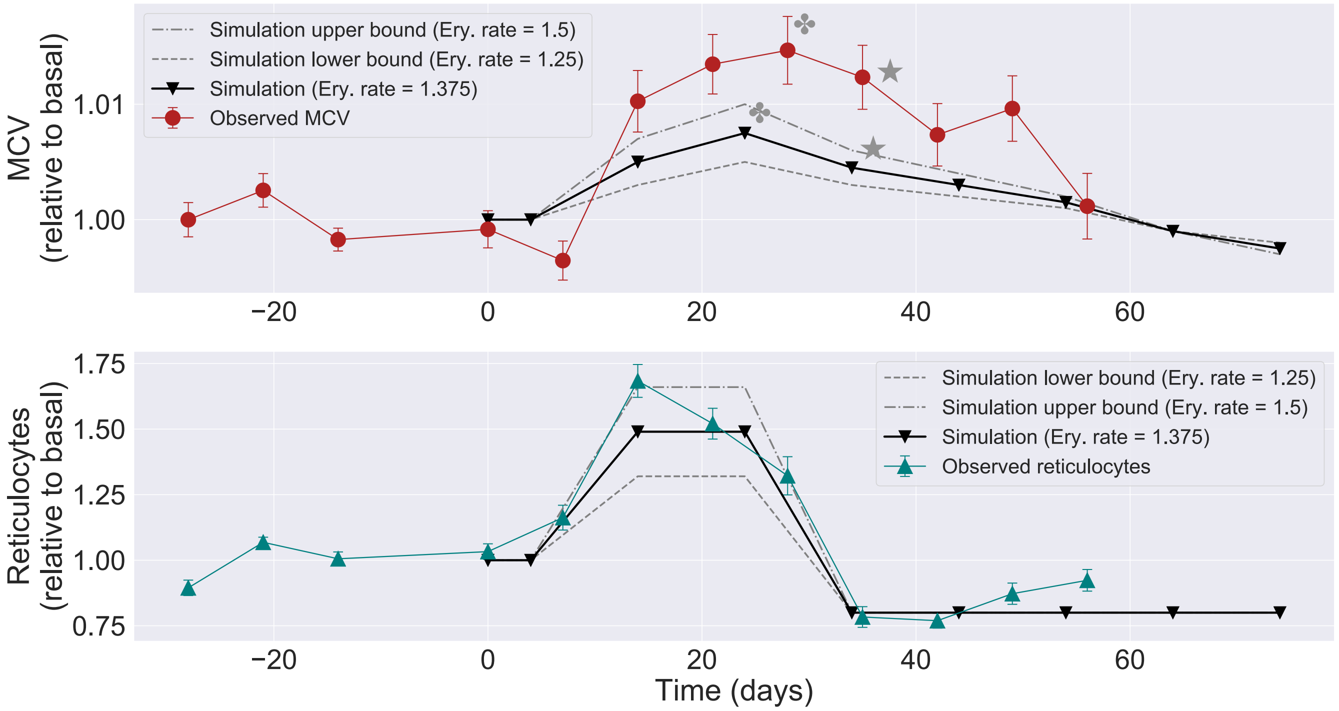

Figure 1: Observed and simulated MCV and reticulocytes, for human intervention study #1 (Supplementary Figure 14). Simulation was for Epo treatment for 20 days, increasing erythropoietic rate by 1.25 to 1.5-fold the basal rate.

**Table I**	**Increase in observed MCV (mean ±sem, n=25)**	**Simulated increase in MCV Ery rate = 1.375 (range: 1.25 -1.5)**
**peak** ✤	1.47 ±0.29%	0.75% (0.5% - 1.0%)
**post-treatment** ★	1.23 ±0.28%	0.5% (0.3%-0.7%)

For the simulated increase in MCV to match the observed increase in MCV would require a 1.75 -fold increase in erythropoietic rate, generating >2-fold increase in circulating reticulocytes, well above the observed reticulocyte number (simulation: green line in **Figure 2**; observed values in Figure1, lower panel).

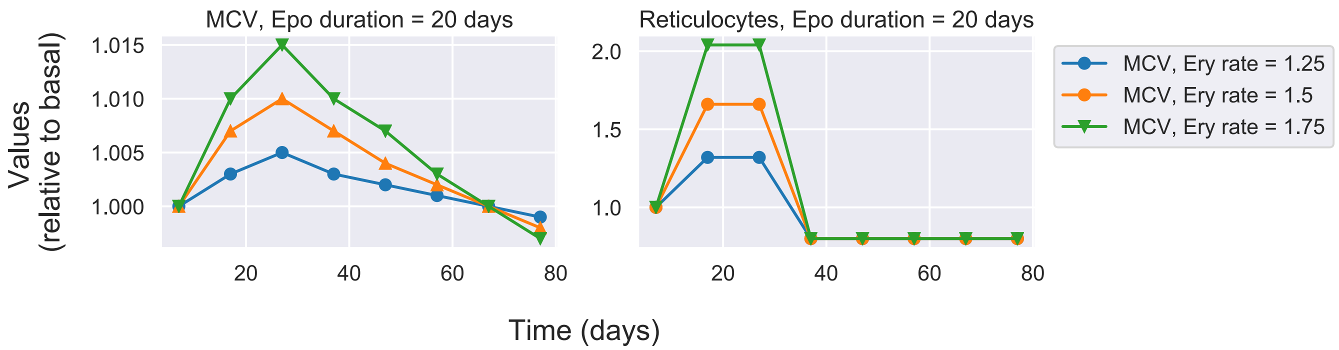

Figure 2: Simulation of MCV and reticulocyte number during and following a 20-day Epo treatment, for different levels of increase in erythropoietic rate.

In human intervention study #2 (**Figure 3**), participants were administered ~100 IU/kg Epoetin β weekly for 7 weeks. Observed reticulocyte counts increased 1.43, 1.30 and 1.24-fold, averaging a 1.32 -fold increase, which corresponds to a 1.25-fold increase in erythropoietic rate. The observed peak increase in MCV was 2.9 ±0.25 %, nearly three times the simulated increase of 1.0 ±0.3%. Similarly, the predicted post-treatment MCV was lower than the observed value (**Figure 3** and **Table II**).

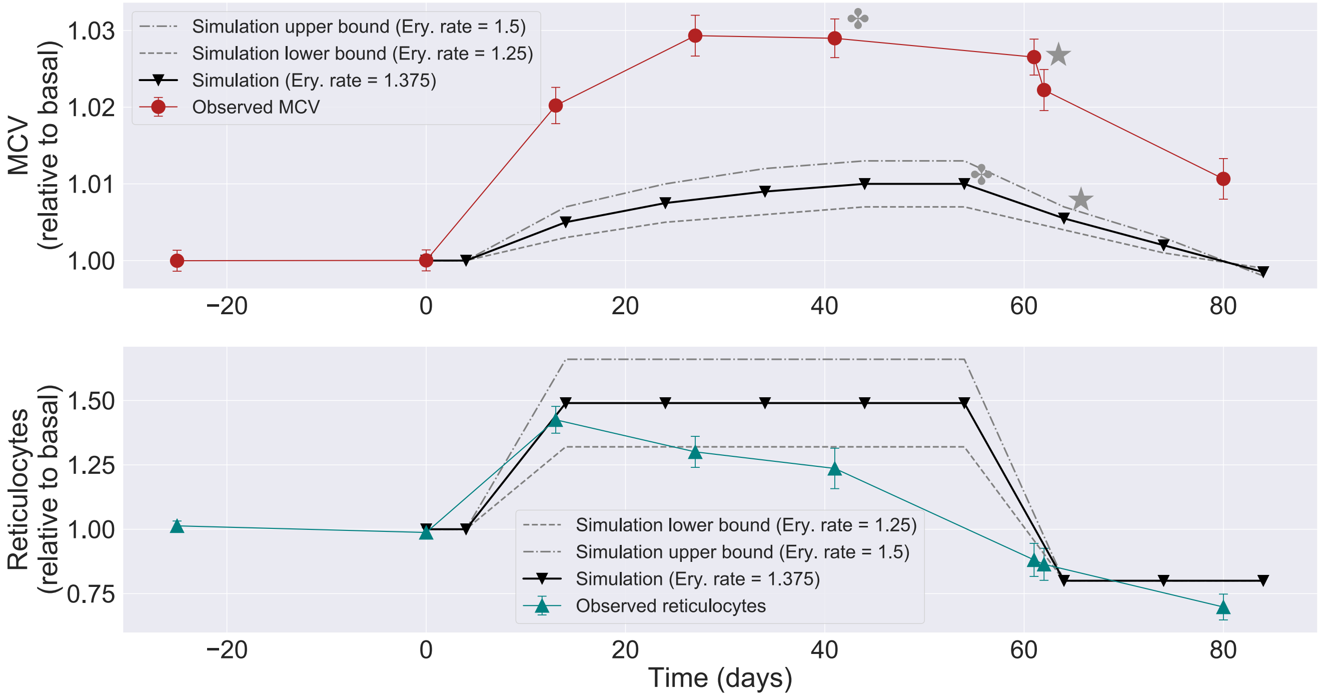

Figure 3: Observed and simulated MCV and reticulocytes, for human intervention study #2 (Supplementary Figure 15). Simulation was for Epo treatment for 50 days, increasing erythropoietic rate by 1.25 to 1.5-fold.

**Table II**	**Increase in observed MCV (mean ± sem, n=24)**	**Simulated increase in MCV Ery rate = 1.375 (range: 1.25 -1.5)**
**peak** ✣	2.93 ±0.25%	1.0% (0.7% - 1.3%)
**post-treatment** ★	2.65 ±0.23%	0.55% (0.4%-0.7%)

Even a 2-fold increase in erythropoietic rate, corresponding to a far larger than observed increase in reticulocytes, would not be sufficient to account for the observed increase in MCV (red line, **Figure 4**).

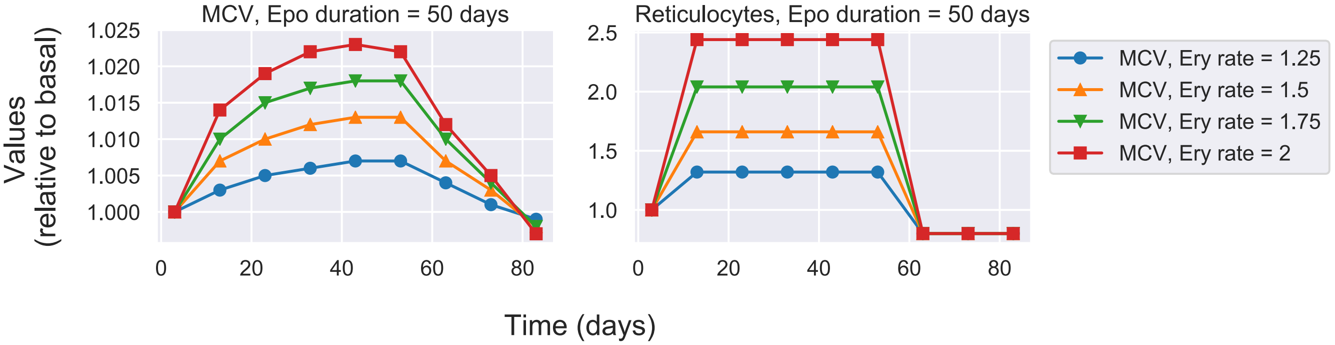

Figure 4: Simulation of MCV and reticulocyte number during and following a 50-day Epo treatment, for different levels of increase in erythropoietic rate.

The simulations considered above assumed that RC lifespan is 100 days. We repeated the simulation for a range of RC lifespans, from 95 days to 120 days (**Figure 5**). We did this in two different ways. In Figure 5, we assumed that overall RC volume decreased by the same extent over the RC lifespan, regardless of lifespan duration. We also simulated an alternative, in which the rate of RC volume loss was constant throughout the RC lifespan, so that a larger overall volume is lost if lifespan is longer. In either case, there was little effect on the MCV time course. In simulating human study #2, peak MCV was slightly higher for RC lifespan of 120, compared with RC lifespan of 95 days (peaking at a 1.3% increase instead of 1.2%) but this was still well short of the observed, 2.9% increase (**Figure 5**, lower panel).

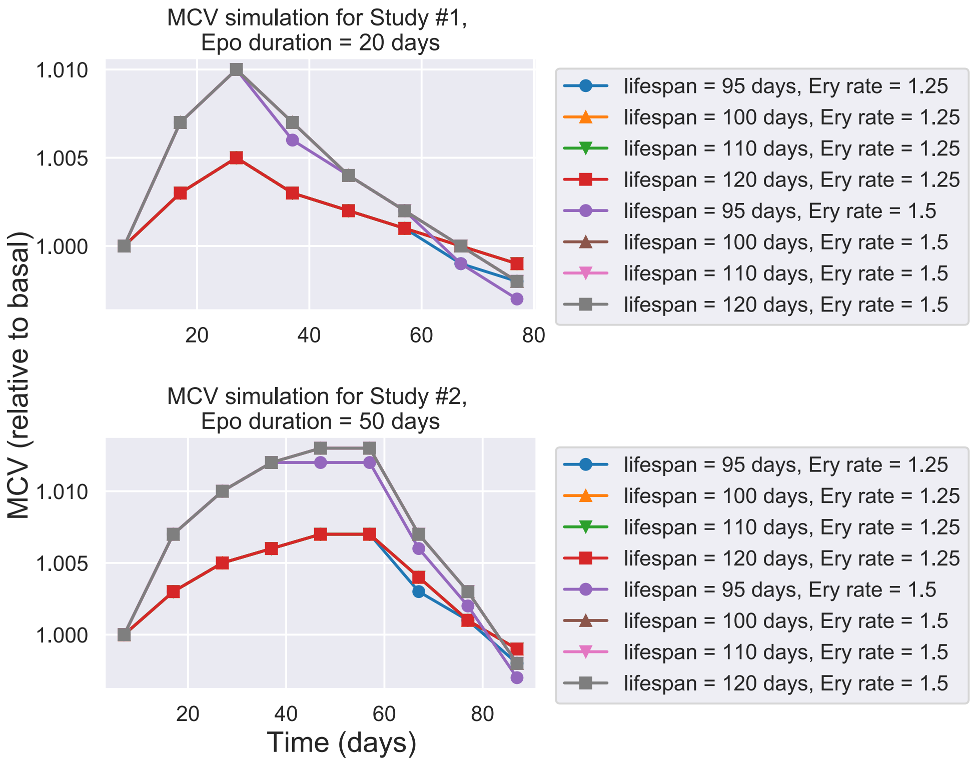

Figure 5: The effect of RC lifespan on the MCV time-course during and following Epo treatments, simulating human intervention studies #1 (top) and #2 (bottom). RC lifespan is indicated. We simulated an upper bound (Ery rate= 1.5 fold) and a lower bound (Ery rate= 1.25 fold) for each study and RC lifespan combination. Note that where MCV values are identical, overlaid symbols are hidden.

III Conclusions

Simulation of the change in MCV in response to Epo treatment using the null hypothesis accounts for only 30% to 50% of the observed increase in MCV, in two human intervention studies. We conclude that additional factors contribute to the Epo -driven increase in MCV. This finding is consistent with our experimental findings in the mouse, that in addition to increase RC number, EpoR signaling promotes an increase in erythroblast and RC size.

IV Simulation Method and Rationale

Code:

The python script used for this simulation is available at https://github.com/socolovm/Simulation-of-MCV

Calculation of MCV: general principle

Reticulocyte and RC volumes decrease as they age. The MCV is the average volume of all circulating reticulocytes and RCs. Therefore, to calculate the MCV, we first need to know the characteristic RC volume corresponding to each RC age over the entire life span (approximately 100 days). Second, we need to know the number of RCs of each age in the circulation. The MCV is then calculated simply by multiplying the number of cells of each age by their corresponding volume, adding this up across all RC ages, and dividing by the total number of RCs and reticulocytes.

Units used in simulation

Cell volumes, cell number and erythropoietic rate are all expressed relative to their value in the basal state. Specifically, the MCV, erythropoietic rate, and reticulocyte number in the basal state are each set to '1'. This is similar to the way we expressed observations in the human intervention studies (Figure 6h-i and Supplementary Figures 14-16). We considered erythropoietic rates in the range 1 to 10, which covers the entire physiological and stress range of erythropoiesis.

Choice of parameter values

Current literature suggests that, starting with circulating reticulocytes, there is an overall ~30% decrease in volume across RC lifespan [1, 2, 8]. This loss happens in two phases: a rapid loss of 10 to 14% in circulating reticulocytes, during the course of ~1 day; and an additional, slower 16-17% loss during the rest of RC life [3] (the surface area/volume ratio remain constant [3]). The loss in volume over the life of the RC is linear, as seen from the linear decline in volume with RC Hemoglobin A1c (an indicator of RC age), and from the linear decline in flow-cytometric forward scatter with RC age [4].

We used this information to model the relationship between RC volume and age. Because there is a range of values reported in the literature for the extent of volume loss during the reticulocyte stage (10 to 14%) v. mature RCs (the remainder from the total volume loss of 30%), we set the loss in reticulocyte volume close to the minimum reported experimentally (11%), and set the additional volume lost by mature RCs over their lifespan to 20%, at the very highest limit reported. This choice favors the null hypothesis, since it maximizes the chance that changes in the MCV are the result of skewing of the mature (non-reticuloctye) RC population. Based on published work, we set the volume of basal-state reticulocytes during their one day of maturation in the circulation to 1.25 -fold the basal MCV [9, 10].

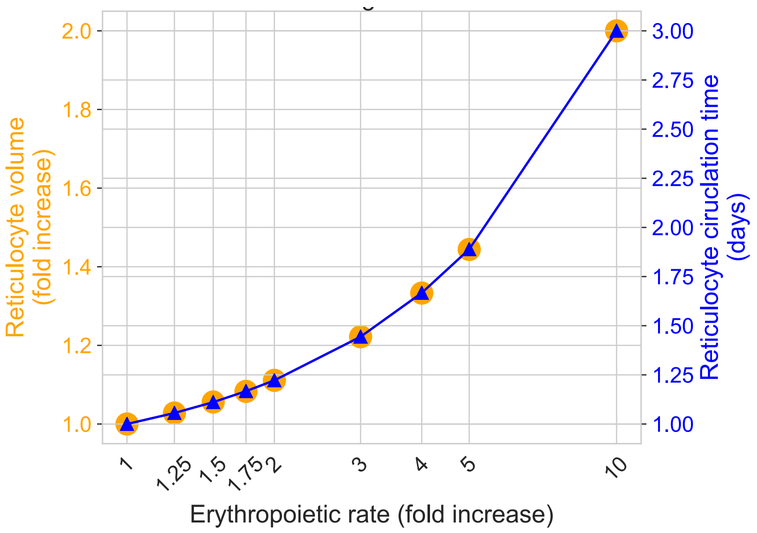

Figure 6: Increasing erythropoietic rate results in release of immature, larger reticulocytes from the bone marrow, with longer maturation times in the circulation. (NB Reticulocyte volumes here are relative to the baseline reticulocyte volumes)

Relationship between erythropoietic rate,

reticulocyte volumes and reticulocyte circulation times

In the basal state, reticulocytes mature over 3.5 - 4 days, with only the last 24 hours of this process taking place in the circulation. Increased erythropoietic rate leads to earlier release of immature, and therefore larger, reticulocytes from the bone marrow [5-7]. In response to maximal levels of stress, as seen in aplastic anemia, or in phenylhydrazine treatment in animals, reticulocyte volumes double [10], and their maturation time in the circulation increases approximately linearly with falling hematocrit [5,11]. We used these data to determine the relationship between erythropoietic rate, circulating reticulocyte volumes, and increased reticulocyte maturation time in the circulation (**Fig 6**).

Relationship between RC volume and RC age, in the basal state and following Epo administration

The null hypothesis posits that RC volume is not altered by Epo stimulation. The only change takes place at the reticulocyte stage. We used the relationship computed in **Figure 6** between erythropoietic rate, reticulocyte volume and reticulocyte circulation time, to set up the relationship between RC

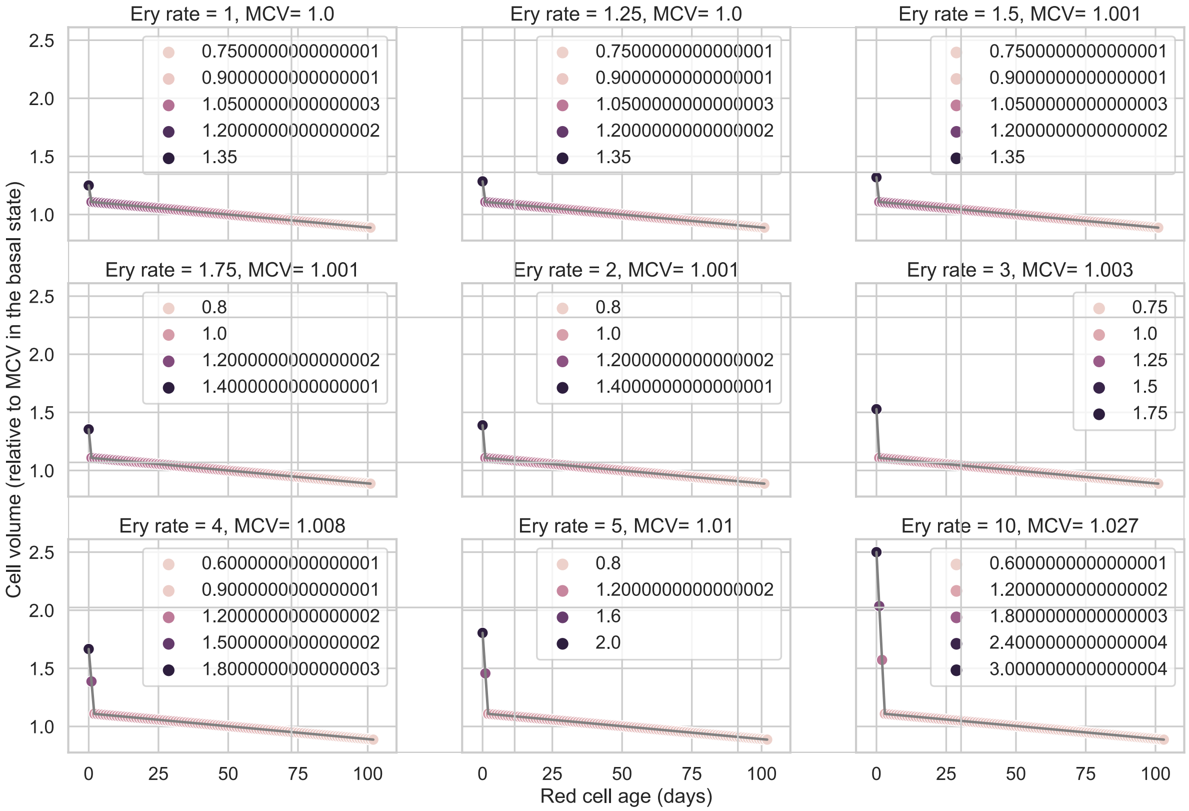

Figure 7: RC and reticulocyte volume, as a function of age, for different erythropoietic rates. Legend refers to RC volumes, relative to MCV in the basal state. In the basal state (Erythropoietic rate = 1, top left panel), rapid loss of volume at the reticulocyte stage (age = 1 day) is followed by a slower, linear loss for the remaining RC lifespan. Higher erythropoietic rates during stress lead to earlier release of larger, immature reticulocytes form the bone marrow with longer maturation times in the circulation.

volume and RC age, across reticulocyte maturation time and RC lifespan of 100 days, for the basal state and for a range of erythropoietic rates (**Fig 7**). We also simulated lifespans of 95, 110 & 120 days.

RC volume distributions and MCV during and following Epo administration

The MCV is the mean value of the circulating RCs at a given point in time. In the steady state, which can be defined as a constant erythropoietic rate (whether high or low) lasting for at least as long as the

RC lifespan, there is an equal number of RCs of every age. The MCV is therefore the mean value of all RC volumes of every age, as depicted in **Figure 7** and the top panel of **Figure 8**.

Figure 8 illustrates the RC volume distributions during and following an Epo treatment shorter than the RC lifespan. In this example, erythropoietic rate increased by 1.5 fold for 30 days. During each of the 30 treatment days, the number of new reticulocytes increases by 1.5 fold relative to the basal state. To obtain the RC volume distribution on day 30 (last day of treatment, second panel from top), we made use of the volume/age relationship in **Figure 8**, corresponding to an erythropoietic rate = 1.5 (the top right panel). RCs and reticulocytes whose age is ≤ 30 days are present in the circulation at 1.5 fold their number in the basal state; whereas all older cells are present in the same numbers as in the basal state. This creates a RC distribution that is skewed in favor of younger, larger cells (2nd panel, red curve), increasing the MCV to 1.012. On day 60, the number of RCs younger than 30 days is the same as would be found in the basal state, since they were generated after Epo treatment had terminated; cells older than 30 days but younger than 60 days are present at 1.5 fold the basal numbers, since they were generated during the period of Epo treatment; and finally, all cells older than 60 days are present in the same numbers as in the basal state (3rd panel, purple curve). MCV at this time point is almost back to normal, since the ‘bump’ in RC numbers involves cells whose volumes are near the mean. Finally, by day 90, cells whose age is >60 days are present at 1.5 -fold the number of all other cells. These older cells are smaller than the mean, bringing the MCV down below 1 (lowest panel, black curve).

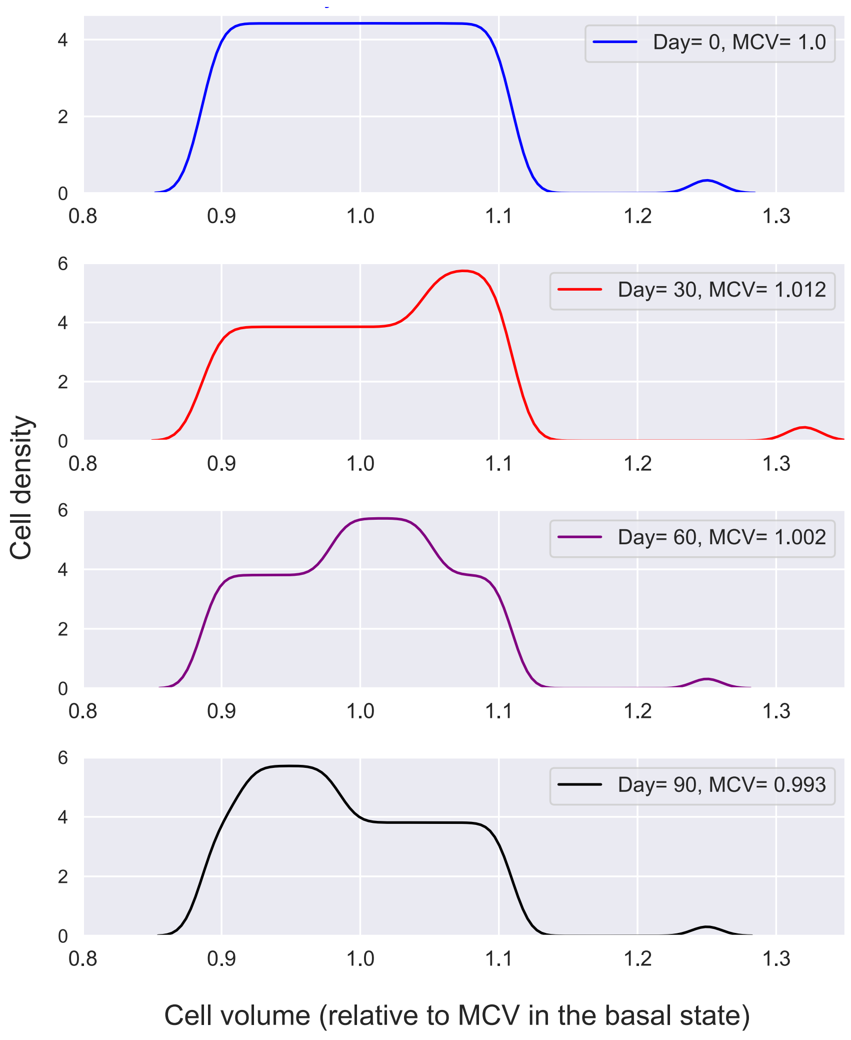

Figure 8: RC volume distributions during and following a 30 day Epo treatment in which erythropoietic rate increased by 1.5 fold. The MCV is computed as the mean of each of these distributions. Note that before the start of treatment (day 0, blue curve, top panel) the distribution is flat, since there are equal numbers of RCs of every age and volume. On day 30 (red curve, second panel from top) the distribution is skewed towards younger (≤30 days), larger cells, since there is a 1.5 fold increase in their number. By day 60 (purple curve, third panel from top), cells generated during the Epo treatment period have aged and are in the middle of the volume distribution. Finally, on day 90, these cells are skewing the distribution in favor of older cells with smaller volumes, reducing the MCV below the starting basal value. Although volume changes are continuous, for simplicity we considered them as occurring at daily intervals (see Fig 2). The large daily volume change during reticulocyte maturation therefore result in a multi-phasic distribution.

MCV and reticulocyte time course during and following Epo treatment

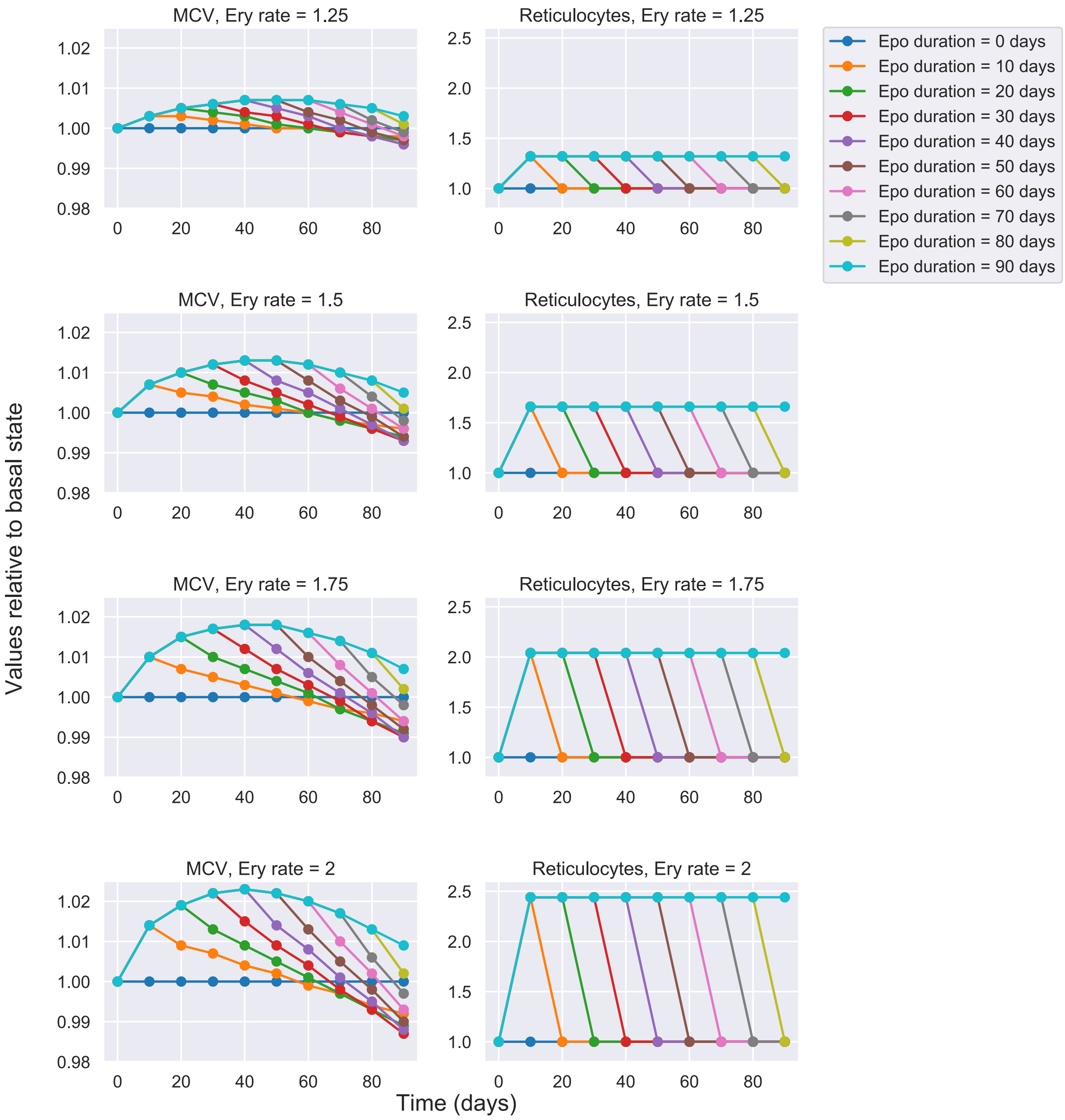

Figure 9: Time course simulation of changes to MCV and to circulating reticulocytes, during and following Epo treatment, starting on day=0 and lasting for the indicated number of days. Erythropoietic rate increased as a result of Epo treatment by 1.25 to 2 -fold.

We used the approach illustrated in **Figure 8** to compute the MCV at 10-day intervals, during and following Epo treatments of various durations, for erythropoietic rates between 1 and 10 -fold the

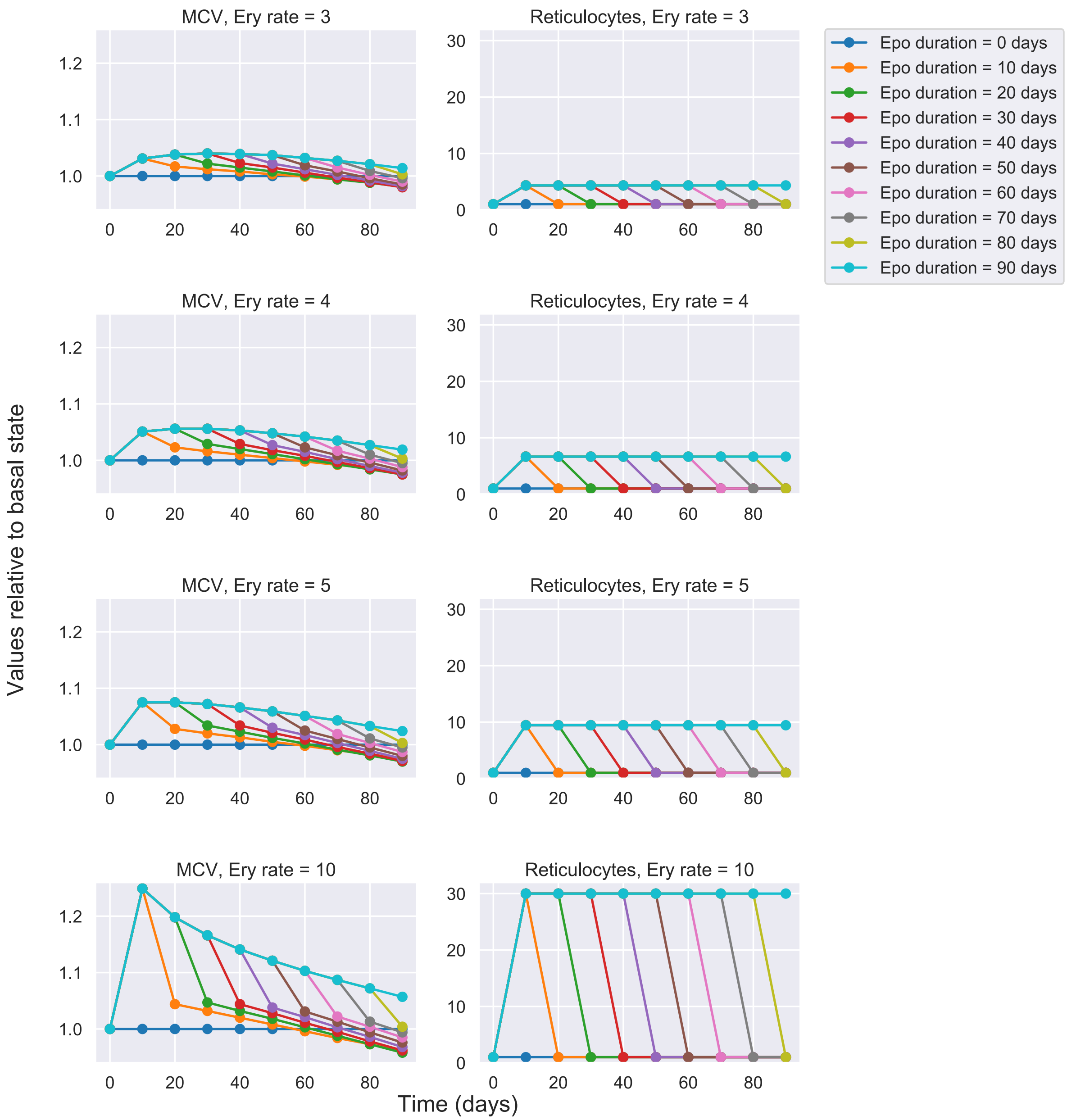

Figure 10: Time course simulation of changes to MCV and to circulating reticulocytes, during and following Epo treatment, starting on day=0 and lasting for the indicated number of days. Erythropoietic rate increased as a result of Epo treatment by 3 to 10 -fold.

basal rate (**Figures 9, 10**). To simulate the expected MCV changes in the human intervention studies, we used the reticulocyte count to estimate erythropoietic rate during the study; and predicted MCV values for the relevant erythropoietic rate, treatment duration and time point.

V Supplementary References for the 'Simulation of MCV' section:

1. Bosch, F.H., *et al.* Characteristics of red blood cell populations fractionated with a combination of counterflow centrifugation and Percoll separation. *Blood* **79**, 254-260 (1992).
2. Willekens, F.L., *et al.* Hemoglobin loss from erythrocytes in vivo results from spleen-facilitated vesiculation. *Blood* **101**, 747-751 (2003).

3. Gifford, S.C., Derganc, J., Shevkoplyas, S.S., Yoshida, T. & Bitensky, M.W. A detailed study of time-dependent changes in human red blood cells: from reticulocyte maturation to erythrocyte senescence. *Br J Haematol* **135**, 395-404 (2006).
4. Franco, R.S.*, et al.* Changes in the properties of normal human red blood cells during in vivo aging. *Am J Hematol* **88**, 44-51 (2013).
5. Hillman, R.S. Characteristics of marrow production and reticulocyte maturation in normal man in response to anemia. *J Clin Invest* **48**, 443-453 (1969).
6. Major, A., Bauer, C., Breymann, C., Huch, A. & Huch, R. rh-erythropoietin stimulates immature reticulocyte release in man. *Br J Haematol* **87**, 605-608 (1994).
7. Rhodes, M.M.*, et al.* Stress reticulocytes lose transferrin receptors by an extrinsic process involving spleen and macrophages. *Am J Hematol* **91**, 875-882 (2016).
8. Willekens, F.L., Bosch, F.H., Roerdinkholder-Stoelwinder, B., Groenen-Döpp, Y.A. & Werre, J.M. Quantification of loss of haemoglobin components from the circulating red blood cell in vivo. *Eur J Haematol* **58**, 246-250 (1997).
9. d'Onofrio, G.*, et al.* Simultaneous measurement of reticulocyte and red blood cell indices in healthy subjects and patients with microcytic and macrocytic anemia. *Blood* **85**, 818-823 (1995).
10. Brugnara, C. Use of reticulocyte cellular indices in the diagnosis and treatment of hematological disorders. *Int J Clin Lab Res* **28**, 1-11 (1998).
11. Finch, C.A. Erythropoiesis, erythropoietin, and iron. *Blood* **60**, 1241-1246 (1982).

Code

Data analysis of Epo intervention study # 1, Hidalgo et al.

By Lihua Julie Zhu

Citation:

1. Lindstrom, M.J. and Bates, D.M. (1988) "Newton-Raphson and EM Algorithms for Linear Mixed-Effects Models for Repeated-Measures Data", Journal of the American Statistical Association, 83, 1014–1022.
2. Hothorn T, Bretz F, Westfall P (2008). "Simultaneous Inference in General Parametric Models." Biometrical Journal, 50(3), 346–363.
3. Benjamini, Y., and Hochberg, Y. (1995). Controlling the false discovery rate: a practical and powerful approach to multiple testing. Journal of the Royal Statistical Society Series B, 57, 289–300.http://www.jstor.org/stable/2346101.

Data summary

```
[1] "Thu Mar 19 18:32:15 2020"
[1] 407  11
```

	Treatment <fctr>	Subject <int>	time_point <fctr>	HbgdL <dbl>	MCHpg <dbl>	MCHCgdL <dbl>	MCVfl <dbl>	RDWCV <dbl>	RDWSDfl <dbl>
1	EPO	1	Baseline 1	12.73875	31.92750	33.70125	94.7	12.0	42.3
2	EPO	1	Baseline 2	12.25500	32.18550	34.50750	93.2	12.2	42.2
3	EPO	1	Baseline 3	12.73875	32.04037	33.86250	94.6	12.1	42.4
4	EPO	1	Baseline 4	12.80000	31.50000	33.10000	95.0	12.0	42.4
5	EPO	1	EPO wk1	13.10000	32.00000	34.10000	94.1	12.2	42.1
6	EPO	1	EPO wk2	13.00000	32.40000	34.00000	95.5	12.3	43.7

6 rows | 1-10 of 11 columns

```
Correlation matrix among different measurements
                  HbgdL       MCHpg      MCHCgdL       MCVfl       RDWCV     RDWSDfl    Retics
HbgdL       1.000000000  0.10296731 -0.004754063  0.12475968 -0.06211799  0.03608573 0.0754058
MCHpg       0.102967313  1.00000000  0.555489534  0.77843006 -0.47903236  0.08661093 0.2558222
MCHCgdL    -0.004754063  0.55548953  1.000000000 -0.08842794 -0.52550521 -0.54151516 0.1870510
MCVfl       0.124759678  0.77843006 -0.088427942  1.00000000 -0.17906623  0.51124013 0.1649677
RDWCV      -0.062117994 -0.47903236 -0.525505208 -0.17906623  1.00000000  0.73977857 0.1235085
RDWSDfl     0.036085730  0.08661093 -0.541515158  0.51124013  0.73977857  1.00000000 0.1748552
Retics      0.075405804  0.25582219  0.187050951  0.16496774  0.12350851  0.17485524 1.0000000
Retics109L  0.091634764  0.19595026  0.224058174  0.06575297  0.11089097  0.09971701 0.9749060
           Retics109L
HbgdL      0.09163476
MCHpg      0.19595026
MCHCgdL    0.22405817
MCVfl      0.06575297
RDWCV      0.11089097
RDWSDfl    0.09971701
Retics     0.97490601
Retics109L 1.00000000
```

Compute mean of 4 baseline points for each subject

	Treatment <fctr>	Subject <int>	time_point <fctr>	HbgdL <dbl>	MCHpg <dbl>	MCHCgdL <dbl>	MCVfl <dbl>	RDWCV <dbl>	RDWSDfl <dbl>
1	EPO	1	Baseline 1	12.73875	31.92750	33.70125	94.7	12.0	42.3
2	EPO	1	Baseline 2	12.25500	32.18550	34.50750	93.2	12.2	42.2
3	EPO	1	Baseline 3	12.73875	32.04037	33.86250	94.6	12.1	42.4
4	EPO	1	Baseline 4	12.80000	31.50000	33.10000	95.0	12.0	42.4
5	EPO	1	EPO wk1	13.10000	32.00000	34.10000	94.1	12.2	42.1
6	EPO	1	EPO wk2	13.00000	32.40000	34.00000	95.5	12.3	43.7

6 rows | 1-10 of 19 columns

```
[1] 272  19
[1] EPO wk1  EPO wk2  EPO wk3  EPO wk4  post wk1 post wk2 post wk3 post wk4
12 Levels: Baseline 1 Baseline 2 Baseline 3 Baseline 4 EPO wk1 EPO wk2 EPO wk3 ... post wk4
```

	Treatment <fctr>	Subject <int>	time_point <fctr>	HbgdL <dbl>	MCHpg <dbl>	MCHCgdL <dbl>	MCVfl <dbl>	RDW... <dbl>	RDWSDfl <dbl>
5	EPO	1	EPO wk1	0.466875	0.08665625	0.3071875	-0.275	0.125	-0.225
6	EPO	1	EPO wk2	0.366875	0.48665625	0.2071875	1.125	0.225	1.375
7	EPO	1	EPO wk3	0.266875	0.58665625	0.5071875	0.125	0.325	1.775
8	EPO	1	EPO wk4	1.166875	0.38665625	0.0071875	1.325	0.725	2.575
9	EPO	1	post wk1	1.466875	0.08665625	0.0071875	0.425	0.125	0.875
10	EPO	1	post wk2	0.366875	-0.11334375	-0.1928125	0.325	0.025	0.175

6 rows | 1-10 of 19 columns

```
########Number of Subjects for each treatment ######
    EPO placebo
     25       9
```

```
#############################################################



##############################################################

########### Repeated measures analysis results for  HbgdL ############

###############################################################
```

	numDF<br><int>	denDF<br><dbl>	F-value<br><chr>	p-value<br><chr>
(Intercept)	1	217	3.475773	0.0636
Treatment	1	31	12.892589	0.0011
time_point	7	217	5.433239	<.0001
Treatment:time_point	7	217	2.009929	0.0551

4 rows

```
############ Compare Epo with Palcebo at each time point using contrasts ###########


###############################################################

######################## Raw p-values ########################


     Simultaneous Tests for General Linear Hypotheses

Multiple Comparisons of Means: User-defined Contrasts


Fit: lme.formula(fixed = thisY ~ -1 + EpoTime, data = y, random = ~1 |
    Subject)

Linear Hypotheses:
                          Estimate Std. Error z value Pr(>|z|)
EpoVsPlacebo.EpoWk1 == 0   0.06244    0.21614   0.289 0.772650
EpoVsPlacebo.EpoWk2 == 0   0.30550    0.21614   1.413 0.157519
EpoVsPlacebo.EpoWk3 == 0   0.45411    0.21614   2.101 0.035637 *
EpoVsPlacebo.EpoWk4 == 0   0.72911    0.21614   3.373 0.000742 ***
EpoVsPlacebo.postWk1 == 0  0.84161    0.21614   3.894 9.86e-05 ***
EpoVsPlacebo.postWk2 == 0  0.27911    0.21614   1.291 0.196576
EpoVsPlacebo.postWk3 == 0  0.45967    0.21614   2.127 0.033441 *
EpoVsPlacebo.postWk4 == 0  0.65967    0.21614   3.052 0.002272 **
---
Signif. codes:  0 ‘***’ 0.001 ‘**’ 0.01 ‘*’ 0.05 ‘.’ 0.1 ‘ ’ 1
(Adjusted p values reported -- none method)

##########################################################


############## BH adjusted p-values ####################
Benjamini, Y., and Hochberg, Y. (1995). Controlling the false discovery rate: a practical and powerful approach t
o multiple testing. Journal of the Royal Statistical Society Series B, 57, 289–300.http://www.jstor.org/stable/23
46101.
##############################################################

     Simultaneous Tests for General Linear Hypotheses

Multiple Comparisons of Means: User-defined Contrasts


Fit: lme.formula(fixed = thisY ~ -1 + EpoTime, data = y, random = ~1 |
    Subject)

Linear Hypotheses:
                          Estimate Std. Error z value Pr(>|z|)
EpoVsPlacebo.EpoWk1 == 0   0.06244    0.21614   0.289 0.772650
EpoVsPlacebo.EpoWk2 == 0   0.30550    0.21614   1.413 0.210026
EpoVsPlacebo.EpoWk3 == 0   0.45411    0.21614   2.101 0.057019 .
EpoVsPlacebo.EpoWk4 == 0   0.72911    0.21614   3.373 0.002970 **
EpoVsPlacebo.postWk1 == 0  0.84161    0.21614   3.894 0.000789 ***
EpoVsPlacebo.postWk2 == 0  0.27911    0.21614   1.291 0.224658
EpoVsPlacebo.postWk3 == 0  0.45967    0.21614   2.127 0.057019 .
EpoVsPlacebo.postWk4 == 0  0.65967    0.21614   3.052 0.006060 **
---
Signif. codes:  0 ‘***’ 0.001 ‘**’ 0.01 ‘*’ 0.05 ‘.’ 0.1 ‘ ’ 1
(Adjusted p values reported -- BH method)


#############################################################
```

4242

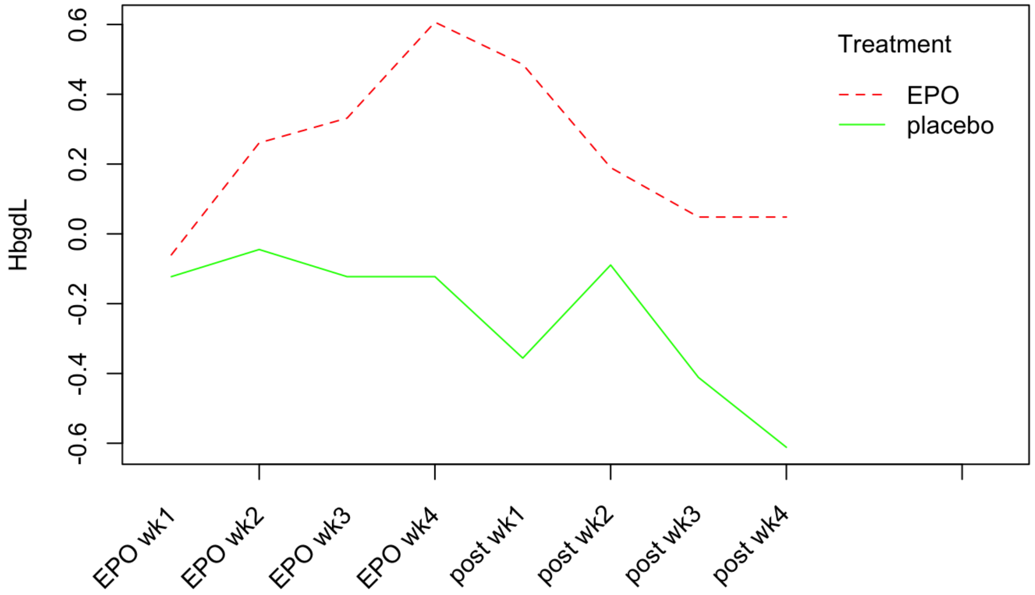


```
##############################################################
########### Repeated measures analysis results for  MCHpg ############
###############################################################
```

	numDF<br><int>	denDF<br><dbl>	F-value<br><chr>	p-value<br><chr>
(Intercept)	1	224	2.2165316	0.1379
Treatment	1	32	0.2185137	0.6433
time_point	7	224	1.3468076	0.2294
Treatment:time_point	7	224	1.1691200	0.3215

4 rows

```
############ Compare Epo with Palcebo at each time point using contrasts ###########
```

4343

```
################################################################

######################## Raw p-values ########################


     Simultaneous Tests for General Linear Hypotheses

Multiple Comparisons of Means: User-defined Contrasts


Fit: lme.formula(fixed = thisY ~ -1 + EpoTime, data = y, random = ~1 |
    Subject)

Linear Hypotheses:
                           Estimate Std. Error z value Pr(>|z|)
EpoVsPlacebo.EpoWk1 == 0  -0.036286   0.119087  -0.305    0.761
EpoVsPlacebo.EpoWk2 == 0   0.006825   0.119087   0.057    0.954
EpoVsPlacebo.EpoWk3 == 0   0.072603   0.119087   0.610    0.542
EpoVsPlacebo.EpoWk4 == 0   0.066381   0.119087   0.557    0.577
EpoVsPlacebo.postWk1 == 0 -0.142064   0.119087  -1.193    0.233
EpoVsPlacebo.postWk2 == 0  0.007714   0.119087   0.065    0.948
EpoVsPlacebo.postWk3 == 0 -0.143842   0.119087  -1.208    0.227
EpoVsPlacebo.postWk4 == 0 -0.154508   0.119087  -1.297    0.194
(Adjusted p values reported -- none method)

###########################################################


############## BH adjusted p-values ####################
Benjamini, Y., and Hochberg, Y. (1995). Controlling the false discovery rate: a practical and powerful approach t
o multiple testing. Journal of the Royal Statistical Society Series B, 57, 289–300.http://www.jstor.org/stable/23
46101.
###############################################################

     Simultaneous Tests for General Linear Hypotheses

Multiple Comparisons of Means: User-defined Contrasts


Fit: lme.formula(fixed = thisY ~ -1 + EpoTime, data = y, random = ~1 |
    Subject)

Linear Hypotheses:
                           Estimate Std. Error z value Pr(>|z|)
EpoVsPlacebo.EpoWk1 == 0  -0.036286   0.119087  -0.305    0.954
EpoVsPlacebo.EpoWk2 == 0   0.006825   0.119087   0.057    0.954
EpoVsPlacebo.EpoWk3 == 0   0.072603   0.119087   0.610    0.924
EpoVsPlacebo.EpoWk4 == 0   0.066381   0.119087   0.557    0.924
EpoVsPlacebo.postWk1 == 0 -0.142064   0.119087  -1.193    0.621
EpoVsPlacebo.postWk2 == 0  0.007714   0.119087   0.065    0.954
EpoVsPlacebo.postWk3 == 0 -0.143842   0.119087  -1.208    0.621
EpoVsPlacebo.postWk4 == 0 -0.154508   0.119087  -1.297    0.621
(Adjusted p values reported -- BH method)


##############################################################
```

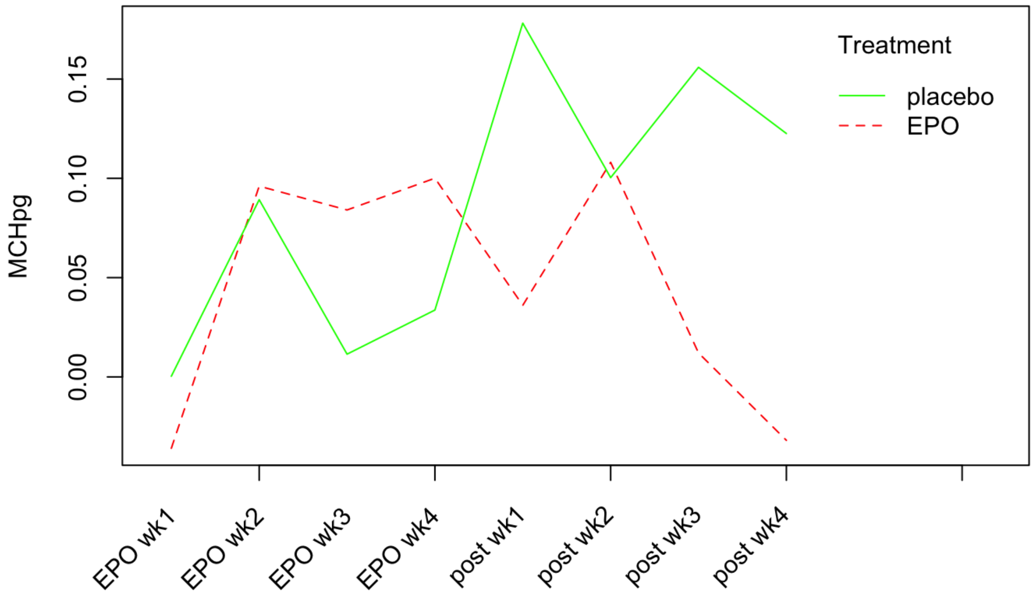

```
##############################################################

########### Repeated measures analysis results for  MCHCgdL ############

##############################################################
```

	numDF	denDF	F-value	p-value
	<int>	<dbl>	<chr>	<chr>
(Intercept)	1	224	13.550041	0.0003
Treatment	1	32	6.695523	0.0144
time_point	7	224	6.931815	<.0001
Treatment:time_point	7	224	3.145059	0.0034

4 rows

```
############ Compare Epo with Palcebo at each time point using contrasts ###########


##############################################################

######################## Raw p-values ########################


     Simultaneous Tests for General Linear Hypotheses

Multiple Comparisons of Means: User-defined Contrasts


Fit: lme.formula(fixed = thisY ~ -1 + EpoTime, data = y, random = ~1 |
    Subject)

Linear Hypotheses:
                          Estimate Std. Error z value Pr(>|z|)
EpoVsPlacebo.EpoWk1 == 0   -0.1742     0.1483  -1.175  0.24004
EpoVsPlacebo.EpoWk2 == 0   -0.3627     0.1483  -2.446  0.01446 *
EpoVsPlacebo.EpoWk3 == 0   -0.3756     0.1483  -2.533  0.01132 *
EpoVsPlacebo.EpoWk4 == 0   -0.4653     0.1483  -3.138  0.00170 **
EpoVsPlacebo.postWk1 == 0  -0.4644     0.1483  -3.132  0.00174 **
EpoVsPlacebo.postWk2 == 0  -0.0809     0.1483  -0.546  0.58540
EpoVsPlacebo.postWk3 == 0  -0.2671     0.1483  -1.801  0.07166 .
EpoVsPlacebo.postWk4 == 0   0.1387     0.1483   0.935  0.34978
---
Signif. codes:  0 ‘***’ 0.001 ‘**’ 0.01 ‘*’ 0.05 ‘.’ 0.1 ‘ ’ 1
(Adjusted p values reported -- none method)

##########################################################


############## BH adjusted p-values ####################
Benjamini, Y., and Hochberg, Y. (1995). Controlling the false discovery rate: a practical and powerful approach t
o multiple testing. Journal of the Royal Statistical Society Series B, 57, 289–300.http://www.jstor.org/stable/23
46101.
##############################################################

     Simultaneous Tests for General Linear Hypotheses

Multiple Comparisons of Means: User-defined Contrasts


Fit: lme.formula(fixed = thisY ~ -1 + EpoTime, data = y, random = ~1 |
    Subject)

Linear Hypotheses:
                          Estimate Std. Error z value Pr(>|z|)
EpoVsPlacebo.EpoWk1 == 0   -0.1742     0.1483  -1.175  0.32005
EpoVsPlacebo.EpoWk2 == 0   -0.3627     0.1483  -2.446  0.02892 *
EpoVsPlacebo.EpoWk3 == 0   -0.3756     0.1483  -2.533  0.02892 *
EpoVsPlacebo.EpoWk4 == 0   -0.4653     0.1483  -3.138  0.00695 **
EpoVsPlacebo.postWk1 == 0  -0.4644     0.1483  -3.132  0.00695 **
EpoVsPlacebo.postWk2 == 0  -0.0809     0.1483  -0.546  0.58540
EpoVsPlacebo.postWk3 == 0  -0.2671     0.1483  -1.801  0.11466
EpoVsPlacebo.postWk4 == 0   0.1387     0.1483   0.935  0.39975
---
Signif. codes:  0 ‘***’ 0.001 ‘**’ 0.01 ‘*’ 0.05 ‘.’ 0.1 ‘ ’ 1
(Adjusted p values reported -- BH method)


#############################################################
```

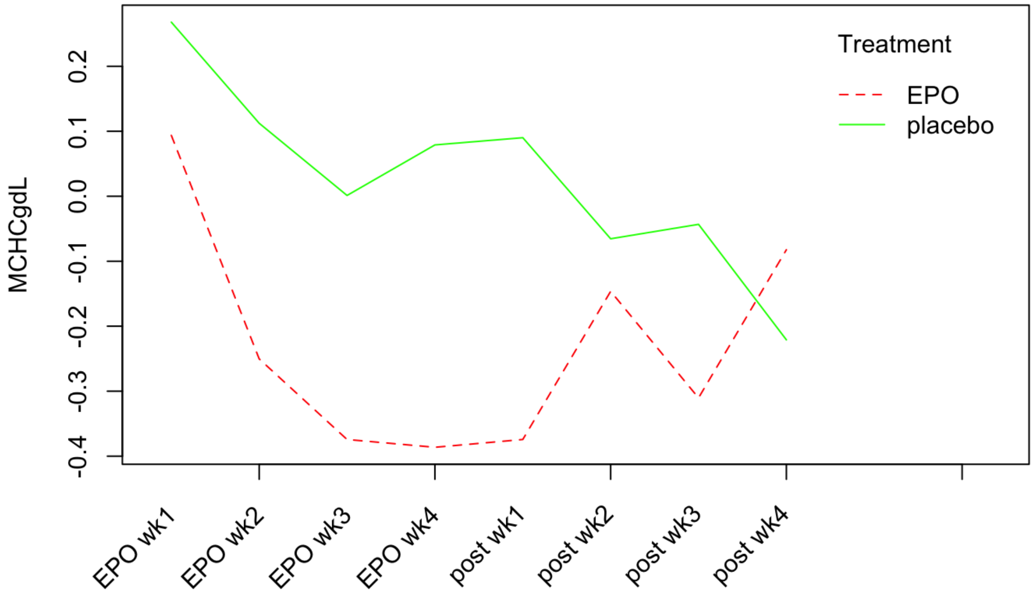


```
################################################################
########### Repeated measures analysis results for  MCVfl ############
#################################################################
```

	numDF <int>	denDF <dbl>	F-value <chr>	p-value <chr>
(Intercept)	1	224	14.637513	0.0002
Treatment	1	32	2.663040	0.1125
time_point	7	224	12.843467	<.0001
Treatment:time_point	7	224	6.180984	<.0001

4 rows

4646

```
########### Compare Epo with Palcebo at each time point using contrasts ##########


################################################################

######################## Raw p-values ########################


     Simultaneous Tests for General Linear Hypotheses

Multiple Comparisons of Means: User-defined Contrasts


Fit: lme.formula(fixed = thisY ~ -1 + EpoTime, data = y, random = ~1 |
    Subject)

Linear Hypotheses:
                          Estimate Std. Error z value Pr(>|z|)
EpoVsPlacebo.EpoWk1 == 0    0.4076     0.4306   0.947  0.34384
EpoVsPlacebo.EpoWk2 == 0    0.9680     0.4306   2.248  0.02456 *
EpoVsPlacebo.EpoWk3 == 0    1.1965     0.4306   2.779  0.00546 **
EpoVsPlacebo.EpoWk4 == 0    1.3934     0.4306   3.236  0.00121 **
EpoVsPlacebo.postWk1 == 0   0.8076     0.4306   1.876  0.06071 .
EpoVsPlacebo.postWk2 == 0   0.1707     0.4306   0.396  0.69178
EpoVsPlacebo.postWk3 == 0   0.3080     0.4306   0.715  0.47437
EpoVsPlacebo.postWk4 == 0  -0.8177     0.4306  -1.899  0.05755 .
---
Signif. codes:  0 ‘***’ 0.001 ‘**’ 0.01 ‘*’ 0.05 ‘.’ 0.1 ‘ ’ 1
(Adjusted p values reported -- none method)

###########################################################


############## BH adjusted p-values #####################
Benjamini, Y., and Hochberg, Y. (1995). Controlling the false discovery rate: a practical and powerful approach t
o multiple testing. Journal of the Royal Statistical Society Series B, 57, 289–300.http://www.jstor.org/stable/23
46101.
###############################################################

     Simultaneous Tests for General Linear Hypotheses

Multiple Comparisons of Means: User-defined Contrasts


Fit: lme.formula(fixed = thisY ~ -1 + EpoTime, data = y, random = ~1 |
    Subject)

Linear Hypotheses:
                          Estimate Std. Error z value Pr(>|z|)
EpoVsPlacebo.EpoWk1 == 0    0.4076     0.4306   0.947   0.4585
EpoVsPlacebo.EpoWk2 == 0    0.9680     0.4306   2.248   0.0655 .
EpoVsPlacebo.EpoWk3 == 0    1.1965     0.4306   2.779   0.0218 *
EpoVsPlacebo.EpoWk4 == 0    1.3934     0.4306   3.236   0.0097 **
EpoVsPlacebo.postWk1 == 0   0.8076     0.4306   1.876   0.0971 .
EpoVsPlacebo.postWk2 == 0   0.1707     0.4306   0.396   0.6918
EpoVsPlacebo.postWk3 == 0   0.3080     0.4306   0.715   0.5421
EpoVsPlacebo.postWk4 == 0  -0.8177     0.4306  -1.899   0.0971 .
---
Signif. codes:  0 ‘***’ 0.001 ‘**’ 0.01 ‘*’ 0.05 ‘.’ 0.1 ‘ ’ 1
(Adjusted p values reported -- BH method)


##############################################################
```

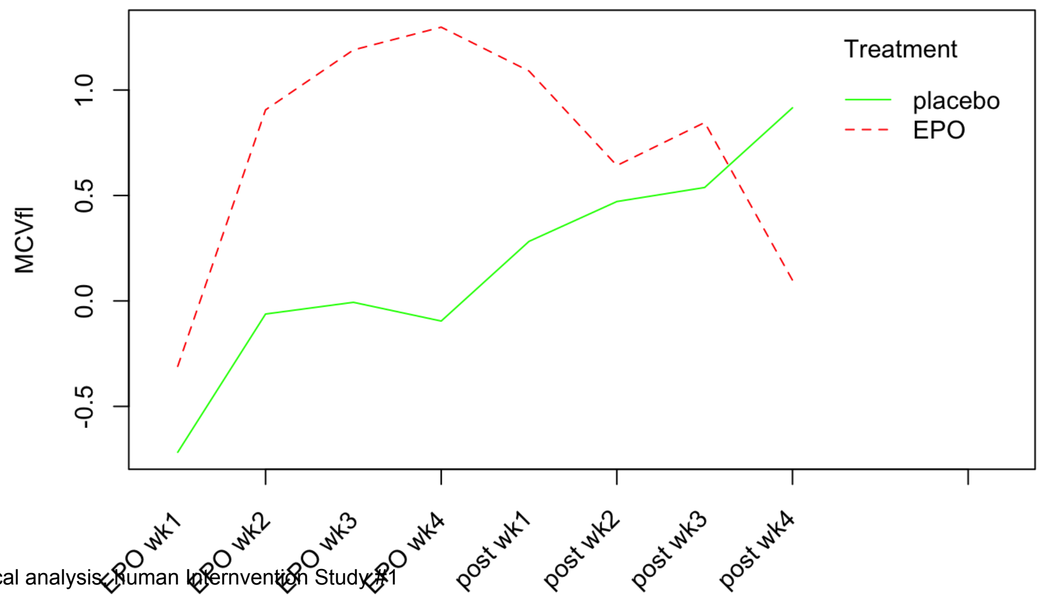

4747
```
##############################################################
########### Repeated measures analysis results for  RDWCV ############
##############################################################
```

	numDF	denDF	F-value	p-value
	<int>	<dbl>	<chr>	<chr>
(Intercept)	1	224	35.71804	<.0001
Treatment	1	32	6.79828	0.0137
time_point	7	224	33.86688	<.0001
Treatment:time_point	7	224	15.77146	<.0001

4 rows

```
############ Compare Epo with Palcebo at each time point using contrasts ###########


##############################################################
######################## Raw p-values ########################


     Simultaneous Tests for General Linear Hypotheses

Multiple Comparisons of Means: User-defined Contrasts


Fit: lme.formula(fixed = thisY ~ -1 + EpoTime, data = y, random = ~1 | 
    Subject)

Linear Hypotheses:
                          Estimate Std. Error z value Pr(>|z|)    
EpoVsPlacebo.EpoWk1 == 0   0.16689    0.11319   1.474   0.1404    
EpoVsPlacebo.EpoWk2 == 0   0.51000    0.11319   4.506 6.61e-06 ***
EpoVsPlacebo.EpoWk3 == 0   0.50422    0.11319   4.455 8.40e-06 ***
EpoVsPlacebo.EpoWk4 == 0   0.61533    0.11319   5.436 5.44e-08 ***
EpoVsPlacebo.postWk1 == 0  0.26689    0.11319   2.358   0.0184 *  
EpoVsPlacebo.postWk2 == 0  0.04156    0.11319   0.367   0.7135    
EpoVsPlacebo.postWk3 == 0 -0.03844    0.11319  -0.340   0.7341    
EpoVsPlacebo.postWk4 == 0 -0.17400    0.11319  -1.537   0.1242    
---
Signif. codes:  0 ‘***’ 0.001 ‘**’ 0.01 ‘*’ 0.05 ‘.’ 0.1 ‘ ’ 1
(Adjusted p values reported -- none method)

##########################################################


############## BH adjusted p-values ####################
Benjamini, Y., and Hochberg, Y. (1995). Controlling the false discovery rate: a practical and powerful approach t
o multiple testing. Journal of the Royal Statistical Society Series B, 57, 289–300.http://www.jstor.org/stable/23
46101.
##############################################################

     Simultaneous Tests for General Linear Hypotheses

Multiple Comparisons of Means: User-defined Contrasts


Fit: lme.formula(fixed = thisY ~ -1 + EpoTime, data = y, random = ~1 | 
    Subject)

Linear Hypotheses:
                          Estimate Std. Error z value Pr(>|z|)    
EpoVsPlacebo.EpoWk1 == 0   0.16689    0.11319   1.474   0.1871    
EpoVsPlacebo.EpoWk2 == 0   0.51000    0.11319   4.506 2.24e-05 ***
EpoVsPlacebo.EpoWk3 == 0   0.50422    0.11319   4.455 2.24e-05 ***
EpoVsPlacebo.EpoWk4 == 0   0.61533    0.11319   5.436 4.35e-07 ***
EpoVsPlacebo.postWk1 == 0  0.26689    0.11319   2.358   0.0368 *  
EpoVsPlacebo.postWk2 == 0  0.04156    0.11319   0.367   0.7341    
EpoVsPlacebo.postWk3 == 0 -0.03844    0.11319  -0.340   0.7341    
EpoVsPlacebo.postWk4 == 0 -0.17400    0.11319  -1.537   0.1871    
---
Signif. codes:  0 ‘***’ 0.001 ‘**’ 0.01 ‘*’ 0.05 ‘.’ 0.1 ‘ ’ 1
(Adjusted p values reported -- BH method)


#############################################################
```

4848

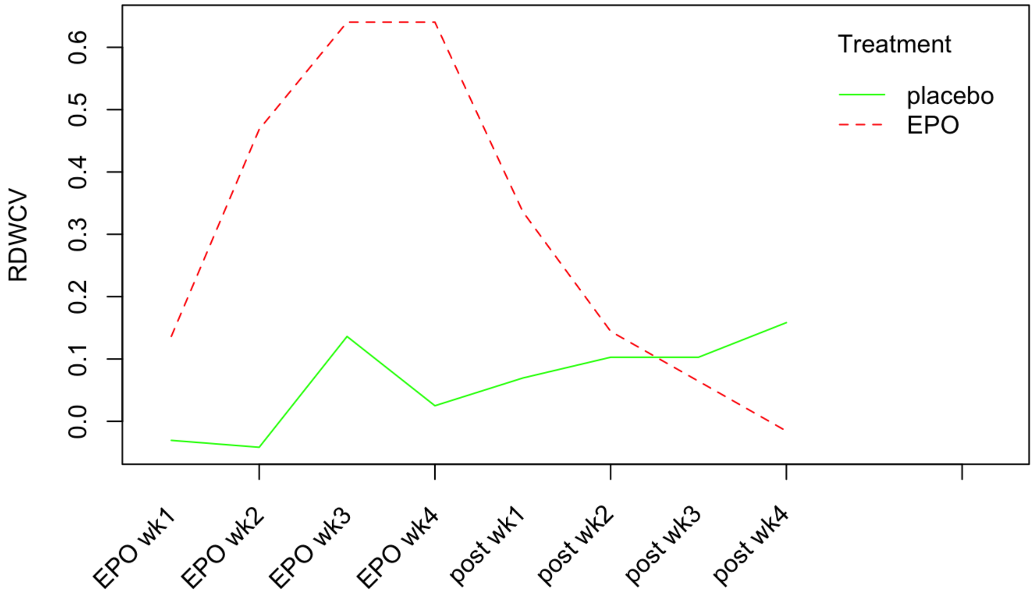


```
##############################################################
########### Repeated measures analysis results for  RDWSDfl ############
###############################################################
```

	numDF <int>	denDF <dbl>	F-value <chr>	p-value <chr>
(Intercept)	1	224	39.46842	<.0001
Treatment	1	32	8.95499	0.0053
time_point	7	224	27.83700	<.0001
Treatment:time_point	7	224	12.98759	<.0001

4 rows

4949

```
########### Compare Epo with Palcebo at each time point using contrasts ###########


################################################################

####################### Raw p-values #######################


     Simultaneous Tests for General Linear Hypotheses

Multiple Comparisons of Means: User-defined Contrasts


Fit: lme.formula(fixed = thisY ~ -1 + EpoTime, data = y, random = ~1 |
    Subject)

Linear Hypotheses:
                          Estimate Std. Error z value Pr(>|z|)
EpoVsPlacebo.EpoWk1 == 0    0.7604     0.4879   1.558  0.11916
EpoVsPlacebo.EpoWk2 == 0    1.9510     0.4879   3.999 6.37e-05 ***
EpoVsPlacebo.EpoWk3 == 0    2.2804     0.4879   4.673 2.96e-06 ***
EpoVsPlacebo.EpoWk4 == 0    2.7235     0.4879   5.582 2.38e-08 ***
EpoVsPlacebo.postWk1 == 0   1.4933     0.4879   3.060  0.00221 **
EpoVsPlacebo.postWk2 == 0   0.4115     0.4879   0.843  0.39906
EpoVsPlacebo.postWk3 == 0   0.2430     0.4879   0.498  0.61842
EpoVsPlacebo.postWk4 == 0  -0.8223     0.4879  -1.685  0.09194 .
---
Signif. codes:  0 ‘***’ 0.001 ‘**’ 0.01 ‘*’ 0.05 ‘.’ 0.1 ‘ ’ 1
(Adjusted p values reported -- none method)

##########################################################


############## BH adjusted p-values ####################
Benjamini, Y., and Hochberg, Y. (1995). Controlling the false discovery rate: a practical and powerful approach t
o multiple testing. Journal of the Royal Statistical Society Series B, 57, 289–300.http://www.jstor.org/stable/23
46101.
##############################################################

     Simultaneous Tests for General Linear Hypotheses

Multiple Comparisons of Means: User-defined Contrasts


Fit: lme.formula(fixed = thisY ~ -1 + EpoTime, data = y, random = ~1 |
    Subject)

Linear Hypotheses:
                          Estimate Std. Error z value Pr(>|z|)
EpoVsPlacebo.EpoWk1 == 0    0.7604     0.4879   1.558  0.15888
EpoVsPlacebo.EpoWk2 == 0    1.9510     0.4879   3.999  0.00017 ***
EpoVsPlacebo.EpoWk3 == 0    2.2804     0.4879   4.673 1.18e-05 ***
EpoVsPlacebo.EpoWk4 == 0    2.7235     0.4879   5.582 1.91e-07 ***
EpoVsPlacebo.postWk1 == 0   1.4933     0.4879   3.060  0.00442 **
EpoVsPlacebo.postWk2 == 0   0.4115     0.4879   0.843  0.45607
EpoVsPlacebo.postWk3 == 0   0.2430     0.4879   0.498  0.61842
EpoVsPlacebo.postWk4 == 0  -0.8223     0.4879  -1.685  0.14711
---
Signif. codes:  0 ‘***’ 0.001 ‘**’ 0.01 ‘*’ 0.05 ‘.’ 0.1 ‘ ’ 1
(Adjusted p values reported -- BH method)


#############################################################
```

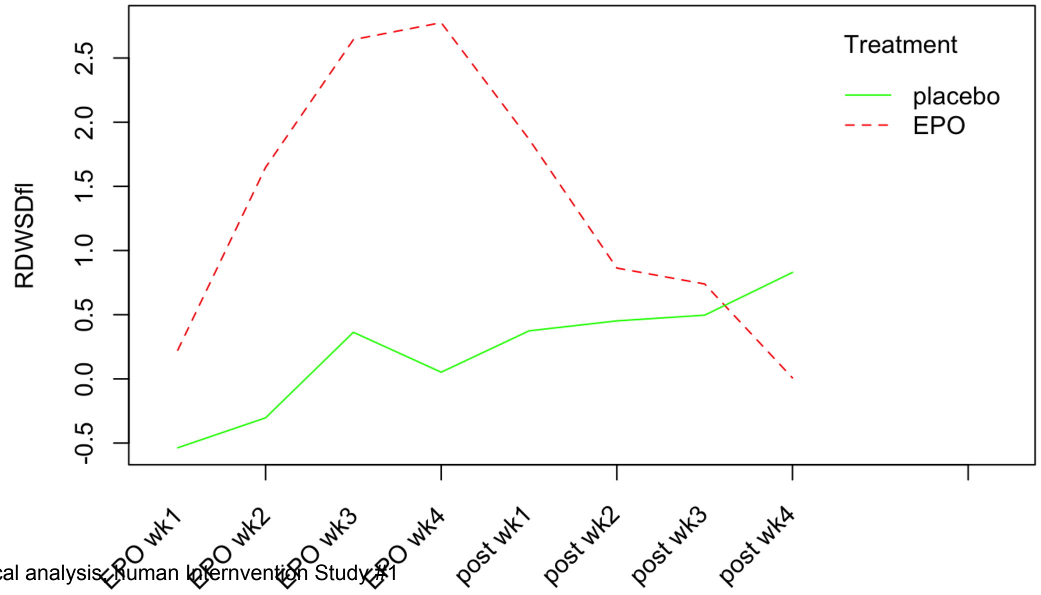

```
##############################################################

########### Repeated measures analysis results for  Retics ############

###############################################################
```

	numDF	denDF	F-value	p-value
	<int>	<dbl>	<chr>	<chr>
(Intercept)	1	224	12.82991	0.0004
Treatment	1	32	0.32422	0.5731
time_point	7	224	49.77240	<.0001
Treatment:time_point	7	224	16.77994	<.0001

4 rows

```
############ Compare Epo with Palcebo at each time point using contrasts ###########


###############################################################

######################## Raw p-values #########################


     Simultaneous Tests for General Linear Hypotheses

Multiple Comparisons of Means: User-defined Contrasts


Fit: lme.formula(fixed = thisY ~ -1 + EpoTime, data = y, random = ~1 |
    Subject)

Linear Hypotheses:
                          Estimate Std. Error z value Pr(>|z|)
EpoVsPlacebo.EpoWk1 == 0   0.13632    0.09377   1.454 0.145997
EpoVsPlacebo.EpoWk2 == 0   0.55090    0.09377   5.875 4.23e-09 ***
EpoVsPlacebo.EpoWk3 == 0   0.35037    0.09377   3.737 0.000187 ***
EpoVsPlacebo.EpoWk4 == 0   0.23574    0.09377   2.514 0.011933 *
EpoVsPlacebo.postWk1 == 0 -0.33403    0.09377  -3.562 0.000368 ***
EpoVsPlacebo.postWk2 == 0 -0.37479    0.09377  -3.997 6.42e-05 ***
EpoVsPlacebo.postWk3 == 0 -0.17168    0.09377  -1.831 0.067120 .
EpoVsPlacebo.postWk4 == 0 -0.14937    0.09377  -1.593 0.111176
---
Signif. codes:  0 ‘***’ 0.001 ‘**’ 0.01 ‘*’ 0.05 ‘.’ 0.1 ‘ ’ 1
(Adjusted p values reported -- none method)

##########################################################


############## BH adjusted p-values ####################
Benjamini, Y., and Hochberg, Y. (1995). Controlling the false discovery rate: a practical and powerful approach t
o multiple testing. Journal of the Royal Statistical Society Series B, 57, 289–300.http://www.jstor.org/stable/23
46101.
##############################################################

     Simultaneous Tests for General Linear Hypotheses

Multiple Comparisons of Means: User-defined Contrasts


Fit: lme.formula(fixed = thisY ~ -1 + EpoTime, data = y, random = ~1 |
    Subject)

Linear Hypotheses:
                          Estimate Std. Error z value Pr(>|z|)
EpoVsPlacebo.EpoWk1 == 0   0.13632    0.09377   1.454 0.145997
EpoVsPlacebo.EpoWk2 == 0   0.55090    0.09377   5.875 3.38e-08 ***
EpoVsPlacebo.EpoWk3 == 0   0.35037    0.09377   3.737 0.000498 ***
EpoVsPlacebo.EpoWk4 == 0   0.23574    0.09377   2.514 0.019093 *
EpoVsPlacebo.postWk1 == 0 -0.33403    0.09377  -3.562 0.000735 ***
EpoVsPlacebo.postWk2 == 0 -0.37479    0.09377  -3.997 0.000257 ***
EpoVsPlacebo.postWk3 == 0 -0.17168    0.09377  -1.831 0.089494 .
EpoVsPlacebo.postWk4 == 0 -0.14937    0.09377  -1.593 0.127058
---
Signif. codes:  0 ‘***’ 0.001 ‘**’ 0.01 ‘*’ 0.05 ‘.’ 0.1 ‘ ’ 1
(Adjusted p values reported -- BH method)


#############################################################
```

5151

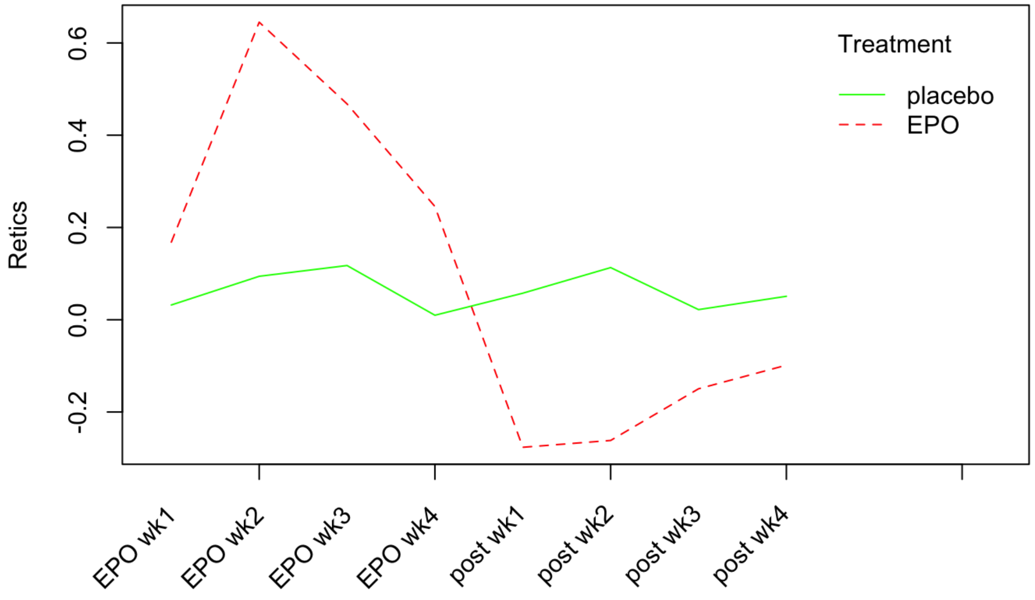


```
##############################################################

########### Repeated measures analysis results for  Retics109L ############

###############################################################
```

	numDF <int>	denDF <dbl>	F-value <chr>	p-value <chr>
(Intercept)	1	224	14.67996	0.0002
Treatment	1	32	1.33941	0.2557
time_point	7	224	52.94137	<.0001
Treatment:time_point	7	224	16.77202	<.0001

4 rows

```
########### Compare Epo with Palcebo at each time point using contrasts ##########


###############################################################

####################### Raw p-values #######################


     Simultaneous Tests for General Linear Hypotheses

Multiple Comparisons of Means: User-defined Contrasts


Fit: lme.formula(fixed = thisY ~ -1 + EpoTime, data = y, random = ~1 |
    Subject)

Linear Hypotheses:
                          Estimate Std. Error z value Pr(>|z|)
EpoVsPlacebo.EpoWk1 == 0     6.681      4.377   1.527 0.126865
EpoVsPlacebo.EpoWk2 == 0    26.618      4.377   6.082 1.19e-09 ***
EpoVsPlacebo.EpoWk3 == 0    18.323      4.377   4.187 2.83e-05 ***
EpoVsPlacebo.EpoWk4 == 0    13.460      4.377   3.075 0.002102 **
EpoVsPlacebo.postWk1 == 0  -13.196      4.377  -3.015 0.002569 **
EpoVsPlacebo.postWk2 == 0  -16.797      4.377  -3.838 0.000124 ***
EpoVsPlacebo.postWk3 == 0   -6.748      4.377  -1.542 0.123122
EpoVsPlacebo.postWk4 == 0   -5.203      4.377  -1.189 0.234516
---
Signif. codes:  0 '***' 0.001 '**' 0.01 '*' 0.05 '.' 0.1 ' ' 1
(Adjusted p values reported -- none method)

##########################################################


############## BH adjusted p-values ####################
Benjamini, Y., and Hochberg, Y. (1995). Controlling the false discovery rate: a practical and powerful approach t
o multiple testing. Journal of the Royal Statistical Society Series B, 57, 289–300.http://www.jstor.org/stable/23
46101.
##############################################################

     Simultaneous Tests for General Linear Hypotheses

Multiple Comparisons of Means: User-defined Contrasts


Fit: lme.formula(fixed = thisY ~ -1 + EpoTime, data = y, random = ~1 |
    Subject)

Linear Hypotheses:
                          Estimate Std. Error z value Pr(>|z|)
EpoVsPlacebo.EpoWk1 == 0     6.681      4.377   1.527 0.144988
EpoVsPlacebo.EpoWk2 == 0    26.618      4.377   6.082  9.5e-09 ***
EpoVsPlacebo.EpoWk3 == 0    18.323      4.377   4.187 0.000113 ***
EpoVsPlacebo.EpoWk4 == 0    13.460      4.377   3.075 0.004110 **
EpoVsPlacebo.postWk1 == 0  -13.196      4.377  -3.015 0.004110 **
EpoVsPlacebo.postWk2 == 0  -16.797      4.377  -3.838 0.000331 ***
EpoVsPlacebo.postWk3 == 0   -6.748      4.377  -1.542 0.144988
EpoVsPlacebo.postWk4 == 0   -5.203      4.377  -1.189 0.234516
---
Signif. codes:  0 '***' 0.001 '**' 0.01 '*' 0.05 '.' 0.1 ' ' 1
(Adjusted p values reported -- BH method)
```

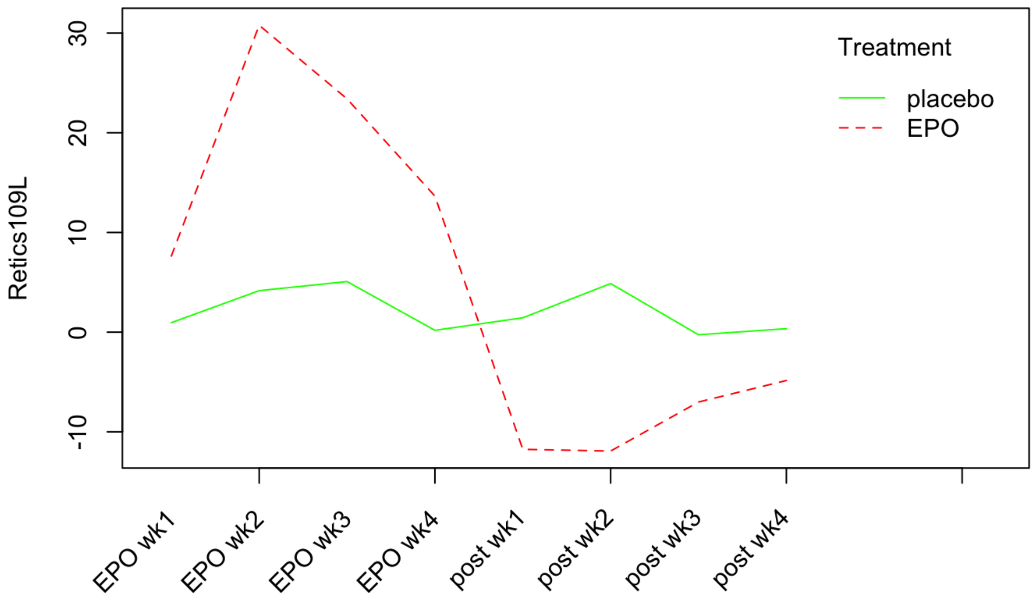

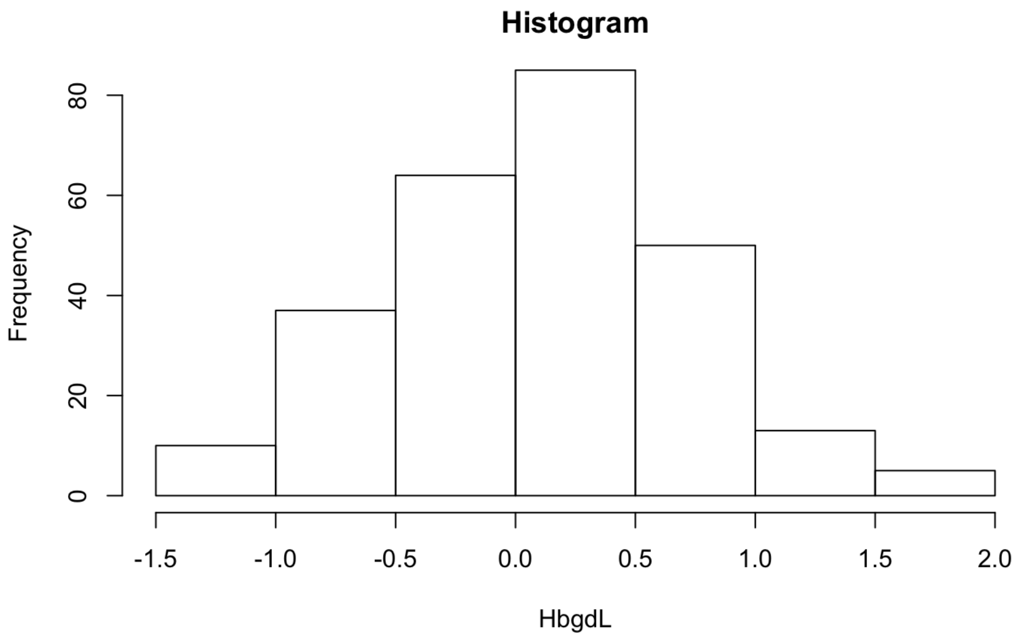
Histogram
Frequency
0
20
40
60
80
-1.5
-1.0
-0.5
0.0
0.5
1.0
1.5
2.0
HbgdL

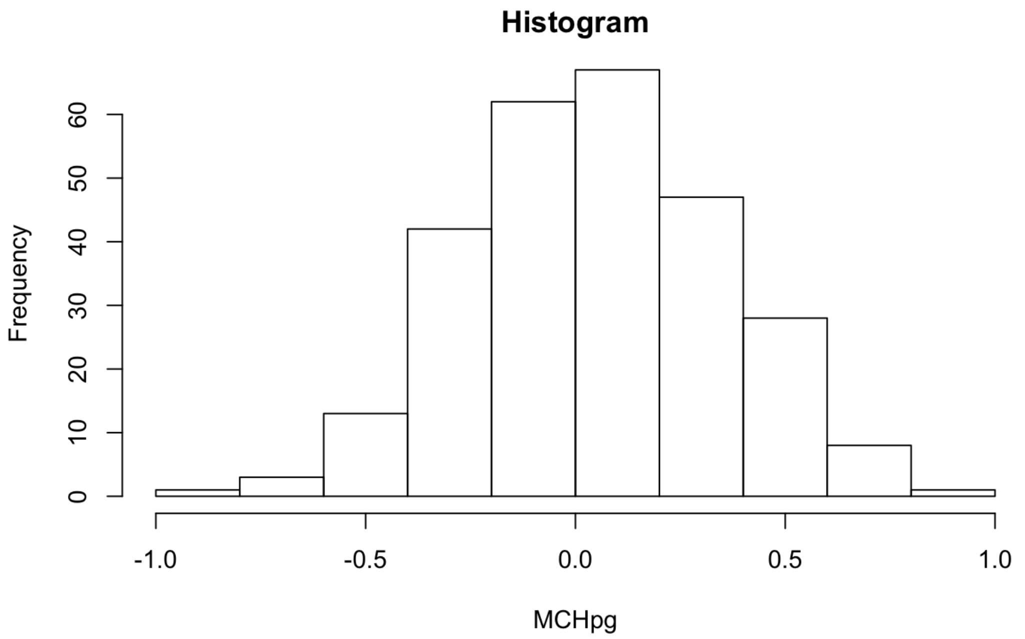
Histogram
Frequency
0
10
20
30
40
50
60
-1.0
-0.5
0.0
0.5
1.0
MCHpg

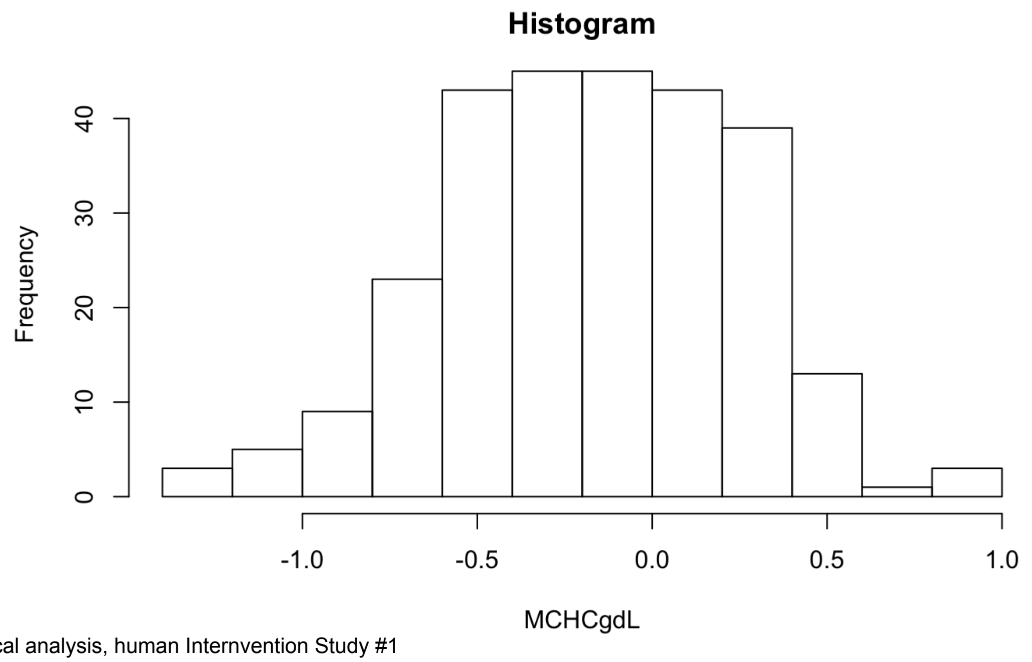
Histogram
Frequency
0
10
20
30
40
-1.0
-0.5
0.0
0.5
1.0
MCHCgdL

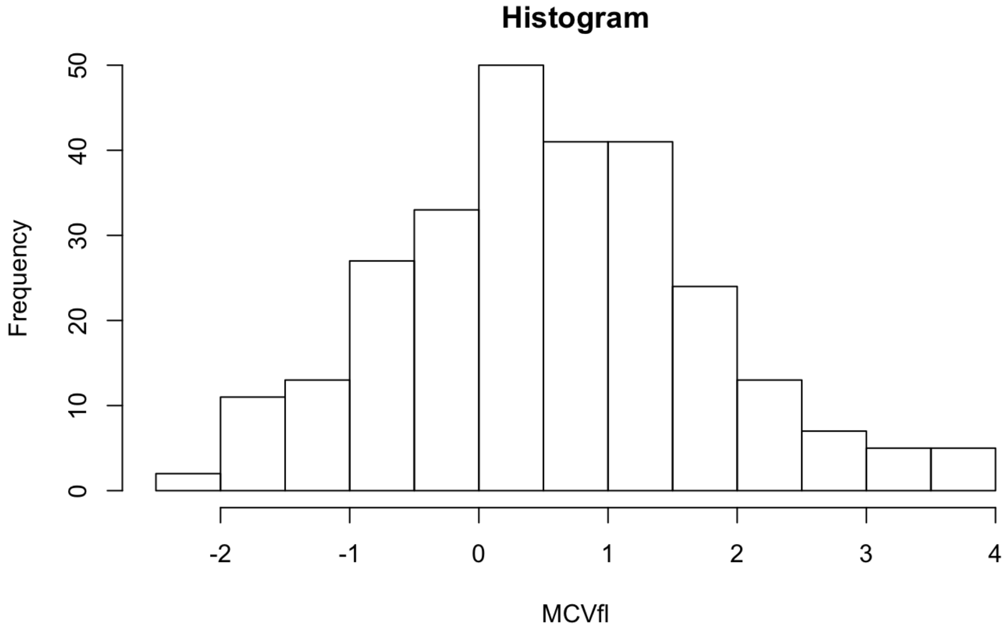
Histogram
Frequency
0
10
20
30
40
50
-2
-1
0
1
2
3
4
MCVfl

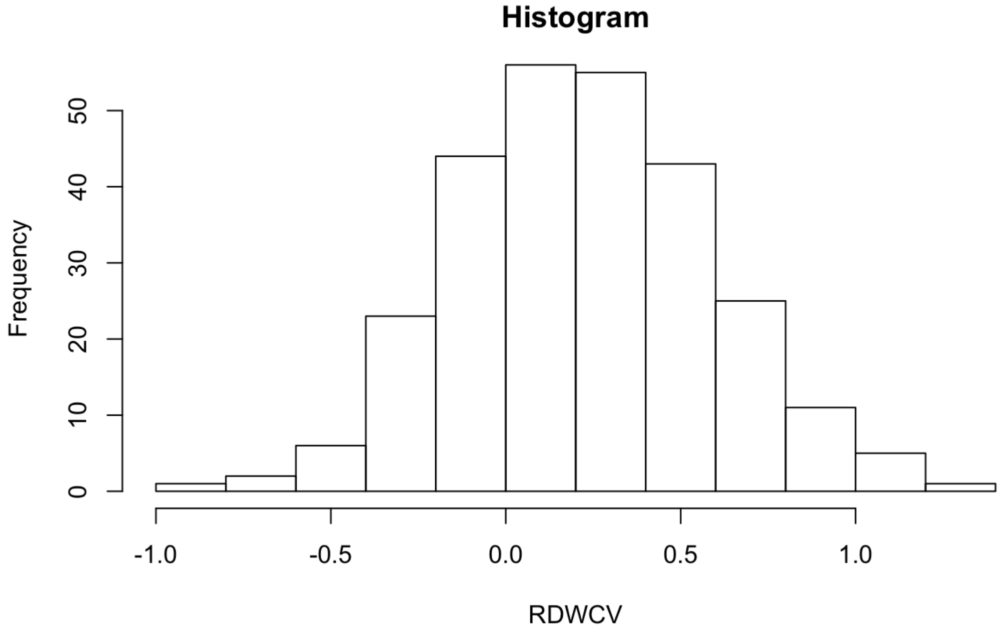
Histogram
Frequency
0
10
20
30
40
50
-1.0
-0.5
0.0
0.5
1.0
RDWCV

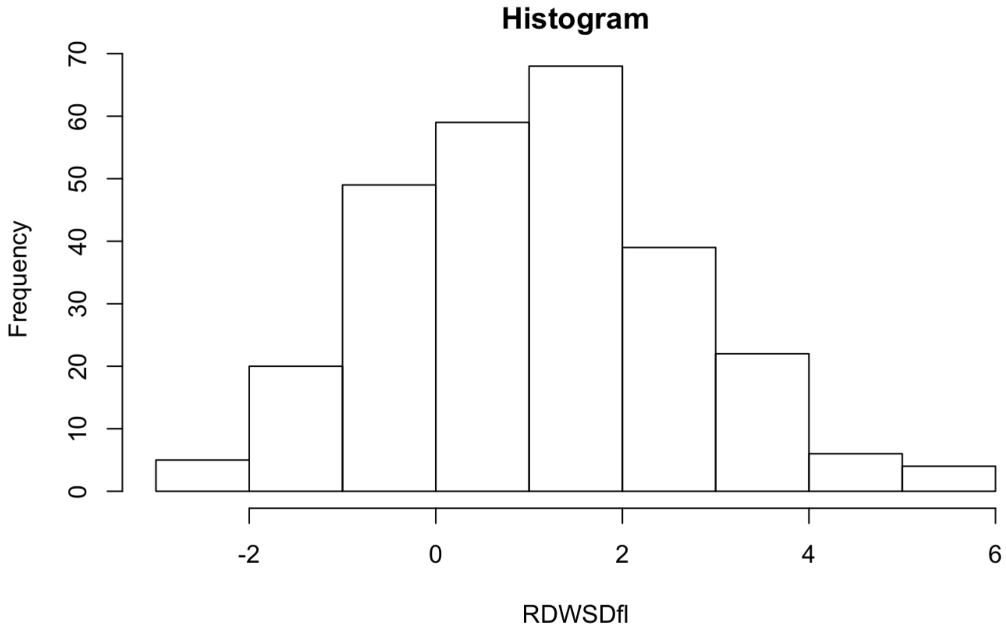
Histogram
Frequency
0
10
20
30
40
50
60
70
-2
0
2
4
6
RDWSDfl

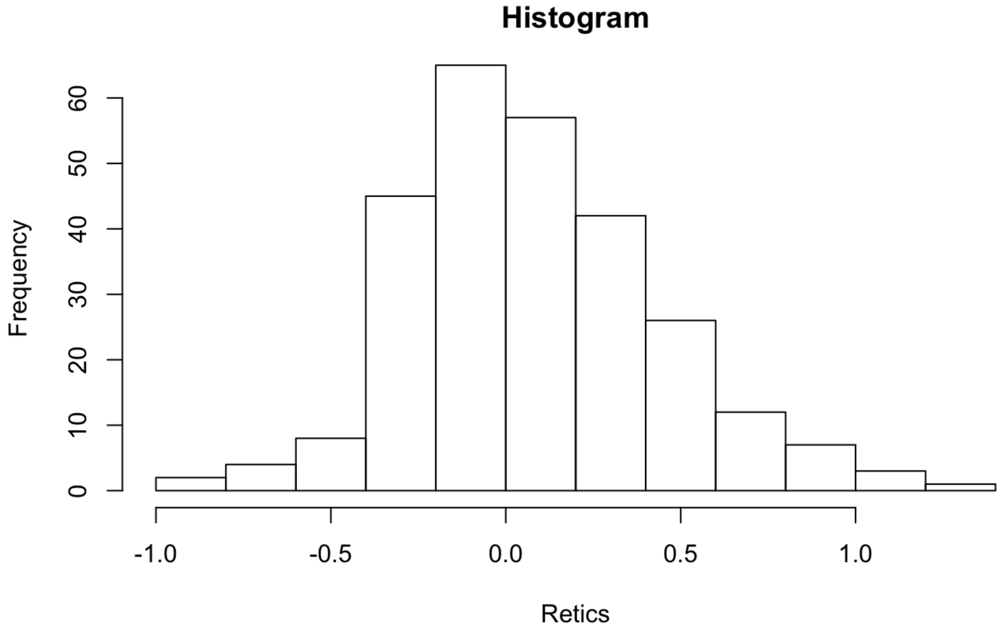
Histogram
Frequency
0
10
20
30
40
50
60
-1.0
-0.5
0.0
0.5
1.0
Retics

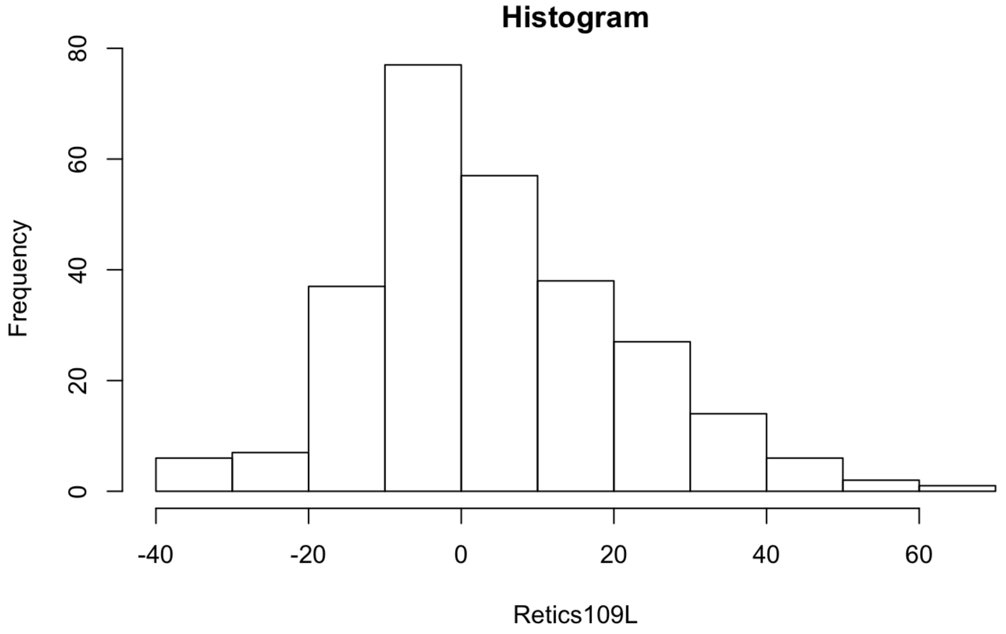
Histogram
Frequency
0
20
40
60
80
-40
-20
0
20
40
60
Retics109L

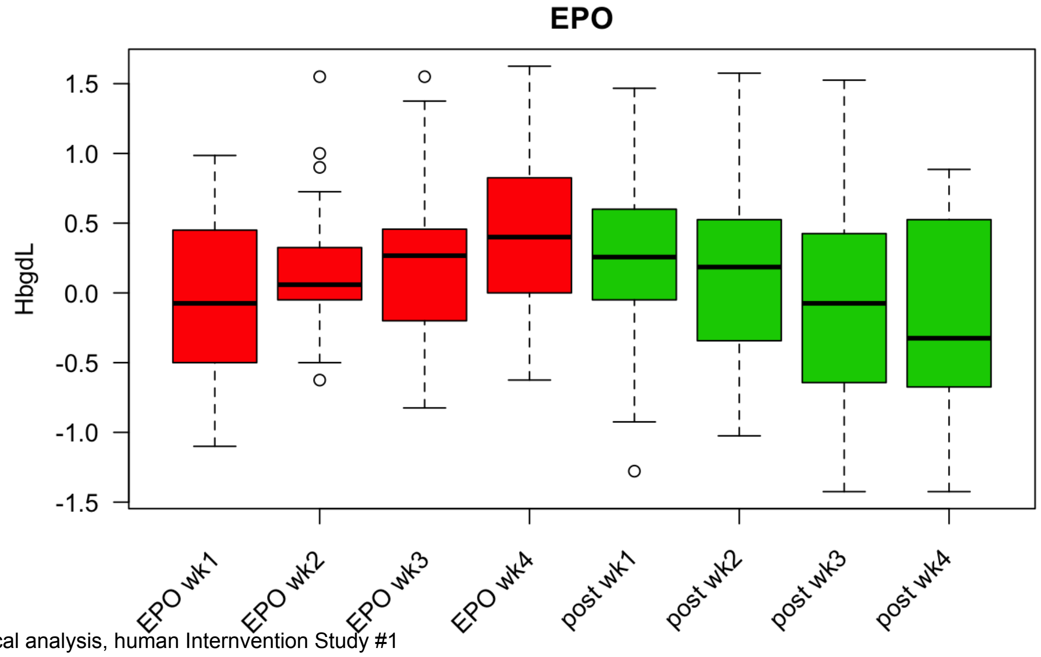
EPO
HbgdL
1.5
1.0
0.5
0.0
-0.5
-1.0
-1.5
EPO wk1
EPO wk2
EPO wk3
EPO wk4
post wk1
post wk2
post wk3
post wk4

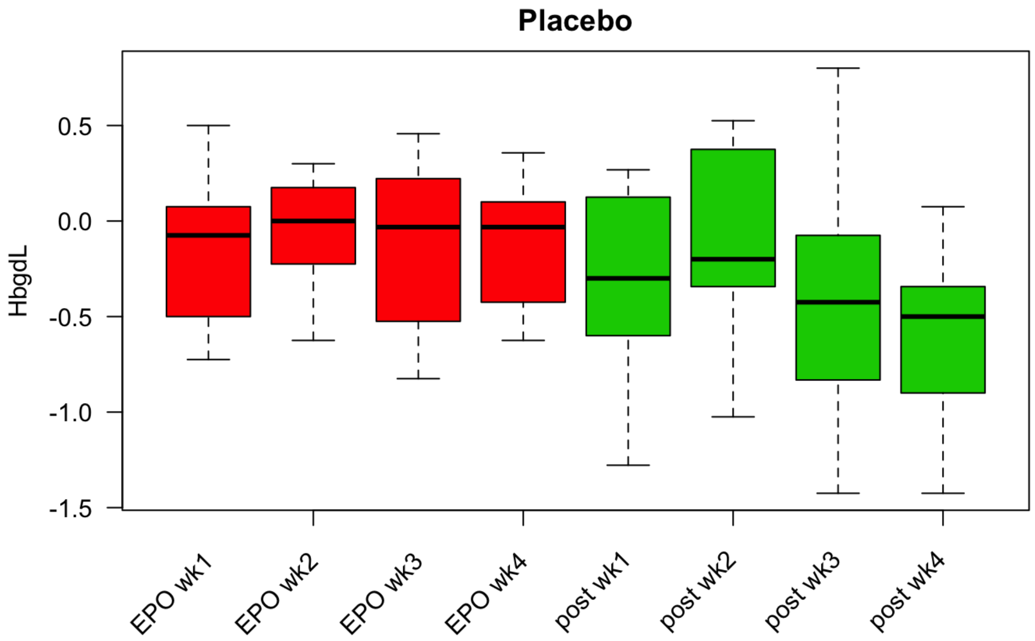
Placebo
HbgdL
0.5
0.0
-0.5
-1.0
-1.5
EPO wk1
EPO wk2
EPO wk3
EPO wk4
post wk1
post wk2
post wk3
post wk4

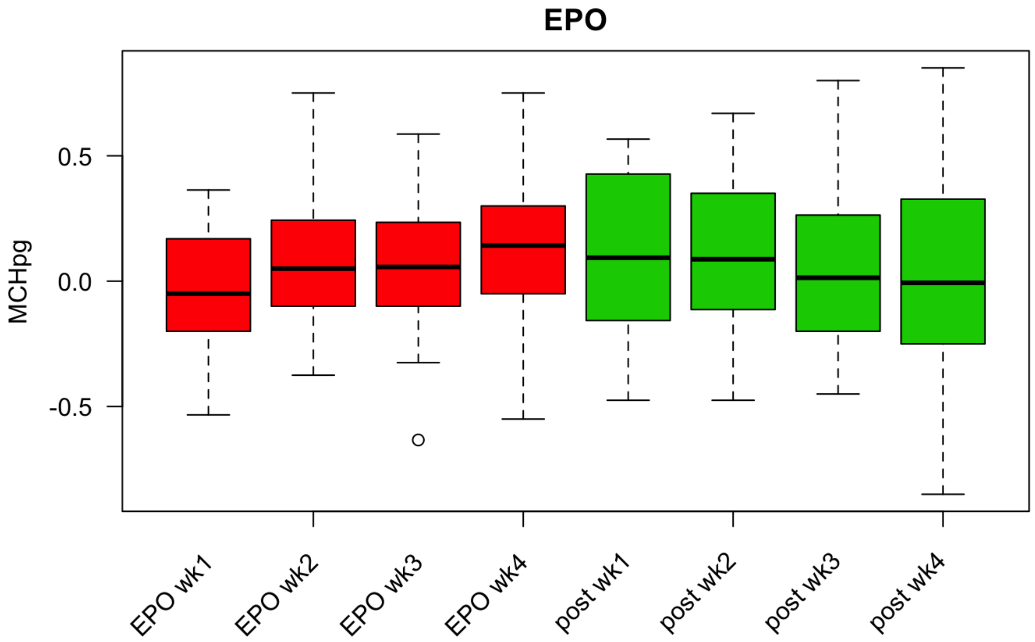
EPO
MCHpg
0.5
0.0
-0.5
EPO wk1
EPO wk2
EPO wk3
EPO wk4
post wk1
post wk2
post wk3
post wk4

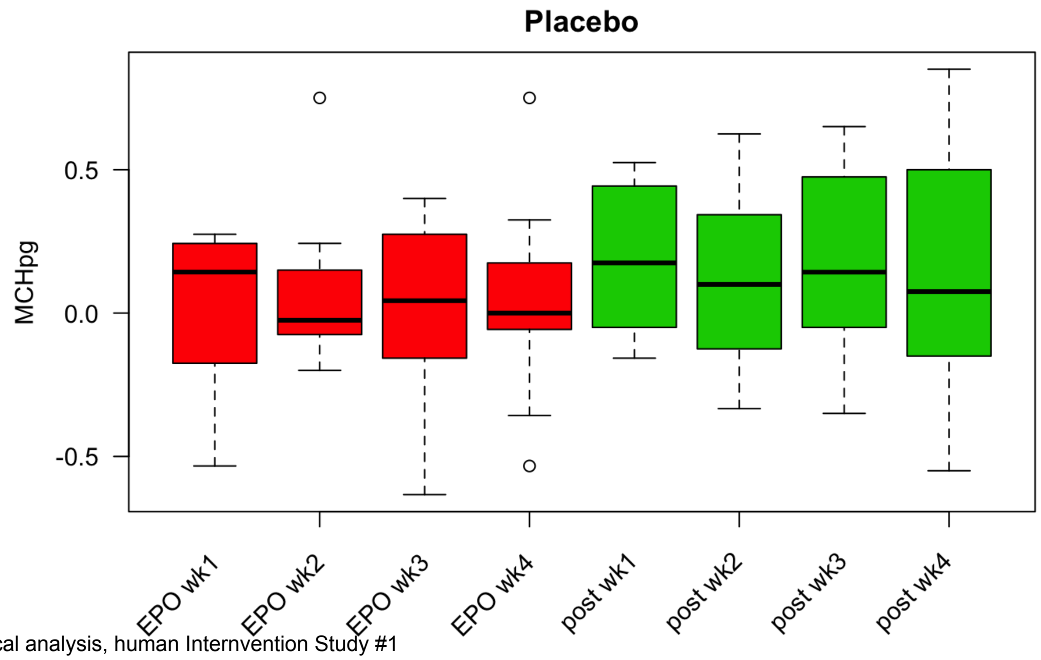
Placebo
MCHpg
0.5
0.0
-0.5
EPO wk1
EPO wk2
EPO wk3
EPO wk4
post wk1
post wk2
post wk3
post wk4

5757

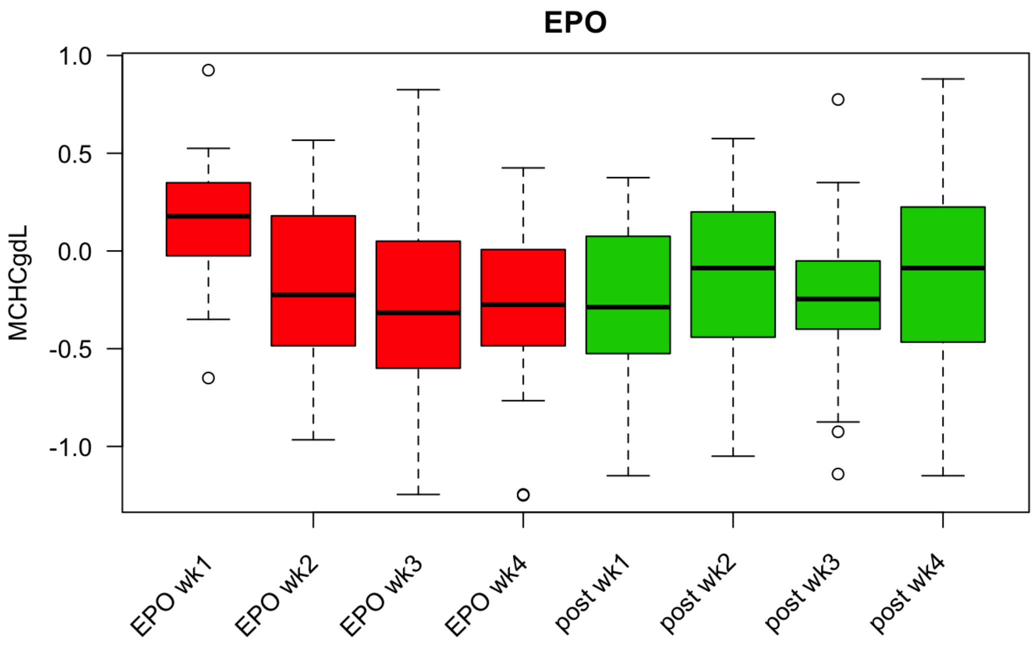
EPO
MCHCgdL
1.0
0.5
0.0
-0.5
-1.0
EPO wk1
EPO wk2
EPO wk3
EPO wk4
post wk1
post wk2
post wk3
post wk4

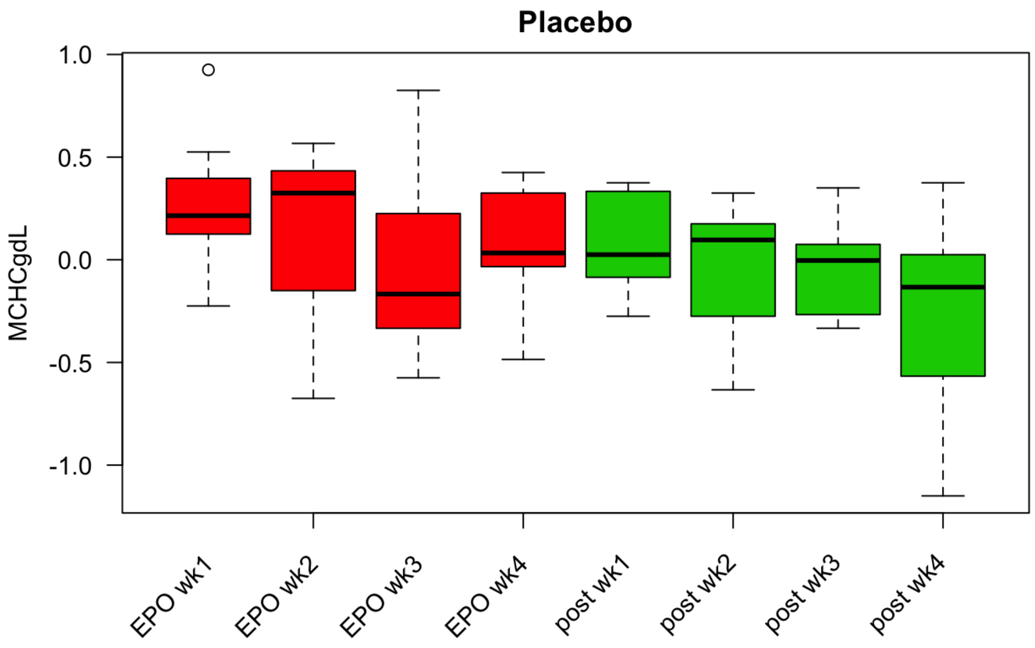
Placebo
MCHCgdL
1.0
0.5
0.0
-0.5
-1.0
EPO wk1
EPO wk2
EPO wk3
EPO wk4
post wk1
post wk2
post wk3
post wk4

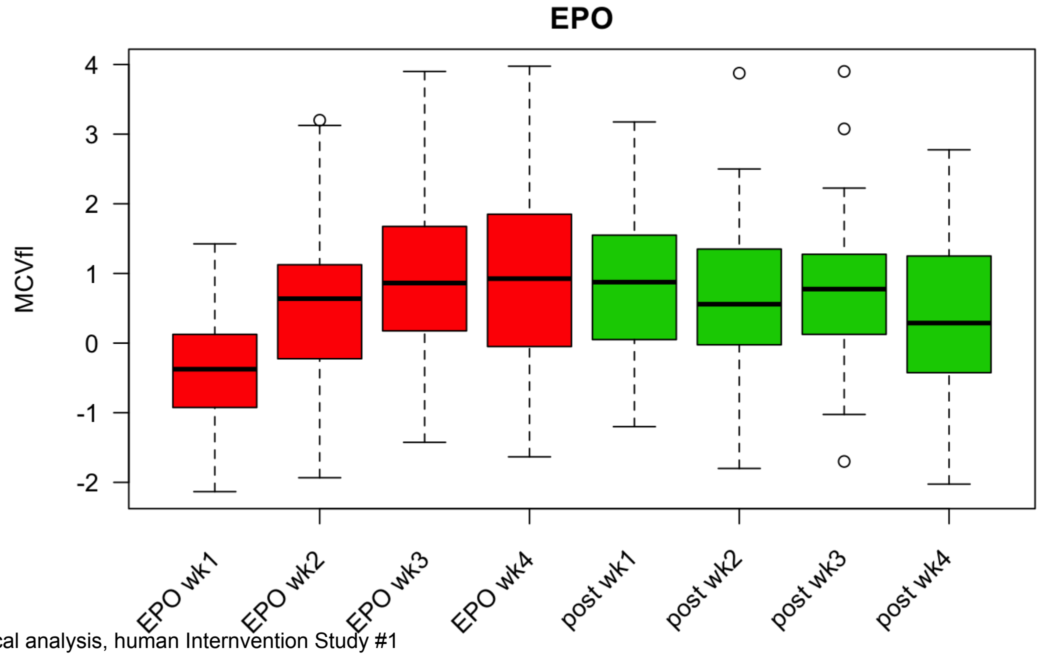
EPO
MCVfl
4
3
2
1
0
-1
-2
EPO wk1
EPO wk2
EPO wk3
EPO wk4
post wk1
post wk2
post wk3
post wk4

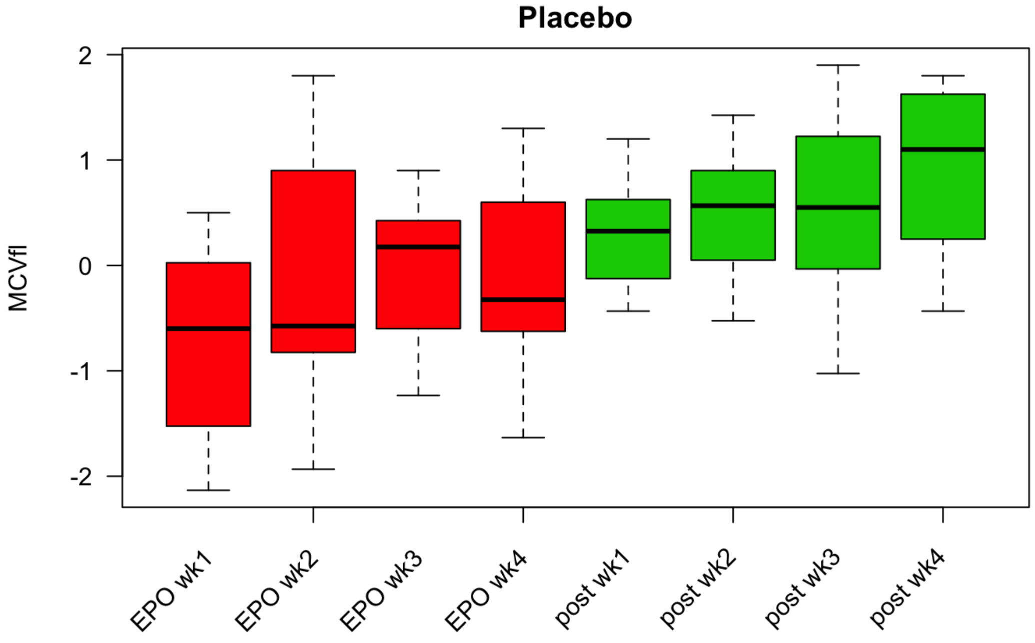
Placebo
MCVfl
2
1
0
-1
-2
EPO wk1
EPO wk2
EPO wk3
EPO wk4
post wk1
post wk2
post wk3
post wk4

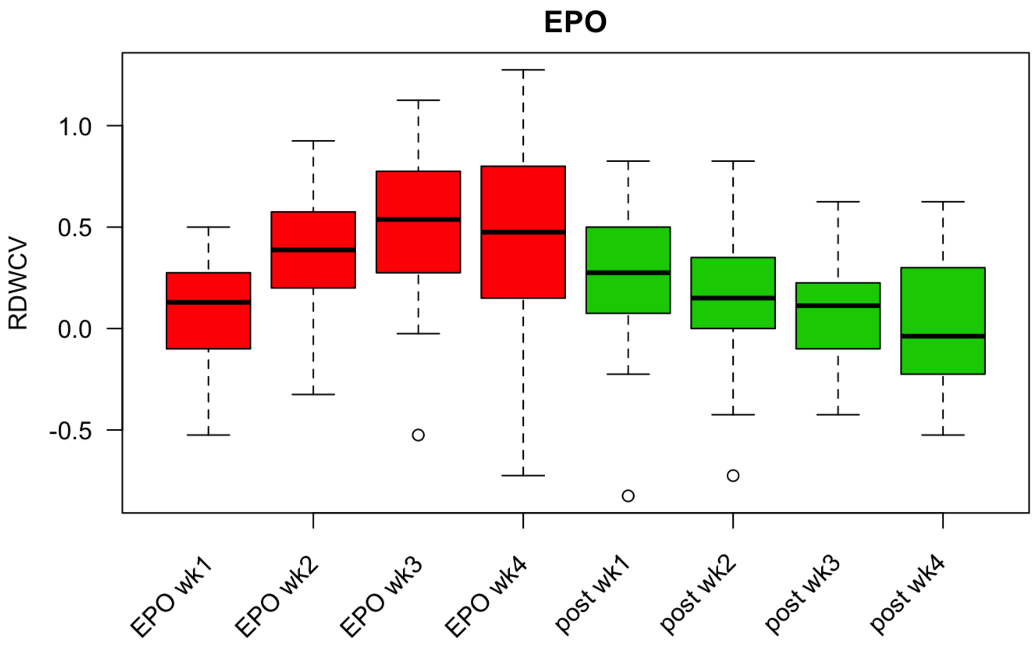
EPO
RDWCV
1.0
0.5
0.0
-0.5
EPO wk1
EPO wk2
EPO wk3
EPO wk4
post wk1
post wk2
post wk3
post wk4

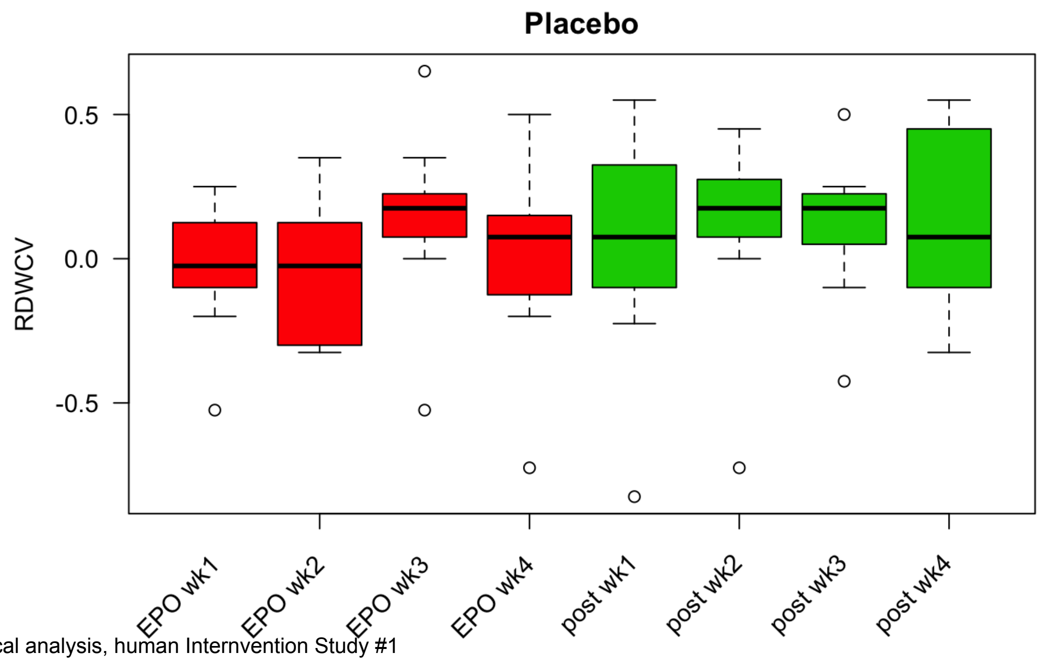
Placebo
RDWCV
0.5
0.0
-0.5
EPO wk1
EPO wk2
EPO wk3
EPO wk4
post wk1
post wk2
post wk3
post wk4

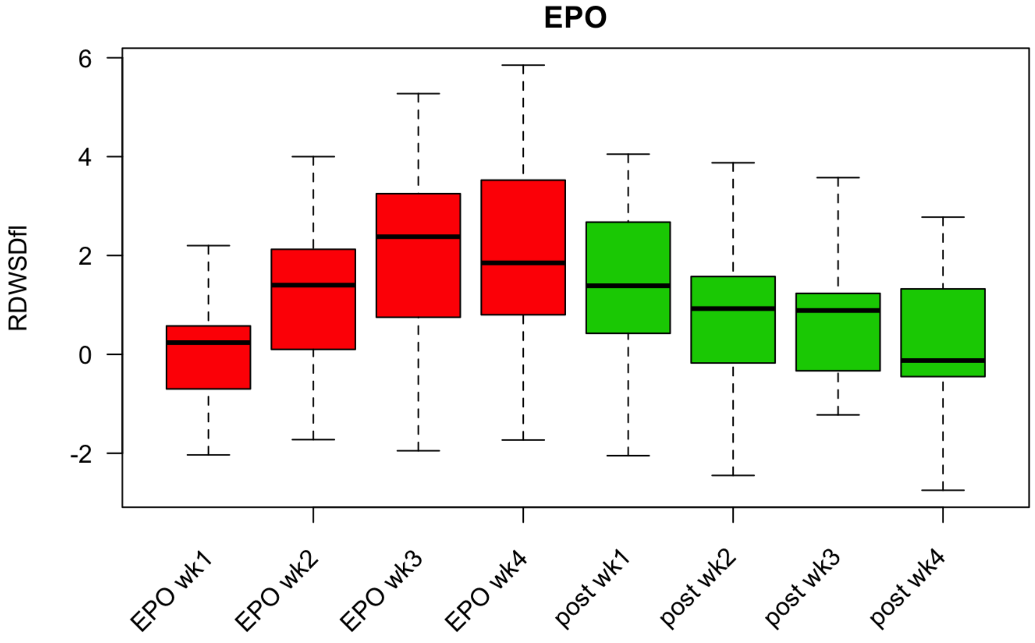
EPO
RDWSDfl
6
4
2
0
-2
EPO wk1
EPO wk2
EPO wk3
EPO wk4
post wk1
post wk2
post wk3
post wk4

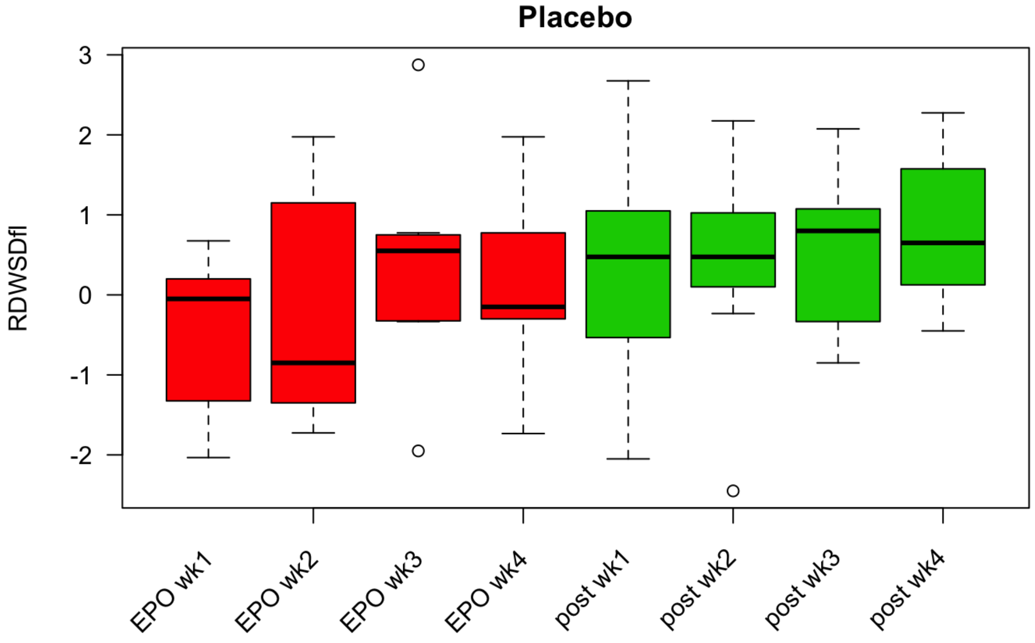
Placebo
RDWSDfl
3
2
1
0
-1
-2
EPO wk1
EPO wk2
EPO wk3
EPO wk4
post wk1
post wk2
post wk3
post wk4

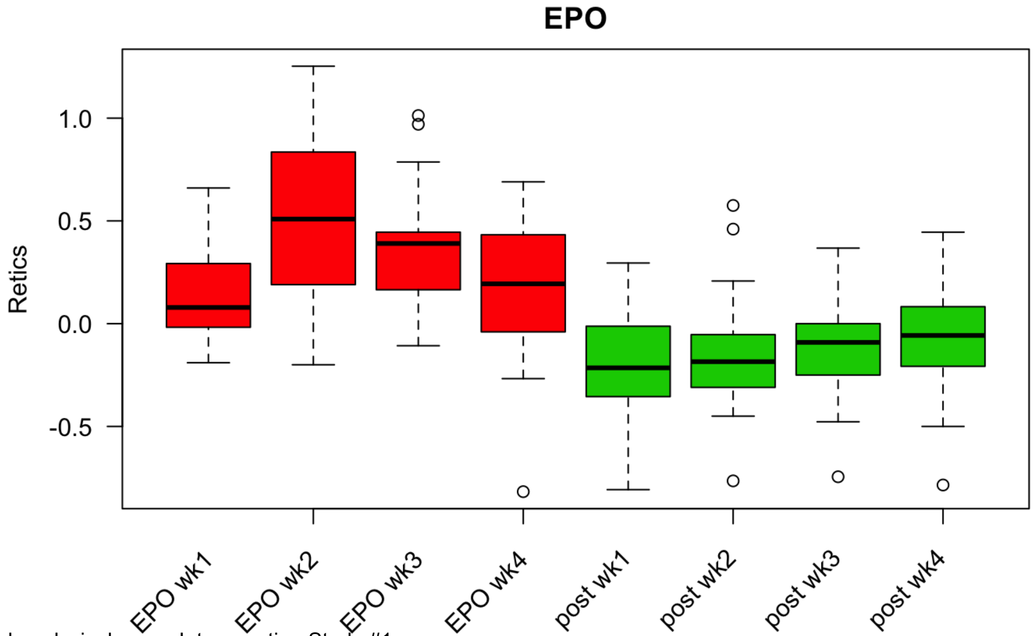
EPO
Retics
1.0
0.5
0.0
-0.5
EPO wk1
EPO wk2
EPO wk3
EPO wk4
post wk1
post wk2
post wk3
post wk4

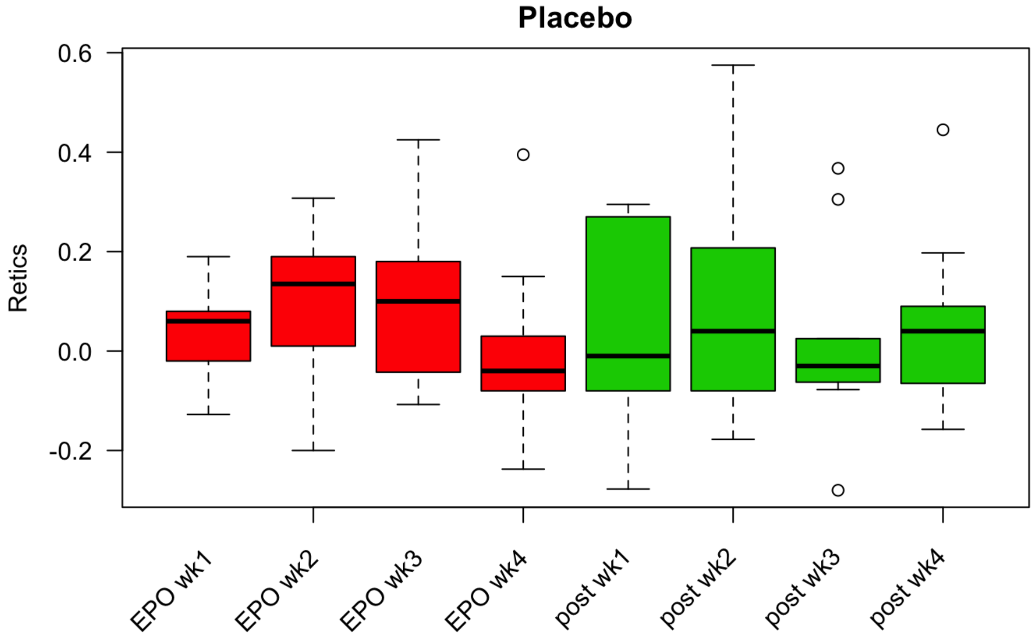
Placebo
Retics
0.6
0.4
0.2
0.0
-0.2
EPO wk1
EPO wk2
EPO wk3
EPO wk4
post wk1
post wk2
post wk3
post wk4

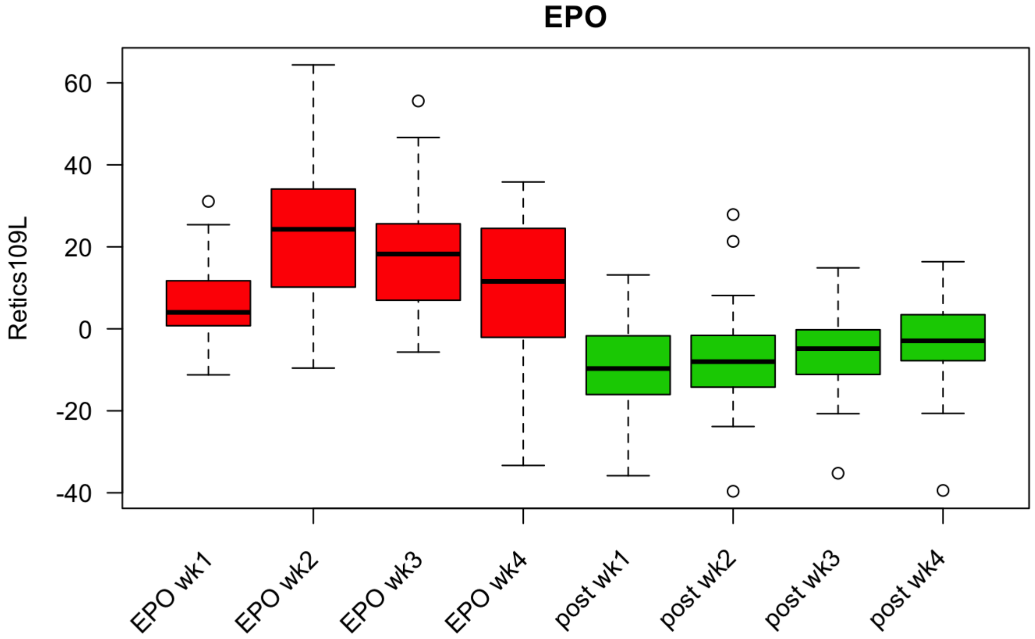
EPO
Retics109L
60
40
20
0
-20
-40
EPO wk1
EPO wk2
EPO wk3
EPO wk4
post wk1
post wk2
post wk3
post wk4

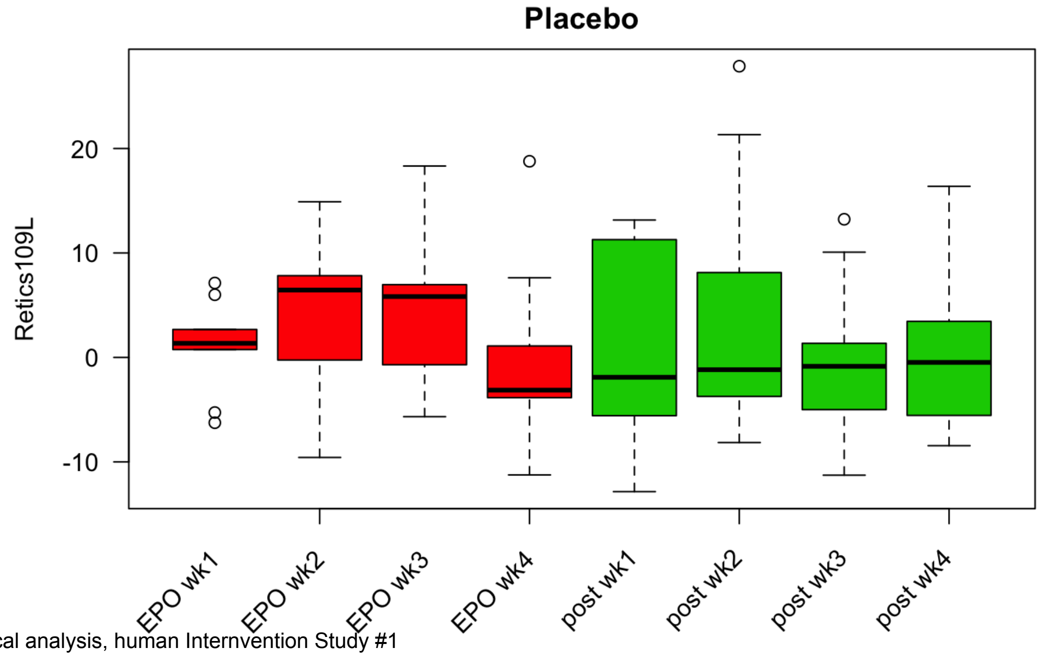
Placebo
Retics109L
20
10
0
-10
EPO wk1
EPO wk2
EPO wk3
EPO wk4
post wk1
post wk2
post wk3
post wk4

6161

```
R version 3.6.1 Patched (2019-09-23 r77210)
Platform: x86_64-apple-darwin15.6.0 (64-bit)
Running under: macOS Mojave 10.14.6

Matrix products: default
BLAS:   /System/Library/Frameworks/Accelerate.framework/Versions/A/Frameworks/vecLib.framework/Versions/A/libBLA
S.dylib
LAPACK: /Library/Frameworks/R.framework/Versions/3.6/Resources/lib/libRlapack.dylib

locale:
[1] en_US.UTF-8/en_US.UTF-8/en_US.UTF-8/C/en_US.UTF-8/en_US.UTF-8

attached base packages:
[1] stats     graphics  grDevices utils     datasets  methods   base

other attached packages:
[1] multcomp_1.4-12 TH.data_1.0-10  MASS_7.3-51.5   survival_3.1-8  mvtnorm_1.0-12  nlme_3.1-143

loaded via a namespace (and not attached):
 [1] Rcpp_1.0.3       knitr_1.27       magrittr_1.5     splines_3.6.1    lattice_0.20-38
 [6] rlang_0.4.2      stringr_1.4.0    tools_3.6.1      grid_3.6.1       xfun_0.12
[11] htmltools_0.4.0  yaml_2.2.0       digest_0.6.23    Matrix_1.2-18    base64enc_0.1-3
[16] codetools_0.2-16 rsconnect_0.8.16 evaluate_0.14    rmarkdown_2.1    sandwich_2.5-1
[21] stringi_1.4.5    compiler_3.6.1   jsonlite_1.6     zoo_1.8-7
```

Data analysis of Epo intervention study # 2, Hidalgo et al.

Code

By Lihua Julie Zhu

Citation:

1. 1. Lindstrom, M.J. and Bates, D.M. (1988) “Newton-Raphson and EM Algorithms for Linear Mixed-Effects Models for Repeated-Measures Data”, Journal of the American Statistical Association, 83, 1014–1022.
2. Hothorn T, Bretz F, Westfall P (2008). “Simultaneous Inference in General Parametric Models.” Biometrical Journal, 50(3), 346–363.
3. Benjamini, Y., and Hochberg, Y. (1995). Controlling the false discovery rate: a practical and powerful approach to multiple testing. Journal of the Royal Statistical Society Series B, 57, 289–300.http://www.jstor.org/stable/2346101.

Data summary

```
[1] "Thu Mar 19 18:28:21 2020"
[1] 384  14
```

	Treatment <fctr>	SubjectNr <int>	Days_corrected <dbl>	Erythrocytes <dbl>	Hematocrit <dbl>	Hemoglobin <dbl>	IRF <dbl>	MCH <dbl>	M... <dbl>
1	Epo	2	-25	4.90	0.430	8.7	NA	1.780	20.2
2	Epo	2	0	4.98	0.436	8.7	9.7	1.750	20.0
3	Epo	2	13	5.11	0.452	9.0	8.7	1.761	19.9
4	Epo	2	27	5.18	0.462	9.1	5.9	1.757	19.7
5	Epo	2	41	5.23	0.469	9.2	9.8	1.759	19.6
6	Epo	2	61	5.07	0.455	9.0	4.1	1.775	19.8

6 rows | 1-10 of 14 columns

```
[1] 384  14
```

	Treatment <fctr>	Subject <int>	time_point <dbl>	Erythrocytes <dbl>	Hematocrit <dbl>	Hemoglobin <dbl>	IRF <dbl>	MCH <dbl>	MC... <dbl>
1	Epo	2	-25	4.90	0.430	8.7	NA	1.780	20.2
2	Epo	2	0	4.98	0.436	8.7	9.7	1.750	20.0
3	Epo	2	13	5.11	0.452	9.0	8.7	1.761	19.9
4	Epo	2	27	5.18	0.462	9.1	5.9	1.757	19.7
5	Epo	2	41	5.23	0.469	9.2	9.8	1.759	19.6
6	Epo	2	61	5.07	0.455	9.0	4.1	1.775	19.8

6 rows | 1-10 of 14 columns

```
Correlation matrix among different measurements
             Erythrocytes  Hematocrit  Hemoglobin         IRF         MCH          MCHC          MCV
Erythrocytes   1.00000000  0.82382229  0.76224650 -0.21111015 -0.46730438 -0.123225940 -0.46685009
Hematocrit     0.82382229  1.00000000  0.92171103 -0.16972995  0.01177281 -0.152100261  0.11359931
Hemoglobin     0.76224650  0.92171103  1.00000000 -0.19673417  0.21254048  0.241721958  0.09190742
IRF           -0.21111015 -0.16972995 -0.19673417  1.00000000  0.04333773 -0.083806217  0.10655835
MCH           -0.46730438  0.01177281  0.21254048  0.04333773  1.00000000  0.520135885  0.83146491
MCHC          -0.12322594 -0.15210026  0.24172196 -0.08380622  0.52013588  1.000000000 -0.04142845
MCV           -0.46685009  0.11359931  0.09190742  0.10655835  0.83146491 -0.041428450  1.00000000
RDW_SD        -0.17643173  0.11537341 -0.01572582  0.28517235  0.25782734 -0.342623100  0.52477730
Reticulo_per  -0.11694348 -0.07618071 -0.04115495  0.56324228  0.12007292  0.096009557  0.07928422
Reticulocyte   0.11219894  0.11718677  0.13663948  0.51440820  0.01013854  0.056001662 -0.02290604
Thrombocytes   0.05332675  0.08754265  0.08085743  0.05574185  0.02160299 -0.007037408  0.03028515
                  RDW_SD Reticulo_per Reticulocyte Thrombocytes
Erythrocytes -0.17643173  -0.11694348   0.11219894  0.053326750
Hematocrit    0.11537341  -0.07618071   0.11718677  0.087542655
Hemoglobin   -0.01572582  -0.04115495   0.13663948  0.080857433
IRF           0.28517235   0.56324228   0.51440820  0.055741847
MCH           0.25782734   0.12007292   0.01013854  0.021602992
MCHC         -0.34262310   0.09600956   0.05600166 -0.007037408
MCV           0.52477730   0.07928422  -0.02290604  0.030285153
RDW_SD        1.00000000   0.15792487   0.12172182 -0.051137552
Reticulo_per  0.15792487   1.00000000   0.97429694  0.307169691
Reticulocyte  0.12172182   0.97429694   1.00000000  0.297618890
Thrombocytes -0.05113755   0.30716969   0.29761889  1.000000000
```

Compute mean of 4 baseline points for each subject

	Treatment <fctr>	Subject <int>	time_point <dbl>	Erythrocytes <dbl>	Hematocrit <dbl>	Hemoglobin <dbl>	IRF <dbl>	MCH <dbl>	MC... <dbl>
1	Epo	13	-25	5.04	0.453	9.4	NA	1.860	20.8
2	Epo	13	0	4.87	0.440	9.0	13.9	1.850	20.5
3	Epo	13	13	5.01	0.462	9.2	13.3	1.836	19.9
4	Epo	13	27	5.26	0.489	9.6	15.0	1.825	19.6
5	Epo	13	41	5.35	0.492	9.9	11.8	1.850	20.1

	Treatment	Subject	time_point	Erythrocytes	Hematocrit	Hemoglobin	IRF	MCH	MC...
	<fctr>	<int>	<dbl>	<dbl>	<dbl>	<dbl>	<dbl>	<dbl>	<dbl>
6363 6	Epo	13	61	5.41	0.495	9.8	8.7	1.811	19.8

6 rows | 1-10 of 25 columns

```
[1] 288  25
[1] 13 27 41 61 62 80
```

	Treatment	Subject	time_point	Erythrocytes	Hematocrit	Hemoglobin	IRF	MCH	MCHC
	<fctr>	<int>	<dbl>	<dbl>	<dbl>	<dbl>	<dbl>	<dbl>	<dbl>
3	Epo	13	13	0.055	0.0155	0.0	-0.6	-0.019	-0.75
4	Epo	13	27	0.305	0.0425	0.4	1.1	-0.030	-1.05
5	Epo	13	41	0.395	0.0455	0.7	-2.1	-0.005	-0.55
6	Epo	13	61	0.455	0.0485	0.6	-5.2	-0.044	-0.85
7	Epo	13	62	0.375	0.0385	0.6	-8.6	-0.016	-0.45
8	Epo	13	80	0.405	0.0515	0.6	-3.8	-0.027	-0.95

6 rows | 1-10 of 25 columns

```
########Number of Subjects for each treatment ######
    Epo Placebo
     24      24
```

```
##############################################################

###############################################################

########### Repeated measures analysis results for  Erythrocytes ############

################################################################
```

	numDF	denDF	F-value	p-value
	<int>	<dbl>	<chr>	<chr>
(Intercept)	1	221	55.57089	<.0001
Treatment	1	46	59.51913	<.0001
time_point	5	221	30.48662	<.0001
Treatment:time_point	5	221	3.86877	0.0022

4 rows

6464

```
########### Compare Epo with Palcebo at each time point using contrasts ###########


################################################################

######################## Raw p-values ########################


     Simultaneous Tests for General Linear Hypotheses

Multiple Comparisons of Means: User-defined Contrasts


Fit: lme.formula(fixed = thisY ~ -1 + EpoTime, data = y, random = ~1 |
    Subject)

Linear Hypotheses:
                            Estimate Std. Error z value Pr(>|z|)
EpoVsPlacebo.EpoDay13 == 0   0.20542    0.05979   3.435 0.000591 ***
EpoVsPlacebo.EpoDay27 == 0   0.32784    0.06079   5.393 6.94e-08 ***
EpoVsPlacebo.EpoDay41 == 0   0.34185    0.06032   5.667 1.45e-08 ***
EpoVsPlacebo.EpoDay61 == 0   0.39138    0.06085   6.432 1.26e-10 ***
EpoVsPlacebo.postDay62 == 0  0.45753    0.06187   7.395 1.42e-13 ***
EpoVsPlacebo.postDay80 == 0  0.22375    0.05979   3.742 0.000182 ***
---
Signif. codes:  0 '***' 0.001 '**' 0.01 '*' 0.05 '.' 0.1 ' ' 1
(Adjusted p values reported -- none method)

##########################################################


############## BH adjusted p-values ####################
Benjamini, Y., and Hochberg, Y. (1995). Controlling the false discovery rate: a practical and powerful approach t
o multiple testing. Journal of the Royal Statistical Society Series B, 57, 289–300.http://www.jstor.org/stable/23
46101.
##############################################################

     Simultaneous Tests for General Linear Hypotheses

Multiple Comparisons of Means: User-defined Contrasts


Fit: lme.formula(fixed = thisY ~ -1 + EpoTime, data = y, random = ~1 |
    Subject)

Linear Hypotheses:
                            Estimate Std. Error z value Pr(>|z|)
EpoVsPlacebo.EpoDay13 == 0   0.20542    0.05979   3.435 0.000591 ***
EpoVsPlacebo.EpoDay27 == 0   0.32784    0.06079   5.393 1.04e-07 ***
EpoVsPlacebo.EpoDay41 == 0   0.34185    0.06032   5.667 2.90e-08 ***
EpoVsPlacebo.EpoDay61 == 0   0.39138    0.06085   6.432 3.77e-10 ***
EpoVsPlacebo.postDay62 == 0  0.45753    0.06187   7.395 8.51e-13 ***
EpoVsPlacebo.postDay80 == 0  0.22375    0.05979   3.742 0.000219 ***
---
Signif. codes:  0 '***' 0.001 '**' 0.01 '*' 0.05 '.' 0.1 ' ' 1
(Adjusted p values reported -- BH method)


#############################################################
```

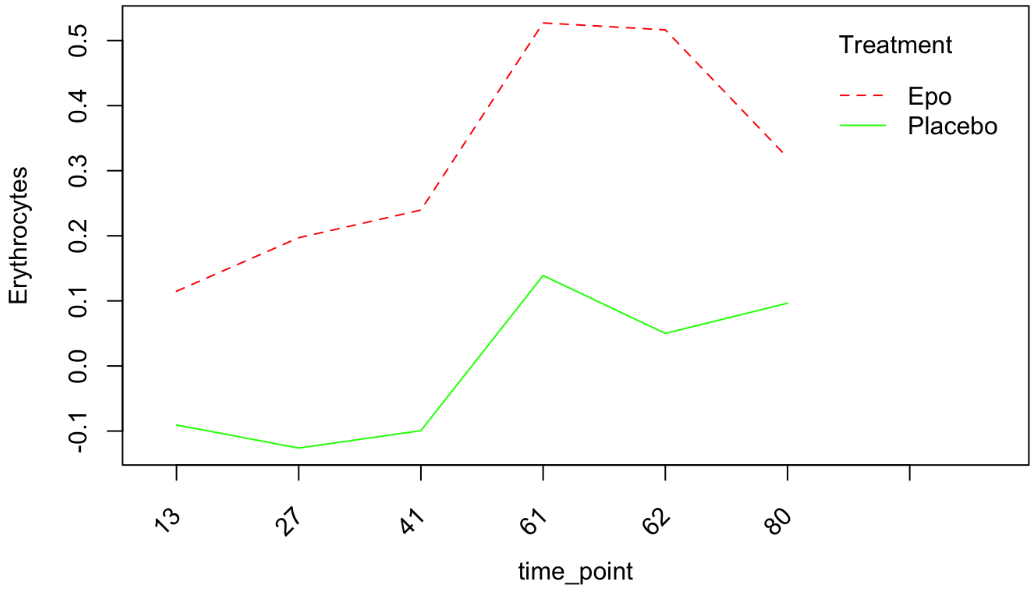

6565
```
##############################################################

########### Repeated measures analysis results for  Hematocrit ############

###############################################################
```

	numDF	denDF	F-value	p-value
	<int>	<dbl>	<chr>	<chr>
(Intercept)	1	221	160.33697	<.0001
Treatment	1	46	100.41793	<.0001
time_point	5	221	30.58785	<.0001
Treatment:time_point	5	221	5.44892	1e-04

4 rows

```
############ Compare Epo with Palcebo at each time point using contrasts ###########


###############################################################

######################## Raw p-values ########################


     Simultaneous Tests for General Linear Hypotheses

Multiple Comparisons of Means: User-defined Contrasts


Fit: lme.formula(fixed = thisY ~ -1 + EpoTime, data = y, random = ~1 |
    Subject)

Linear Hypotheses:
                            Estimate Std. Error z value Pr(>|z|)
EpoVsPlacebo.EpoDay13 == 0  0.023521   0.005168   4.551 5.33e-06 ***
EpoVsPlacebo.EpoDay27 == 0  0.036135   0.005260   6.870 6.42e-12 ***
EpoVsPlacebo.EpoDay41 == 0  0.037982   0.005216   7.282 3.29e-13 ***
EpoVsPlacebo.EpoDay61 == 0  0.041754   0.005265   7.931 2.22e-15 ***
EpoVsPlacebo.postDay62 == 0 0.047576   0.005359   8.877  < 2e-16 ***
EpoVsPlacebo.postDay80 == 0 0.021521   0.005168   4.164 3.12e-05 ***
---
Signif. codes:  0 ‘***’ 0.001 ‘**’ 0.01 ‘*’ 0.05 ‘.’ 0.1 ‘ ’ 1
(Adjusted p values reported -- none method)

##########################################################


############## BH adjusted p-values #####################
Benjamini, Y., and Hochberg, Y. (1995). Controlling the false discovery rate: a practical and powerful approach t
o multiple testing. Journal of the Royal Statistical Society Series B, 57, 289–300.http://www.jstor.org/stable/23
46101.
##############################################################

     Simultaneous Tests for General Linear Hypotheses

Multiple Comparisons of Means: User-defined Contrasts


Fit: lme.formula(fixed = thisY ~ -1 + EpoTime, data = y, random = ~1 |
    Subject)

Linear Hypotheses:
                            Estimate Std. Error z value Pr(>|z|)
EpoVsPlacebo.EpoDay13 == 0  0.023521   0.005168   4.551 6.40e-06 ***
EpoVsPlacebo.EpoDay27 == 0  0.036135   0.005260   6.870 9.62e-12 ***
EpoVsPlacebo.EpoDay41 == 0  0.037982   0.005216   7.282 6.59e-13 ***
EpoVsPlacebo.EpoDay61 == 0  0.041754   0.005265   7.931 6.66e-15 ***
EpoVsPlacebo.postDay62 == 0 0.047576   0.005359   8.877  < 2e-16 ***
EpoVsPlacebo.postDay80 == 0 0.021521   0.005168   4.164 3.12e-05 ***
---
Signif. codes:  0 ‘***’ 0.001 ‘**’ 0.01 ‘*’ 0.05 ‘.’ 0.1 ‘ ’ 1
(Adjusted p values reported -- BH method)


#############################################################
```

6666

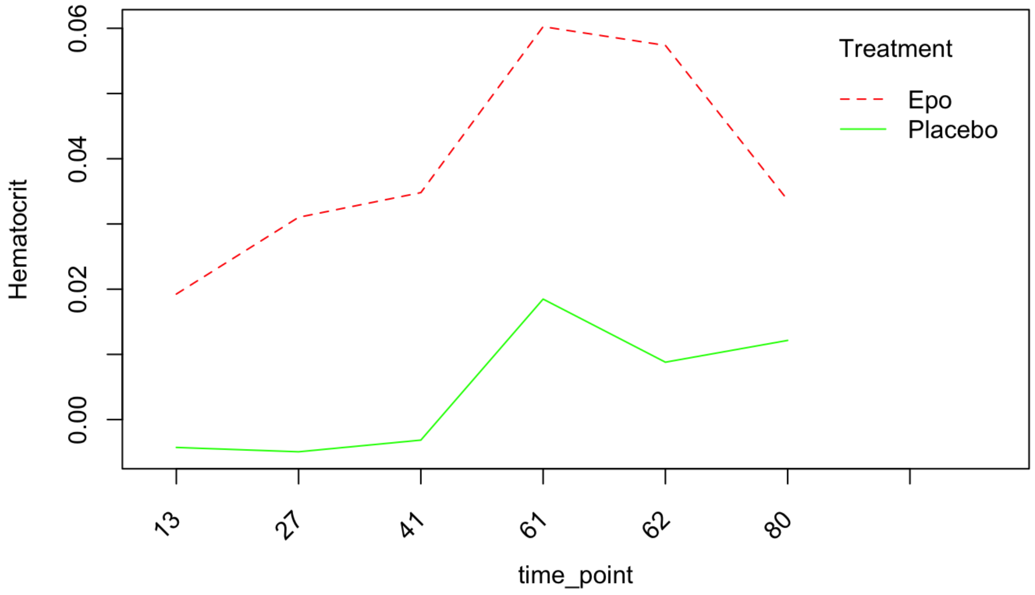


```
##############################################################

########### Repeated measures analysis results for  Hemoglobin ############

###############################################################
```

	numDF <int>	denDF <dbl>	F-value <chr>	p-value <chr>
(Intercept)	1	221	24.81583	<.0001
Treatment	1	46	60.47537	<.0001
time_point	5	221	38.73160	<.0001
Treatment:time_point	5	221	3.92180	0.002

4 rows

6767

```
########### Compare Epo with Palcebo at each time point using contrasts ##########


###############################################################

######################## Raw p-values ########################


     Simultaneous Tests for General Linear Hypotheses

Multiple Comparisons of Means: User-defined Contrasts


Fit: lme.formula(fixed = thisY ~ -1 + EpoTime, data = y, random = ~1 |
    Subject)

Linear Hypotheses:
                            Estimate Std. Error z value Pr(>|z|)
EpoVsPlacebo.EpoDay13 == 0    0.4229     0.1129   3.745 0.000181 ***
EpoVsPlacebo.EpoDay27 == 0    0.6827     0.1148   5.947 2.73e-09 ***
EpoVsPlacebo.EpoDay41 == 0    0.6953     0.1139   6.104 1.04e-09 ***
EpoVsPlacebo.EpoDay61 == 0    0.7571     0.1149   6.589 4.41e-11 ***
EpoVsPlacebo.postDay62 == 0   0.8204     0.1168   7.024 2.16e-12 ***
EpoVsPlacebo.postDay80 == 0   0.3812     0.1129   3.376 0.000736 ***
---
Signif. codes:  0 '***' 0.001 '**' 0.01 '*' 0.05 '.' 0.1 ' ' 1
(Adjusted p values reported -- none method)

##########################################################


############## BH adjusted p-values ####################
Benjamini, Y., and Hochberg, Y. (1995). Controlling the false discovery rate: a practical and powerful approach t
o multiple testing. Journal of the Royal Statistical Society Series B, 57, 289—300.http://www.jstor.org/stable/23
46101.
##############################################################

     Simultaneous Tests for General Linear Hypotheses

Multiple Comparisons of Means: User-defined Contrasts


Fit: lme.formula(fixed = thisY ~ -1 + EpoTime, data = y, random = ~1 |
    Subject)

Linear Hypotheses:
                            Estimate Std. Error z value Pr(>|z|)
EpoVsPlacebo.EpoDay13 == 0    0.4229     0.1129   3.745 0.000217 ***
EpoVsPlacebo.EpoDay27 == 0    0.6827     0.1148   5.947 4.10e-09 ***
EpoVsPlacebo.EpoDay41 == 0    0.6953     0.1139   6.104 2.07e-09 ***
EpoVsPlacebo.EpoDay61 == 0    0.7571     0.1149   6.589 1.32e-10 ***
EpoVsPlacebo.postDay62 == 0   0.8204     0.1168   7.024 1.30e-11 ***
EpoVsPlacebo.postDay80 == 0   0.3812     0.1129   3.376 0.000736 ***
---
Signif. codes:  0 '***' 0.001 '**' 0.01 '*' 0.05 '.' 0.1 ' ' 1
(Adjusted p values reported -- BH method)


#############################################################
```

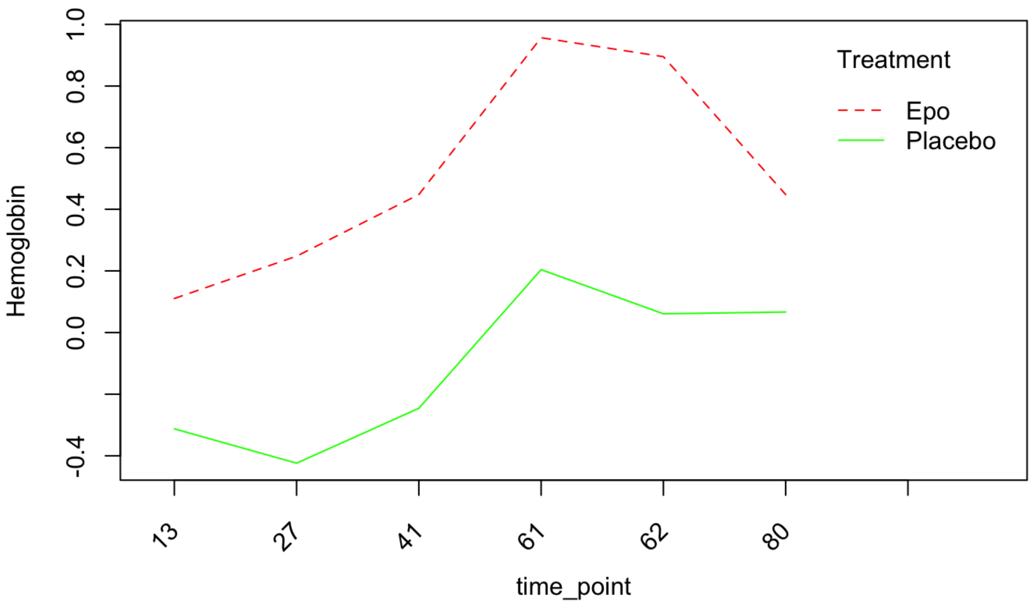

```
##############################################################

########### Repeated measures analysis results for  IRF ############

###############################################################
```

	numDF	denDF	F-value	p-value
	<int>	<dbl>	<chr>	<chr>
(Intercept)	1	221	0.049419	0.8243
Treatment	1	46	1.536417	0.2214
time_point	5	221	14.500704	<.0001
Treatment:time_point	5	221	18.468077	<.0001

4 rows

```
############ Compare Epo with Palcebo at each time point using contrasts ###########


###############################################################

######################## Raw p-values ########################


     Simultaneous Tests for General Linear Hypotheses

Multiple Comparisons of Means: User-defined Contrasts


Fit: lme.formula(fixed = thisY ~ -1 + EpoTime, data = y, random = ~1 |
    Subject)

Linear Hypotheses:
                            Estimate Std. Error z value Pr(>|z|)
EpoVsPlacebo.EpoDay13 == 0    1.6333     0.8013   2.038  0.04153 *
EpoVsPlacebo.EpoDay27 == 0    0.9957     0.8117   1.227  0.21996
EpoVsPlacebo.EpoDay41 == 0    1.4390     0.8069   1.783  0.07454 .
EpoVsPlacebo.EpoDay61 == 0   -3.2588     0.8123  -4.012 6.03e-05 ***
EpoVsPlacebo.postDay62 == 0  -3.4052     0.8228  -4.139 3.49e-05 ***
EpoVsPlacebo.postDay80 == 0  -2.4167     0.8013  -3.016  0.00256 **
---
Signif. codes:  0 ‘***’ 0.001 ‘**’ 0.01 ‘*’ 0.05 ‘.’ 0.1 ‘ ’ 1
(Adjusted p values reported -- none method)

##########################################################


############## BH adjusted p-values ####################
Benjamini, Y., and Hochberg, Y. (1995). Controlling the false discovery rate: a practical and powerful approach t
o multiple testing. Journal of the Royal Statistical Society Series B, 57, 289–300.http://www.jstor.org/stable/23
46101.
##############################################################

     Simultaneous Tests for General Linear Hypotheses

Multiple Comparisons of Means: User-defined Contrasts


Fit: lme.formula(fixed = thisY ~ -1 + EpoTime, data = y, random = ~1 |
    Subject)

Linear Hypotheses:
                            Estimate Std. Error z value Pr(>|z|)
EpoVsPlacebo.EpoDay13 == 0    1.6333     0.8013   2.038 0.062289 .
EpoVsPlacebo.EpoDay27 == 0    0.9957     0.8117   1.227 0.219965
EpoVsPlacebo.EpoDay41 == 0    1.4390     0.8069   1.783 0.089447 .
EpoVsPlacebo.EpoDay61 == 0   -3.2588     0.8123  -4.012 0.000181 ***
EpoVsPlacebo.postDay62 == 0  -3.4052     0.8228  -4.139 0.000181 ***
EpoVsPlacebo.postDay80 == 0  -2.4167     0.8013  -3.016 0.005127 **
---
Signif. codes:  0 ‘***’ 0.001 ‘**’ 0.01 ‘*’ 0.05 ‘.’ 0.1 ‘ ’ 1
(Adjusted p values reported -- BH method)


#############################################################
```

6969

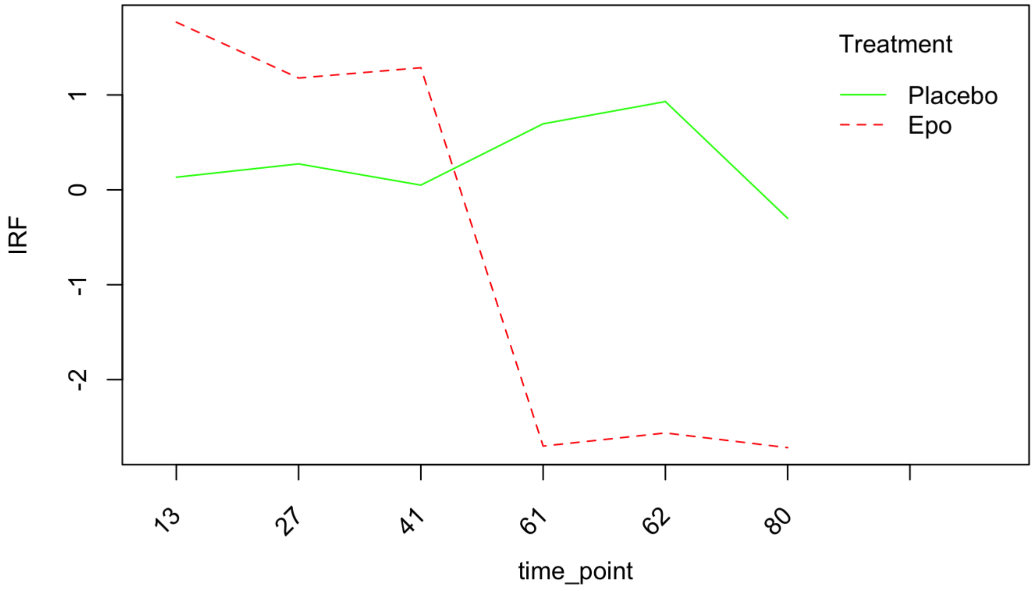


```
#################################################################
########### Repeated measures analysis results for  MCH ############
##################################################################
```

	numDF	denDF	F-value	p-value
	<int>	<dbl>	<chr>	<chr>
(Intercept)	1	220	24.770706	<.0001
Treatment	1	46	0.196088	0.6600
time_point	5	220	15.091237	<.0001
Treatment:time_point	5	220	2.595238	0.0264

4 rows

```
############ Compare Epo with Palcebo at each time point using contrasts ###########


################################################################

######################## Raw p-values ########################


     Simultaneous Tests for General Linear Hypotheses

Multiple Comparisons of Means: User-defined Contrasts


Fit: lme.formula(fixed = thisY ~ -1 + EpoTime, data = y, random = ~1 |
    Subject)

Linear Hypotheses:
                             Estimate Std. Error z value Pr(>|z|)
EpoVsPlacebo.EpoDay13 == 0   0.008750   0.008861   0.987    0.323
EpoVsPlacebo.EpoDay27 == 0   0.013783   0.008970   1.537    0.124
EpoVsPlacebo.EpoDay41 == 0   0.010579   0.008924   1.186    0.236
EpoVsPlacebo.EpoDay61 == 0   0.003309   0.008975   0.369    0.712
EpoVsPlacebo.postDay62 == 0 -0.009398   0.009075  -1.036    0.300
EpoVsPlacebo.postDay80 == 0 -0.006019   0.008924  -0.674    0.500
(Adjusted p values reported -- none method)

##########################################################


############## BH adjusted p-values ####################
Benjamini, Y., and Hochberg, Y. (1995). Controlling the false discovery rate: a practical and powerful approach t
o multiple testing. Journal of the Royal Statistical Society Series B, 57, 289–300.http://www.jstor.org/stable/23
46101.
##############################################################

     Simultaneous Tests for General Linear Hypotheses

Multiple Comparisons of Means: User-defined Contrasts


Fit: lme.formula(fixed = thisY ~ -1 + EpoTime, data = y, random = ~1 |
    Subject)

Linear Hypotheses:
                             Estimate Std. Error z value Pr(>|z|)
EpoVsPlacebo.EpoDay13 == 0   0.008750   0.008861   0.987    0.485
EpoVsPlacebo.EpoDay27 == 0   0.013783   0.008970   1.537    0.485
EpoVsPlacebo.EpoDay41 == 0   0.010579   0.008924   1.186    0.485
EpoVsPlacebo.EpoDay61 == 0   0.003309   0.008975   0.369    0.712
EpoVsPlacebo.postDay62 == 0 -0.009398   0.009075  -1.036    0.485
EpoVsPlacebo.postDay80 == 0 -0.006019   0.008924  -0.674    0.600
(Adjusted p values reported -- BH method)


#############################################################
```

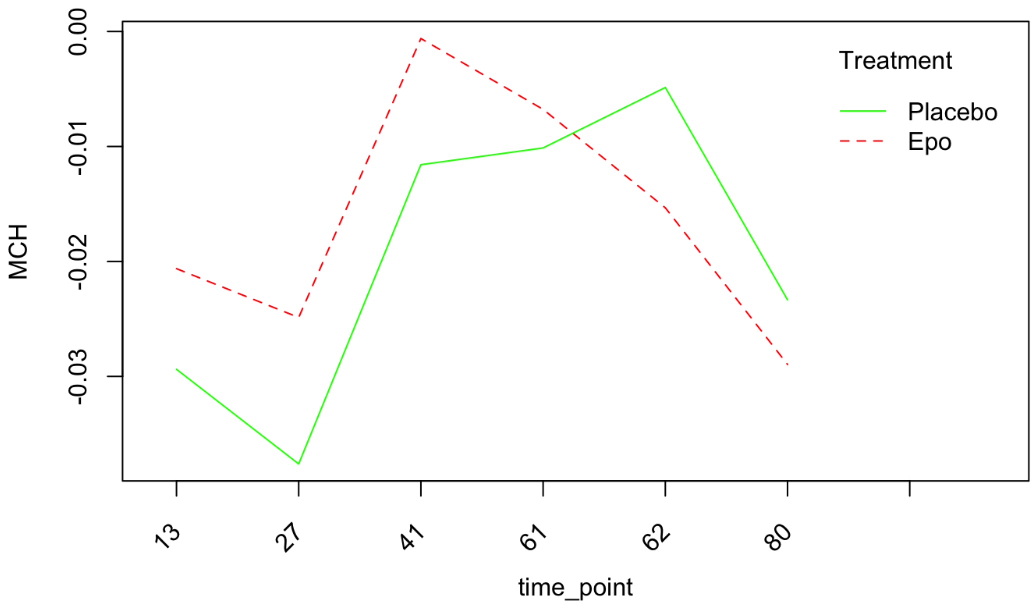


```
##############################################################

########### Repeated measures analysis results for  MCHC ############
```



```
##############################################################
```

7171

	numDF <int>	denDF <dbl>	F-value <chr>	p-value <chr>
(Intercept)	1	221	225.14362	<.0001
Treatment	1	46	5.45011	0.0240
time_point	5	221	10.71895	<.0001
Treatment:time_point	5	221	0.94348	0.4537

4 rows

```
########### Compare Epo with Palcebo at each time point using contrasts ###########


###############################################################

####################### Raw p-values #######################


     Simultaneous Tests for General Linear Hypotheses

Multiple Comparisons of Means: User-defined Contrasts


Fit: lme.formula(fixed = thisY ~ -1 + EpoTime, data = y, random = ~1 |
    Subject)

Linear Hypotheses:
                            Estimate Std. Error z value Pr(>|z|)
EpoVsPlacebo.EpoDay13 == 0   -0.1042     0.1017  -1.024  0.30580
EpoVsPlacebo.EpoDay27 == 0   -0.1126     0.1033  -1.089  0.27598
EpoVsPlacebo.EpoDay41 == 0   -0.1607     0.1026  -1.567  0.11710
EpoVsPlacebo.EpoDay61 == 0   -0.1896     0.1034  -1.833  0.06677 .
EpoVsPlacebo.postDay62 == 0  -0.3171     0.1051  -3.018  0.00255 **
EpoVsPlacebo.postDay80 == 0  -0.1250     0.1017  -1.229  0.21911
---
Signif. codes:  0 ‘***’ 0.001 ‘**’ 0.01 ‘*’ 0.05 ‘.’ 0.1 ‘ ’ 1
(Adjusted p values reported -- none method)

##########################################################


############# BH adjusted p-values ####################
Benjamini, Y., and Hochberg, Y. (1995). Controlling the false discovery rate: a practical and powerful approach t
o multiple testing. Journal of the Royal Statistical Society Series B, 57, 289–300.http://www.jstor.org/stable/23
46101.
##############################################################

     Simultaneous Tests for General Linear Hypotheses

Multiple Comparisons of Means: User-defined Contrasts


Fit: lme.formula(fixed = thisY ~ -1 + EpoTime, data = y, random = ~1 |
    Subject)

Linear Hypotheses:
                            Estimate Std. Error z value Pr(>|z|)
EpoVsPlacebo.EpoDay13 == 0   -0.1042     0.1017  -1.024   0.3058
EpoVsPlacebo.EpoDay27 == 0   -0.1126     0.1033  -1.089   0.3058
EpoVsPlacebo.EpoDay41 == 0   -0.1607     0.1026  -1.567   0.2342
EpoVsPlacebo.EpoDay61 == 0   -0.1896     0.1034  -1.833   0.2003
EpoVsPlacebo.postDay62 == 0  -0.3171     0.1051  -3.018   0.0153 *
EpoVsPlacebo.postDay80 == 0  -0.1250     0.1017  -1.229   0.3058
---
Signif. codes:  0 ‘***’ 0.001 ‘**’ 0.01 ‘*’ 0.05 ‘.’ 0.1 ‘ ’ 1
(Adjusted p values reported -- BH method)


#############################################################
```

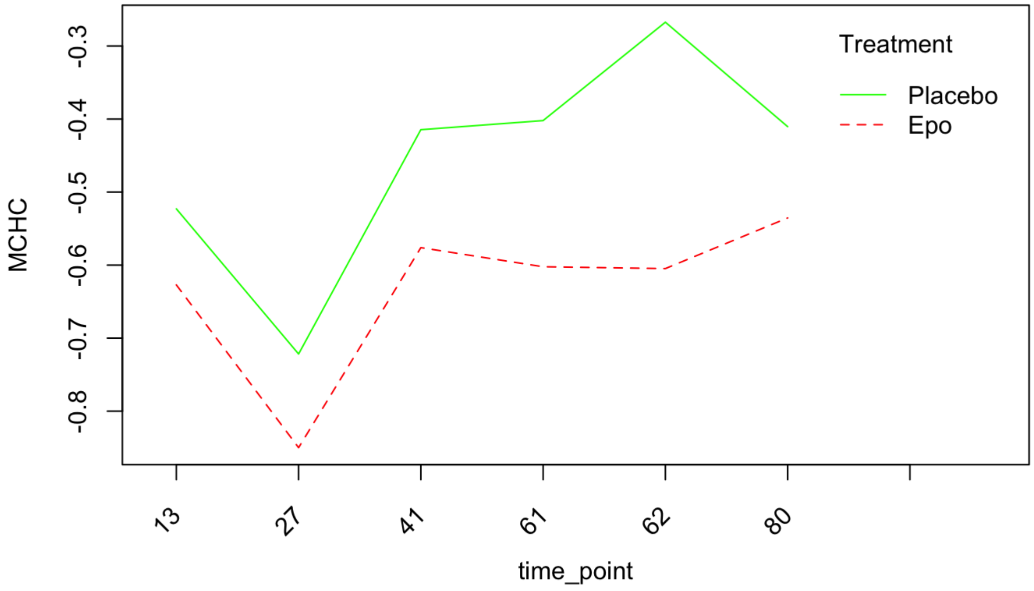


```
##############################################################

########### Repeated measures analysis results for  MCV ############

###############################################################
```

	numDF <int>	denDF <dbl>	F-value <chr>	p-value <chr>
(Intercept)	1	221	105.96797	<.0001
Treatment	1	46	11.91240	0.0012
time_point	5	221	11.83883	<.0001
Treatment:time_point	5	221	1.87568	0.0996

4 rows

```
############ Compare Epo with Palcebo at each time point using contrasts ###########
```

7373

```
################################################################

######################## Raw p-values ########################


     Simultaneous Tests for General Linear Hypotheses

Multiple Comparisons of Means: User-defined Contrasts


Fit: lme.formula(fixed = thisY ~ -1 + EpoTime, data = y, random = ~1 |
    Subject)

Linear Hypotheses:
                            Estimate Std. Error z value Pr(>|z|)
EpoVsPlacebo.EpoDay13 == 0    1.0375     0.3742   2.773 0.005559 **
EpoVsPlacebo.EpoDay27 == 0    1.2458     0.3789   3.288 0.001010 **
EpoVsPlacebo.EpoDay41 == 0    1.3542     0.3767   3.595 0.000325 ***
EpoVsPlacebo.EpoDay61 == 0    1.1483     0.3792   3.028 0.002462 **
EpoVsPlacebo.postDay62 == 0   1.0576     0.3840   2.754 0.005886 **
EpoVsPlacebo.postDay80 == 0   0.3542     0.3742   0.947 0.343884
---
Signif. codes:  0 '***' 0.001 '**' 0.01 '*' 0.05 '.' 0.1 ' ' 1
(Adjusted p values reported -- none method)

###########################################################


############## BH adjusted p-values #####################
Benjamini, Y., and Hochberg, Y. (1995). Controlling the false discovery rate: a practical and powerful approach t
o multiple testing. Journal of the Royal Statistical Society Series B, 57, 289–300.http://www.jstor.org/stable/23
46101.
###############################################################

     Simultaneous Tests for General Linear Hypotheses

Multiple Comparisons of Means: User-defined Contrasts


Fit: lme.formula(fixed = thisY ~ -1 + EpoTime, data = y, random = ~1 |
    Subject)

Linear Hypotheses:
                            Estimate Std. Error z value Pr(>|z|)
EpoVsPlacebo.EpoDay13 == 0    1.0375     0.3742   2.773  0.00706 **
EpoVsPlacebo.EpoDay27 == 0    1.2458     0.3789   3.288  0.00303 **
EpoVsPlacebo.EpoDay41 == 0    1.3542     0.3767   3.595  0.00195 **
EpoVsPlacebo.EpoDay61 == 0    1.1483     0.3792   3.028  0.00492 **
EpoVsPlacebo.postDay62 == 0   1.0576     0.3840   2.754  0.00706 **
EpoVsPlacebo.postDay80 == 0   0.3542     0.3742   0.947  0.34388
---
Signif. codes:  0 '***' 0.001 '**' 0.01 '*' 0.05 '.' 0.1 ' ' 1
(Adjusted p values reported -- BH method)


##############################################################
```

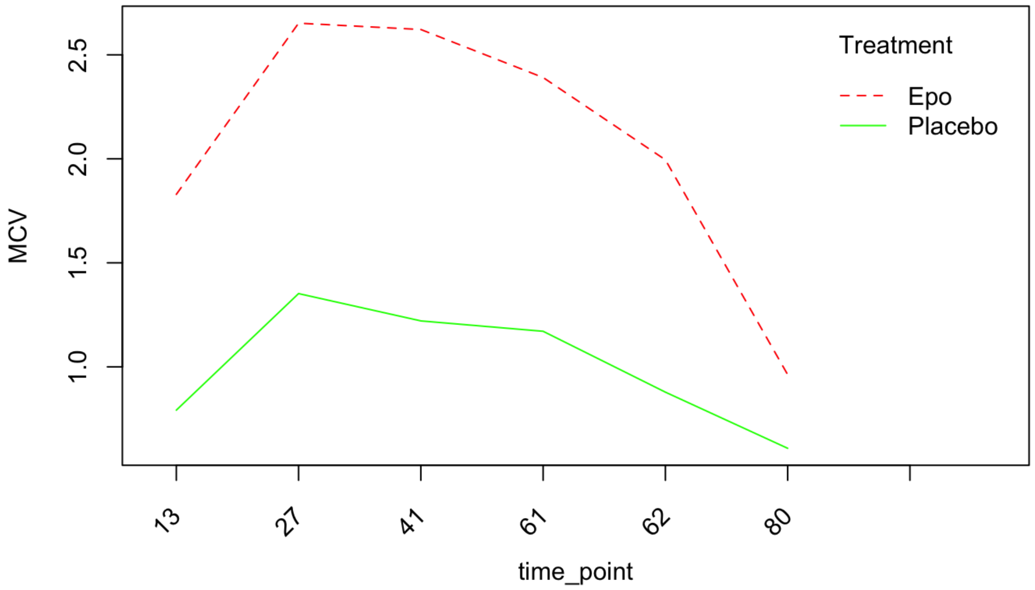

```
##############################################################
########### Repeated measures analysis results for  RDW_SD ############
###############################################################
```

	numDF	denDF	F-value	p-value
	<int>	<dbl>	<chr>	<chr>
(Intercept)	1	219	32.04582	<.0001
Treatment	1	46	17.70673	1e-04
time_point	5	219	30.09056	<.0001
Treatment:time_point	5	219	9.09271	<.0001

4 rows

```
############ Compare Epo with Palcebo at each time point using contrasts ###########


###############################################################

######################## Raw p-values ########################


     Simultaneous Tests for General Linear Hypotheses

Multiple Comparisons of Means: User-defined Contrasts


Fit: lme.formula(fixed = thisY ~ -1 + EpoTime, data = y, random = ~1 |
    Subject)

Linear Hypotheses:
                            Estimate Std. Error z value Pr(>|z|)
EpoVsPlacebo.EpoDay13 == 0    2.2750     0.4841   4.700 2.61e-06 ***
EpoVsPlacebo.EpoDay27 == 0    2.1992     0.4925   4.466 7.97e-06 ***
EpoVsPlacebo.EpoDay41 == 0    2.1981     0.4874   4.510 6.49e-06 ***
EpoVsPlacebo.EpoDay61 == 0    1.7946     0.4901   3.661 0.000251 ***
EpoVsPlacebo.postDay62 == 0   1.4846     0.4954   2.997 0.002728 **
EpoVsPlacebo.postDay80 == 0  -0.1585     0.4874  -0.325 0.745109
---
Signif. codes:  0 ‘***’ 0.001 ‘**’ 0.01 ‘*’ 0.05 ‘.’ 0.1 ‘ ’ 1
(Adjusted p values reported -- none method)

##########################################################


############## BH adjusted p-values ####################
Benjamini, Y., and Hochberg, Y. (1995). Controlling the false discovery rate: a practical and powerful approach t
o multiple testing. Journal of the Royal Statistical Society Series B, 57, 289–300.http://www.jstor.org/stable/23
46101.
##############################################################

     Simultaneous Tests for General Linear Hypotheses

Multiple Comparisons of Means: User-defined Contrasts


Fit: lme.formula(fixed = thisY ~ -1 + EpoTime, data = y, random = ~1 |
    Subject)

Linear Hypotheses:
                            Estimate Std. Error z value Pr(>|z|)
EpoVsPlacebo.EpoDay13 == 0    2.2750     0.4841   4.700 1.56e-05 ***
EpoVsPlacebo.EpoDay27 == 0    2.1992     0.4925   4.466 1.59e-05 ***
EpoVsPlacebo.EpoDay41 == 0    2.1981     0.4874   4.510 1.59e-05 ***
EpoVsPlacebo.EpoDay61 == 0    1.7946     0.4901   3.661 0.000376 ***
EpoVsPlacebo.postDay62 == 0   1.4846     0.4954   2.997 0.003274 **
EpoVsPlacebo.postDay80 == 0  -0.1585     0.4874  -0.325 0.745109
---
Signif. codes:  0 ‘***’ 0.001 ‘**’ 0.01 ‘*’ 0.05 ‘.’ 0.1 ‘ ’ 1
(Adjusted p values reported -- BH method)


#############################################################
```

7575

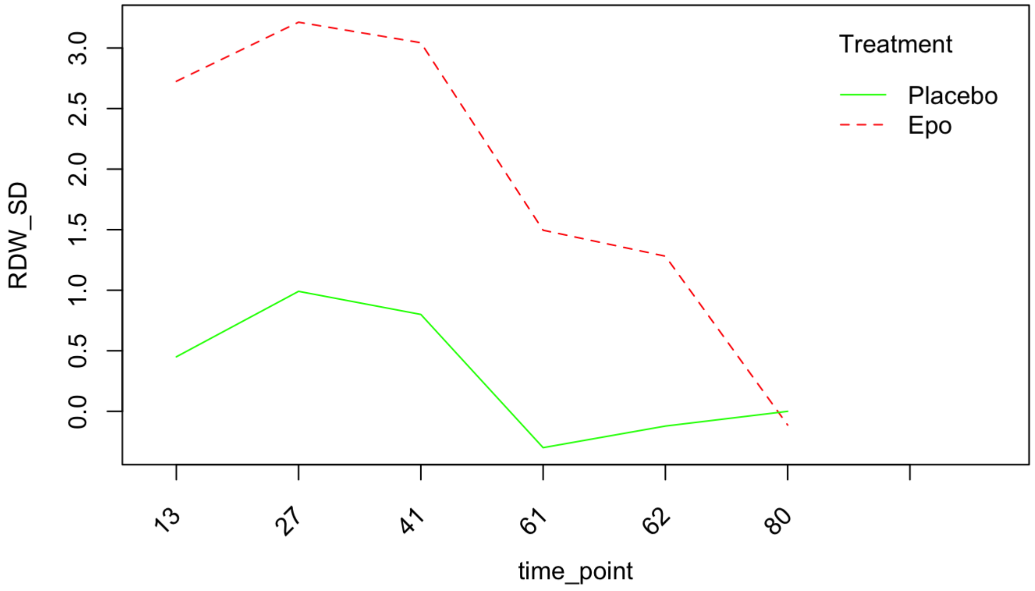


```
################################################################
########### Repeated measures analysis results for  Reticulo_per ############
#################################################################
```

	numDF <int>	denDF <dbl>	F-value <chr>	p-value <chr>
(Intercept)	1	220	0.004686	0.9455
Treatment	1	46	0.083848	0.7735
time_point	5	220	24.837678	<.0001
Treatment:time_point	5	220	30.711048	<.0001

4 rows

```
########### Compare Epo with Palcebo at each time point using contrasts ##########


################################################################

######################## Raw p-values ########################


     Simultaneous Tests for General Linear Hypotheses

Multiple Comparisons of Means: User-defined Contrasts


Fit: lme.formula(fixed = thisY ~ -1 + EpoTime, data = y, random = ~1 |
    Subject)

Linear Hypotheses:
                            Estimate Std. Error z value Pr(>|z|)
EpoVsPlacebo.EpoDay13 == 0   0.40417    0.07003   5.772 7.85e-09 ***
EpoVsPlacebo.EpoDay27 == 0   0.22739    0.07179   3.167 0.001538 **
EpoVsPlacebo.EpoDay41 == 0   0.23532    0.07064   3.331 0.000865 ***
EpoVsPlacebo.EpoDay61 == 0  -0.27589    0.07126  -3.872 0.000108 ***
EpoVsPlacebo.postDay62 == 0 -0.29188    0.07245  -4.029 5.61e-05 ***
EpoVsPlacebo.postDay80 == 0 -0.26042    0.07003  -3.719 0.000200 ***
---
Signif. codes:  0 '***' 0.001 '**' 0.01 '*' 0.05 '.' 0.1 ' ' 1
(Adjusted p values reported -- none method)

###########################################################


############## BH adjusted p-values ####################
Benjamini, Y., and Hochberg, Y. (1995). Controlling the false discovery rate: a practical and powerful approach t
o multiple testing. Journal of the Royal Statistical Society Series B, 57, 289–300.http://www.jstor.org/stable/23
46101.
##############################################################

     Simultaneous Tests for General Linear Hypotheses

Multiple Comparisons of Means: User-defined Contrasts


Fit: lme.formula(fixed = thisY ~ -1 + EpoTime, data = y, random = ~1 |
    Subject)

Linear Hypotheses:
                            Estimate Std. Error z value Pr(>|z|)
EpoVsPlacebo.EpoDay13 == 0   0.40417    0.07003   5.772 4.71e-08 ***
EpoVsPlacebo.EpoDay27 == 0   0.22739    0.07179   3.167 0.001538 **
EpoVsPlacebo.EpoDay41 == 0   0.23532    0.07064   3.331 0.001038 **
EpoVsPlacebo.EpoDay61 == 0  -0.27589    0.07126  -3.872 0.000216 ***
EpoVsPlacebo.postDay62 == 0 -0.29188    0.07245  -4.029 0.000168 ***
EpoVsPlacebo.postDay80 == 0 -0.26042    0.07003  -3.719 0.000300 ***
---
Signif. codes:  0 '***' 0.001 '**' 0.01 '*' 0.05 '.' 0.1 ' ' 1
(Adjusted p values reported -- BH method)


##############################################################
```

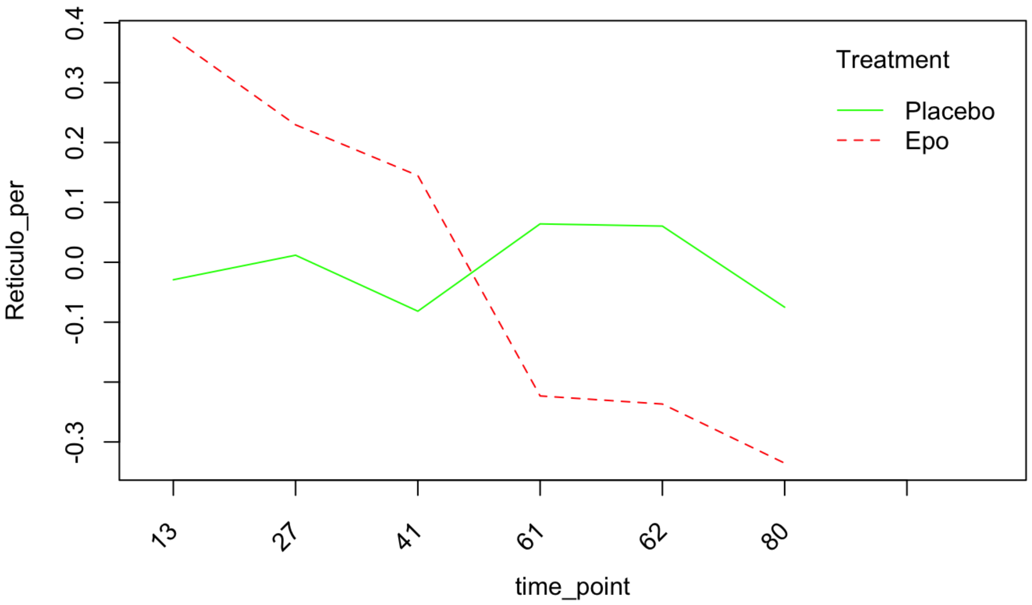

7777

```
################################################################
########### Repeated measures analysis results for  Reticulocyte ############
################################################################
```

	numDF	denDF	F-value	p-value
	<int>	<dbl>	<chr>	<chr>
(Intercept)	1	221	2.294845	0.1312
Treatment	1	46	1.017214	0.3185
time_point	5	221	17.398805	<.0001
Treatment:time_point	5	221	28.143622	<.0001

4 rows

```
############ Compare Epo with Palcebo at each time point using contrasts ###########


################################################################

######################## Raw p-values ########################


     Simultaneous Tests for General Linear Hypotheses

Multiple Comparisons of Means: User-defined Contrasts


Fit: lme.formula(fixed = thisY ~ -1 + EpoTime, data = y, random = ~1 |
    Subject)

Linear Hypotheses:
                            Estimate Std. Error z value Pr(>|z|)
EpoVsPlacebo.EpoDay13 == 0    21.098      3.398   6.209 5.33e-10 ***
EpoVsPlacebo.EpoDay27 == 0    14.097      3.462   4.071 4.67e-05 ***
EpoVsPlacebo.EpoDay41 == 0    13.324      3.431   3.883 0.000103 ***
EpoVsPlacebo.EpoDay61 == 0   -12.174      3.466  -3.513 0.000444 ***
EpoVsPlacebo.postDay62 == 0  -12.491      3.533  -3.535 0.000407 ***
EpoVsPlacebo.postDay80 == 0  -13.052      3.398  -3.841 0.000122 ***
---
Signif. codes:  0 ‘***’ 0.001 ‘**’ 0.01 ‘*’ 0.05 ‘.’ 0.1 ‘ ’ 1
(Adjusted p values reported -- none method)

###########################################################


############## BH adjusted p-values #####################
Benjamini, Y., and Hochberg, Y. (1995). Controlling the false discovery rate: a practical and powerful approach t
o multiple testing. Journal of the Royal Statistical Society Series B, 57, 289–300.http://www.jstor.org/stable/23
46101.
###############################################################

     Simultaneous Tests for General Linear Hypotheses

Multiple Comparisons of Means: User-defined Contrasts


Fit: lme.formula(fixed = thisY ~ -1 + EpoTime, data = y, random = ~1 |
    Subject)

Linear Hypotheses:
                            Estimate Std. Error z value Pr(>|z|)
EpoVsPlacebo.EpoDay13 == 0    21.098      3.398   6.209  3.2e-09 ***
EpoVsPlacebo.EpoDay27 == 0    14.097      3.462   4.071 0.000140 ***
EpoVsPlacebo.EpoDay41 == 0    13.324      3.431   3.883 0.000184 ***
EpoVsPlacebo.EpoDay61 == 0   -12.174      3.466  -3.513 0.000444 ***
EpoVsPlacebo.postDay62 == 0  -12.491      3.533  -3.535 0.000444 ***
EpoVsPlacebo.postDay80 == 0  -13.052      3.398  -3.841 0.000184 ***
---
Signif. codes:  0 ‘***’ 0.001 ‘**’ 0.01 ‘*’ 0.05 ‘.’ 0.1 ‘ ’ 1
(Adjusted p values reported -- BH method)


##############################################################
```

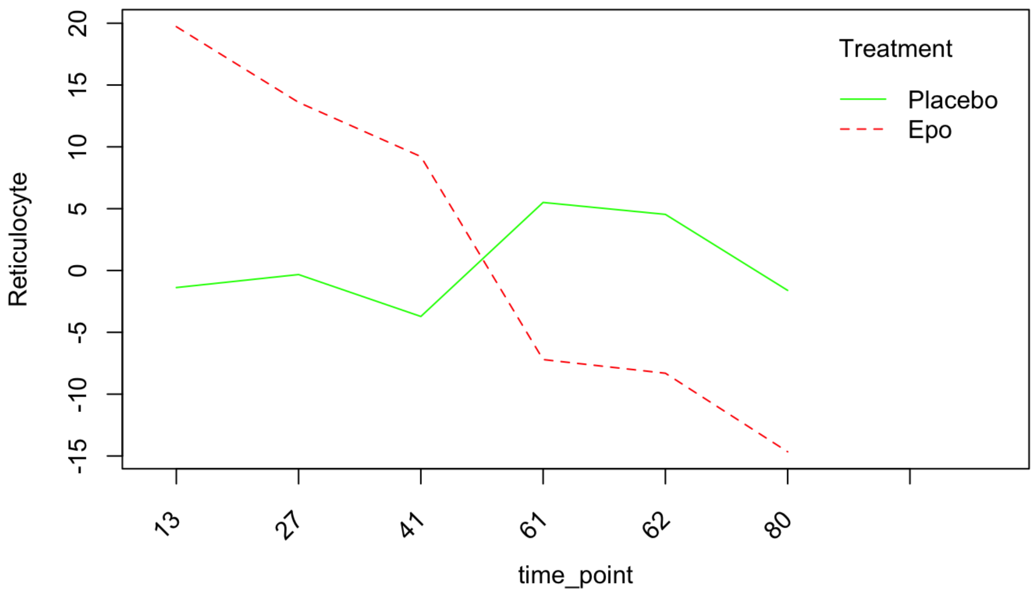


```
##############################################################
########### Repeated measures analysis results for  Thrombocytes ############
###############################################################
```

	numDF	denDF	F-value	p-value
	<int>	<dbl>	<chr>	<chr>
(Intercept)	1	221	8.443376	0.0040
Treatment	1	46	0.000478	0.9827
time_point	5	221	6.081569	<.0001
Treatment:time_point	5	221	2.263850	0.0492

4 rows

7979

```
########### Compare Epo with Palcebo at each time point using contrasts ###########


###############################################################

####################### Raw p-values #######################


     Simultaneous Tests for General Linear Hypotheses

Multiple Comparisons of Means: User-defined Contrasts


Fit: lme.formula(fixed = thisY ~ -1 + EpoTime, data = y, random = ~1 |
    Subject)

Linear Hypotheses:
                            Estimate Std. Error z value Pr(>|z|)
EpoVsPlacebo.EpoDay13 == 0     8.687      7.673   1.132    0.258
EpoVsPlacebo.EpoDay27 == 0    12.476      7.814   1.597    0.110
EpoVsPlacebo.EpoDay41 == 0     4.498      7.746   0.581    0.561
EpoVsPlacebo.EpoDay61 == 0   -11.817      7.821  -1.511    0.131
EpoVsPlacebo.postDay62 == 0   -9.326      7.968  -1.170    0.242
EpoVsPlacebo.postDay80 == 0   -5.396      7.673  -0.703    0.482
(Adjusted p values reported -- none method)

##########################################################


############# BH adjusted p-values ###################
Benjamini, Y., and Hochberg, Y. (1995). Controlling the false discovery rate: a practical and powerful approach t
o multiple testing. Journal of the Royal Statistical Society Series B, 57, 289–300.http://www.jstor.org/stable/23
46101.
##############################################################

     Simultaneous Tests for General Linear Hypotheses

Multiple Comparisons of Means: User-defined Contrasts


Fit: lme.formula(fixed = thisY ~ -1 + EpoTime, data = y, random = ~1 |
    Subject)

Linear Hypotheses:
                            Estimate Std. Error z value Pr(>|z|)
EpoVsPlacebo.EpoDay13 == 0     8.687      7.673   1.132    0.386
EpoVsPlacebo.EpoDay27 == 0    12.476      7.814   1.597    0.386
EpoVsPlacebo.EpoDay41 == 0     4.498      7.746   0.581    0.561
EpoVsPlacebo.EpoDay61 == 0   -11.817      7.821  -1.511    0.386
EpoVsPlacebo.postDay62 == 0   -9.326      7.968  -1.170    0.386
EpoVsPlacebo.postDay80 == 0   -5.396      7.673  -0.703    0.561
(Adjusted p values reported -- BH method)
```

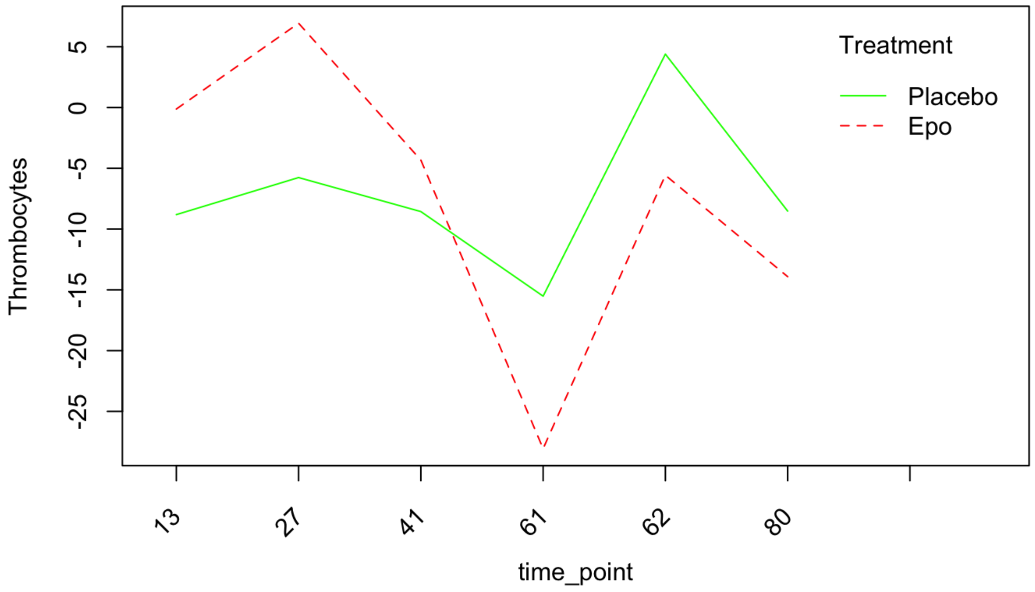

Data visualization

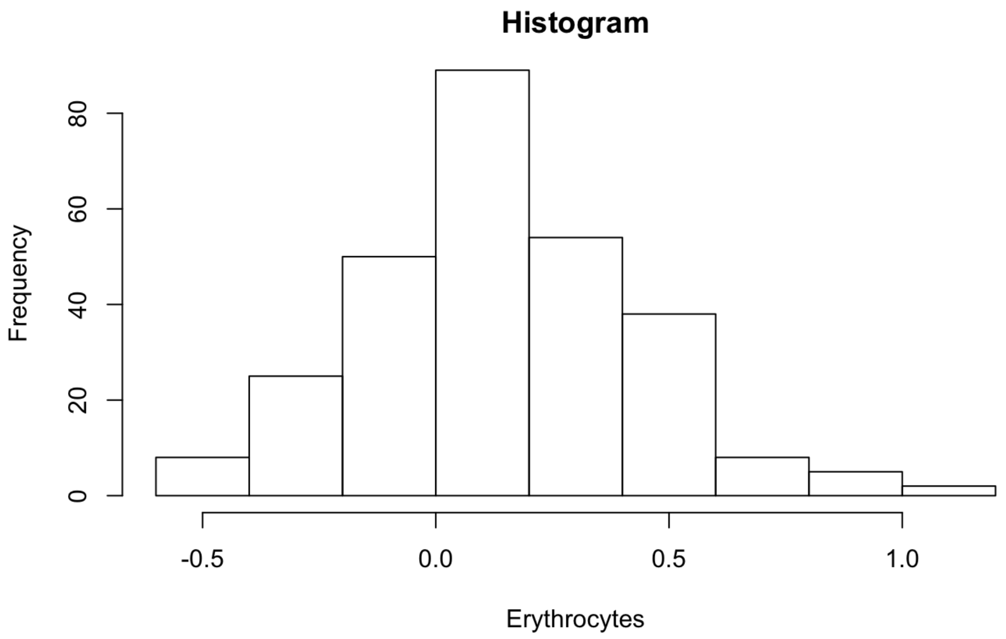
Histogram
Frequency
0
20
40
60
80
-0.5
0.0
0.5
1.0
Erythrocytes

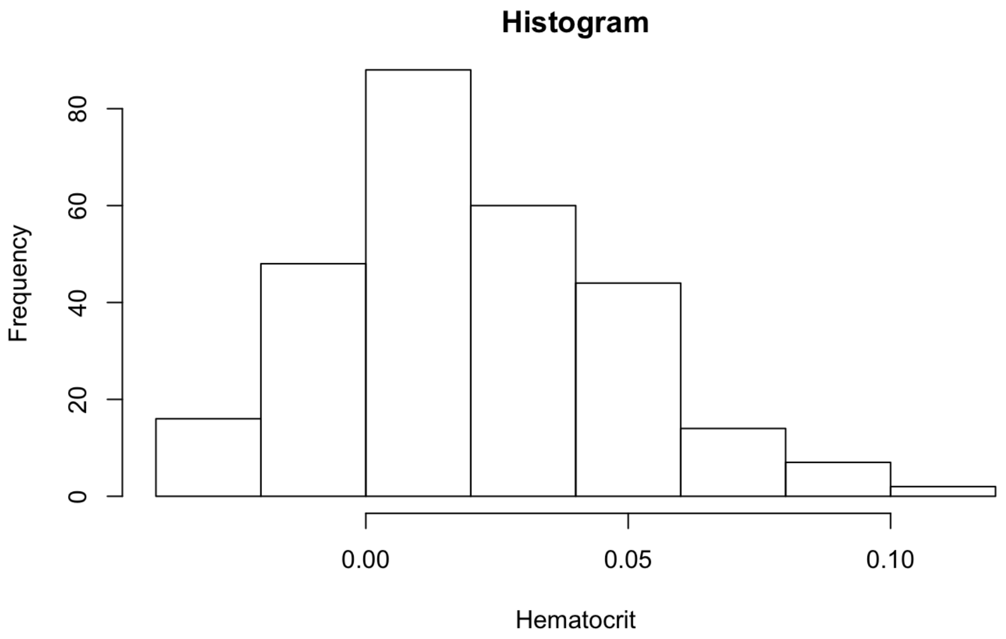
Histogram
Frequency
0
20
40
60
80
0.00
0.05
0.10
Hematocrit

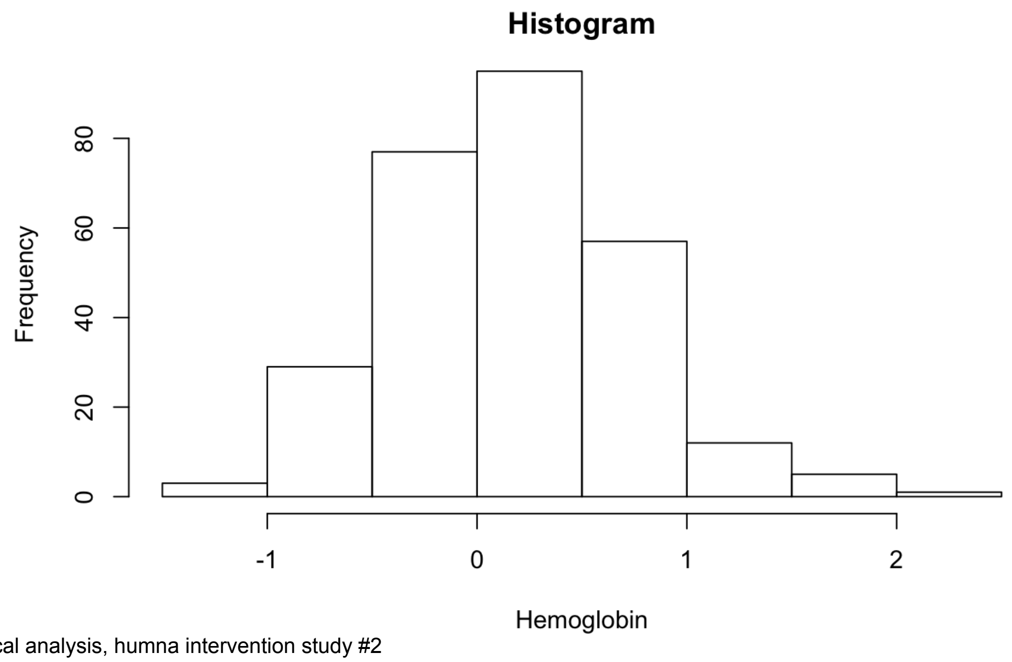
Histogram
Frequency
0
20
40
60
80
-1
0
1
2
Hemoglobin

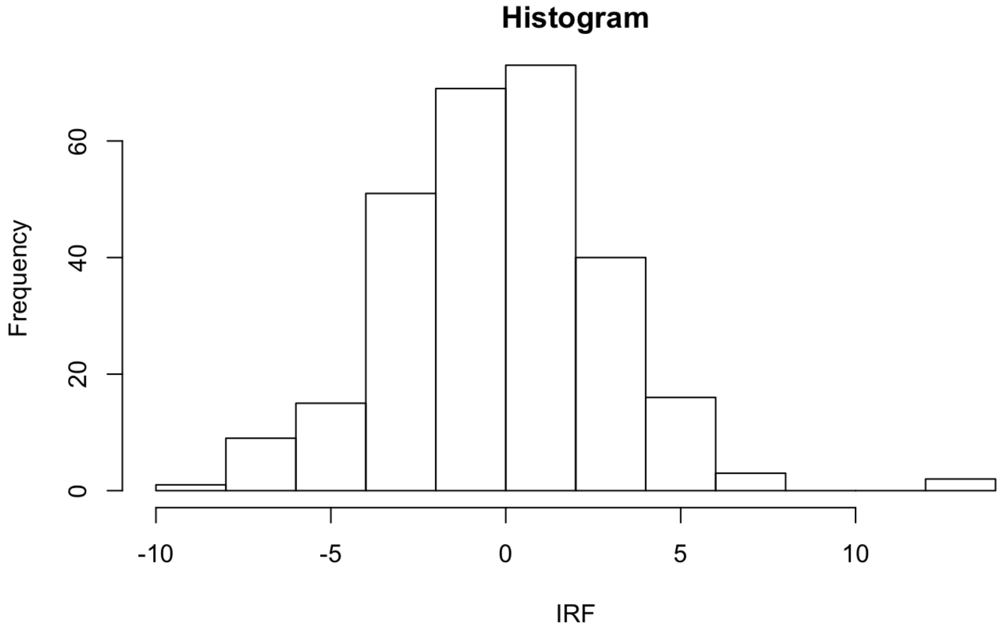
Histogram
Frequency
0
20
40
60
-10
-5
0
5
10
IRF

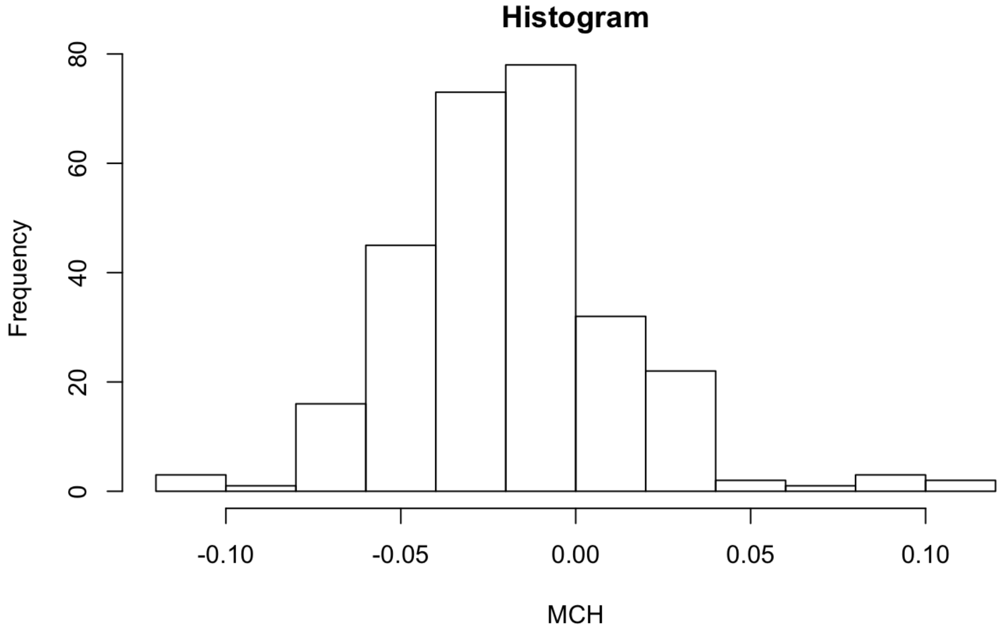
Histogram
Frequency
0
20
40
60
80
-0.10
-0.05
0.00
0.05
0.10
MCH

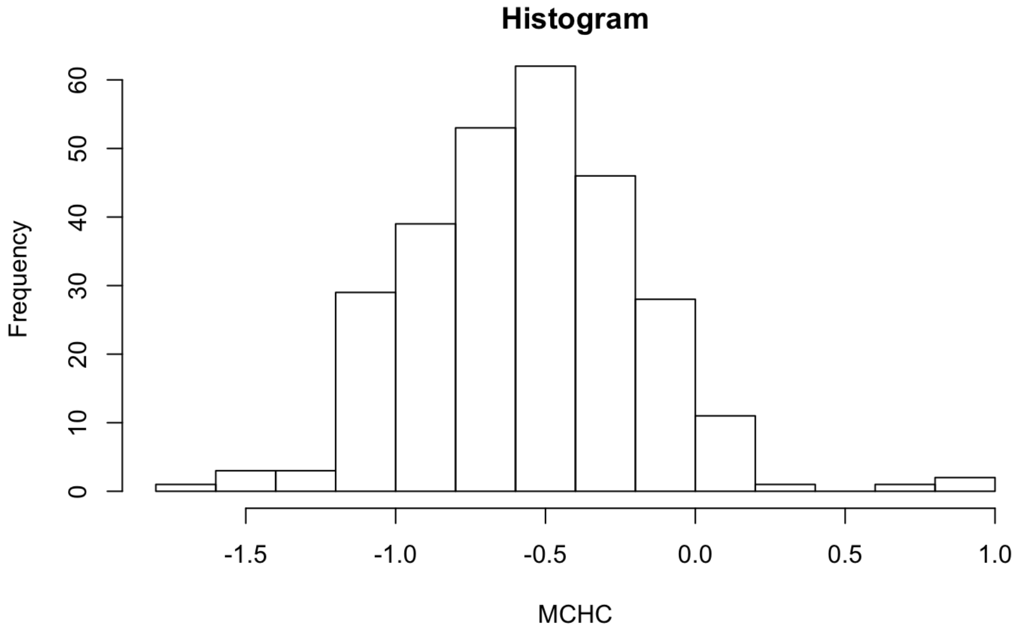
Histogram
Frequency
0
10
20
30
40
50
60
-1.5
-1.0
-0.5
0.0
0.5
1.0
MCHC

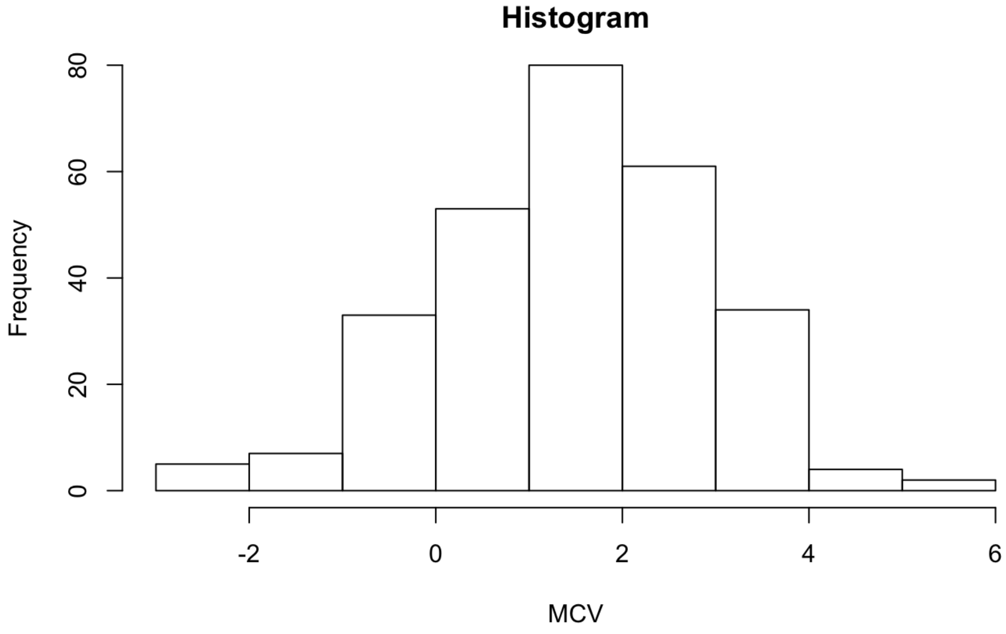
Histogram
Frequency
0
20
40
60
80
-2
0
2
4
6
MCV

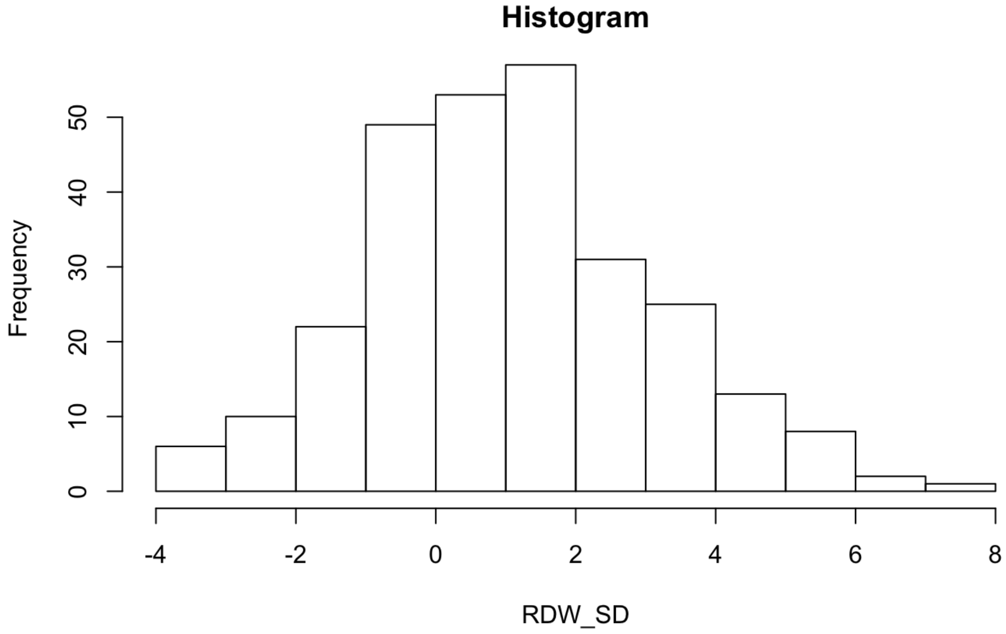
Histogram
Frequency
0
10
20
30
40
50
-4
-2
0
2
4
6
8
RDW_SD

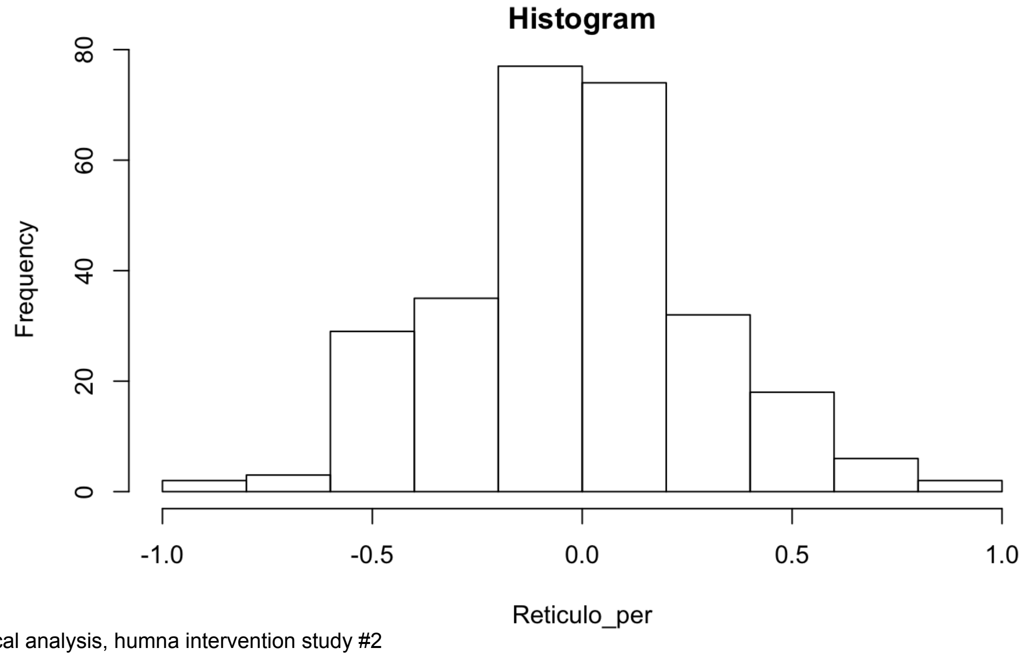
Histogram
Frequency
0
20
40
60
80
-1.0
-0.5
0.0
0.5
1.0
Reticulo_per

8383

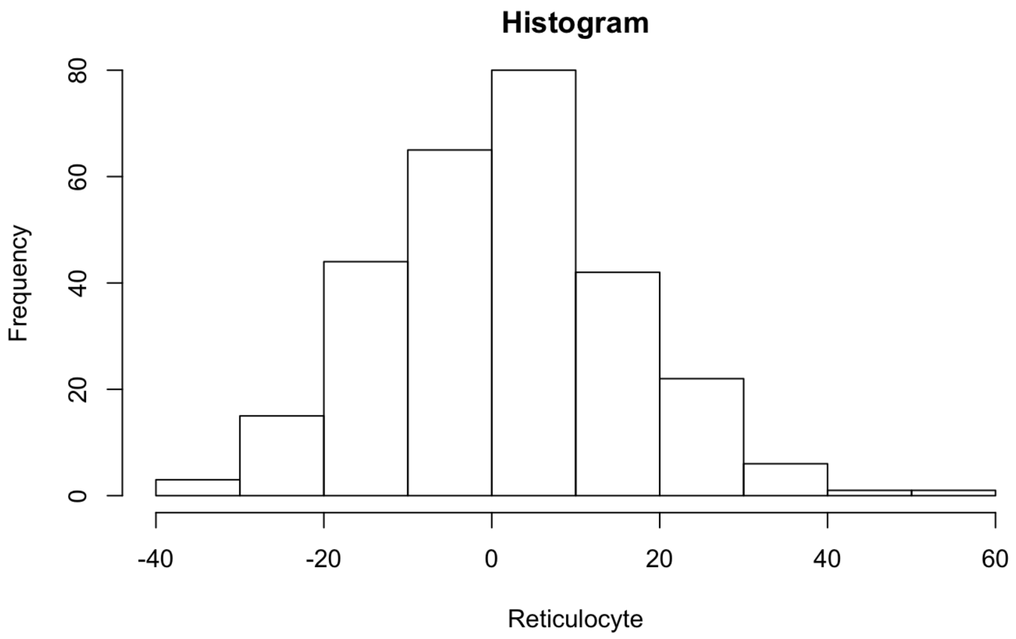
Histogram
Frequency
0
20
40
60
80
-40
-20
0
20
40
60
Reticulocyte

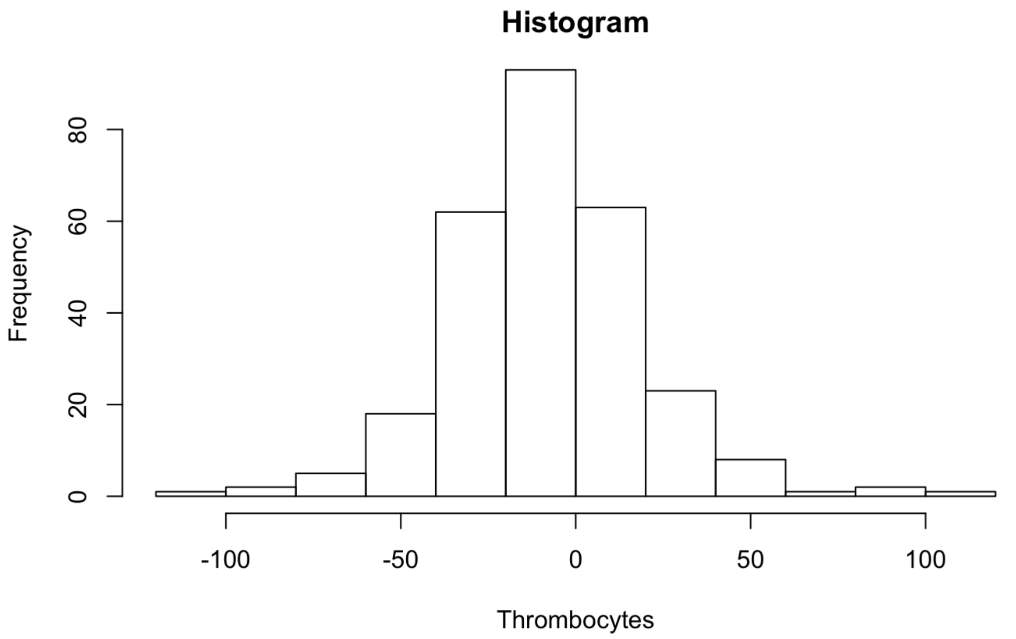
Histogram
Frequency
0
20
40
60
80
-100
-50
0
50
100
Thrombocytes

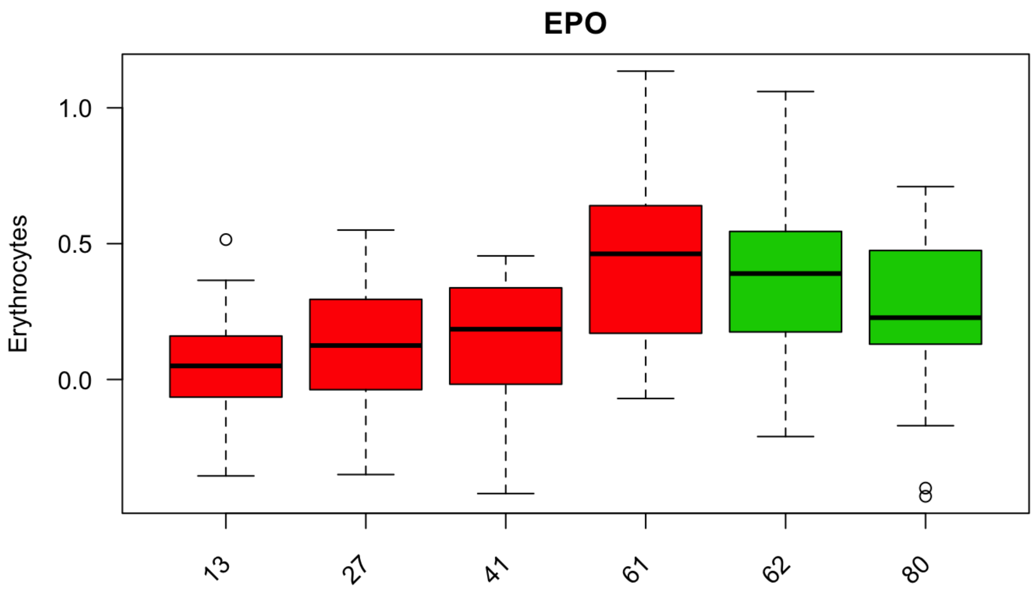
EPO
Erythrocytes
0.0
0.5
1.0
13
27
41
61
62
80

8484

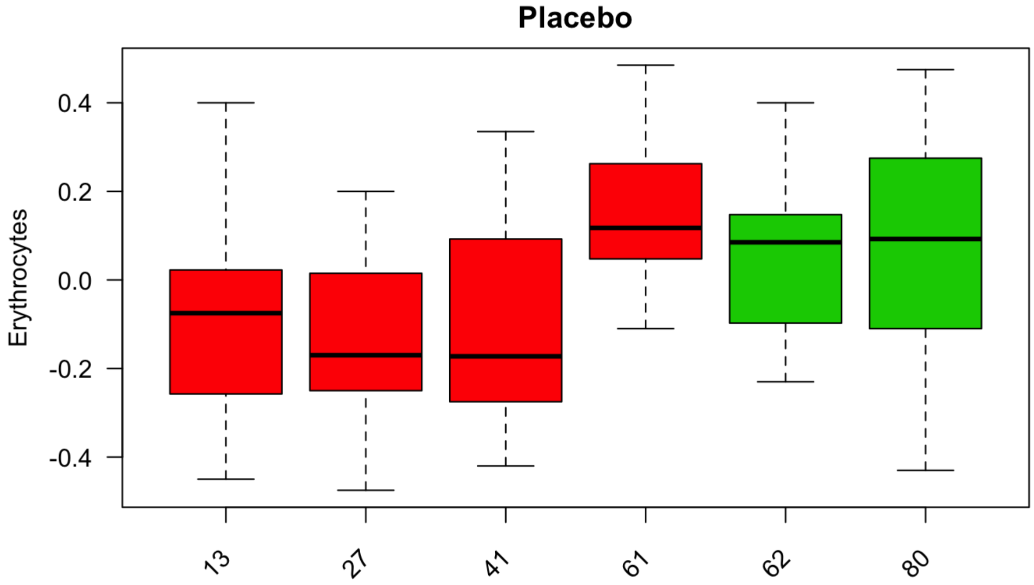
Placebo
Erythrocytes
0.4
0.2
0.0
-0.2
-0.4
13
27
41
61
62
80

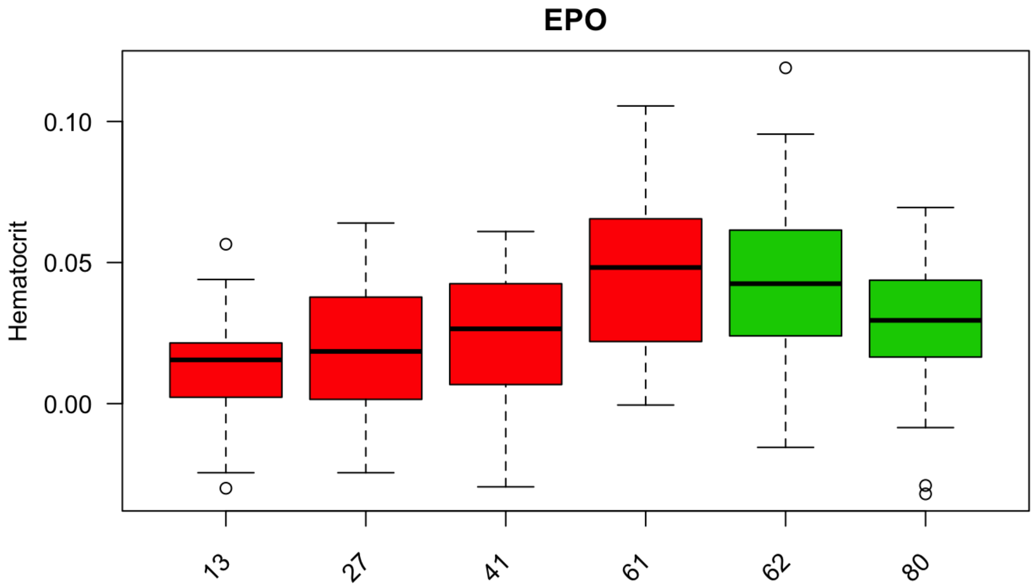
EPO
Hematocrit
0.10
0.05
0.00
13
27
41
61
62
80

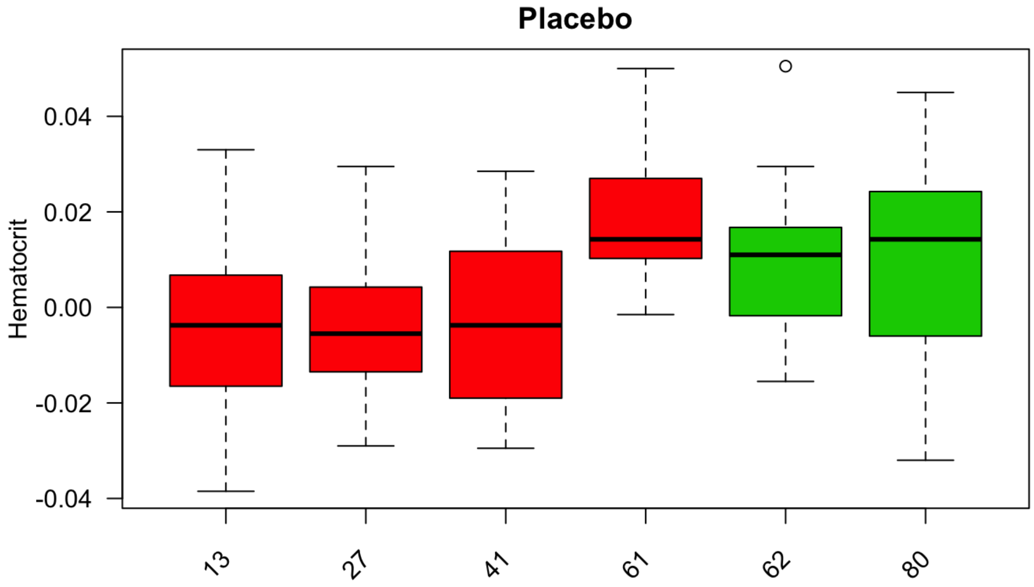
Placebo
Hematocrit
0.04
0.02
0.00
-0.02
-0.04
13
27
41
61
62
80

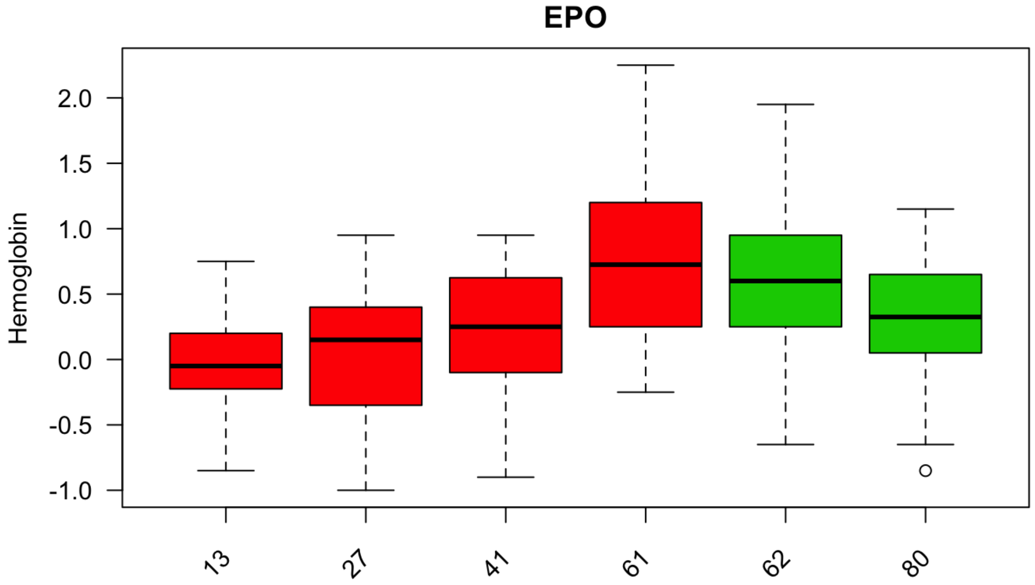
EPO
Hemoglobin
2.0
1.5
1.0
0.5
0.0
-0.5
-1.0
13
27
41
61
62
80

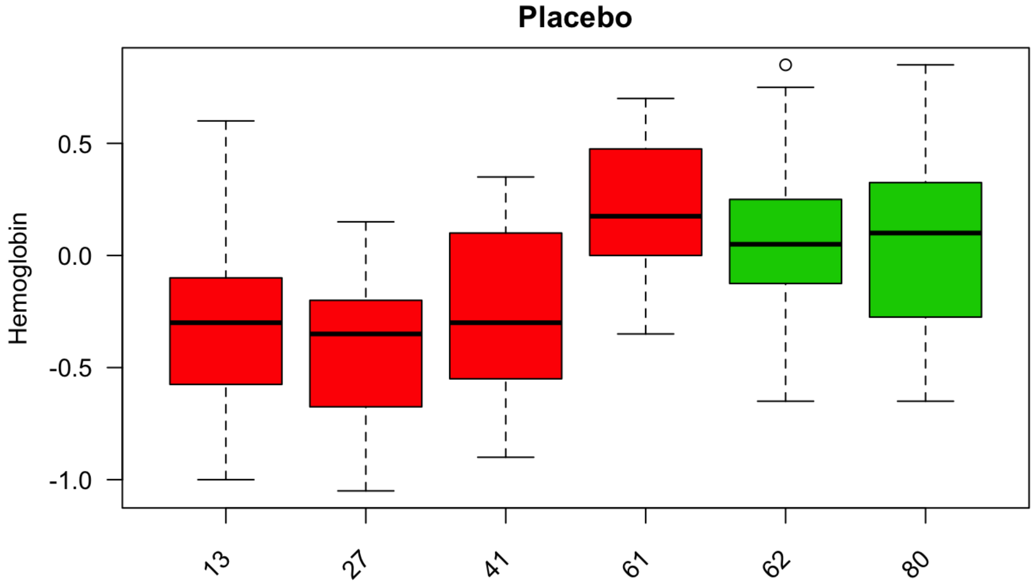
Placebo
Hemoglobin
0.5
0.0
-0.5
-1.0
13
27
41
61
62
80

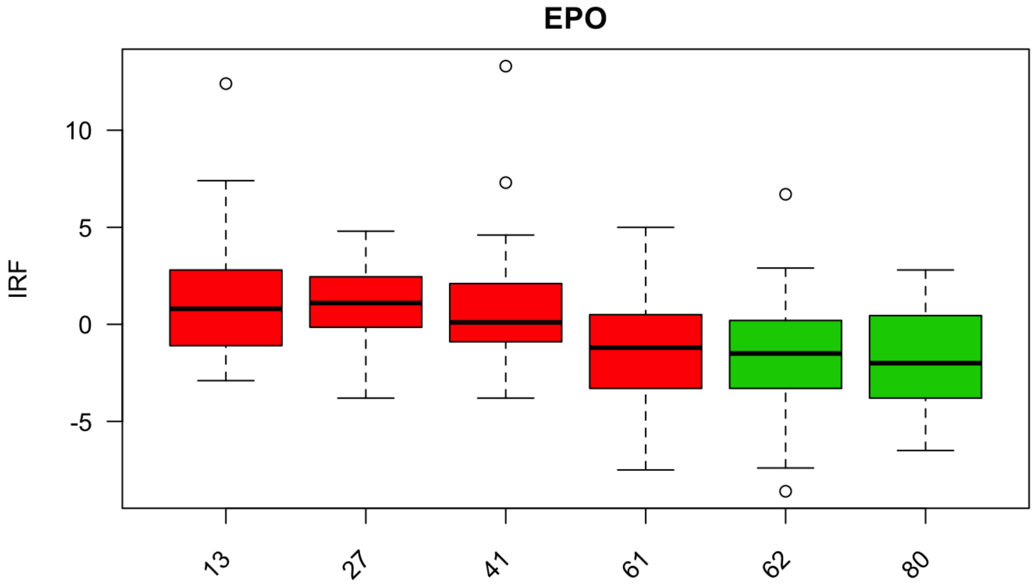
EPO
IRF
10
5
0
-5
13
27
41
61
62
80

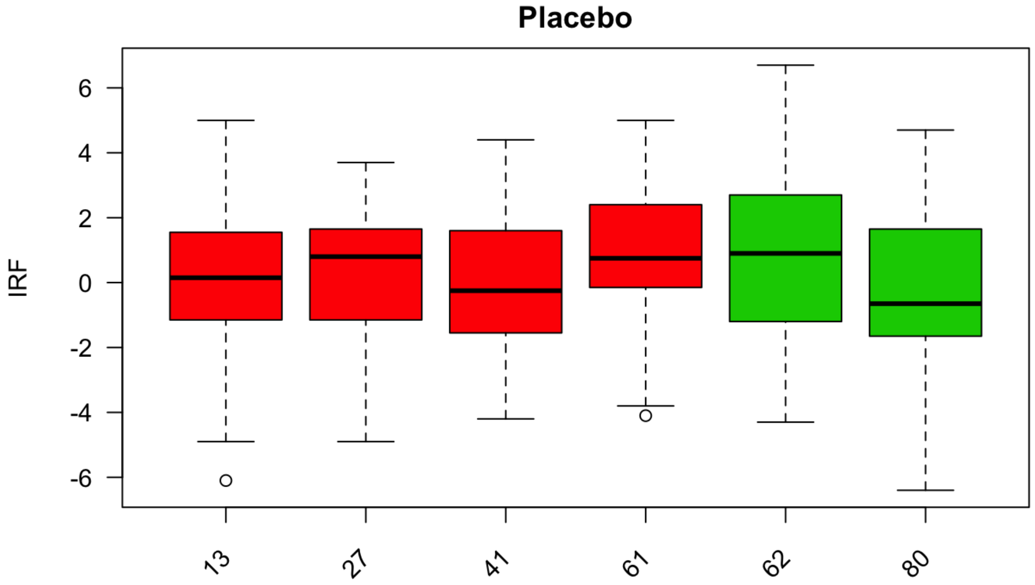
Placebo
IRF
6
4
2
0
-2
-4
-6
13
27
41
61
62
80

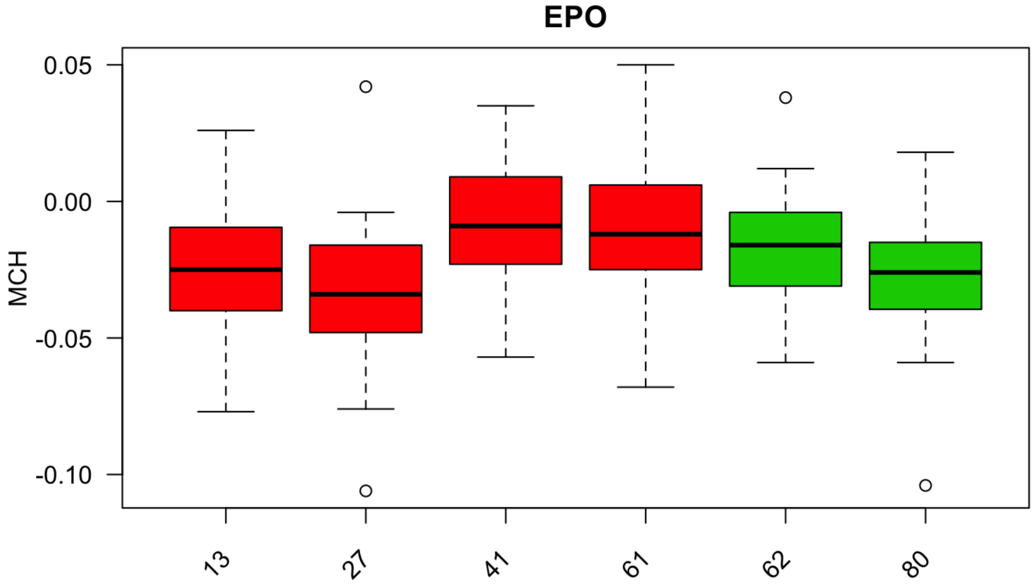
EPO
MCH
0.05
0.00
-0.05
-0.10
13
27
41
61
62
80

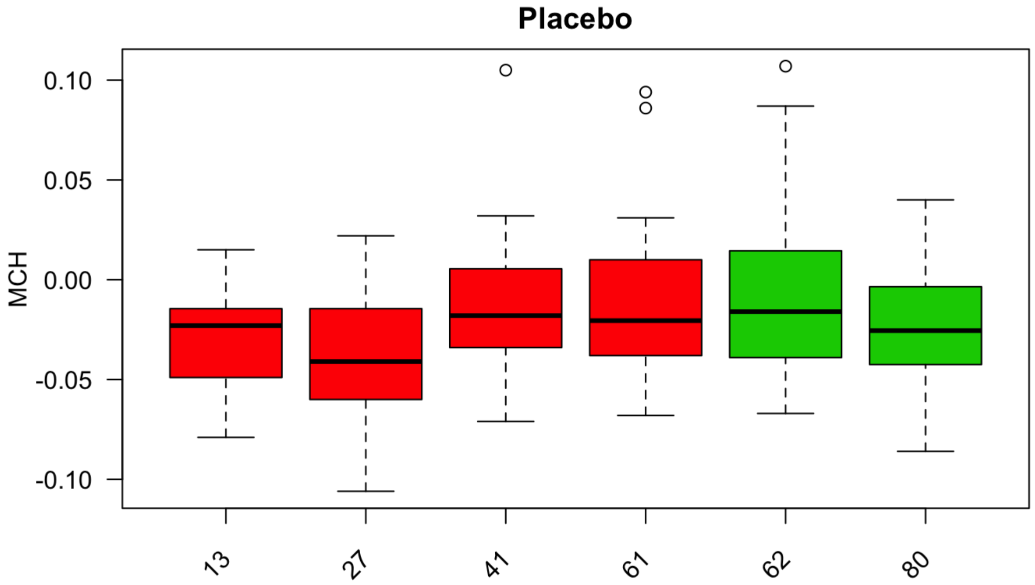
Placebo
MCH
0.10
0.05
0.00
-0.05
-0.10
13
27
41
61
62
80

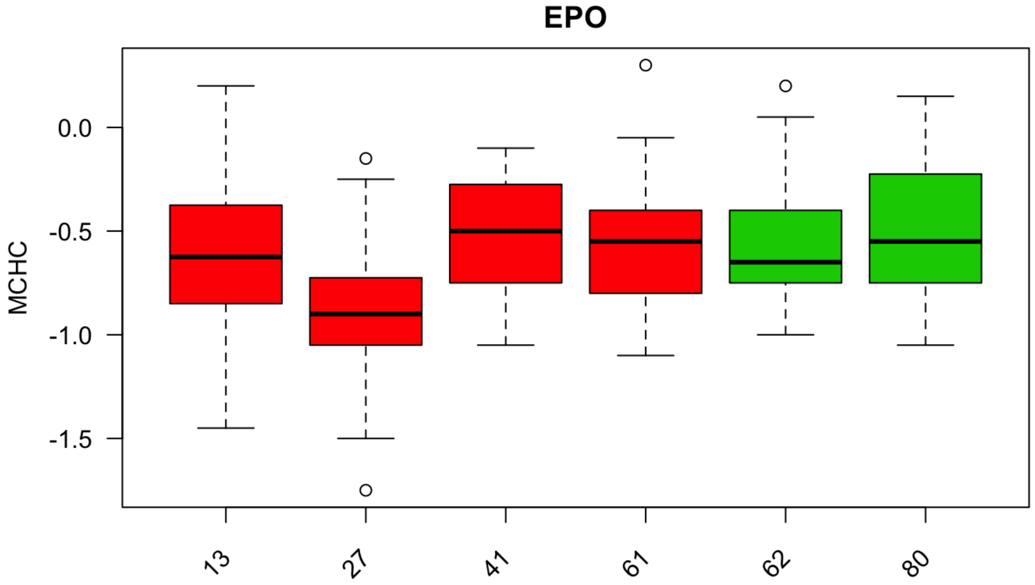
EPO
MCHC
0.0
-0.5
-1.0
-1.5
13
27
41
61
62
80

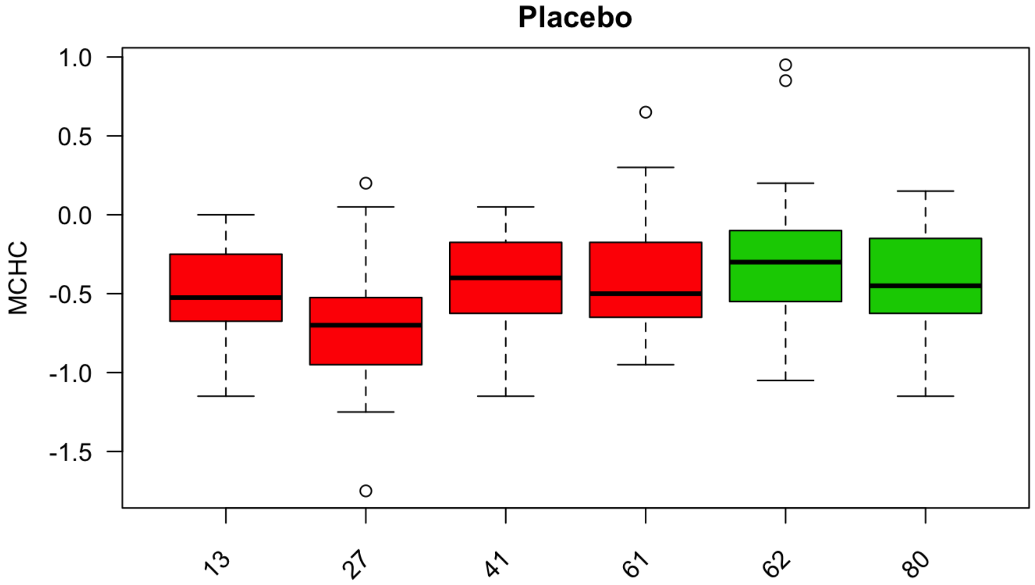
Placebo
MCHC
1.0
0.5
0.0
-0.5
-1.0
-1.5
13
27
41
61
62
80

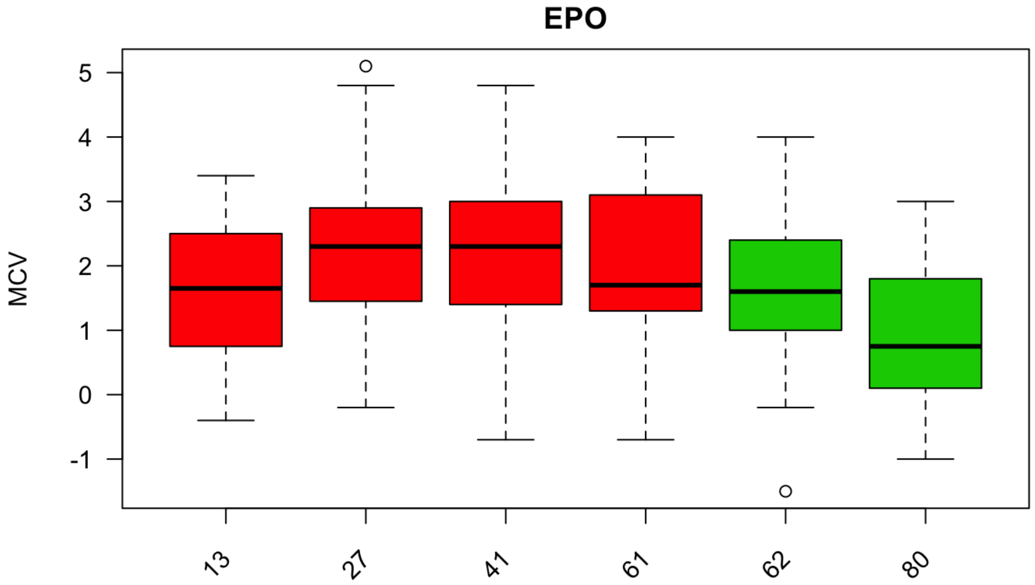
EPO
MCV
5
4
3
2
1
0
-1
13
27
41
61
62
80

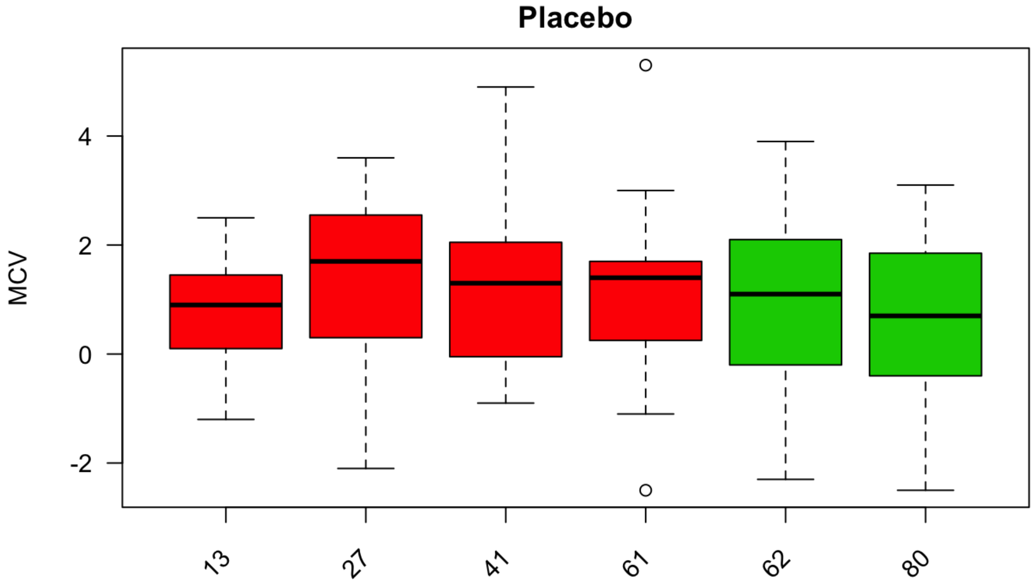
Placebo
MCV
4
2
0
-2
13
27
41
61
62
80

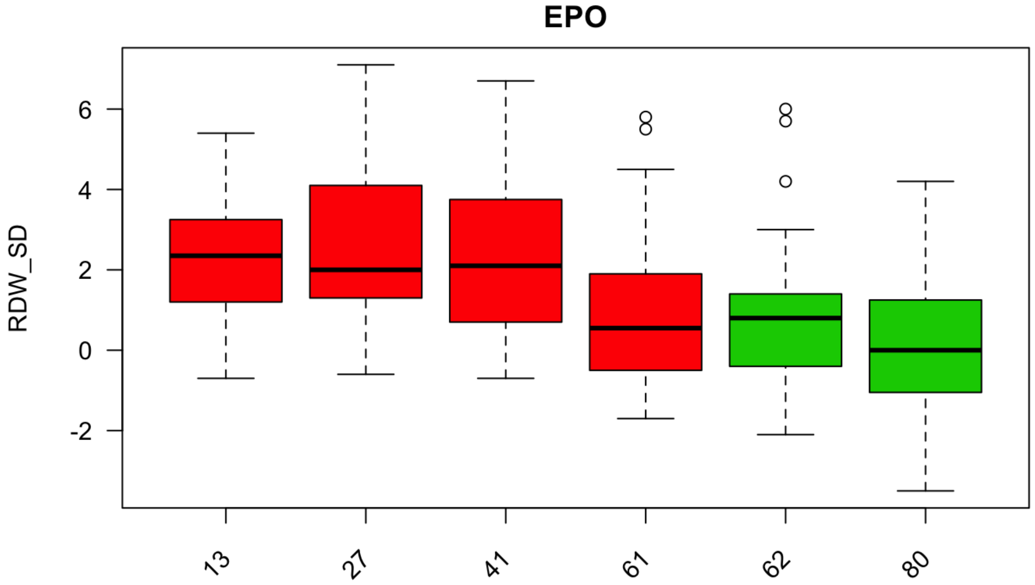
EPO
RDW_SD
6
4
2
0
-2
13
27
41
61
62
80

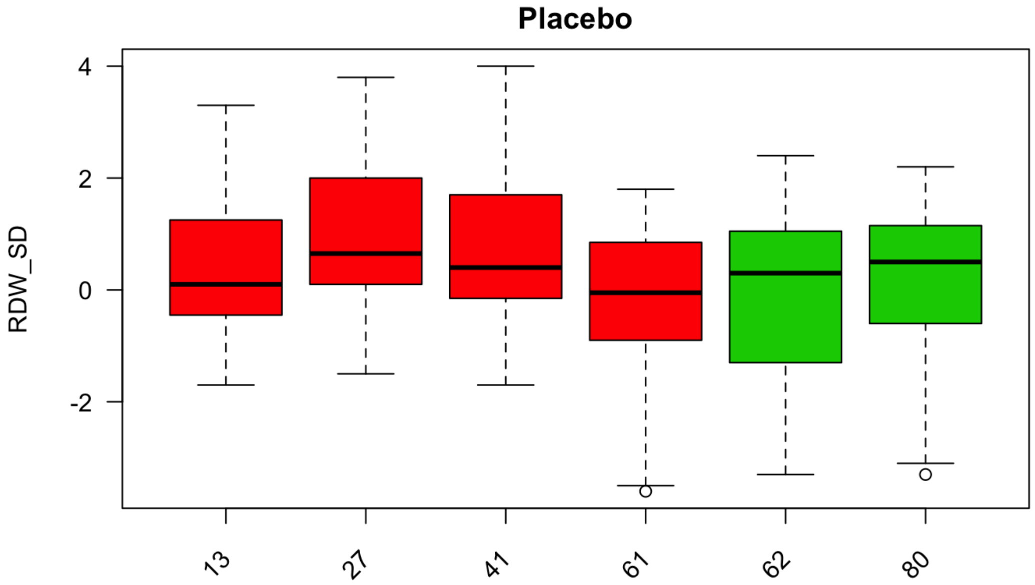
Placebo
RDW_SD
4
2
0
-2
13
27
41
61
62
80

8989

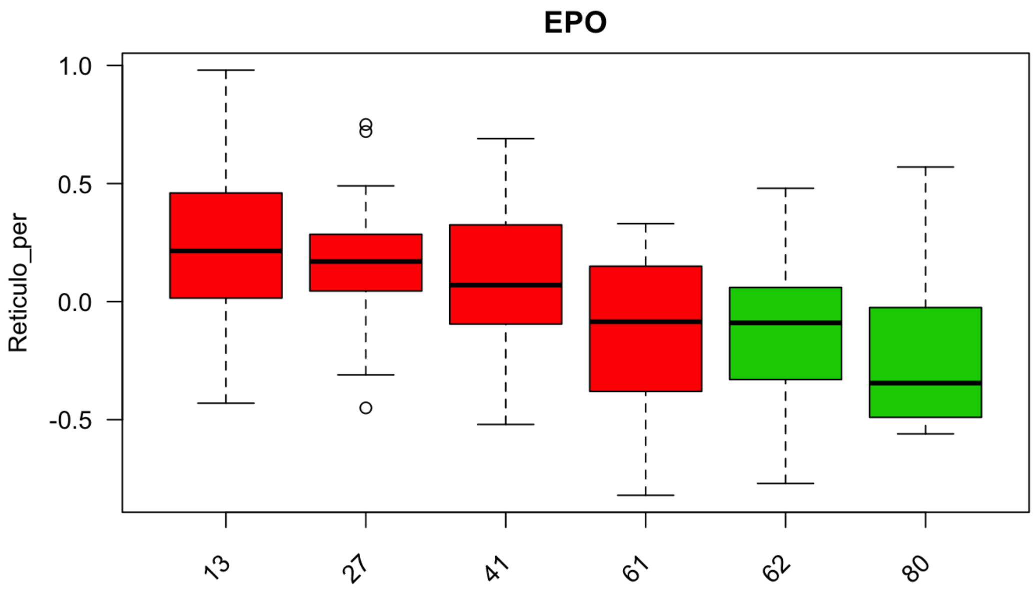
EPO
Reticulo_per
1.0
0.5
0.0
-0.5
13
27
41
61
62
80

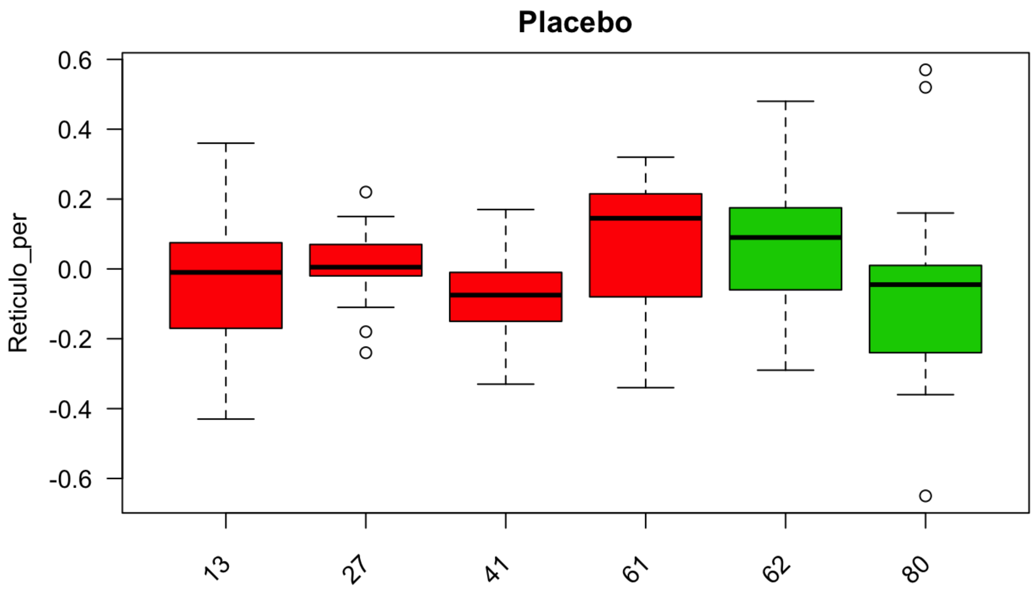
Placebo
Reticulo_per
0.6
0.4
0.2
0.0
-0.2
-0.4
-0.6
13
27
41
61
62
80

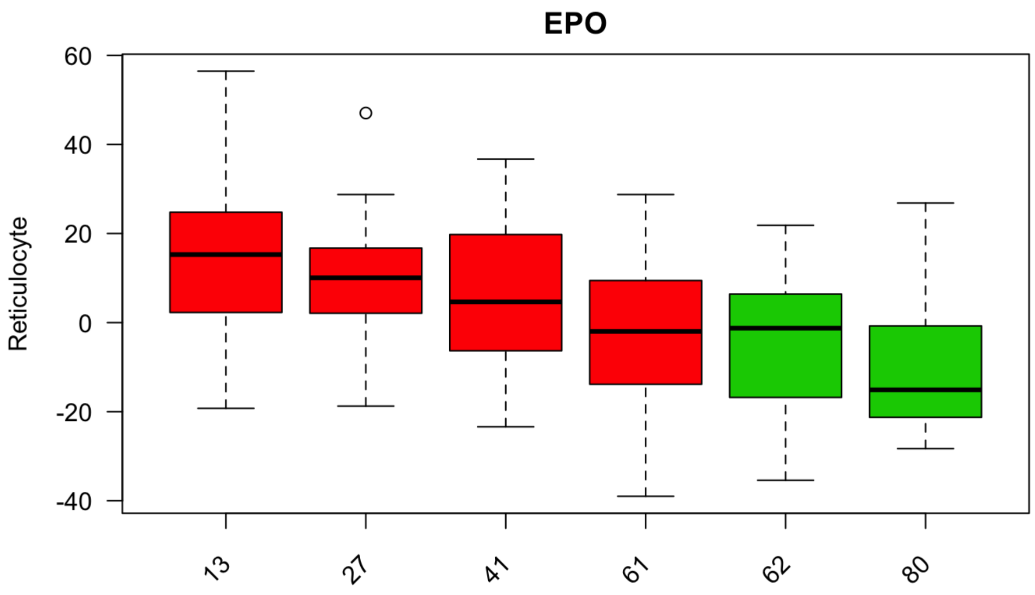
EPO
Reticulocyte
60
40
20
0
-20
-40
13
27
41
61
62
80

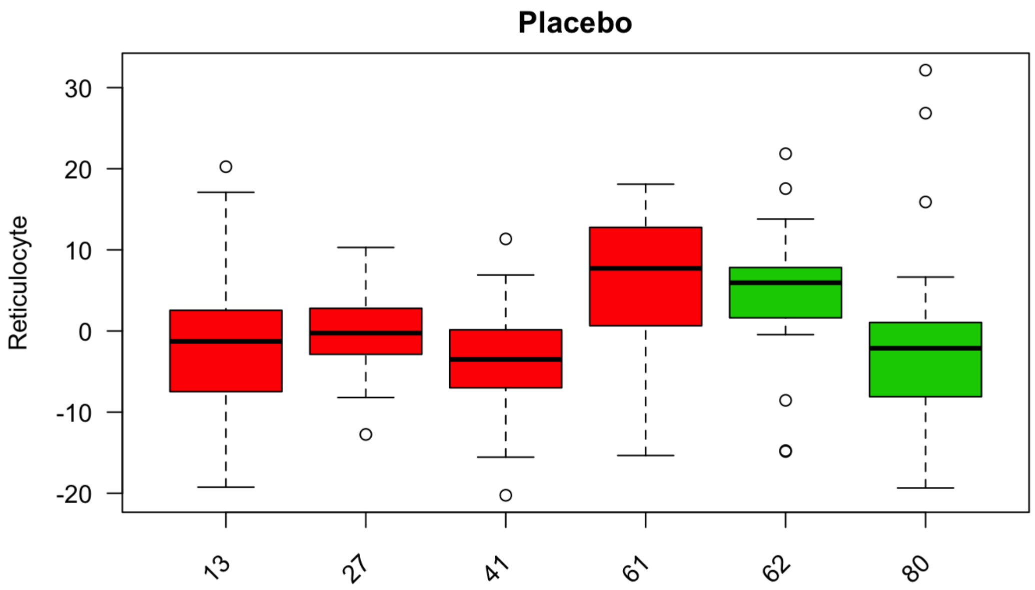
Placebo
Reticulocyte
30
20
10
0
-10
-20
13
27
41
61
62
80

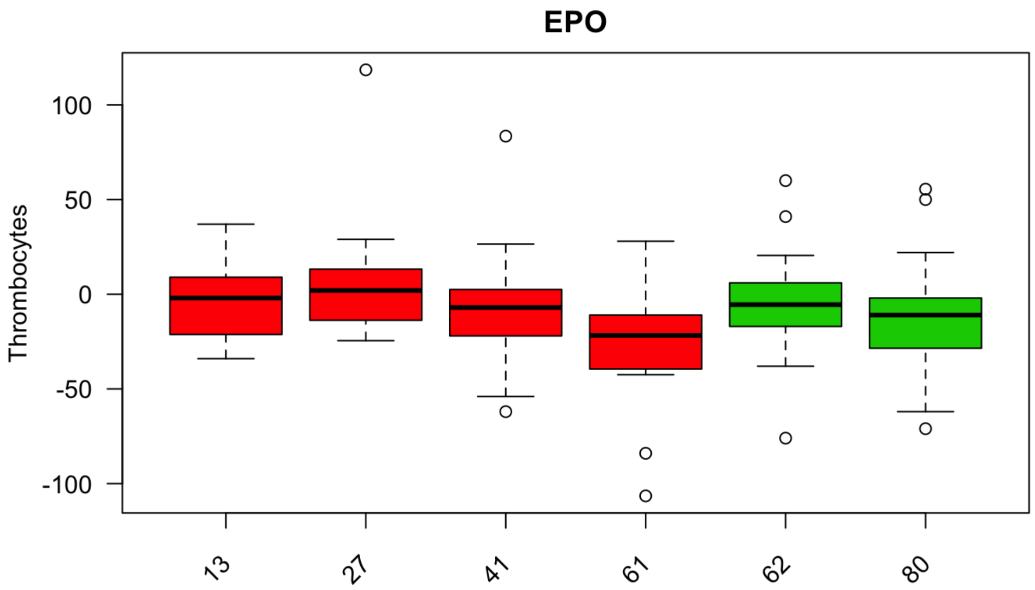
EPO
Thrombocytes
100
50
0
-50
-100
13
27
41
61
62
80

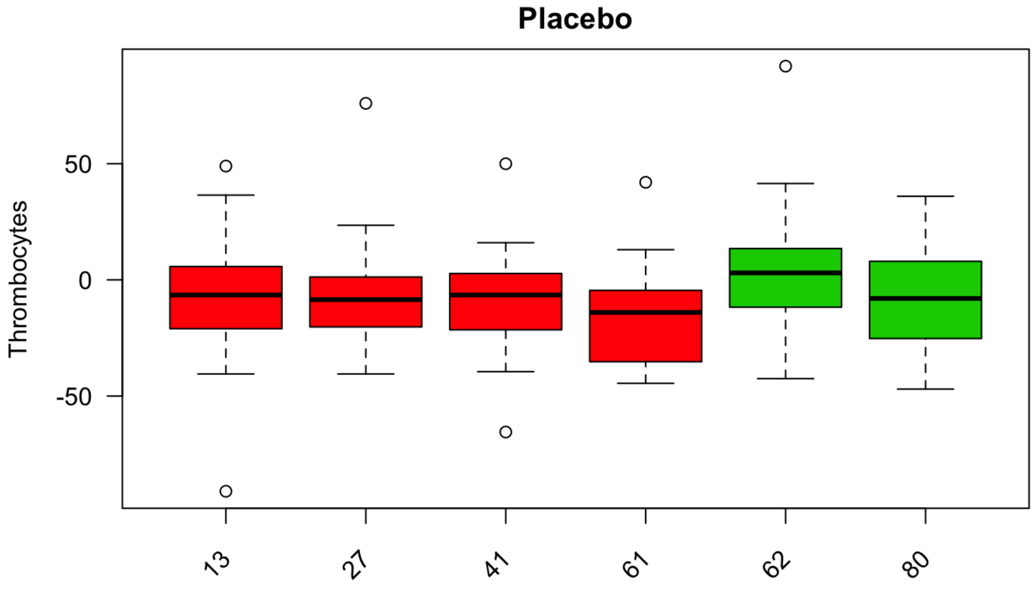
Placebo
Thrombocytes
50
0
-50
13
27
41
61
62
80

9191

```
R version 3.6.1 Patched (2019-09-23 r77210)
Platform: x86_64-apple-darwin15.6.0 (64-bit)
Running under: macOS Mojave 10.14.6

Matrix products: default
BLAS:   /System/Library/Frameworks/Accelerate.framework/Versions/A/Frameworks/vecLib.framework/Versions/A/libBLA
S.dylib
LAPACK: /Library/Frameworks/R.framework/Versions/3.6/Resources/lib/libRlapack.dylib

locale:
[1] en_US.UTF-8/en_US.UTF-8/en_US.UTF-8/C/en_US.UTF-8/en_US.UTF-8

attached base packages:
[1] stats     graphics  grDevices utils     datasets  methods   base

other attached packages:
[1] multcomp_1.4-12 TH.data_1.0-10  MASS_7.3-51.5   survival_3.1-8  mvtnorm_1.0-12  nlme_3.1-143

loaded via a namespace (and not attached):
 [1] Rcpp_1.0.3       knitr_1.27       magrittr_1.5     splines_3.6.1    lattice_0.20-38
 [6] rlang_0.4.2      stringr_1.4.0    tools_3.6.1      grid_3.6.1       xfun_0.12
[11] htmltools_0.4.0  yaml_2.2.0       digest_0.6.23    Matrix_1.2-18    base64enc_0.1-3
[16] codetools_0.2-16 rsconnect_0.8.16 evaluate_0.14    rmarkdown_2.1    sandwich_2.5-1
[21] stringi_1.4.5    compiler_3.6.1   jsonlite_1.6     zoo_1.8-7
```

9292

Data analysis of phlebotomy intervention study # 3, Hidalgo et al.

Code

By Lihua Julie Zhu

Citation:Lindstrom, M.J. and Bates, D.M. (1988) “Newton-Raphson and EM Algorithms for Linear Mixed-Effects Models for Repeated-Measures Data”, Journal of the American Statistical Association, 83, 1014–1022.

Data summary

```
[1] 201   9
```

	Treatment	Subject	time_point	Hb..g.dL.	MCH..pg.	MCHC..g.dL.	MCV..fl.	Retic....	Retic.count
	<fctr>	<int>	<fctr>	<dbl>	<dbl>	<dbl>	<dbl>	<dbl>	<dbl>
1	Phlebotomy	20	Baseline 1	14.67375	30.61060	33.37875	90	0.8606557	42
2	Phlebotomy	20	Baseline 2	14.35125	30.61060	33.70125	90	1.0403397	49
3	Phlebotomy	20	3 days	12.41625	30.61060	33.37875	92	1.4285714	58
4	Phlebotomy	20	14 days	12.73875	30.61060	33.37875	91	2.1615202	91
5	Phlebotomy	20	25 days	14.35125	30.61060	33.86250	90	1.7094017	80
6	Phlebotomy	21	Baseline 1	16.28625	28.99952	35.63625	81	0.9769094	55

6 rows

	Subject	Round.1	Round.2
	<int>	<fctr>	<fctr>
1	20	Transfusion	Placebo
2	21	Placebo	Transfusion
3	22	Placebo	Transfusion
4	23	Placebo	Transfusion
5	24	Transfusion	Placebo
6	25	Transfusion	Placebo
7	26	Placebo	Transfusion
8	27	Transfusion	Placebo
9	28	Placebo	Transfusion
10	29	Placebo	Transfusion

1-10 of 21 rows Previous **1** 2 3 Next

```
[1] Transfusion Placebo
Levels:  Placebo Transfusion
[1] Placebo          Transfusion      DID NOT COMPLETE
Levels:  DID NOT COMPLETE Placebo Transfusion
, , time_point = 14 days

            Subject
Treatment    1 2 3 4 5 6 7 8 9 20 21 22 23 24 25 26 27 28 29 30 31
  Phlebotomy 1 1 1 1 1 1 1 0 1  1  1  1  1  1  1  1  1  1  1  1  1
  placebo    1 1 1 1 0 1 1 1 1  1  1  1  1  1  1  1  1  1  1  1  1

, , time_point = 25 days

            Subject
Treatment    1 2 3 4 5 6 7 8 9 20 21 22 23 24 25 26 27 28 29 30 31
  Phlebotomy 1 1 1 1 1 1 1 0 1  1  1  1  1  1  1  1  1  1  1  1  1
  placebo    0 1 1 1 1 1 1 1 1  1  1  1  1  1  1  1  1  1  1  1  1

, , time_point = 3 days

            Subject
Treatment    1 2 3 4 5 6 7 8 9 20 21 22 23 24 25 26 27 28 29 30 31
  Phlebotomy 1 1 1 1 1 1 1 0 1  1  1  1  1  1  1  1  1  1  1  1  1
  placebo    1 1 1 1 1 1 1 1 1  1  1  1  1  1  1  1  1  1  1  1  1

, , time_point = Baseline 1

            Subject
Treatment    1 2 3 4 5 6 7 8 9 20 21 22 23 24 25 26 27 28 29 30 31
  Phlebotomy 1 1 1 1 1 1 1 0 1  1  1  1  1  1  1  1  1  1  1  1  1
  placebo    1 1 1 1 1 1 1 1 1  1  1  1  1  1  1  1  1  1  1  1  1

, , time_point = Baseline 2

            Subject
Treatment    1 2 3 4 5 6 7 8 9 20 21 22 23 24 25 26 27 28 29 30 31
  Phlebotomy 1 1 1 1 1 1 1 0 1  1  1  1  1  1  1  1  1  1  1  1  1
  placebo    0 1 0 1 1 1 1 1 1  1  1  1  1  1  1  1  1  1  1  1  1

[1] "Treatment"   "Subject"     "time_point"  "Hb..g.dL."   "MCH..pg."    "MCHC..g.dL."
[7] "MCV..fl."    "Retic...."   "Retic.count"


Correlation matrix for the dependent varilables
               Hb..g.dL.    MCH..pg. MCHC..g.dL.    MCV..fl.  Retic.... Retic.count
Hb..g.dL.    1.000000000 -0.06580095   0.2300736 -0.23750662 -0.2420295 0.007253484
MCH..pg.    -0.065800953  1.00000000   0.2607208  0.75688745  0.2619209 0.123775019
MCHC..g.dL.  0.230073640  0.26072085   1.0000000 -0.31399836  0.1030669 0.123685146
MCV..fl.    -0.237506619  0.75688745  -0.3139984  1.00000000  0.1938071 0.025301734
Retic....   -0.242029523  0.26192087   0.1030669  0.19380706  1.0000000 0.955760979
Retic.count  0.007253484  0.12377502   0.1236851  0.02530173  0.9557610 1.000000000
[1] Baseline 1 Baseline 2 3 days     14 days    25 days
Levels: 14 days 25 days 3 days Baseline 1 Baseline 2
```

Cross-over design

```
##########Use the mean of Baseline 1 and 2 as Baseline value
###############[1] 162  11
[1] 3 days   14 days  25 days  Baseline
Levels: 14 days 25 days 3 days Baseline 1 Baseline 2 Baseline


################ time point:  14 days measurement:  Hb..g.dL. ###############
```

	numDF <int>	denDF <dbl>	F-value <chr>	p-value <chr>
(Intercept)	1	18	91.73061	<.0001
Treatment	1	17	55.81482	<.0001
period	1	17	4.12047	0.0583
tseq	1	18	3.23582	0.0888

4 rows

```
#########################################################################
##########################



################ time point:  25 days measurement:  Hb..g.dL. ###############
```

	numDF <int>	denDF <dbl>	F-value <chr>	p-value <chr>
(Intercept)	1	18	24.595951	0.0001
Treatment	1	17	8.268737	0.0105
period	1	17	0.007883	0.9303
tseq	1	18	2.284370	0.1480

4 rows

```
############################################################################
##########################

################ time point:  3 days measurement:  Hb..g.dL. ###############
```

	numDF <int>	denDF <dbl>	F-value <chr>	p-value <chr>
(Intercept)	1	18	111.83610	<.0001
Treatment	1	18	184.52548	<.0001
period	1	18	1.56737	0.2266
tseq	1	18	3.32853	0.0847

4 rows

```
############################################################################
##########################
```

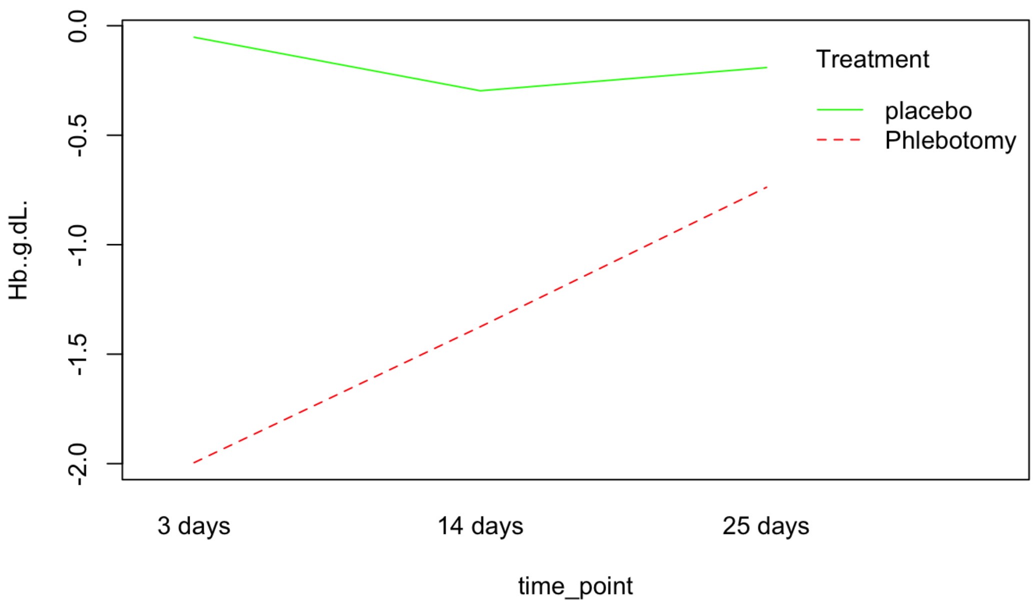


```
################ time point:  14 days measurement:  MCH..pg. ###############
```

	numDF <int>	denDF <dbl>	F-value <chr>	p-value <chr>
(Intercept)	1	18	0.0290347	0.8666
Treatment	1	17	0.7338136	0.4036
period	1	17	1.5821467	0.2255
tseq	1	18	0.0226521	0.8820

4 rows

```
############################################################################
##########################

################ time point:  25 days measurement:  MCH..pg. ###############
```

	numDF <int>	denDF <dbl>	F-value <chr>	p-value <chr>
(Intercept)	1	18	1.9810073	0.1763
Treatment	1	17	0.0272362	0.8709
period	1	17	2.1342341	0.1623
tseq	1	18	0.2755240	0.6061

4 rows

```
############################################################################
#########################

################ time point:  3 days measurement:  MCH..pg. ###############
```

	numDF <int>	denDF <dbl>	F-value <chr>	p-value <chr>
(Intercept)	1	18	0.0583115	0.8119
Treatment	1	18	0.0583115	0.8119
period	1	18	0.5660340	0.4616
tseq	1	18	2.9691754	0.1020

4 rows

```
############################################################################
#########################
```

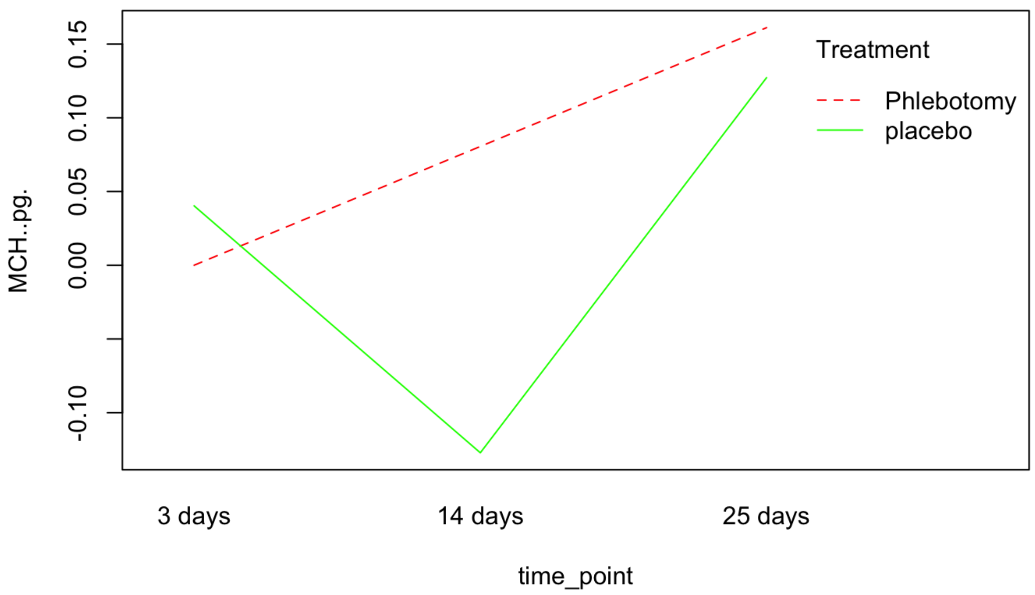


```
################ time point:  14 days measurement:  MCHC..g.dL. ###############
```

	numDF <int>	denDF <dbl>	F-value <chr>	p-value <chr>
(Intercept)	1	18	2.0187549	0.1725
Treatment	1	17	0.0004147	0.9840
period	1	17	1.2044672	0.2877
tseq	1	18	0.0683320	0.7967

4 rows

```
############################################################################
#########################

################ time point:  25 days measurement:  MCHC..g.dL. ###############
```

	numDF <int>	denDF <dbl>	F-value <chr>	p-value <chr>
(Intercept)	1	18	1.4746692	0.2403
Treatment	1	17	0.8481687	0.3700
period	1	17	0.0047336	0.9460
tseq	1	18	0.0615946	0.8068

4 rows

```
##########################################################################
##########################

################ time point:  3 days measurement:  MCHC..g.dL. ###############
```

	numDF	denDF	F-value	p-value
	<int>	<dbl>	<chr>	<chr>
(Intercept)	1	18	1.3797674	0.2554
Treatment	1	18	0.3338669	0.5705
period	1	18	0.1374699	0.7151
tseq	1	18	1.3085410	0.2676

4 rows

```
##########################################################################
##########################
```

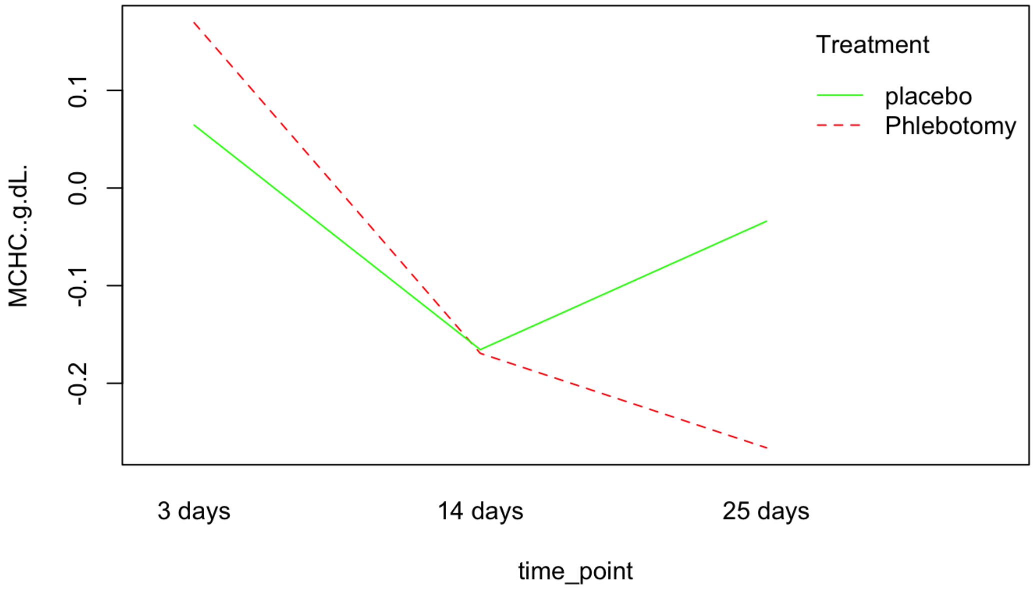


```
################ time point:  14 days measurement:  MCV..fl. ###############
```

	numDF	denDF	F-value	p-value
	<int>	<dbl>	<chr>	<chr>
(Intercept)	1	18	14.437097	0.0013
Treatment	1	17	3.364034	0.0842
period	1	17	0.679356	0.4212
tseq	1	18	0.841953	0.3710

4 rows

```
##########################################################################
##########################

################ time point:  25 days measurement:  MCV..fl. ###############
```

	numDF	denDF	F-value	p-value
	<int>	<dbl>	<chr>	<chr>
(Intercept)	1	18	16.901658	0.0007
Treatment	1	17	6.744724	0.0188
period	1	17	1.023332	0.3259
tseq	1	18	1.672958	0.2122

4 rows

```
############################################################################
#########################


################ time point:  3 days measurement:  MCV..fl. ###############
```

	numDF <int>	denDF <dbl>	F-value <chr>	p-value <chr>
(Intercept)	1	18	0.1562853	0.6972
Treatment	1	18	0.0173650	0.8966
period	1	18	2.4839018	0.1324
tseq	1	18	0.2401286	0.6300

4 rows

```
############################################################################
#########################
```

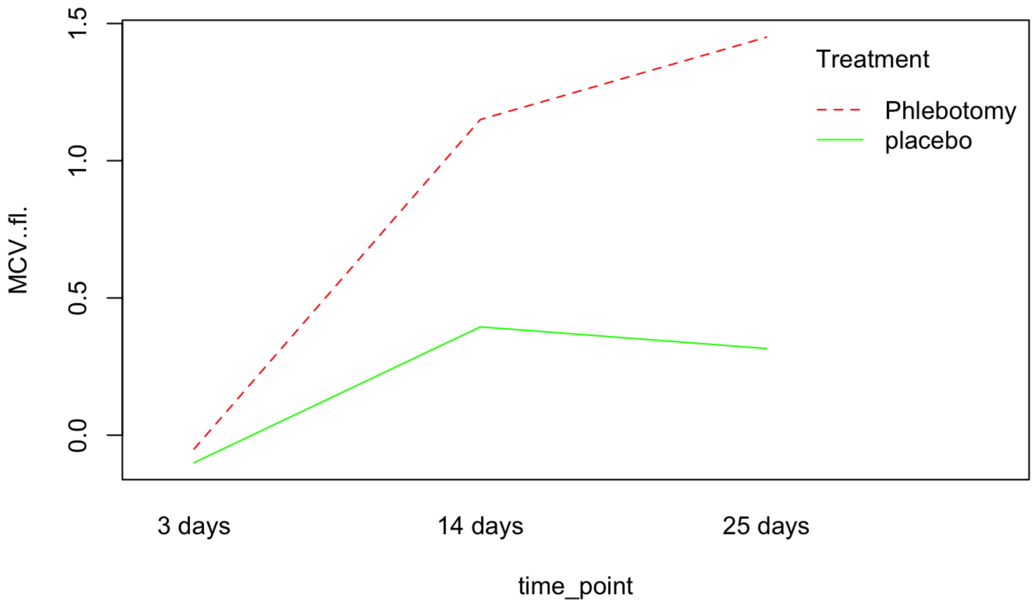


```
################ time point:  14 days measurement:  Retic.... ###############
```

	numDF <int>	denDF <dbl>	F-value <chr>	p-value <chr>
(Intercept)	1	18	125.71963	<.0001
Treatment	1	17	76.59931	<.0001
period	1	17	0.71310	0.4101
tseq	1	18	1.45510	0.2433

4 rows

```
############################################################################
#########################


################ time point:  25 days measurement:  Retic.... ###############
```

	numDF <int>	denDF <dbl>	F-value <chr>	p-value <chr>
(Intercept)	1	18	24.552824	0.0001
Treatment	1	17	15.699562	0.0010
period	1	17	0.112146	0.7418
tseq	1	18	0.842159	0.3709

4 rows

```
##########################################################################
##########################


################ time point:  3 days measurement:  Retic.... ###############
```

	numDF	denDF	F-value	p-value
	<int>	<dbl>	<chr>	<chr>
(Intercept)	1	18	51.63309	<.0001
Treatment	1	18	35.08276	<.0001
period	1	18	0.00583	0.9400
tseq	1	18	0.00277	0.9586

4 rows

```
##########################################################################
##########################
```

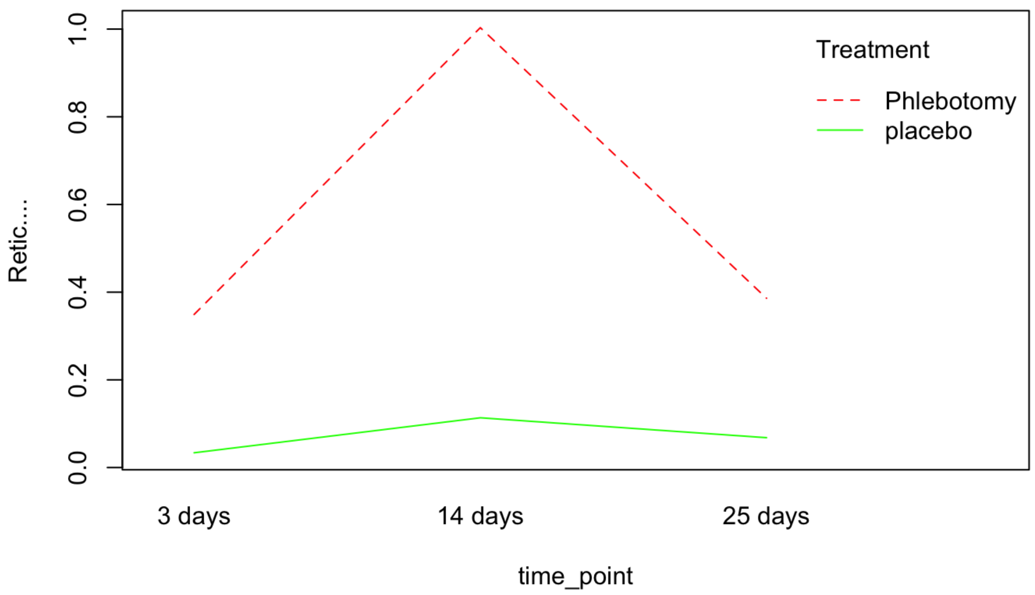


```
################ time point:  14 days measurement:  Retic.count ###############
```

	numDF	denDF	F-value	p-value
	<int>	<dbl>	<chr>	<chr>
(Intercept)	1	18	115.28009	<.0001
Treatment	1	17	70.15011	<.0001
period	1	17	2.06103	0.1693
tseq	1	18	1.17870	0.2919

4 rows

```
##########################################################################
##########################


################ time point:  25 days measurement:  Retic.count ###############
```

	numDF	denDF	F-value	p-value
	<int>	<dbl>	<chr>	<chr>
(Intercept)	1	18	14.198772	0.0014
Treatment	1	17	10.587278	0.0047
period	1	17	0.125420	0.7276
tseq	1	18	0.242599	0.6283

4 rows

```
############################################################################
##########################
```
9999

```
################ time point:  3 days measurement:  Retic.count ###############
```

	numDF <int>	denDF <dbl>	F-value <chr>	p-value <chr>
(Intercept)	1	18	9.186449	0.0072
Treatment	1	18	5.519200	0.0304
period	1	18	0.088983	0.7689
tseq	1	18	0.410961	0.5296

4 rows

```
############################################################################
##########################
```

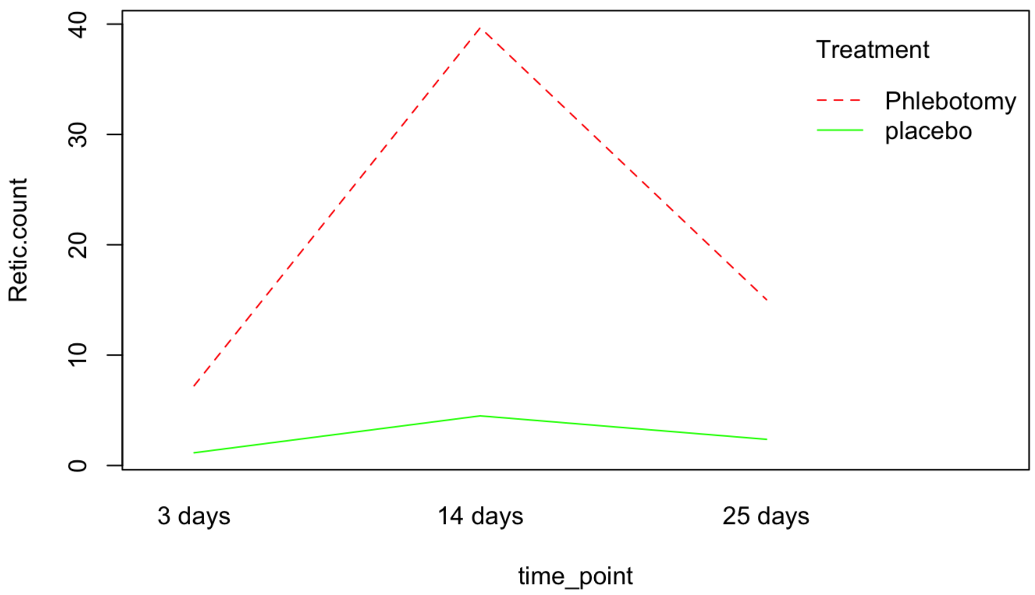

Data visualization

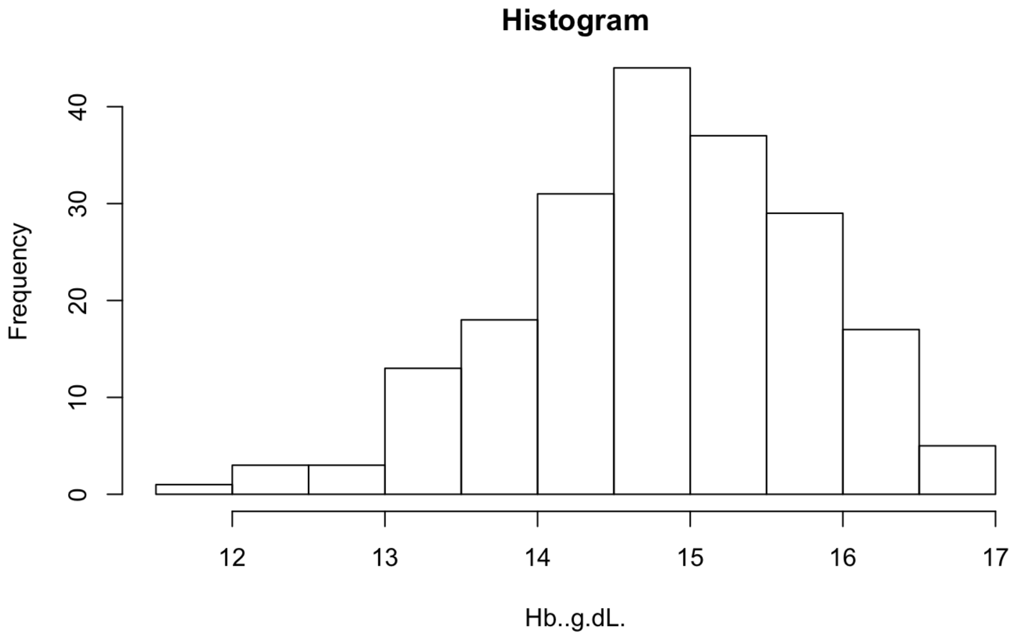

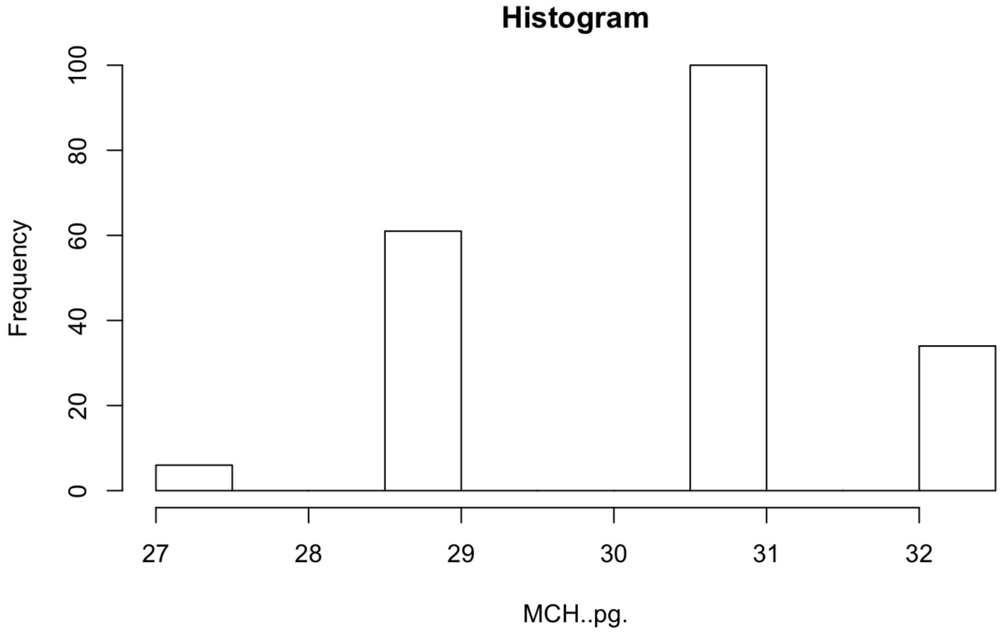
Histogram
Frequency
0
20
40
60
80
100
27
28
29
30
31
32
MCH..pg.

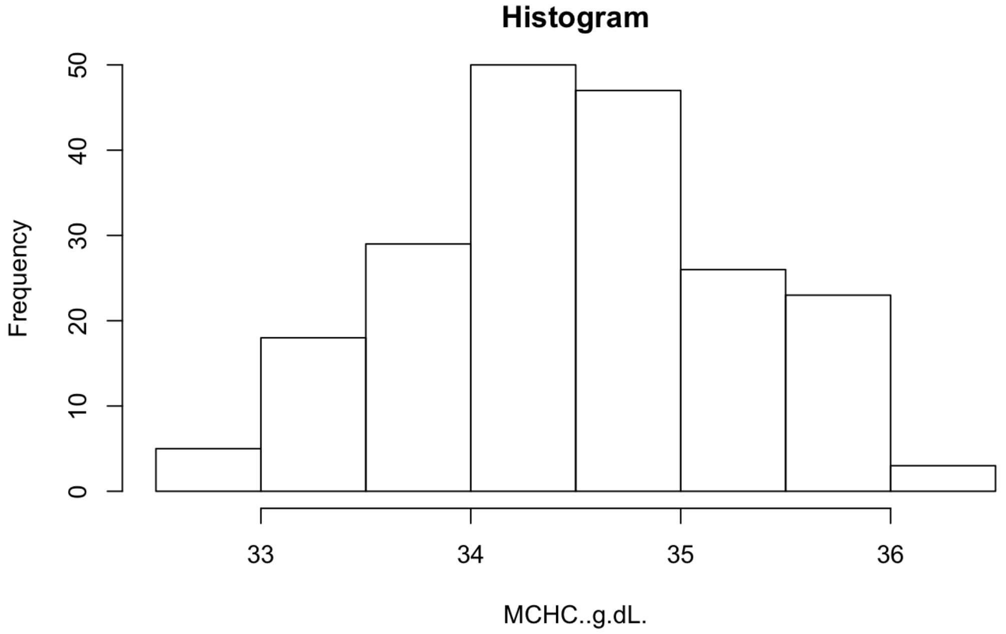
Histogram
Frequency
0
10
20
30
40
50
33
34
35
36
MCHC..g.dL.

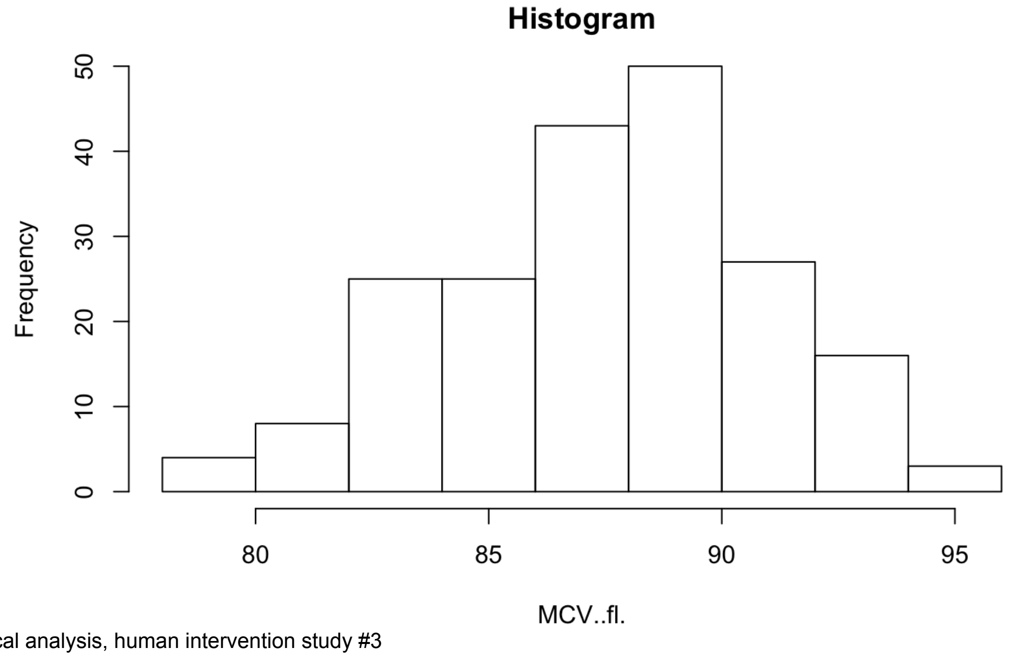
Histogram
Frequency
0
10
20
30
40
50
80
85
90
95
MCV..fl.

101101

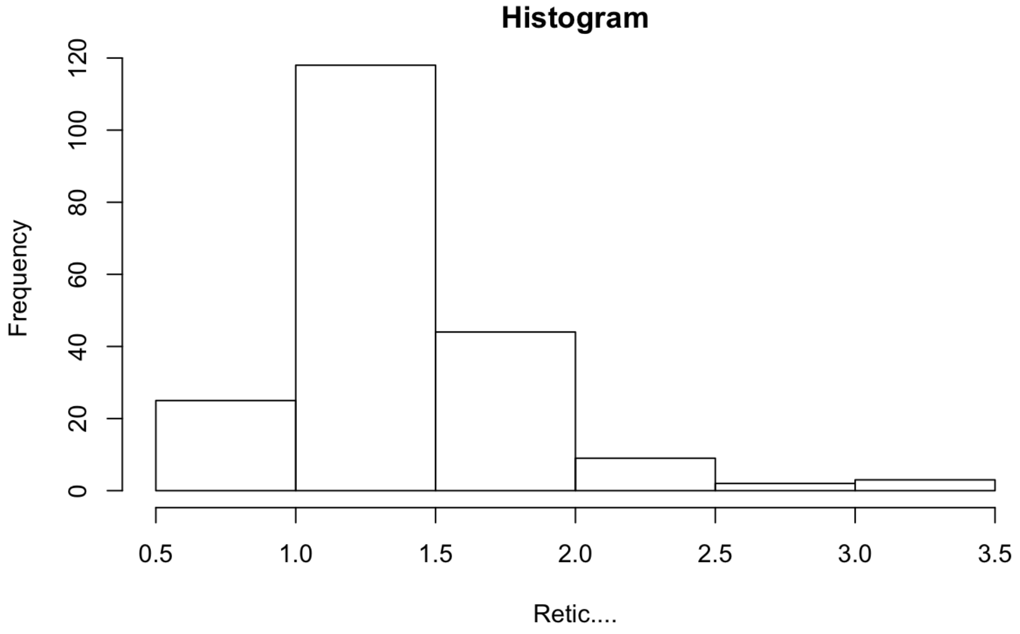
Histogram
Frequency
0
20
40
60
80
100
120
0.5
1.0
1.5
2.0
2.5
3.0
3.5
Retic....

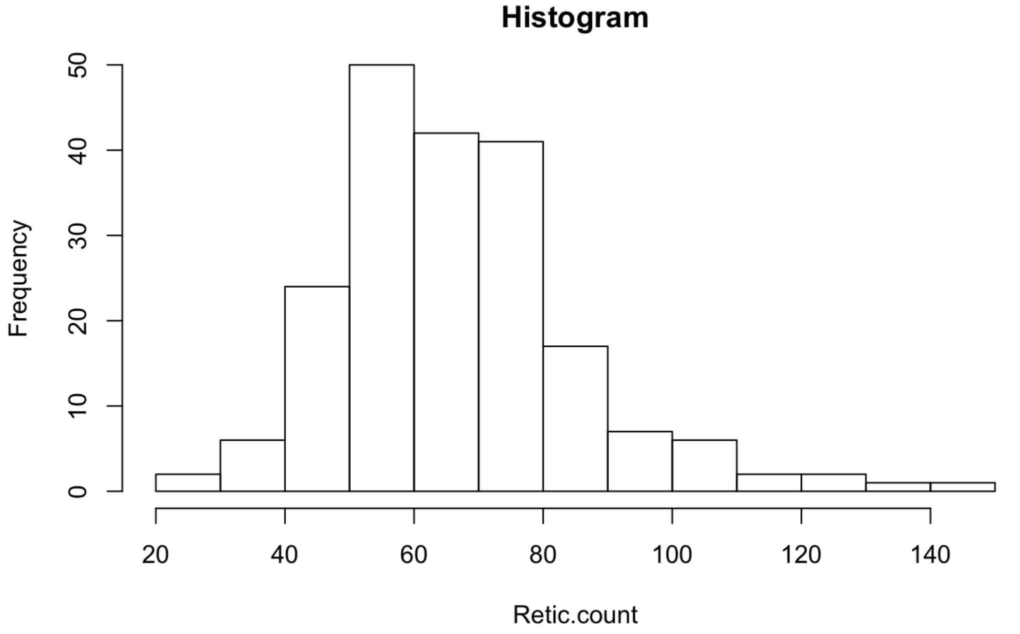
Histogram
Frequency
0
10
20
30
40
50
20
40
60
80
100
120
140
Retic.count

Protocol

Title

Markers for altitude exposure and recombinant human erythropoietin

Introduction

It is known that altitude exposure cause fluctuations of the hematological values with relevance for the Athlete Biological Passport in elite athletes, both during as well as after the altitude exposure (Bonne et al. 2014; Schumacher et al. 2015). These fluctuations can be decisive to whether the athletes hematological values in the Athlete Biological Passport are interpreted as being normal or abnormal (e.g. due to doping). I the present project we wish to identify biomarkers with a high sensitivity towards altitude exposure by applying a metabolomics and proteomics analysis, as ‘omics’ methods have proven valuable in other contexts where biomarkers of an exposure has been identified (Boyard-Kieken et al. 2011; Kiss et al. 2013). A sensitive biomarker will improve the interpretation of the hematological values in the Athlete Biological Passport.

Furthermore, the currently applied methods in detection of recombinant human erythropoietin (rHuEPO) are challenged by a short window of detection (Dehnes et al. 2013; Martin et al. 2016) and the influence of confounders (Bejder et al. 2015). Thus, it is necessary to investigate the application of novel methods. In the present study we wish to apply a metabolomics and proteomics analysis in the identification of biomarkers with high sensitivity towards rHuEPO treatment.

In addition, biomarkers related to the iron metabolism as well as the exercise performance is expected to be sensitive to both altitude exposure and rHuEPO treatment, since both altitude exposure and rHuEPO affect the red blood cell production. Thus, in the present project we also wish to investigate the sensitivity of markers related to the iron metabolism and exercise performance towards altitude exposure and rHuEPO treatment. As it is known that the fluctuation of biomarkers with relevance to anti-doping is different between genders, it will be necessary to investigate the applicability of the methods in both men and women.

Finally, anecdotal evidence suggests that some athletes combine altitude exposure and rHuEPO treatment, which may be due to an expectation that the fluctuations caused by altitude cannot be separated by the fluctuations caused by rHuEPO treatment. Thus, in the present project we wish to

investigate whether the identified biomarkers with high sensitivity towards altitude exposure and rHuEPO treatment can be used to separated subjects who received rHuEPO during altitude exposure from subjects who did not receive rHuEPO during altitude exposure.

Hypothesis

The primary hypothesis for the present study is that metabolomics and proteomics are able to identify biomarkers with high sensitivity to altitude exposure and recombinant human erythropoietin treatment.

Design & methods

Inclusion criteria

We will include 20 Danish male and 20 Danish female subjects with an age of 18-35 years. The subjects must have a maximal oxygen uptake of > 48 ml/min/kg for men and > 43 ml/kg/min for females. The subjects must have a history of regular exercise, including running (>2 times/week, >30 min/session) of more than two years and be able to complete a 5,000 m run in less than 22 min for men and less than 25 min for females in sea-level and dry conditions. Furthermore, the subjects may not have had any running related injuries for the past two years. Prior to the study the subjects will be screened for the mentioned variables as well as the following (inclusion criteria in the parenthesis): blood pressure (diastolic / systolic < 130 / 90 mmHg), Hb1Ac (< 40 mmol/mol), HDL (> 1 mM), LDL (> 3.0 mM), hemoglobin (7-10,5 mM), iron (9-34 uM), transferrin (24-41 uM), red blood cell count (4,25- 5,71 x 10^{12} / L) and electrocardiogram (normal). Additionally, the subjects must be healthy and must not have donated any blood three months prior to the study and must not donate blood for other purposes during the study period.

Design

The study protocol consists of two experimental periods and all subjects must complete both periods. Both experimental periods contain a four week baseline section followed by four weeks of training camp and four weeks of follow-up. The training camp during the first experimental period will be conducted at sea-level while the training camp during the second experimental period will be conducted at 2320 m above sea-level. The experimental periods are separated by at least two

months to secure that the subjects return to baseline. During the study the subjects will be transported by flight and car, for instance during the transport to the training camp.

Experimental period 1

Week 1-4 (baseline) - The subjects follow an individual training schedule at sea-level.

Week 5-8 (intervention) - The subjects follow an individual training schedule at sea-level during the participation in a four week long training camp at sea-level.

Week 9-12 (follow-up) - The subjects follow an individual training schedule at sea-level.

Experimental period 2

Week 1-4 (baseline) – The subjects follow an individual training schedule at sea-level.

Week 5-8 (intervention) – The subjects follow an individual training schedule during the participation in a four week altitude training camp at 2320 m of altitude in Sierra Nevada, Spain. The subjects will be staying at the Centro de Alto Rendimiento (C.A.R) in Sierra Nevada due to the availability of altitude training camp facilities, including accommodation, training facilities and medical facilities (www.carsierranevada.com). An altitude exposure for four weeks has been chosen to ensure a complete hematological response, since three weeks of altitude exposure is close to the borderline for a complete response to occur, even in 3,400 m of altitude (Siebenmann et al. 2015).

rHuEPO treatment

The subjects will undergo a rHuEPO treatment in order to investigate whether the described methods can identify biomarkers with high sensitivity to the physiological response of a rHuEPO treatment.

The subjects will randomly be divided into four groups as illustrated in the table below. The table illustrates when the groups will be treated with rHuEPO or placebo.

The distribution of subjects into the four groups allows the following:

- 28 subjects receive rHuEPO at sea-level, enabling an identification of biomarkers with high sensitivity towards rHuEPO
- 28 subjects receive placebo during altitude exposure, enabling an identification of biomarkers with high sensitivity towards altitude exposure
- 12 subjects receive placebo during sea-level, enabling an identification of random fluctuations in the identified biomarkers
- 12 subjects receive rHuEPO during altitude exposure, enabling an investigation of whether the identified biomarkers are able to differentiate between subjects received rHuEPO during altitude exposure from subjects receiving placebo during altitude exposure.
- 8 subjects receive placebo during both sea-level and altitude exposure, enabling an identification of the individual and random fluctuations of the identified biomarkers in a long term (> 6 months). As these random fluctuations can be determined by n=1, the inclusion of n=8 will be sufficient.

The subjects will be treated with rHuEPO during the first three weeks of the training camp. The rHuEPO injection will be intravenous and consist of 20 international units (IU) per kg bodymass Eprex (Janssen) every second day for three weeks to ensure a sufficient physiological response. The placebo treatment will be given at the same time points as the rHuEPO injections and consist of 0,9 % NaCl in the same amount as the Eprex-injections. The subjects will be blinded, meaning that they don’t know if the receive rHuEPO or saline.

Table 1. Distribution of rHuEPO and placebo treatment during the two experimental periods in the four groups

	n	*Experimental period 1 (sea-level)*	*Experimental period 2 (altitude)*
Group A	20	rHuEPO	Placebo
Group B	8	rHuEPO	rHuEPO
Group C	8	Placebo	Placebo
Group D	4	Placebo	rHuEPO

Nutrition

It is well known that altitude exposure increase the sympathetic outflow (Mazzeo et al 1994; Hansen et al. 2000), stimulating the basal metabolism (Butterfield 1999) as well as the lipolysis (McClelland et al. 2001). In has been demonstrated that exercise at ~2,200 m of altitude can cause a loss of body fat in Olympic athletes (Bonne et al. 2014). There, it is important to monitor the body composition of the athletes during the intervention period and ensure a stable body weight through nutrition guidelines. Thus, the body composition will be measured weekly using the InBody 570-system. In addition, the subject's ferritin concentration will be measured weekly, and the participants must ingest 100 mg iron (Ferrosulfat, ACO) daily during the study period to ensure sufficient iron stores for the expected increased erythropoiesis due to rHuEPO or altitude exposure.

Exercise training

The subjects must adhere to the individual training schedule during the entire study period. The training schedule will be tailored for each individual and contain a mix of running and strength training for 1-2 hours per day. All subjects will be equipped with a running watch and a heart rate monitor, enabling a precise registration of altitude exposure, running speeds as well as daily activity. The training intensity will be analyzed by pulse, time spent at different running speeds and work:rest ratios.

Blood volume

The determination of blood- and plasma volume as well as total hemoglobin mass will be determined weekly during the two experimental periods by rebreathing of a small amount of carbon monoxide (CO) mixed with 100 % oxygen. CO-bound hemoglobin, hemoglobin concentration and hematocrit will be analyzed on a Radiometer ABL 800 before and after the rebreathing in a blood sample. The blood sample will be collected via a fingerstick or by the use of a “venflon” (small plastic hose) in an arm vein without the use of a tourniquet.

Performance testing

The exercise performance of the subjects will be measured weekly during the two experimental periods. The testing includes running tests on submaximal and maximal intensities to exhaustion. Blood will be collected during the test via a fingerstick or a venflon in an arm vein. The purpose of the blood samples is to analyze metabolite concentrations related to exercise intensity, e.g. lactate. Furthermore, a range of physiological measures will be conducted with non-invase techniques (e.g. heart rate, blood pressure, oxygen uptake etc.) All performance tests are conducted by blinded staff members.

Blood and urine samples

All blood- and urine samples are collected according to the guidelines by World Anti-Doping Agency (WADA 2014). Blood- and urine samples will be collected weekly during the two experimental periods. During the study, the amount of collected blood will be less than 25 mL per week corresponding to less than 300 mL during one experimental period. Moreover, the amount of collected urine will amount to less than 100 mL per week corresponding to less than 1200 mL during one experimental period. The purpose of the blood- and urine samples is an identification of sensitive biomarkers towards altitude exposure and rHuEPO treatment.

Analyzes

Whole blood is collected in EDTA containing vacutainers for a complete blood count. Following the complete blood count the plasma will be separated from the red- and white blood cells by centrifugation. Plasma will immediately be stored at -80 °C for later analysis of biomarkers related to iron metabolism (e.g. hepcidine or erythroferrone).

In addition, whole blood will be collected in lithium heparin containing vacutainers. The plasma will be separated from the red- and white blood cells by centrifugation and immediately stored at -80 °C for later analysis of biomarkers related to iron metabolism (e.g. ferritin and transferrin).

For the metabolomic analysis, a blood spot will be collected (< 100 uL) on dry filter paper. The paper is air dried and stored at room temperature. Additionally, whole blood is collected in EDTA containing vacutainers for metabolomic and proteomic analysis. The plasma will be separated from the red- and white blood cells by centrifugation and immediately stored at -80 °C. The remaining cells are immediately washed twice with a phosphate buffered solution before the cells are lysed by a 1:1 dilution with water. The cells are then stored at -80 °C for later analysis. Furthermore, urine samples are collected in sterile containers. The specific gravity of the urine will be determined by a refractometer and 2 x 1 mL of urine will be stored at -80 °C. The purpose of the samples is an identification of biomarkers with a high sensitivity towards altitude exposure and rHuEPO treatment.

Finally, whole blood is collected in EDTA containing vacutainers for future non-specific research. The plasma will be separated from the red- and white blood cells by centrifugation and immediately stored at -80 °C.

All plasma samples collected in Sierra Nevada, Spain, will be shipped to Denmark with the purpose of storing the samples in the research biobank until analysis or further shipment.

Research biobank

A research biobank will be established with the purpose of storing collected biological material consisting of blood- and urine samples until the analyses are complete and the project has been finalized and published. The biological material will be stored in coded containers, meaning that the biological material can only be identified to a person with a keycode stored by the principal investigator. The research biobank will expire no later than the 1st of January 2015. If any biological material is in excess it will be destroyed.

Furthermore a biobank is established. We wish to store any excess biological material as well as extra biological material collected during the project for future non-specific research. The subjects will be asked whether they wish to donate the excess as well as the extra biological material to the biobank for possible future research projects. If the subject wishes to donate excess and extra

biological material, a separate informed consent will have to be signed, which is in correspondence to current guidelines (Appendix 1).

The biological material of the biobank will be stored in coded containers, who only can be identified with a keycode stored by the principal investigator, for no more than 15 years. If the material is used within 15 years it will be destroyed. The donation to the biobank is voluntary and the decision to donate or not will not affect the possibility of participating in the present research project. The subject may at any time contact the principal investigator and ask that the donated material is destroyed.

The biobank material can only be used for future research if the future research project is approved by the Danish scientific ethical committee, unless the samples are anonymized, meaning that the keycode has been destroyed and that the material cannot be identified to a person. The applicant is aware that the collection of biological material for the biobank does not fall under the code of the ethical committee but the law of personal information. However, as we consider the procedure relevant to the full understand of the present research protocol, the information is included.

Blood- and urine samples from the research biobank will be shipped to German Sport University Cologne, Institute for Biochemistry / Center for Preventive Doping Research, Cologne, German in a pseudo anonymized way, meaning that the data handler will not know the identity of the subject. The purpose of the shipment is to conduct the metabolomic analysis, as the laboratory has extensive experience in such analysis. Furthermore, pseudo anonymized blood- and urine samples will be shipped to a World Anti-Doping Agency accredited laboratory within the EU, with the purpose of conducting the proteomics analysis, as World Anti-Doping Agency laboratories have extensive experience with such analysis.

Regarding the shipment of blood- and urine samples, personal data will be protected by Danish legislation in accordance with the Directive 95/46/EC of the European Parliament and the Council of the 24th of October 1995 on the protection of individuals with regard to the processing of personal data and on the free movement such information. In addition, the law of personal information will be respected.

The research biobank, the biobank and data processing agreements have been reported to the Danish Data Protection Agency through the Faculty SCIENCE Joint Report with journal number 2015-57-0116 to the Danish Data Protection Agency

Statistics

As this study applies a non-specific metabolome and proteome analysis for the first time, in an attempt to identify biomarkers for hypoxic exposure and rHuEPO treatment, it is not possible to calculate the expected or necessary power. However, previous investigations at our department have shown that 8-12 participants enable detection of small changes in e.g. whey protein intake. Thus, it is expected that n=28 receiving placebo at altitude and n=28 receiving rHuEPO at sea level is sufficient to identify biomarkers with a high sensitivity for these exposures/treatments, which is the primary aim of the present project.

In addition, the identified biomarkers are validated in smaller groups (n=12), i.e. we investigate whether the biomarkers are useful for separating altitude exposure from altitude exposure combined with rHuEPO treatment, which is the secondary aim of the present project. As this also represents a first attempt, it is not possible to calculate the expected or necessary power, but n=12 is expected to be sufficient based on previous studies.

Ultimately, the goal is to identify markers of rHuEPO abuse at sea level or in conjunction with altitude training, that are sufficiently specific to allow evaluation in single individuals. Therefore, it is also necessary, at the individual level, to know the normal fluctuation over a long period of time. This is investigated with n = 8 receiving placebo during both sea level and altitude exposure. This will make it possible to identify the normal fluctuation at the individual level, both during each experimental round but also over a longer period.

It is expected that a maximum of 25% of the subjects (corresponding to n = 10) will drop out of the experiment. As it has previously been possible to detect small changes with the methods used on 8-12 trial participants, a dropout of 10 subjects will not have a significant effect on the primary purpose of the study. However, the secondary objectives may be compromised if the outcome of subjects falls unfortunate in relation to the grouping of the subjects.

Side effects, risks and disadvantages

rHuEPO administration

rHuEPO accelerates the production of red blood cells. A risk associated with the use of rHuEPO is an increase in the blood viscosity, which may affect the cardiovascular system if it is not monitored.

To protect the subjects from this risk they will be monitored weekly. The blood pressure and hemoglobin concentration will be measured and the treatment will cease if the blood pressure is higher than 160/110 mmHg or the hemoglobin concentration is higher than 11,5 mmol/L. The rHuEPO treatment will only continue once these values are below the thresholds.

The risk of infection is small when rHuEPO is injected intravenously. To reduce the risk even further alcohol wipes will be used to sterilize the injection site and disposable cannulas and syringes will be used.

Vein puncture

Risks include discomfort, bruising and infection. Blood sampling risks include accidental transmission of infection to the subject due to the lack of use of aseptic technique. Further risk of blood sampling involves transmission of infection to staff members through inadequate safety procedures during blood sampling. To reduce these risks, blood sampling will only be conducted by qualified and experienced staff using an aseptic technique. In addition, sterile syringes, lancets, gloves and alcohol wipes are used.

Exercise testing

The subjects will undergo a number of exercise tests including running tests. During these tests, various physiological measurements will be performed with non-invasive techniques (e.g. heart rate, blood pressure, oxygen uptake etc.) that are not associated with discomfort or known risks. Discomfort may be expected as with normal exhausting work.

Total hemoglobin mass

The measurement of total hemoglobin mass is done by rebreathing of a small amount of carbon monoxide mixed with 100 % oxygen. During rebreathing the throat can be slightly irritated. At the same time it may feel as if it ticks on the tongue. Blood sampling occurs either by a fingerstick or by a venflon in an arm vein. There may be some soreness after blood sampling. Carbon monoxide can be life threatening if the inhalation is uncontrolled. The amount inhaled by this measurement results in a carbon monoxide binding to hemoglobin of 5-15 %, which corresponds to the level found in smokers. After 24 hours there is no trace of a possible measurement on the previous day.

There are no known risks associated with inhalation of a small amount of carbon monoxide (Schmidt & Prommer 2005).

Security and approval of applied procedures

All procedures are carried out by the attending physician or an assistant under the responsibility of the physician according to possibility in the law of authorization to use assistance. Blood sampling is performed by the physician or by an assistant with extensive experience under the responsibility of the physician. All methods used are well described and previously used to a great extent.

It should be noted that an approval of the present project also will be applied for at the Spanish authorities.

Respect for the subjects' physical and mental integrity as well as privacy

Information about the subject is protected under the Danish law of personal information and and the law of health. All projects carried out at the Department of Exercise, Nutrition and Sports, University of Copenhagen, have been approved by the Danish Data Protection Agency with journal number 2015-57-0117.

Economy

Financial support ($ 930,105) has been granted to the World Anti-Doping Agency to the present project. The support covers salary, materials, analyzes, travel costs, shipments and publication. The amount is transferred to a project account managed by the Department of Exercise, Nutrition and Sports, University of Copenhagen. The project has been initiated by Nikolai Baastrup Nordsborg, and he has no financial affiliations with the supporters or other private companies with an interest in the project. Any surplus is refunded to the sponsor.

Remuneration or other benefits for subjects

The subjects will be equipped with a running watch with a value of ~3.000 DKK, which is used to collect training data and altitude exposure. If the subject completes the entire study, the subject will be offered to keep the watch. In addition, the subject will be reimbursed 2,500 DKK for the

purchase of running equipment (eg running shoes and running clothes) by completing the entire study. If the subjects withdraw from the trial before time, the subjects will be reimbursed 100 DKK per 14 days they have completed. The subjects will not receive any financial compensation if the maximal oxygen uptake is determined to be less than 48 ml / kg / min for men or less than 43 ml / kg / min for females during screening.

Enrollment of participants

Enrollment of the subjects will take place by posting on the website www.forsoegsperson.dk and posting at higher education institutions. In addition, we will use electronic platforms such as Facebook and club websites. The enrollment will take place on the basis of the fully approved bulletin.

Oral information

Before the oral interview:

When the subject takes contact, the time of the oral information is agreed upon. They are informed that it is a request for participation in a health science research project and that it is possible to bring an assessor if desired. The person is informed that he is entitled to evaluate whether he wants to participate for one day's time after the oral information. Further, the person is informed that if any new information of the person’s health is achieved during the research project, the subject may refuse to receive such information.

Oral interview:

The conversation will be conducted by the investigator and will take place in a locked room without interruption. The person will have all the time needed to read the written information, listen to the oral information and to ask questions. The investigator will present the project in layman language so that all technical terms and expressions are omitted. The conversation will contain detailed information about potential risks, side effects, complications and disadvantages, and that there may be unpredictable risks and strains associated with participation in a health science research project. The investigator will advise on the extent of the informed consent, including that consent includes access to necessary information about the subject's health, other purely private relationships and other confidential information in the context of quality control and monitoring. It is emphasized that

participation is voluntary and that the subject can withdraw from the study at any time without justifying it. The consent only means that he or she has felt sufficiently informed to decide on the participation in the study.

Following the oral interview:

After the oral interview, the informed consent will be sought signed. If the person wishes a one day of reflection time, the informed consent will be sought signed after a reflection period of one day. The subject will be informed if new information about the effects, risks, side effects, complications or disadvantages appears during the trial. In addition, the subject will be informed if changes to the protocol occur. If significant information on the health status of the subject is revealed, the subject shall be notified, unless the subject chose not to be. If the subject wishes, the subject will receive information about the results of the project and any consequences it may have.

Availability of information

The subject is guaranteed access to more information about the project, by contacting the investigator Nikolai Baastrup Nordsborg per. email nbn@nexs.ku.dk

Publication of experimental results

A publication is expected in an international journal. If this cannot be done, data will be published via website and / or conference. All data will be attempted published regardless of whether they are negative, positive or inconclusive.

Scientific ethical statement

The project has a basic research and an anti-doping perspective. The results of the experiment will contribute significantly to the optimization of anti-doping efforts and increase the likelihood of being tested positive for rHuEPO abuse. This will, most likely, reduce the number of people who will use rHuEPO as a performance enhancer. The subjects can receive data on their measured parameters from all trials. The discomfort that the subjects will experience when completing the project is not considered unreasonable. The discomfort will correspond to what subjects have undergone for the last 100 years in exercise physiological research (Poulsen et al. 2009). There is no

known risk of permanent injuries during the experiment. Informed consent of all participants will be obtained in accordance with the guidelines of the Scientific Committee. The information on the subject is protected by the Danish law on personal information and Danish law of health.

Information on compensation or compensation schemes

The subjects are covered by the Danish law on occupational injury insurance under current insurance conditions at the Faculty of SCIENCE, University of Copenhagen. During the entire study they will be covered by the Danish law on Complaints and Compensation Access in the Health Service (Legislation No. 1022 28/08/2017 www.retsinformation.dk).

Supplmenetary References for the Protocol of Human Intervention Study #3:

Bejder J, Hoffmann MF, Ashenden M, Nordsborg NB, Karstoft K, Morkeberg J. Acute hyperhydration reduces athlete biological passport OFF-hr score. *Scand J Med Sci Sports*. 2015

Bonne TC, Lundby C, Lundby AK, Sander M, Bejder J, Nordsborg NB. Altitude training causes haematological fluctuations with relevance for the Athlete Biological Passport. *Drug Test Anal*. 2014.

Bonne TC, Lundby C, Jørgensen S, Johansen L, Mrgan M, Bech SR, Sander M, Papoti M, Nordsborg NB. "Live High-Train High" increases hemoglobin mass in Olympic swimmers. Eur J Appl Physiol. 2014;114(7):1439-49.

Boyard-Kieken F, Dervilly-Pinel G, Garcia P, Paris AC, Popot MA, le Bizec B, Bonnaire Y. Comparison of different liquid chromatography stationary phases in LC-HRMS metabolomics for the detection of recombinant growth hormone doping control. *J Sep Sci*. 2011: **34**: 3493-3501.

Butterfield GE (1999) Nutrient requirements at high altitude. Clin Sports Med 18(3):607–621

Dehnes Y, Shalina A, Myrvold L. Detection of recombinant EPO in blood and urine samples with EPO WGA MAIIA, IEF and SAR-PAGE after microdose injections. *Drug Test Anal*. 2013: **5**: 861-869.

Hansen J, Sander M, Hald CF, Victor RG, Thomas GD (2000) Metabolic modulation of sympathetic vasoconstriction in human skeletal muscle: role of tissue hypoxia. J Physiol 527(Pt 2):387–396

Kiss A, Lucio M, Fildier A, Buisson C, Schmitt-Kopplin P, Cren-Olive C. Doping control using high and ultra-high resolution mass spectrometry based non-targeted metabolomics-a case study of salbutamol and budesonide abuse. *PLoS One*. 2013: **8**: e74584.

Martin L, Ashenden M, Bejder J, Hoffmann M, Nordsborg N, Karstoft K, Morkeberg J, Sharpe K, Lasne F, Marchand A. New insights for identification of doping with recombinant human erythropoietin micro-doses after high hydration. *Drug Test Anal*. 2016: **8**: 1119-1130.

McClelland GB, Hochachka PW, Reidy SP, Weber JM (2001) High-altitude acclimation increases the triacylglycerol/fatty acid cycle at rest and during exercise. Am J Physiol Endocrinol Metab 281(3):E537–E544

Mazzeo RS, Wolfel EE, Butterfield GE, Reeves JT (1994) Sympathetic response during 21 days at high altitude (4,300 m) as determined by urinary and arterial catecholamines. Metabolism 43(10):1226–1232

Poulsen AL, Trangbæk E, Jørgensen K, Nordsborg N. Forskning i bevægelse; Et nyt forskningsfelt i et 100-årigt perspektiv. Copenhagen, Museum Tusculanum, p. 322-325. 2009.

Schmidt W, Prommer N. The optimised CO-rebreathing method: a new tool to determine total haemoglobin mass routinely. *Eur J Appl Physiol*. 2005: **95**: 486-495.

Schumacher YO, Garvican LA, Christian R, Lobigs LM, Qi J, Fan R, He Y, Wang H, Gore CJ, Ma F. High altitude, prolonged exercise, and the athlete biological passport. *Drug Test Anal*. 2015: **7**: 48-55.

Siebenmann C, Cathomen A, Hug M, Keiser S, Lundby AK, Hilty MP, Goetze JP, Rasmussen P, Lundby C. Hemoglobin mass and intravascular volume kinetics during and after exposure to 3,454-m altitude. *J Appl Physiol (1985)*. 2015: **119**: 1194-1201.

WADA. The World Anti-Doping Agency. Blood Sample Collection Guidelines. Version 3.0. https://wada-main-prod.s3.amazonaws.com/resources/files/guidelines_blood_sample_collection_v3_0_2014_10_final_eng_0.pdf. 2014.

Study protocol

Background

It is not possible to test for all existing and future doping substances or methods, which e.g. manipulate the oxygen carrying capacity of the blood (Pottgiesser and Schumacher, 2013). Using the ”Athlete Biological Passport” (ABP), the athlete is provided with an individual profile based on selected physiological variables, which has proven valuable since its introduction in 2009 by the World Anti-Doping Agency (WADA).

However, detection of autologous blood transfusion (ABT) is a major challenge and is currently only detectable by the ABP. The sensitivity of the ABP for detection of autologous blood transfusion is, however, only 20-80% when 450-1350 ml of blood is reinfused (Morkeberg et al., 2011; Pottgiesser et al., 2011). In addition, the ABP is confounded, e.g. by altitude exposure or participation in stage races, which both affect the plasma volume (Sawka et al., 1996; Lombardi, 2013) and thereby also the concentration-dependent variables of the ABP.

In an attempt to improve the sensitivity of the ABP, researchers have explored novel biomarkers, for instance determination of the total hemoglobin mass (Prommer et al., 2008; Morkeberg et al., 2011). However, implementing a measurement of total hemoglobin mass in anti-doping is difficult as inhalation of the toxic gas carbon monoxide, which is required in the most common measurement method, reduces the aerobic performance capacity for at least a few hours (Schmidt and Prommer, 2005). From the existing literature, it is clear that novel methods for detection of ABT are warranted. One possible promising methodology is “metabolomics” (Saugy et al., 2009), where a metabolite profile is created for an athlete based on multiple samples.

We hypothesize that the metabolomics approach enable detection of ABT. This hypothesis is based on the fact that storage of red blood cells (RBC) has extensive impact on the RBC metabolite profile, including membrane stiffness (Relevy et al., 2008), reduced anti-oxidant capacity and glutathione homeostasis (D'Alessandro et al., 2013). Furthermore, a metabolomics analysis demonstrated that storge of blood for more than 7 days cause a clear change in the metabolite content, including at least four metabolic pathways in relation to the cell membrane via eicosanoids (Patel et al., 2014).

A metabolomics approach for detection of doping substances has been successful previously. In general, small-scale studies have shown that identification of salbutamol or budesonide (Kiss et al., 2013), anabolic steroid (Rijk et al., 2009) or recombinant human growth hormone (Boyard-Kieken et al., 2011) misuse is possibly by metabolomics. However, the use of metabolomics in the fight against doping is in its infancy (Reichel, 2011).

In this project, we propose that a metabolomics analysis can act as an important tool for identification of autologous blood transfusions. The primary aim of the project is to evaluate whether a metabolomics analysis is capable of detecting autologous blood transfusions. If that is the case, it is an additional aim to identify the metabolites being influenced the most and having the lowest intra-individual variation.

Autologus blood tranfusion is performance enhancing (Ekblom et al., 1972; Berglund et al., 1987). The effect of an autologous blood transfusion on short high-intenst exercise or exercise with a limited muscle mass is however unknown. Accordingly, this will also be investigated in the present study.

Hypothesis

The main hypothesis for the present study is that autologous blood transfusion is detectable by a metabolomics approach.

Design & Methods

Inclusion and exclusion criteria for participation

We will enroll 24 participants for two difference trial arms (n = 12 for each arm). If not described otherwise, the following design is applied to both trial arms.

The participants must be endurance-trained males in the age of 18-39 years with a fitness level higher than 55 ml O2/min/kg. All participants will be screened, including measurement of hemoglobin and iron levels and the maximal whole body oxygen consumption to determine whether the person can participate in the study. The participants must be healthy non-smokers, must not have donated blood within 3 months, must not donate blood for other purposes during the study and have a hematocrit above 40%. In addition, the participants must not be exposed to altitude of more than 1000m above sea level for at least 2 months before the study.

A double-blind placebo-controlled cross-over design is applied, which include a washout period of 3 months.

Blood and urine samples will be collected twice before blood donation in order to establish an individual baseline metabolome profile and to account for short-term biological variation. As the experiment is conducted in a double-blinded cross-over design, the samples collected during the placebo treatment also serve as baseline samples and provide the opportunity to account for long-term variation of the metabolome profile.

The participants will donate 900 ml whole blood following baseline measurements. The donated blood will be stored at 4°C in a solution of saline, adenine, glucose and mannitol (SAGM) for four weeks. Four weeks have been chosen to allow time for the participants to resynthesize the majority of the lost blood. It should also be noted that it is not possible to store blood at 4°C for a longer time period at the blood bank of University Hospital of Copenhagen.

All participants will receive 66 mg/day of iron supplementation for four weeks after donation, to ensure adequate iron stores for the expected increase in erythropoiesis. In addition, three blood and urine samples will be collected during the four-week recovery period.

A blood sample will be collected from the stored blood after four weeks to determine whether the expected shift in the metabolome profile has occurred (Patel et al., 2014). One the same day the blood will be reinfused, and the participant will receive 900 ml blood in one reinfusion in trial arm 1, whereas the reinfusion is conducted on two consecutive days in trial arm 2. As degradation products from the reinfused blood are likely to be excreted in urine, both blood and urine samples are collected for metabolomics analysis. The optimal sample timing is determined by collection of samples on 8 different time points in the following 14 days.
During the placebo treatment, participants will undergo a sham-donation and sham-reinfusion. A needle is inserted in an antecubital vein at both occasions, and blinding is maintained by a sheet covering the participants arm. The scientific staff will also be blinded as both sham and non-sham donations and reinfusions are performed by the blood bank.

A minimum of 90 days will separate the two treatments (i.e. donation/reinfusion or sham-donation/reinfusion), which is expected to be sufficient for the infused RBCs to be excreted, as RBC has a lifespan of approximately 120 days (Noguchi et al., 2008).

All blood and urine samples will be collected and handled according to World Anti-Doping Agency guidelines (WADA, 2014a; WADA, 2014b).

In additions, the participants in both trial arms will perform exercise tests the week before donation, the week before reinfusion and in the same week as the reinfusion. Moreover, participants of trial arm 2 will perform the exercise tests 6 weeks after the reinfusion. The exercise tests include three cycling tests: a maximal oxygen uptake test, a time trial and a 3-minute all-out test. The participants will be instrumented with a venous catheter during these test for blood sampling. The purpose of the blood samples is to analyze metabolite concentrations related to exercise intensity, e.g. lactate. The participants will also perform an exercise test in a kicking-ergometer to evaluate the effect of an increased oxygen carrying capacity on the performance abilities of a small muscle mass. We will evaluate the muscle function by electrical stimulation during the kicking-ergometer test.

Finally, the total hemoglobin mass is measured twice before donation, before reinfusion as well as after the reinfusion for both trial arms. Participants of trial arm 2 will also have their total hemoglobin mass measured twice six weeks after reinfusion.

Measurements and sample collection

The following applies to both trial arms. We will collect urine and blood samples two times before blood withdrawal. Next, the participants donate two units of 450 ml whole blood each, which after 1-3 h of rest is centrifuged at 4730 g for 8 min and separated into buffy coat, plasma and red blood cells. The red blood cells will be stored in a solution of saline, adenine, glucose and mannitol (SAGM) and leucodepleted. Leucodepletion is applied in many countries and is justified by improving the blood safety, effect and survival. The blood bags will be stored at 4°C until reinfusion. During the storage period, three urine and blood samples will be collected from the participants. The red blood cells will be reinfused after four weeks of storage. Finally, urine and blood samples will be collected at 8 different time points in the following 14 days. The primary purpose of the samples is to analyze the metabolome profile.

Three exercise tests will be completed before the blood withdrawal, the week before reinfusion and within a few days after reinfusion in both trial arms as well as six weeks after reinfusion in trial arm 2. A venous cathether will the inserted into an antecubital vein during the tests for blood sampling. The total hemoglobin mass will be determined in duplicate the week before blood

withdrawal and the week before reinfusion in both trial arms as well as six weeks after reinfusion in trial arm 2.

Due to the nature of a cross-over design, the measurements and collections described above will be performed twice during the study. During the study, the collected amount of blood will be >100 mL per participant (not counting the 900 ml blood withdrawal).

All urine and blood samples are collecte according to WADA guidelines (WADA, 2014a; WADA, 2014b).

Analyses

Investigating the metabolome of blood and urine

Dried blood spots (50 µl) will be collected on filterpaper, which will dry and be stored at room temperature. The remaining blood is separated into plasma, red- and white blood cells by centrifugation. Plasma is stored immediately at -80°C, while the cells are washed twice in a phosphate buffered solution before the cells are lyzed by 1:1 in water and stored at -80°C. Specific gravity will be determined in urine samples and 2x1 mL will be stored at -80°C.

Plasma samples (40uL) are precipitated by addition of 4 vol ice-cold methanol and filtered to remove >98% of the proteins. The filtrate is dried and redissolved (as an emulsion due to phospholipids) in 200uL water with internal standard (13C hippuric acid) in a 96well microtiter tray. A sample pool is produced by adding 10uL from each sample into a tube and used for quality control. All samples, standard, pool and blanks are injected (5uL) into the UPLC-QTOF (Waters Acquity/Premium) and separated by a polarity and flow gradient programme going from water to methanol as described previously (Barri and Dragsted, 2013). The mass detector is set to soft ionization (2.8 kV in negative mode and 3.2 kV in positive mode) and scans approx. 5 s^{-1} for mass in the interval, m/z 50-1000 daltons. The data are centroided in real time and stored as raw data. The raw data files are converted to NetCDF format and aligned by the software, mzMine2 (Pluskal et al., 2010). The aligned data are analyzed by univariate and multivariate analyses in order to detect mass features that are significantly increased or decreased after ABT. For univariate statistics, linear models and false discovery rate analysis is used. For multivariate analyses, PLS-DA is used with extensive internal and external validation as described (Gurdeniz et al., 2013).

Urine samples are diluted 1:1 with pure water, centrifuged to remove precipitates, and analysed directly as described for the redissolved plasma components above.

Determination of total hemoglobin mass

Blood volume, hemoglobin mass and plasma volume is estimated by carbon monoxide (CO) rebreathing. The participant inhales 1.5 ml CO per kg body weight. CO-bound hemoglobin, hemoglobin concentration and hematocrit is determined on a Radiometer ABL 700 before and after the CO rebreathing in a blood sample. The blood sample is collected by a finger prick or by insertion of a cathether in an antecubital vein without application of a tourniquet.

Determination of hematological variables

Hematological variables are determined at the Department of Biochemistry of the National University Hospital of Copenhagen, which following the European standard for quality control I medical laboratories: DS/ES ISO 15189. The quality control is accredited by an accreditation body of the ILAC (International Laboratory Accreditation Cooperation), including EA (European Accreditation) – the European accreditation of laboratories. All blood samples are analyzed according to WADA guidelines for analyses within the biological passport, and complete blood count is performed using a Sysmex XE-2100 instrument.

Research Biobank

Collected blood and urine samples will be stored until the project is complete and published. The purpose of the research biobank is to store the biological material until the analyses are conducted. The biological material will not be given to others or removed from the country. Non-used material will be destroyed by the end of the project.

Statistics

A double-blinded placebo-controlled cross-overstudy provides the best possible chance of detecting relevant changes in the metabolic profile. Since this study represents the first attempt, it is not possible to calculate expected detection limits or the necessary power, but previous investigations have been able to detect small changes (e.g. whey protein intake) with only 8-12 participants.

Side effects, risks and disadvantages

Vein puncture

Risks include discomfort, bruising and infection. Blood sampling risks include accidental transmission of infection to the subject due to the lack of use of aseptic technique. Further risk of blood sampling involves transmission of infection to staff members through inadequate safety procedures during blood sampling. To reduce these risks, blood sampling will only be conducted by qualified and experienced staff using an aseptic technique. In addition, sterile syringes, lancets, gloves and alcohol wipes are used.

Blood transfusion

More than 360,000 blood donations occur yearly in Denmark. The vast majority of blood donation are performed without negative side effects, but side effects do occur at rare occasions. The most common serious side effect during a blood transfusion occur due to confusion resulting in transfusion of blood to the wrong patient or transfusion of wrong blood components. However, most side effects are short-lived and harmless, such as fever and chills, general discomfort, nausea, vomiting, chills, pain, shortness of breath, palpitations, changes in blood pressure, skin rash, diarrhea and red urine and may occur during and immediately after the transfusion. In this case, the patient should be evaluated quickly by a doctor, as the symptoms may be due to hemolysis, contamination of the blood with bacteria, overfilling of the blood vessels or hypersensitivity reactions. In rare cases, infection transmitted by transfusion or destruction of the transfused red blood cells up to several months after the transfusion can cause, for example, fever, unexplained fatigue, loss of appetite, nausea, diarrhea, skin rash, shortness of breath, jaundice and red urine. In these situations, the patient should be encouraged to contact their own doctor or the clinical department where the blood is given to inform about the transfusion and the symptoms. Symptoms may also occur from a nerve affected by insertion of the cannula.

Total hemoglobin mass

The measurement of total hemoglobin mass is done by rebreathing of a small amount of carbon monoxide mixed with 100 % oxygen. During rebreathing the throat can be slightly irritated. At the same time it may feel as if it ticks on the tongue. Blood sampling occurs either by a fingerstick or by a venflon in an arm vein. There may be some soreness after blood sampling. Carbon monoxide can be life threatening if the inhalation is uncontrolled. The amount inhaled by this measurement results in a carbon monoxide binding to hemoglobin of 5-15 %, which corresponds to the level found in smokers. After 24 hours there is no trace of a possible measurement on the previous day. There are no known risks associated with inhalation of a small amount of carbon monoxide (Schmidt & Prommer 2005). However, potentially pregnant women

will not be allowed to participate in the study due to the possible risk of reduced oxygen supply to the fetus.

Exercise testing

The subjects will undergo a number of exercise tests including running tests. During these tests, various physiological measurements will be performed with non-invasive techniques (e.g. heart rate, blood pressure, oxygen uptake etc.) that are not associated with discomfort or known risks. Discomfort may be expected as with normal exhausting work.

Security and approval of applied procedures

All procedures are carried out by the attending physician or an assistant under the responsibility of the physician according to possibility in the law of authorization to use assistance. Blood sampling is performed by the physician or by an assistant with extensive experience under the responsibility of the physician. All methods used are well described and previously used to a great extent. Blood transfusions are performed by the blood bank National University Hospital, which secure the highest possible safety. Participants are covered by the patient insurance, as the trial is being conducted at the University of Copenhagen.

Respect for the subjects' physical and mental integrity as well as privacy

Information about the subject is protected under the Danish law of personal information and and the law of health. All projects carried out at the Department of Exercise, Nutrition and Sports, University of Copenhagen, have been approved by the Danish Data Protection Agency with journal number 2013-54-0522.

Economy

We will apply for funding to the present project, and information on obtained funding will be provided to the ethical committee and the participant information will be updated. The funding will be administered by the Department of Nutrition, Exercise and Sports and University of Copenhagen. The project has been initiated by Nikolai Baastrup Nordsborg, and he has no financial affiliations with the supporters or other private companies with an interest in the project. Any surplus is refunded to the sponsor.

Remuneration or other benefits for subjects

The remuneration consist of 5,000 DKK per participant, which is taxable. The remuneration covers pain, transport and lost earnings. If a participant does not complete all parts of the experiement, the participant receives 1,000 DKK per complete transfusion.

Enrollment of participants

Enrollment of the subjects will take place by posting on the website www.forsoegsperson.dk and posting at higher education institutions. In addition, we will use electronic platforms such as Facebook and club websites. The enrollment will take place on the basis of the fully approved bulletin.

Oral information

Before the oral interview:

When the subject takes contact, the time of the oral information is agreed upon. They are informed that it is a request for participation in a health science research project and that it is possible to bring an assessor if desired. The person is informed that he is entitled to evaluate whether he wants to participate for one day's time after the oral information. Further, the person is informed that if any new information of the person’s health is achieved during the research project, the subject may refuse to receive such information.

Oral interview:

The conversation will be conducted by the investigator and will take place in a locked room without interruption. The person will have all the time needed to read the written information, listen to the oral information and to ask questions. The investigator will present the project in layman language so that all technical terms and expressions are omitted. The conversation will contain detailed information about potential risks, side effects, complications and disadvantages, and that there may be unpredictable risks and strains associated with participation in a health science research project. The investigator will advise on the extent of the informed consent, including that consent includes access to necessary information about the subject's health, other purely private relationships and other confidential information in the context of quality control and monitoring. It is emphasized that participation is voluntary and that the subject can withdraw from the study at any time without justifying it. The consent only means that he or she has felt sufficiently informed to decide on the participation in the study.

Following the oral interview:

After the oral interview, the informed consent will be sought signed. If the person wishes a one day of reflection time, the informed consent will be sought signed after a reflection period of one day. The subject will be informed if new information about the effects, risks, side effects, complications or disadvantages appears during the trial. In addition, the subject will be informed if changes to the protocol occur. If significant information on the health status of the subject is revealed, the subject shall be notified, unless the subject chose not to be. If the subject wishes, the subject will receive information about the results of the project and any consequences it may have.

Availability of information

The subject is guaranteed access to more information about the project, by contacting the investigator Nikolai Baastrup Nordsborg per. email nbn@nexs.ku.dk

Publication of experimental results

A publication is expected in an international journal. If this cannot be done, data will be published via website and / or conference. All data will be attempted published regardless of whether they are negative, positive or inconclusive.

Scientific ethical statement

The project has a basic research and an anti-doping perspective. The subjects can receive data on their measured parameters from all trials. The discomfort that the subjects will experience when completing the project is not considered unreasonable. The discomfort will correspond to what subjects have undergone for the last 100 years in exercise physiological research (Poulsen et al., 2009). There is no known risk of permanent injuries during the experiment. Informed consent of all participants will be obtained in accordance with the guidelines of the Scientific Committee. The information on the subject is protected by the Danish law on personal information and Danish law of health.

Information on compensation or compensation schemes

The patient insurance scheme covers injuries that may be inflicted on subjects during this research project.

Supplmenetary References for the Protocol of Human Intervention Study #1:

Ashenden, M., and J. Morkeberg. 2011. Net haemoglobin increase from reinfusion of refrigerated vs. frozen red blood cells after autologous blood transfusions. *Vox Sang*. 101:320-326.

Barri, T., and L.O. Dragsted. 2013. UPLC-ESI-QTOF/MS and multivariate data analysis for blood plasma and serum metabolomics: effect of experimental artefacts and anticoagulant. *Anal Chim Acta*. 768:118-128.

Berglund, B., P. Hemmingsson, and G. Birgegard. 1987. Detection of autologous blood transfusions in cross-country skiers. *Int J Sports Med*. 8:66-70.

Boyard-Kieken, F., G. Dervilly-Pinel, P. Garcia, A.C. Paris, M.A. Popot, B. le Bizec, and Y. Bonnaire. 2011. Comparison of different liquid chromatography stationary phases in LC-HRMS metabolomics for the detection of recombinant growth hormone doping control. *J Sep Sci*. 34:3493-3501.

D'Alessandro, A., F. Gevi, and L. Zolla. 2013. Red blood cell metabolism under prolonged anaerobic storage. *Mol Biosyst*. 9:1196-1209.

Ekblom, B., A.N. Goldbarg, and B. Gullbring. 1972. Response to exercise after blood loss and reinfusion. *J Appl Physiol*. 33:175-180.

Gurdeniz, G., D. Rago, N.T. Bendsen, F. Savorani, A. Astrup, and L.O. Dragsted. 2013. Effect of trans fatty acid intake on LC-MS and NMR plasma profiles. *PLoS One*. 8:e69589.

Kiss, A., M. Lucio, A. Fildier, C. Buisson, P. Schmitt-Kopplin, and C. Cren-Olive. 2013. Doping control using high and ultra-high resolution mass spectrometry based non-targeted metabolomics-a case study of salbutamol and budesonide abuse. *PLoS One*. 8:e74584.

Lombardi, G. 2013. Reply to Gore et al.: Plasma volume shift during multiday racing. *Clin Chem Lab Med*. 51:e111-112.

Morkeberg, J., K. Sharpe, B. Belhage, R. Damsgaard, W. Schmidt, N. Prommer, C.J. Gore, and M.J. Ashenden. 2011. Detecting autologous blood transfusions: a comparison of three passport approaches and four blood markers. *Scand J Med Sci Sports*. 21:235-243.

Noguchi, C.T., L. Wang, H.M. Rogers, R. Teng, and Y. Jia. 2008. Survival and proliferative roles of erythropoietin beyond the erythroid lineage. *Expert Rev Mol Med*. 10

Patel, R.M., J.D. Roback, K. Uppal, T. Yu, D.P. Jones, and C.D. Josephson. 2014. Metabolomics profile comparisons of irradiated and nonirradiated stored donor red blood cells. *Transfusion*.

Pluskal, T., S. Castillo, A. Villar-Briones, and M. Oresic. 2010. MZmine 2: modular framework for processing, visualizing, and analyzing mass spectrometry-based molecular profile data. *BMC Bioinformatics*. 11:395.

Pottgiesser, T., and Y.O. Schumacher. 2013. Current strategies of blood doping detection. *Anal Bioanal Chem*. 405:9625-9639.

Pottgiesser, T., P.E. Sottas, T. Echteler, N. Robinson, M. Umhau, and Y.O. Schumacher. 2011. Detection of autologous blood doping with adaptively evaluated biomarkers of doping: a longitudinal blinded study. *Transfusion*. 51:1707-1715.

Pottgiesser, T., W. Specker, M. Umhau, H.H. Dickhuth, K. Roecker, and Y.O. Schumacher. 2008. Recovery of hemoglobin mass after blood donation. *Transfusion*. 48:1390-1397.

Poulsen, A.L., E. Trangbæk, K. Jørgensen, and N. Nordsborg. 2009. Forskning i bevægelse; Et nyt forskningsfelt i et 100-årigt perspektiv. Copenhagen, Museum Tusculanum, p. 322-325.

Prommer, N., P.E. Sottas, C. Schoch, Y.O. Schumacher, and W. Schmidt. 2008. Total hemoglobin mass--a new parameter to detect blood doping? *Med Sci Sports Exerc*. 40:2112-2118.

Reichel, C. 2011. OMICS-strategies and methods in the fight against doping. *Forensic Sci Int*. 213:20-34.

Relevy, H., A. Koshkaryev, N. Manny, S. Yedgar, and G. Barshtein. 2008. Blood banking-induced alteration of red blood cell flow properties. *Transfusion*. 48:136-146.
Rijk, J.C., A. Lommen, M.L. Essers, M.J. Groot, J.M. Van Hende, T.G. Doeswijk, and M.W. Nielen. 2009. Metabolomics approach to anabolic steroid urine profiling of bovines treated with prohormones. *Anal Chem*. 81:6879-6888.
Saugy, M., N. Robinson, and C. Saudan. 2009. The fight against doping: back on track with blood. *Drug Test Anal*. 1:474-478.
Sawka, M.N., A.J. Young, P.B. Rock, T.P. Lyons, R. Boushel, B.J. Freund, S.R. Muza, A. Cymerman, R.C. Dennis, K.B. Pandolf, and C.R. Valeri. 1996. Altitude acclimatization and blood volume: effects of exogenous erythrocyte volume expansion. *J Appl Physiol (1985)*. 81:636-642.
Schmidt, W., and N. Prommer. 2005. The optimised CO-rebreathing method: a new tool to determine total haemoglobin mass routinely. *Eur J Appl Physiol*. 95:486-495.
WADA. 2014a. The World Anti-Doping Agency. Blood Sample Collection Guidelines. Version 3.0. https://wada-main-prod.s3.amazonaws.com/resources/files/wada_guidelines_blood_sample_collection_2014_v1.0_en.pdf. Accessed November 19, 2014.
WADA. 2014b. The World Anti-Doping Agency. Urine Sample Collection Guidelines. Version 6.0. https://wada-main-prod.s3.amazonaws.com/resources/files/wada_guidelines_urine_sample_collection_2014_v1.0_en.pdf. Accessed November 19, 2014
