# Supplementary material for: EpoR stimulates rapid cycling and larger red cells during mouse and human erythropoiesis
Source: Nat Commun. 2021 Dec 17;12:7334. doi: 10.1038/s41467-021-27562-4 (PMC8683474; doi:10.1038/s41467-021-27562-4)
Supplement: Supplementary file 2 — Reporting Summary [file 41467_2021_27562_MOESM2_ESM.pdf]

nature portfolio

Corresponding author(s): Socolovsky/Nordsborg

Last updated by author(s): 2021/10/26

Reporting Summary

Nature Portfolio wishes to improve the reproducibility of the work that we publish. This form provides structure for consistency and transparency in reporting. For further information on Nature Portfolio policies, see our Editorial Policies and the Editorial Policy Checklist.

Statistics

For all statistical analyses, confirm that the following items are present in the figure legend, table legend, main text, or Methods section.

n/a	Confirmed	
☐	☒	The exact sample size (*n*) for each experimental group/condition, given as a discrete number and unit of measurement
☐	☒	A statement on whether measurements were taken from distinct samples or whether the same sample was measured repeatedly
☐	☒	The statistical test(s) used AND whether they are one- or two-sided<br>*Only common tests should be described solely by name; describe more complex techniques in the Methods section.*
☐	☒	A description of all covariates tested
☐	☒	A description of any assumptions or corrections, such as tests of normality and adjustment for multiple comparisons
☐	☒	A full description of the statistical parameters including central tendency (e.g. means) or other basic estimates (e.g. regression coefficient) AND variation (e.g. standard deviation) or associated estimates of uncertainty (e.g. confidence intervals)
☐	☒	For null hypothesis testing, the test statistic (e.g. *F*, *t*, *r*) with confidence intervals, effect sizes, degrees of freedom and *P* value noted<br>*Give P values as exact values whenever suitable.*
☒	☐	For Bayesian analysis, information on the choice of priors and Markov chain Monte Carlo settings
☒	☐	For hierarchical and complex designs, identification of the appropriate level for tests and full reporting of outcomes
☐	☒	Estimates of effect sizes (e.g. Cohen's *d*, Pearson's *r*), indicating how they were calculated

Our web collection on statistics for biologists contains articles on many of the points above.

Software and code

Policy information about availability of computer code

Data collection	Flow cytometry data was colleacted LSRII (BD Biosciences) cytometers using BD DIVA 8.0.1 software (BD Biosciences).<br>Imaging Flow cytometry was done on an Amnis Flowsight cytometer (Luminex Corporation, TX) using INSPIRE software v6.5 (Luminex Corporation, TX). Flow sorting was done on a BD FACS ARIA II.<br>Cytospins were obtained with ShandonTM Cytospin3 and imaged with a Zeiss Axioskop 40 microscope using a SPOT Flex Camera (Diagnostic Instruments, Inc.)<br>Quantitative PCR was performed using the ABI 7300 sequence detection system
Data analysis	Amnis data was analyzed using IDEAS software v6.0 (Luminex Corporation, TX).<br>Flow cytometry data were analyzed using FlowJo v9 (v9.2 to v9.9) and v10.<br>Cytospin images were analyzed with the SPOT v.5.6 software (SPOT Imaging)<br>Colony area was measured using ImageJ version: 2.0.0-r-54/1.51h<br>Data was analyzed and plotted using Microsoft Excel versions 2013, 2016 and 2019; GraphPad Prism Version 9.2.0; RStudio Version 1.2.1335 © 2009-2019 RStudio, Inc; Python 3<br>Human intervention study data was anayzed using the linear mixed-effect models using the nlme package<br>The MCV simulation python script is deposited in https://github.com/socolovm/Simulation-of-MCV.

For manuscripts utilizing custom algorithms or software that are central to the research but not yet described in published literature, software must be made available to editors and reviewers. We strongly encourage code deposition in a community repository (e.g. GitHub). See the Nature Portfolio guidelines for submitting code & software for further information.

Data

Policy information about availability of data

All manuscripts must include a data availability statement. This statement should provide the following information, where applicable:

- Accession codes, unique identifiers, or web links for publicly available datasets
- A description of any restrictions on data availability
- For clinical datasets or third party data, please ensure that the statement adheres to our policy

Source Data is provided with this paper for Figures 1 to 6 and for Supplementary Figures 2, 4 to 13. Complete Blood Count source data for the human studies is provided in the 'Statistical analysis of hematological parameters' supplementary information. Additional flow cytometry data is available upon request.

Field-specific reporting

Please select the one below that is the best fit for your research. If you are not sure, read the appropriate sections before making your selection.

- [x] Life sciences
- [ ] Behavioural & social sciences
- [ ] Ecological, evolutionary & environmental sciences

For a reference copy of the document with all sections, see nature.com/documents/nr-reporting-summary-flat.pdf

Life sciences study design

All studies must disclose on these points even when the disclosure is negative.

Sample size	All relevant sample sizes are described in the legend to each figure and/or in the materials and methods section. Sample sizes were determined based on previous experience with each of the experimental systems. Statistical tests were not used to pre-determine sample size.
Data exclusions	No data was excluded from analysis
Replication	At least two independent experiments were performed for each data panel in the manuscript figures. All reported data were reproduced reliably.
Randomization	Experiments with mice or harvested mouse tissue entailed manipulating very similar primary cell samples, derived from inbred mice of similar age, sex and weight, making randomization not applicable. Human subjects in the human studies were randomized into either experimental or treatment groups. rhEPO treatment: The studies 1 and 2 used a randomized single-blinded placebo-controlled design. Phlebotomy (study 3) used a randomized single-blinded placebo-controlled crossover design.
Blinding	Experiments with mice or harvested mouse tissue did not employ blinding since readouts were quantitative and not prone to subjective judgment of investigators. Human studies were single-blinded design.

Reporting for specific materials, systems and methods

We require information from authors about some types of materials, experimental systems and methods used in many studies. Here, indicate whether each material, system or method listed is relevant to your study. If you are not sure if a list item applies to your research, read the appropriate section before selecting a response.

Materials & experimental systems

n/a	Involved in the study	
[ ]	[x]	Antibodies
[ ]	[x]	Eukaryotic cell lines
[x]	[ ]	Palaeontology and archaeology
[ ]	[x]	Animals and other organisms
[ ]	[x]	Human research participants
[x]	[ ]	Clinical data
[x]	[ ]	Dual use research of concern

Methods

n/a	Involved in the study	
[x]	[ ]	ChIP-seq
[ ]	[x]	Flow cytometry
[x]	[ ]	MRI-based neuroimaging

Antibodies

Antibodies used	Antibodies used:<br>PE Mouse Anti-Human CD4 (RPA-T4) (BD Biosciences) dilution 1:50<br>PE/Cy7 Rat Anti-Mouse CD71 (RI7217) (BioLegend) dilution 1:100<br>APC /Cyanine7 Rat Anti-Mouse Ter119 (Ter119) (BioLegend) dilution 1:100<br>PE Rat Anti-Mouse Ter119 (Ter119) (BD Biosciences) dilution 1:100

	APC Rat Anti-Mouse Ter119 (Ter119) (BD Biosciences) dilution 1:100<br>biotin Rat Anti-Mouse CD71 (C2) (BD Biosciences) dilution 1:100<br>biotin Rat Anti-Mouse Ter119 (BD Biosciences) dilution 1:100<br>biotin Rat Anti-Mouse Ly-6G and Ly-6C/Gr1 (RB6-8C5) (BD Biosciences) dilution 1:100<br>biotin Rat Anti-Mouse CD11b/Mac1 (M1/70) (BD Biosciences) dilution 1:100<br>biotin Rat Anti-Mouse CD41 (MWReg30) (Thermo Scientific) dilution 1:100<br>FITC Rat Anti-Mouse Ly-6G and Ly-6C/Gr1 (RB6-8C5) (BD Biosciences) dilution 1:100<br>FITC Rat Anti-Mouse CD11b/Mac1 (M1/70) (BD Biosciences) dilution 1:100<br>FITC Rat Anti-Mouse CD41 (MWReg30) (BD Biosciences) dilution 1:100<br>FITC Rat Anti-Mouse CD45R/B220 (RA3-6B2) (BD Biosciences) dilution 1:100<br>FITC Hamster Anti-Mouse CD3e (145-2C11) (BD Biosciences) dilution 1:100<br>PE Rat Anti-Mouse Ly-6G and Ly-6C/Gr1 (RB6-8C5) (BioLegend) dilution 1:100<br>PE Rat Anti-Mouse CD11b/Mac1 (M1/70) (BioLegend) dilution 1:100<br>PE Rat Anti-Mouse CD41 (MWReg30) (BD Biosciences) dilution 1:100<br>PE Rat Anti-Mouse CD45R/B220 (RA3-6B2) (BD Biosciences) dilution 1:100<br>PE Hamster Anti-Mouse CD3e (500A2) (BioLegend) dilution 1:100
Validation	All antibodies were validated in our laboratory. This involve appropriate negative and positive controls. hCD4 reporter of retroviral infection was validated with un-infected fetal liver cells. CD71 and Ter119, both markers of specific stages of erythroid terminal differentiation, were validated by ensuring that they show the appropriate pattern of staining, with proerythroblasts as positive controls for CD71 and late erythroblasts/red cells as positive controls for Ter119. We relied on the manufacturer's validation for all Lin+ antibodies, which include B220, Mac1, Gr1, CD41, CD3e.

Eukaryotic cell lines

Policy information about cell lines

Cell line source(s)	We used the Phoenix ecotropic retroviral packaging cell line, obtained from the Baltimore laboratory (from Warren Pear) approxiamtely 20 years ago. This line had been passaged in our laboratory since.
Authentication	We regularly ensue that there is no replication competent virus generated by the line. We carry out no other authentication, other than ensure that high titre replication defective retroviral supernatant is generated when the line is transduced with the relevant packaging vectors.
Mycoplasma contamination	We have not tested this line for mycoplasma contamination.
Commonly misidentified lines<br>(See ICLAC register)	No commonly misidentified cell lines were used in this study

Animals and other organisms

Policy information about studies involving animals; ARRIVE guidelines recommended for reporting animal research

Laboratory animals	For all fetal liver experiments we used embryos at embryonic age 12.5 days (E12.5). Strain: Balb/C. Sex: we have not determined the sex of the embryos. The Epo/Saline injection experiment on adult mice were conducted on male C57BL6 fluorescence timer (FT) transgenic mice. Mice are housed at a dedicated facility, with regulated enviroment, temperature in the range 20 to 26°C, a 12 h/12 h dark /light cycle and 30 to 70% humidity. They are fed on Iso Pro 3000 irradiated rodent diet #5P76.
Wild animals	No wild animals were used in this work
Field-collected samples	Not Applicable.
Ethics oversight	All experiments were conducted in accordance with animal protocol A-1586 approved by the University of Massachusetts Medical School Institutional Animal Care and Use Committee.

Note that full information on the approval of the study protocol must also be provided in the manuscript.

Human research participants

Policy information about studies involving human research participants

Population characteristics	Young (18-50 yrs) healthy, normal weight, non-smoking Caucasian males and females with an active life style.<br>Study #1, males n=19 – age: 24 ± 2 years (mean ± SD)<br>females n=15 – age: 25 ± 3 years<br>combined age: 25 ± 3 years<br>Study #2: All participants were male, age was 33.5 years (20-50) in the placebo group and 33.5 (22-48) in the EPO group<br>Study #3: males n=21 age: 29 ± 6 years
Recruitment	Participants were recruited via advertising on social media, dedicated web-pages and flyers.<br>There is a potential selection bias towards healthier than average participants since the studies examined the effect of Epo on

athletic performance. This appears unlikely to influence the results.

Ethics oversight	Human studies #1 and #3 were conducted in Copenhagen, Denmark according to all applicable national and international rules and regulations including the Helsinki II declaration. Ethics approval letters for the studies (protocol numbers H-2-2014-109 & H-17036662) were granted by the Regional Branch (Copenhagen Region) of the Danish National Committee on Health Research Ethics (https://en.nvk.dk/). All participants were informed both orally and in writing of potential risks and discomforts associated with participation before written consent was obtained.

Note that full information on the approval of the study protocol must also be provided in the manuscript.

Flow Cytometry

Plots

Confirm that:

- [x] The axis labels state the marker and fluorochrome used (e.g. CD4-FITC).
- [x] The axis scales are clearly visible. Include numbers along axes only for bottom left plot of group (a 'group' is an analysis of identical markers).
- [x] All plots are contour plots with outliers or pseudocolor plots.
- [x] A numerical value for number of cells or percentage (with statistics) is provided.

Methodology

Sample preparation	Fresh or cultured fetal liver cells were mechanically dissociated and labeled in 'staining medium' (phosphate-buffered saline containing 0.2% bovine serum albumin and 200 microgram/ml rabbit IgG) and specific antibodies at the indicated concentrations.
Instrument	BD LSRII or BD FACS ARIA II
Software	BD DIVA 8.0.1 software (BD Biosciences)
Cell population abundance	Abundance was between 5 and 50%, depending on the specific population. All purities were >80%
Gating strategy	FSC/SSC, singlets, viability gate, Lin- gating, and in most experiments this was followed by gating on the reporter of retroviral transduction, either hCD4-PE or GFP

- [x] Tick this box to confirm that a figure exemplifying the gating strategy is provided in the Supplementary Information.
